# A network-based method using a random walk with restart algorithm and screening tests to identify novel genes associated with Menière's disease

**DOI:** 10.1371/journal.pone.0182592

**Published:** 2017-08-07

**Authors:** Lin Li, YanShu Wang, Lifeng An, XiangYin Kong, Tao Huang

**Affiliations:** 1 Department of Otorhinolaryngology and Head & Neck, China-Japan Union Hospital of Jilin University, Changchun, China; 2 Department of Anesthesia, The First Hospital of Jilin University, Changchun, China; 3 Institute of Health Sciences, Shanghai Institutes for Biological Sciences, Chinese Academy of Sciences, Shanghai, China; Tianjin University, CHINA

## Abstract

As a chronic illness derived from hair cells of the inner ear, Menière’s disease (MD) negatively influences the quality of life of individuals and leads to a number of symptoms, such as dizziness, temporary hearing loss, and tinnitus. The complete identification of novel genes related to MD would help elucidate its underlying pathological mechanisms and improve its diagnosis and treatment. In this study, a network-based method was developed to identify novel MD-related genes based on known MD-related genes. A human protein-protein interaction (PPI) network was constructed using the PPI information reported in the STRING database. A classic ranking algorithm, the random walk with restart (RWR) algorithm, was employed to search for novel genes using known genes as seed nodes. To make the identified genes more reliable, a series of screening tests, including a permutation test, an interaction test and an enrichment test, were designed to select essential genes from those obtained by the RWR algorithm. As a result, several inferred genes, such as *CD4*, *NOTCH2* and *IL6*, were discovered. Finally, a detailed biological analysis was performed on fifteen of the important inferred genes, which indicated their strong associations with MD.

## Introduction

Menière’s disease (MD) is a disorder that involves the inner ear with various episodic symptoms, including vertigo, hearing loss, tinnitus and ear fullness, and it is a frustrating condition with a sensation of pressure in the middle ears [1, 2]. In most patients, MD only affects one ear and may induce at least two to three of the symptoms mentioned above, in contrast to other problems in the ear [3].

In the clinic, MD has specific differential diagnosis standards. Two tests have been widely applied in the diagnostic processes, involving two explicit symptoms of MD. A hearing test is one of the most significant diagnostic methods that has been widely applied for preliminary screening, and it tests whether the patients can hear sounds with different pitches and volumes and can tell the difference between similar sounds [4–6]. A specific test item, named electrocochleography (ECog), is a standard testing method that distinguishes neuropathic and functional hearing disorders and is usually accompanied by an auditory brainstem response test [7, 8]. After the hearing test, to target another specific symptom of MD, vertigo and balance tests are also generally applied for the diagnosis of MD. Balance tests contribute to the functional identification of inner ears. Considering that vertigo is a typical symptom of MD, the function of the inner ears may be disabled in such patients [9]. Generally, during the clinical diagnosis of MD, electronystagmography (ENG) is the most commonly used test and can detect inner ear induced eye movements [10]. In addition to ENG detection, another method, named the rotary chair, has been applied in clinical diagnosis and can reduce the false negative rate of a single test [11]. Other tests, such as the vestibular evoked myogenic potential (VEMP), magnetic resonance imaging (MRI) and computerized tomography (CT), have also been applied to differentially diagnose MD [12].

After the diagnosis of MD, three main treatment methods have been preferred to treat this disease. The first one is effective medication. As mentioned above, the pathogenesis of MD has not been fully revealed, and the usual treatments for such a disease focus on relieving clinical symptoms, such as hearing loss and vertigo [13, 14]. The medicine that may be applied to treat this disease usually provides relief of certain symptoms but does not treat the causes. Therefore, the drug choices rely mainly on diverse symptoms. For example, if nausea and vomiting are main symptoms of MD patients, an antiemetic may be an optimal choice for treatment to avoid vomiting [15]. However, for the patients with severe vertigo symptoms, a diuretic, which contributes to the output of urine, may be the best choice for treatment [16]. In addition to drug therapy, hearing aids have been widely applied as a custom treatment method that contributes to the relief of hearing loss symptoms. With the integration of tutorials and hardware support, hearing aids contribute mainly to balancing the hearing ability in the two ears (healthy and ill), and this also only provides symptom relief [17]. Furthermore, surgery is an option for patients with severe hearing loss and vertigo, targeting the decrease of fluid in the inner ear and relieving specific symptoms [18].

As we have analyzed above, most of the diagnosis and treatment methods for clinical applications concentrate on relieving specific symptoms but do not address the pathogenesis of MD. For the potential pathogenesis of this disease, inflammation induced endolymphatic hydrops have been identified as a secondary pathogenesis of the disease, with the initial triggers remaining not fully understood [19]. Although the initial triggers of the disease have not been validated, various genes and variants have been confirmed to be related to MD, which also suggests a specific family genetic predisposition and implies that genetic factors may play an essential role in the initiation and progression of MD [20, 21]. There are three main groups of genes that have been confirmed to contribute to the initiation and progression of this disease. The first group contains immune-associated genes, particularly innate immune-associated genes that have been widely reported to contribute to the disease. Toll-like receptor coding genes, including TLR3, TLR7, TLR8 and TLR10, have all been directly confirmed to be related to the progression of MD, thus implying a specific role for the immune system during the pathological processes [22]. Apart from immune associated genes, water and ion channel protein coding genes and their regulatory factors have also been widely reported to participate in the pathogenesis. AQP2, AQP4 and AQP5 are three representative water channel protein coding genes that are related to MD and may directly contribute to hearing impairment, deafness, and the severe complications during the pathological processes [23]. In addition, proliferation- and cell survival-associated genes have also been widely reported to contribute to the disease. The NOTCH and NF-KB signaling pathways have been confirmed to be abnormally regulated by specific variants during the initiation and progression of this disease, implying an abnormal proliferation of certain cell subtypes that may also contribute to the pathogenesis [24]. Apart from these three subgroups of genes, functional genes such as COCH and the DFNA family that do not belong to a particular functional category participating in the initiation and progression of MD, thus validating the irreplaceable role of a genetic background for MD [25, 26].

As mentioned above, the genetic background has been confirmed to play a specific role during the initiation and progression of MD and related complications. Currently, the identification and validation of MD associated genes depend mainly on the genetic screening of clinical patients and their families. However, since the pathological mechanisms of such diseases are quite complicated and involve various aspects of the biological processes, it is quite difficult and time-consuming to identify each specific MD by experimental dependent genetic screening [27]. With the development of bioinformatics, some computational methods have been presented to contribute to the identification of similar disease associated genes [28] and other related problems [29–31]. Among them, a network-based method is an important type of computational method [32–37], such as Guilt-by-association (GBA)-based methods [38–40], the random walk with restart (RWR) algorithm [41–43], and the shortest path algorithm [43–51]. This study also built a network-based method to identify novel MD-related genes. A protein-protein interaction (PPI) network was constructed using the PPI information retrieved from the STRING database [52]. Then, the RWR algorithm was applied to the network to search for possible genes by setting known MD-related genes as seed nodes. Furthermore, a series of screening tests, including a permutation test, an interaction test and an enrichment test, were designed to pick out essential genes from the genes yielded by the RWR algorithm. Several inferred genes were produced and were deemed to be closely related to MD. A biological analysis of fifteen important inferred genes was performed, validating their strong relationships with MD and uncovering the potential molecular processes that these genes may participate in.

## Materials and methods

### 2.1 Materials

MD-related genes were collected from the literature indexed by PubMed (http://www.ncbi.nlm.nih.gov/pubmed/). The keywords “Menière’s disease” and “gene” were used to search the literature in PubMed, resulting in 120 papers (January, 2017). Among these papers, 72 papers reported novel MD associated genes, from which we accessed 84 genes, which are provided in S1 Table. According to these screened out papers, there are three principal methods that have been applied to identify MD-associated genes: (I) sequencing (either high-throughput sequencing or Sanger sequencing) together with pedigree analysis in MD families; (II) in situ immune-histochemical localization of target genes and gene products of clinical samples; and (III) in vitro cloning and expression of target genes in proper models together with functional validation. Because we used the PPI network reported in the STRING database, in which Ensembl IDs were adopted to represent proteins, all 84 genes were mapped to their Ensembl IDs, producing 106 Ensembl IDs, which are also provided in S1 Table. These Ensembl IDs were used to search for novel MD-related genes.

### 2.2 PPI network

Many proteins participating in intracellular and intercellular biological processes are always formed as protein complexes to execute their normal functions, such as the functionally active hemoglobin molecule, which is composed of four subunits, each of which is a protein monomer that has its own tertiary structure [53]. Furthermore, proteins that can form a PPI always share related functions or co-locate in same metabolism pathways [54–59]. Therefore, some useful information can be mined from the set of PPIs, which can uncover properties, functions and interaction relationships of proteins. Based on PPI information, several computational methods have been built to predict the properties of proteins, such as protein functions [56–58, 60], disease genes [61–65], and protein phenotypes [66]. Thus, we also adopted PPI information to infer the novel genes associated with MD in this study.

The STRING database [52] is a well-known public database for both direct (physical) and indirect (functional) PPIs that are derived from (1) genomic contexts, (2) high-throughput experiments, (3) (conserved) co-expression, and (4) previous knowledge. It is easy to see that these PPIs can widely measure the associations between proteins. They were employed in this study to construct the network. All PPIs covering 1,133 organisms in STRING were collected in a file labeled ‘protein.links.v9.1.txt.gz’, from which we extracted 2,425,314 human PPIs involving 20,770 proteins. Each PPI contains two proteins, represented by Ensembl IDs, and one score that indicates the strength of the interaction. For two given proteins *p*_*a*_ and *p*_*b*_, the score of the interaction between them was formulated as *S*(*p*_*a*_, *p*_*b*_), with a larger score value meaning that an interaction between the proteins was more likely to occur.

The constructed network, denoted as *G*, defined 20,770 proteins as nodes, and each edge in *G* represented a human PPI; *i*.*e*., two nodes were connected if and only if their corresponding proteins were composed of a PPI reported in STRING. In addition, to indicate different roles of edges in *G*, each edge was assigned a weight that was defined as the score of its corresponding PPI.

### 2.3 RWR algorithm

The RWR algorithm [41] is a type of ranking algorithm. It has been deemed a useful tool to expand novel objects from known ones. This algorithm always simulates a random walker starting from a seed node or a set of seed nodes, representing known objects, and it calculates the probability of each node being a novel object. For the identification of novel genes associated with MD, the known genes mentioned in Section 2.1 were deemed to be seed nodes, on which the RWR algorithm would be used to infer novel ones. The RWR algorithm repeatedly updated a probability vector *P*_*i*_ that contained 20,770 components, each of which indicated the probability of a node in *G* being a novel MD gene. In the initialization of the RWR algorithm, *P*_0_ was constructed by setting the components of the corresponding seed nodes to 1/106 and the others to zero. The subscript of *P*_*i*_ represents the number of loops that had been run; *i*.*e*., *P*_*i*_ representing the probabilities after the *i*-th round of the loop had been run. *P*_*i*_ can be updated by the following formula:

$$P_{i+1}=(1-c)A^{T}P_{i}+cP_{0}$$ (1)

where *A* was the column-normalized adjacency matrix of *G*, and *c* was the restart probability (it was set to 0.8 in this study to indicate the importance of known MD genes). The loop stopped when **||** *P*_*i*+1_ –*P*_*i*_ **|| <** 1E-06 [41], indicating the probability vector was stable. The probability vector *P*_*i*+1_ was output as the outcome of the RWR algorithm.

Based on the outcome of the RWR algorithm, each node received a probability of being a novel gene associated with MD. A higher probability meant that the corresponding gene was more likely to relate to MD. For wide detection, we set the threshold of 1E-05 to the probability; *i*.*e*., genes with output probabilities larger than 1E-05 were selected as possible genes. For convenience, we called them RWR genes in the following context.

### 2.4 Screening tests

After the RWR algorithm was executed on the PPI network mentioned in Section 2.3, some RWR genes with probabilities higher than 1E-05 were found. However, there may be false positives among them, so this section presents a series of screening tests to control for this possibility, thereby obtaining the most related genes.

#### Permutation test

It is clear that the utility of the RWR algorithm is strongly based on the PPI network. The topological structure of the PPI network may cause the selection of some false positives. Obviously, these types of RWR genes are not closely related to MD. To exclude these genes, a permutation test [64, 67, 68] was utilized. First, 1,000 Ensembl ID sets (namely, *S*_1_, *S*_2_,…, *S*_1000_) were constructed, and each of the sets consisted of 106 randomly selected Ensembl IDs from the network. Second, for each set, the RWR algorithm was applied on the PPI network with Ensembl IDs in the set as seed nodes, thus providing a probability for each RWR gene. Finally, a measurement called the p-value was calculated for each RWR gene based on the probability yielded by the RWR algorithm on 106 MD associated genes and 1,000 probabilities yielded by the RWR algorithm on 1,000 randomly produced sets. It can be computed by

p−value(g)=Θ/1000 (2)

where Θ is the number of randomly produced Ensembl ID sets on which the probability of the RWR gene *g* is higher than that of the 106 MD associated genes. It is clear that RWR genes with high p-values are not special for MD because they can be produced by several randomly produced sets. According to the widely accepted significance level in statistical analysis, 0.05 was used as the threshold of the p-value; *i*.*e*., RWR genes with p-values greater than or equal to 0.05 were screened out. The remaining RWR genes were called candidate genes, which would be further checked by the tests mentioned below.

#### Interaction and enrichment test

The purpose of this study was to identify novel genes associated with MD. Among the candidate genes, some had a strong association with MD, while others had a weak association. To mine the most related candidate genes, two tests, namely, the interaction test and the enrichment test, were built in this section to directly or indirectly measure the association between the candidate genes and MD.

The first test was built based on the PPI information mentioned in Section 2.2. It has been widely accepted that two proteins that can interact with each other are more likely to share related functions. Thus, candidate genes that can interact with at least one MD associated gene are more likely to be novel MD associated genes. For each candidate gene *g*, a measurement, namely, the maximum interaction score (*MIS*), was computed by

MIS(g)=max{S(g,g′):g′ is a MD-related gene} (3)

where *S*(*g*, *g*′) represents the interaction score of *g* and *g*′. Clearly, candidate genes with high *MIS*s can interact with an MD-related gene with a high probability, implying they may be novel MD-related genes. Because 900 is set to be the cutoff of highest confidence in STRING, it was also set to be the threshold of *MIS*; *i*.*e*., candidate genes with *MIS*s greater than or equal to 900 were selected.

Gene ontology (GO) [69] can clearly describe a given gene and its product based on three aspects: molecular function, biological process, and cellular component. On the other hand, the Kyoto Encyclopedia of Genes and Genomes (KEGG) [70] provides many biological pathways that include several genes. The second test was built based on the GO terms and KEGG pathways of candidate genes and MD-related genes. It is clear that the MD associated genes must be related to some common GO terms and KEGG pathways. Additionally, some GO terms and KEGG pathways have no relationship with these MD associated genes. If a candidate gene exhibits a similar relationship with GO terms and KEGG pathways to those of MD-related genes, it is more likely to be a novel MD-related gene. According to the enrichment theory of GO terms and KEGG pathways [30, 31, 71, 72], the relationship between a gene *g* and GO terms or KEGG pathways can be encoded as a numeric vector, denoted by *FV*(*g*). The proximity of two genes *g* and *g*′ on GO terms and KEGG pathways can be measured by the direction cosine of vectors *FV*(g) and *FV*(*g*′), which can be formulated as

$$MFS(g)=\frac{FV(g)\cdot FV(g\prime )}{\left\|FV(g)\right\|\cdot \left\|FV(g\prime )\right\|}$$ (4)

According to the arguments mentioned above, for each candidate gene, we should measure its relationship to all MD-related genes in this regard and take the maximum to imply its association with MD. Thus, another measurement, namely, the maximum enrichment score (*MES*), was calculated for each candidate gene *g*, which was defined by:

MES(g)=max{Γ(g,g′):g′ is a MD-related gene} (5)

Obviously, a larger *MES* means that several overlapping GO terms and KEGG pathways are shared by the candidate gene *g* and an MD-related gene. A threshold of 0.8 was set for the *MES* in this study; *i*.*e*., candidate genes with *MES*s larger than 0.8 were selected.

Of the candidate genes filtered by the permutation test, those with *MIS*s greater than or equal to 900 and *MES*s larger than 0.8 were finally selected. They were deemed to be of special interest for MD. For convenience, they are called inferred genes.

## Results

**Fig 1:**
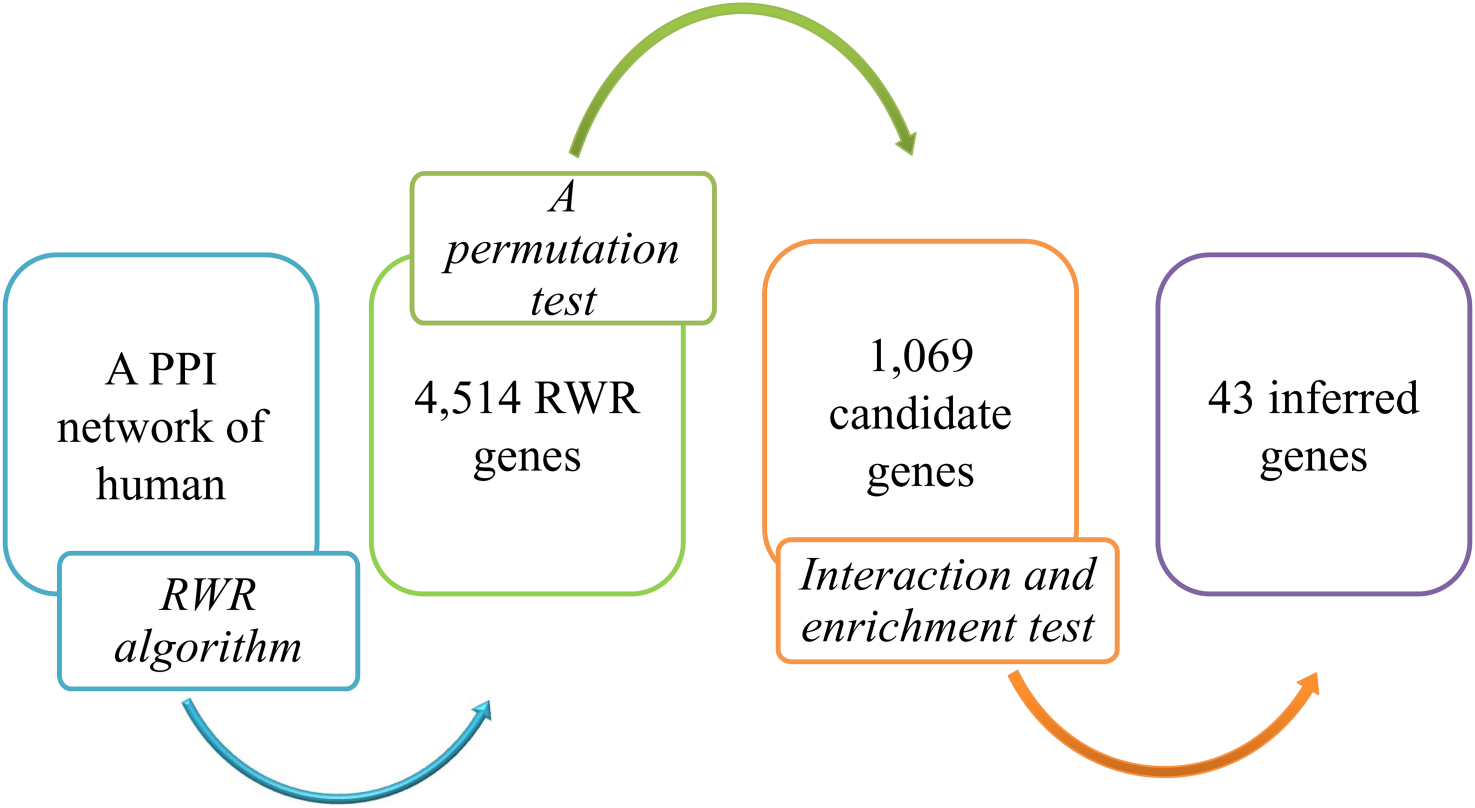
The flowchart of the network-based method to identify novel MD-related genes.

To clearly illustrate all procedures of the network-based method for the identification of genes associated with MD, a flowchart is shown in Fig 1. This section shows the detailed results yielded by the different procedures of this method.

The RWR algorithm was applied on the PPI network constructed in Section 2.2 by setting the 106 Ensembl IDs of MD-related genes as seed nodes. As a result, each node in the network was assigned a probability of it being a novel MD-related gene. 1E-05 was set as the threshold of probability, resulting in 4,514 RWR genes. These genes, together with the probabilities yielded by the RWR algorithm, are listed in S2 Table.

As mentioned above, a large number of RWR genes were found by the RWR algorithm. However, some false positives were selected due to the structure of the network. In fact, they have little relationship with MD. Thus, the permutation test mentioned in Section 2.4 was applied to evaluate each RWR gene. A measurement called the p-value was calculated for each RWR gene, which is listed in S2 Table. Genes with p-values less than 0.05 were selected, producing 1,069 candidate genes. Compared with the RWR genes, the candidate genes are more likely to relate to MD. The 1,069 candidate genes are provided in S3 Table.

**Fig 2:**
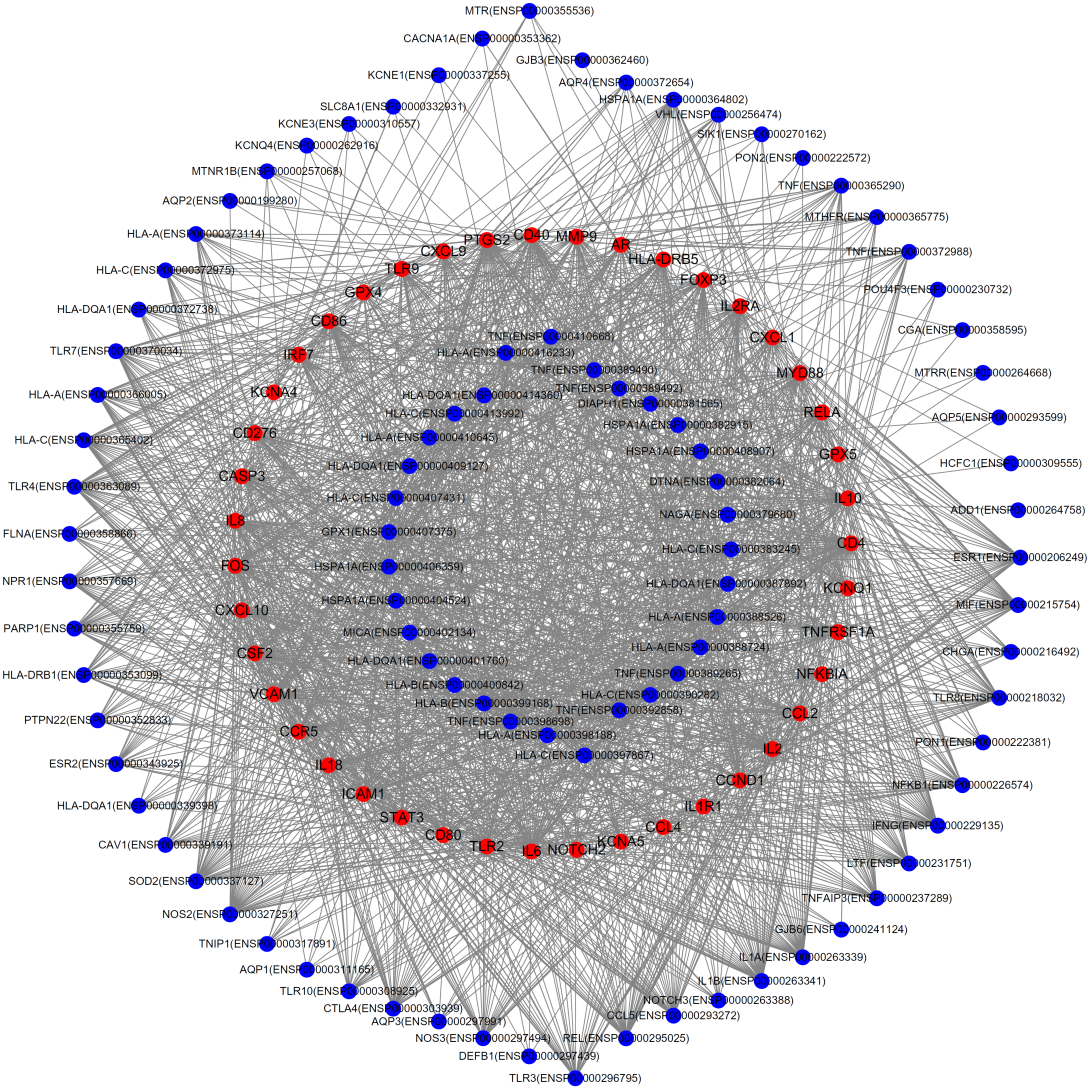
The relationship between the inferred genes and known MD-related genes in the PPI network. Red nodes represent inferred genes, and blue nodes represent known MD genes.

For the 1,069 candidate genes, an interaction test and an enrichment test were used to further evaluate each candidate gene and select the most important candidate genes among them. Two measurements: *MIS* and *MES* (cf. Eqs 3 and 5), were yielded by these two tests. Each of the 1,069 candidate genes received these two measurements, with the results provided in S3 Table. Values of 900 and 0.8 were set to be the thresholds of *MIS* and *MES*, respectively, yielding the 43 inferred genes listed in S4 Table. These 43 genes are deemed to be tightly associated with MD. To partly elaborate this fact, a bipartite subgraph of the PPI network, using inferred and known MD-related genes as nodes, is shown in Fig 2, which indicates that each inferred gene is closely related to at least one known gene, indicating its close relationship with MD. The interactions used in Fig 2 are available in S5 Table.

## Discussion

Relying on the network-based method, we identified 43 functional genes that may participate in MD associated biological processes. As described in Section 1, three specific biological processes have been widely reported to participate in MD: (1) immune associated biological processes; (2) cell surface channel associated processes, and (3) proliferation and cell survival associated biological processes, involving different groups of functional genes. From the 43 inferred genes, we chose fifteen for our analysis, which are listed in Table 1. These genes have been shown to contribute to two of the aforementioned three biological processes according to recent publications.

**Table 1:** The details of fifteen important inferred genes.

Ensembl ID	Gene symbol	Description	Probability ^a^	P-value ^b^	MIS (most related MD-related gene) ^c^	MFS (most related MD-related gene) ^d^
ENSP00000258743	IL6	Interleukin 6	3.62E-04	0.004	992 (IL1B)	0.920 (TNF)
ENSP00000353874	TLR9	Toll Like Receptor 9	8.65E-05	<0.001	927 (TLR3)	0.895 (TLR7)
ENSP00000305651	CXCL10	C-X-C Motif Chemokine Ligand 10	1.05E-04	0.002	994 (CCL5)	0.891 (CCL5)
ENSP00000392398	GPX5	Glutathione Peroxidase 5	8.36E-05	0.002	919 (SOD2)	0.890 (GPX1)
ENSP00000260010	TLR2	Toll Like Receptor 2	1.80E-04	<0.001	964 (TNF)	0.888 (TLR4)
ENSP00000346103	GPX4	Glutathione Peroxidase 4	4.93E-05	0.037	919 (SOD2)	0.886 (GPX1)
ENSP00000379625	MYD88	Myeloid Differentiation Primary Response 88	1.37E-04	0.001	999 (TLR4)	0.880 (TLR4)
ENSP00000354901	CXCL9	C-X-C Motif Chemokine Ligand 9	6.37E-05	0.029	986 (CCL5)	0.874 (CCL5)
ENSP00000011653	CD4	CD4 Molecule	3.11E-04	0.003	998 (HLA-DRB1)	0.870 (IFNG)
ENSP00000256646	NOTCH2	Notch 2	6.06E-05	0.038	905 (NOTCH3)	0.869 (NOTCH3)
ENSP00000233946	IL1R1	Interleukin 1 Receptor Type 1	8.11E-05	0.015	999 (IL1B)	0.858 (TLR3)
ENSP00000280357	IL18	Interleukin 18	1.21E-04	0.002	994 (IFNG)	0.852 (TLR4)
ENSP00000412237	IL10	Interleukin 10	2.11E-04	<0.001	976 (TNF)	0.851 (TLR4)
ENSP00000356438	PTGS2	Prostaglandin-Endoperoxide Synthase 2	1.94E-04	0.007	976 (IL1B)	0.847 (IL1B)
ENSP00000225831	CCL2	C-C Motif Chemokine Ligand 2	1.46E-04	0.006	968 (TNF)	0.841 (TLR4)

^a^: The value in this column is obtained by the RWR algorithm. A high probability means the corresponding gene is more likely to relate to MD.

^b^: The value in this column is obtained in the permutation test (cf. Eq 2). A low value means the corresponding gene is special for MD.

^c^: The value in this column is obtained in the interaction test (cf. Eq 3). A high value indicates the corresponding gene is more likely to be a novel MD-related gene.

^d^: The value in this column is obtained in the enrichment test (cf. Eq 5). A high value indicates the corresponding gene is more likely to be a novel MD-related gene.

### 4.1 Immune associated genes

Among the fifteen genes listed in Table 1, eleven genes were shown to contribute to immune associated biological processes. The specific gene **CD4 (ENSP00000011653)** is a potential pathological gene for MD. As a membrane glycoprotein of T lymphocytes, CD4 mainly contributes to MHC class-II antigen/T-cell receptor interactions, regulating the activation of T cells [73]. Based on recent publications, the interactions between MHC II molecules of antigen presenting cells (APCs) and specific T cell receptors have been confirmed to contribute to MD associated immune reactions [74]. During the initiation and progression of MD, it has been reported that the proportion of CD4+ T cells have been increased, indicating the specific function of such interactions for MD [74]. Further, CD4 molecules have been reported to participate in allergy associated biological processes [75]. Because allergy is definitely associated with MD, it is quite reasonable to conclude that CD4 may be a potential MD-related gene [76, 77].

Apart from CD4, there are three genes encoding functional components of the interleukin family, together with one specific gene encoding a respective receptor. **IL6 (ENSP00000258743)**, as the abbreviation of interleukin 6, is one member of the interleukin family that may contribute to the initiation and progression of MD. As a specific functional cytokine, it mainly participates in the regulation of immune responses, hematopoiesis, platelet production and bone resorption [78–81]. Although few publications have revealed the relationships between IL6 and MD, a recent publication confirmed that during the pathological processes of MD, the serum level of IL6 together with IL1 is directly related to the specific complications of MD; e.g., vertigo, implying the underlying relationships between IL6 and MD [82]. Apart from IL6, IL1 may also contribute to MD in similar ways. **IL-1R1 (ENSP00000233946)**, encoding a function component of the IL1 receptor complex, has also been predicted to be an inferred gene that may participate in MD. Binding to interleukin-1, which just has been confirmed to contribute to the pathogenesis of MD, such genes mainly participate in the regulation of IL-1 associated activation of NF-kappa-B, MAPK and other functional signaling pathways [83–85]. There are still no direct interactions that can be revealed between IL-1R1 and MD. However, a recent publication confirmed that genes and proteins that contribute to interleukin-1 signaling pathways may be related to a specific clinical symptom of MD, *i*.*e*., sudden sensorineural hearing loss [86]. Another component of the interleukin family that has also been screened to be an inferred gene is **IL10 (ENSP00000412237)**. IL10 is a cytokine synthesis inhibitory factor and a co-stimulator for the proliferation and differentiation of T and B mast cells [87, 88]. For the relationships between IL10 and MD, although no direct relationships between such genes and the disease have been reported, a specific report on autoimmune hearing loss validated that the abnormal secretion of IL-10 may contribute to specific hearing loss symptoms of experimental autoimmune hearing loss [89]. Because hearing loss is a typical symptom of MD, which is also widely considered an autoimmune disorder, it is quite reasonable that IL10 may also participate in the pathological processes of MD [89, 90]. Another gene **IL18 (ENSP00000280357)** has been widely reported to contribute to the Th-1 mediated cellular immunity and may stimulate interferon gamma production in Th-1 cells [91]. According to recent publications, it has been confirmed that specific Th-1 mediated immunological responses may be associated with sensorineural hearing loss and MD, implying that as a key regulator of Th1 cells, IL18, may also be a specific MD-related gene [92]. Therefore, these four interleukins indicate that interleukins may definitely be a specific group of functional regulators during the initiation and progression of MD.

Three chemokines encoding genes have also been predicted as MD-related genes by our network-based method. **CCL2 (ENSP00000225831)**, as a functional chemokine that attracts monocyte and basophils, has been widely reported to participate in monocyte proliferation associated disease, such as psoriasis and rheumatoid arthritis [93, 94]. Although the gene CCL2 has no direct relationship with MD, a publication confirmed that the distribution and proliferation of monocytes are regulated by CCL2. Therefore, it may cause pathological processes in the human endolymphatic sac, thereby inducing vertigo, tinnitus and hearing loss [95]. Considering that the human endolymphatic sac associated pathological processes has been confirmed to be associated with MD, it is quite reasonable to suggest that CCL2 may also play an irreplaceable role in MD [96, 97]. For another chemokine-associated gene, **CXCL9 (ENSP00000354901)**, which has been widely reported to participate in the regulation of cell growth, movement or activation status was also identified as an inferred gene for MD [98–100]. Similar to CCL2, CXCL9 has been reported to participate in monocyte proliferation associated disease, including psoriasis [93]. CXCL9, as a specific monocyte-associated gene, may also participate in MD in a similar way to CCL2, as we analyzed above. For **CXCL10 (ENSP00000305651)**, similar to CCL2 and CXCL9, it gene has also been widely reported to contribute to the chemotactic regulation of monocytes and T lymphocytes, thus indicating its potential function during the pathological processes of MD [101, 102]. Apart from such potential regulatory mechanisms, recent publications have also confirmed that CXCL10 may directly contribute to immune mediated apoptosis in the ear, inducing human presbycusis, which has also been considered a severe complication of MD, thus implying the potential role of CXCL10 during the pathological processes of MD [103].

Furthermore, we also identified three functional components of the innate immune response. **TLR2 (ENSP00000260010)** is a member of the Toll-like receptor family, which mainly contributes to pathogen recognition and innate immune activation [104, 105]. Recruiting **MYD88 (ENSP00000379625)**, another inferred gene, TLR2 is mainly involved in the innate immunity against Gram positive bacteria [106, 107]. According to recent publications, the initiation and progression of MD have been widely confirmed to be associated with the innate immune system and bacterial infection [22, 76, 108]. Although no direct relationship between TLR2 and MD has been reported, considering the specific relationship between gram-positive bacteria and MD, it is quite reasonable to regard TLR2, which mediates innate immunity against gram-positive bacteria, as a potential MD-related gene [109, 110]. Encoding the downstream recruited functional component of TLR2, another inferred gene MYD88 may also be a candidate gene of MD [111]. Another innate immune associated gene, as the homologue of TLR2, **TLR9 (ENSP00000353874)** has also been screened out as an inferred gene. Different from TLR2, which always interacts with gram-positive bacteria, TLR9 is a nucleotide-sensing TLR that identifies unmethylated cytidine-phosphate-guanosine (CpG) dinucleotides [112, 113]. Recent publications have confirmed that polymorphisms in toll-like receptor (including TLR9) is related to MD, which indicates that TLR9 may be an MD-associated gene [22]. In addition, TLR9 has been confirmed to contribute to the recognition of auto-antigens and induce auto-immune inner ear disease with hearing loss, a specific complication of MD, thus validating its special role in MD [114].

### 4.2 Proliferation and cell survival associated genes

In Table 1, four specific genes were confirmed to contribute to the proliferation and cell survival of certain cell subtypes, inducing their special contributions to the pathological processes of MD. **NOTCH2 (ENSP00000256646)**, a member of the Notch family, mediates cell-cell interactions and contributes to the cell fate decisions of certain cell subtypes [115, 116]. No direct contributions of NOTCH2 have been made to the initiation and progression of MD. However, based on recent publications, it is quite interesting that the development of auditory hair cells may be quite significant for congenital MD, related to hearing loss and vertigo [117, 118]. Therefore, as the core regulator of auditory hair cells, NOTCH2 and its related biological processes may definitely participate in the pathological processes of MD [119]. Two specific glutathione peroxidase encoded genes, **GPX4 (ENSP00000346103)** and **GPX5 (ENSP00000392398)**, have also been predicted to be inferred MD-related genes. These two genes encode two functional glutathiones contribute to the catabolic pathway of activated oxygen species, free radical detoxification [120, 121]. Although they seem to be homologues, they can participate in quite different biological processes. GPX4 plays a functional role during the regulation of primary T cell responses against viruses [122, 123]. Considering the underlying relationship between MD and T cell mediated anti-virus immune response, which has been widely reported, it is quite reasonable to regard GPX4 as a potential MD-related gene [124–126]. For GPX5, such genes protect cells and enzymes from oxidative damage, especially in the sperm membrane lipids [127, 128]. Considering that oxidative stress and damage have been widely reported to contribute to the pathogenesis of MD, as a regulator and protector against oxidative damage, GPX4 may contribute to MD [129–131]. Another inferred gene, **PTGS2 (ENSP00000356438)**, has also been predicted to be an inferred MD-related gene. Also known as cyclooxygenase, this gene mainly contributes to the biosynthesis of prostaglandin as both dioxygenase and peroxidase [132]. As a hormone regulator, PTGS2 contributes to the synthesis of prostaglandin, regulating its specific biological functions [132, 133]. According to recent publications, prostaglandin, which is regulated and synthesized by PTGS2, has a direct relationship with fluctuating hearing loss, a typical symptom of MD, showing the underlying interactions between PTGS2 and MD [134].

Based on the above analysis of fifteen inferred genes, they directly or indirectly participate in the biological processes associated with MD, implying high probabilities of them being novel MD-related genes. For the rest of the inferred genes, we did not discuss our analysis in this report and only provided this in S4 Table. Interested investigators can perform further validations.

## Conclusions

In this study, a network-based method was built to predict putative genes related to MD. Forty-three inferred genes were obtained that could play important roles in the pathogenesis of MD. These newly obtained genes, together with the already-known genes, may not only broaden the scope of known MD genes in human but also clarify the potential pathogenic mechanisms of MD. Furthermore, they also shed light on the diagnosis and therapy of this disease.

## Supporting information

S1 Table Genes associated with Menière’s disease and their Ensembl IDs, sources. (DOCX)

S2 Table The 4,514 RWR genes with probabilities higher than 1E-05. (DOCX)

S3 Table The 1,069 candidate genes with permutation FDRs less than 0.05. (DOCX)

S4 Table The 43 inferred genes with probabilities higher than 1E-05, permutation FDRs less than 0.05, *MIS*s greater than or equal to 900 and *MES*s larger than 0.8. (DOCX)

S5 Table The interactions used for drawing Fig 2. (DOCX)
