# Supplementary material for: A network-based method using a random walk with restart algorithm and screening tests to identify novel genes associated with Menière's disease
Source: PLoS One. 2017 Aug 7;12(8):e0182592. doi: 10.1371/journal.pone.0182592 (PMC5546581; doi:10.1371/journal.pone.0182592)
Supplement: S1 Table — (DOCX) [file pone.0182592.s001.docx]

**S1 Table.** Genes associated with Menière’s disease and their Ensembl IDs, sources.

| **Gene symbol** | **Ensembl ID** | **Reference** |
| --- | --- | --- |
| ADD1 | ENSP00000264758 | [1,2] |
| ADD2 | ENSP00000264436 | [2] |
| ADD3 | ENSP00000348381 | [2] |
| NPR1 | ENSP00000357669 | [3] |
| NPR2 | ENSP00000341083 | [3] |
| NPR3 | ENSP00000398028 | [3] |
| AQP1 | ENSP00000311165 | [4-6] |
| AQP2 | ENSP00000199280 | [1,6-9] |
| AQP3 | ENSP00000297991 | [6] |
| AQP4 | ENSP00000372654 | [5,7,10] |
| AQP5 | ENSP00000293599 | [6,7,10] |
| AQP6 | ENSP00000320247 | [5,10] |
| CACNA1A | ENSP00000353362 | [11,12] |
| CAV1 | ENSP00000339191 | [13] |
| CGA | ENSP00000358595 | [14] |
| CHGA | ENSP00000216492 | [14] |
| COCH | ENSP00000216361 | [1,8,11,15-21] |
| CTLA4 | ENSP00000303939 | [22] |
| DEFB1 | ENSP00000297439 | [23] |
| DIAPH1 | ENSP00000381565 | [24] |
| WFS1 | - | [24] |
| POU4F3 | ENSP00000230732 | [11] |
| GJB3 | ENSP00000362460 | [25] |
| GJB6 | ENSP00000241124 | [26] |
| DTNA | ENSP00000382064 | [11] |
| ESR1 | ENSP00000206249 | [10] |
| ESR2 | ENSP00000343925 | [10] |
| FAM136A | ENSP00000037869 | [27] |
| FCHO1 | ENSP00000252771 | [19] |
| FCHO2 | ENSP00000393776 | [19] |
| FLNA | ENSP00000358866 | [28] |
| GLAST1 | - | [16] |
| GPX1 | ENSP00000407375 | [29] |
| HCFC1 | ENSP00000309555 | [1,30] |
| HLA-A | ENSP00000416233 ENSP00000388526 ENSP00000398188 ENSP00000373114 ENSP00000388724 ENSP00000410645 ENSP00000366005 | [28,31-35] |
| HLA-B | ENSP00000399168 ENSP00000400842 | [31-36] |
| HLA-C | ENSP00000365402 ENSP00000372975 ENSP00000390282 ENSP00000407431 ENSP00000397867 ENSP00000383245 ENSP00000413992 | [28,31-35] |
| HLA-DPA2 | - | [34,35] |
| HLA-DRB1 | ENSP00000353099 | [16,27,32,33,35,37-39] |
| HLA-DQA1 | ENSP00000401760 ENSP00000414360 ENSP00000372738 ENSP00000339398 ENSP00000409127 ENSP00000387892 | [34,35] |
| HSPA1A | ENSP00000404524 ENSP00000408907 ENSP00000382915 ENSP00000406359 ENSP00000364802 | [40] |
| IFNG | ENSP00000229135 | [41] |
| IL1A | ENSP00000263339 | [42] |
| IL1B | ENSP00000263341 | [42] |
| KCNA1 | - | [11,12] |
| KCNE1 | ENSP00000337255 | [1,8,43,44] |
| KCNE3 | ENSP00000310557 | [1,8,43-45] |
| KCNQ4 | ENSP00000262916 | [25,46] |
| LTF | ENSP00000231751 | [23] |
| MICA | ENSP00000402134 | [47] |
| MIF | ENSP00000215754 | [41,48] |
| MRPS12 | ENSP00000308845 | [49] |
| MTHFR | ENSP00000365775 | [50] |
| MTNR1B | ENSP00000257068 | [13] |
| MTR | ENSP00000355536 | [13] |
| MTRR | ENSP00000264668 | [13] |
| NDUFS2 | ENSP00000356972 | [13] |
| NAGA | ENSP00000379680 | [51] |
| NFKB1 | ENSP00000226574 | [52] |
| SLC12A2 | - | [53] |
| NOS2 | ENSP00000327251 | [54] |
| NOS3 | ENSP00000297494 | [13] |
| NOTCH3 | ENSP00000263388 | [55] |
| PARP1 | ENSP00000355759 | [56] |
| PON1 | ENSP00000222381 | [29] |
| PON2 | ENSP00000222572 | [29] |
| PTPN22 | ENSP00000352833 | [8,22] |
| CCL5 | ENSP00000293272 | [57] |
| REL | ENSP00000295025 | [52] |
| SIK1 | ENSP00000270162 | [58] |
| SLC26A4 | - | [59] |
| SLC8A1 | ENSP00000332931 | [58] |
| SOD2 | ENSP00000337127 | [29] |
| TFB1M | ENSP00000356134 | [49] |
| TLR10 | ENSP00000308925 | [60] |
| TLR3 | ENSP00000296795 | [60] |
| TLR4 | ENSP00000363089 | [60,61] |
| TLR7 | ENSP00000370034 | [60] |
| TLR8 | ENSP00000218032 | [60] |
| TNF | ENSP00000398698 ENSP00000389265 ENSP00000389492 ENSP00000392858 ENSP00000389490 ENSP00000410668 ENSP00000372988 ENSP00000365290 | [46] |
| TNFAIP3 | ENSP00000237289 | [52] |
| TNIP1 | ENSP00000317891 | [52] |
| AVPR2 | - | [62] |
| VHL | ENSP00000256474 | [63] |

**References**

1. Hietikko E, Kotimaki J, Okuloff A, Sorri M, Mannikko M (2012) A replication study on proposed candidate genes in Meniere's disease, and a review of the current status of genetic studies. Int J Audiol 51: 841-845.

2. Teggi R, Lanzani C, Zagato L, Carpini SD, Manunta P, et al. (2008) Gly460Trp alpha-adducin mutation as a possible mechanism leading to endolymphatic hydrops in Meniere's syndrome. Otology & Neurotology 29: 824-828.

3. Dornhoffer JL, Zhou L, Danner C, Li SL (2002) Atrial natriuretic peptide receptor upregulation in the rat inner ear. Annals Of Otology Rhinology And Laryngology 111: 1040-1044.

4. Candreia C, Schmuziger N, Gurtler N (2010) Molecular Analysis of Aquaporin Genes 1 to 4 in Patients with Meniere's Disease. Cellular Physiology And Biochemistry 26: 787-792.

5. Ishiyama G, Lopez IA, Beltran-Parrazal L, Ishiyama A (2010) Immunohistochemical localization and mRNA expression of aquaporins in the macula utriculi of patients with Meniere's disease and acoustic neuroma. Cell And Tissue Research 340: 407-419.

6. Takumi Y, Nagelhus EA, Eidet J, Matsubara A, Usami S, et al. (1998) Select types of supporting cell in the inner ear express aquaporin-4 water channel protein. European Journal Of Neuroscience 10: 3584-3595.

7. Eckhard A, Gleiser C, Arnold H, Rask-Andersen H, Kumagami H, et al. (2012) Water channel proteins in the inner ear and their link to hearing impairment and deafness. Molecular Aspects Of Medicine 33: 612-637.

8. Gabrikova D, Frykholm C, Friberg U, Lahsaee S, Entesarian M, et al. (2010) Familiar Meniere's disease restricted to 1.48 Mb on chromosome 12p12.3 by allelic and haplotype association. J Hum Genet 55: 834-837.

9. Mhatre AN, Jero J, Chiappini I, Bolasco G, Barbara M, et al. (2002) Aquaporin-2 expression in the mammalian cochlea and investigation of its role in Meniere's disease. Hear Res 170: 59-69.

10. Nishio N, Teranishi M, Uchida Y, Sugiura S, Ando F, et al. (2013) Polymorphisms in genes encoding aquaporins 4 and 5 and estrogen receptor alpha in patients with Meniere's disease and sudden sensorineural hearing loss. Life Sci 92: 541-546.

11. Requena T, Espinosa-Sanchez JM, Lopez-Escamez JA (2014) Genetics of dizziness: cerebellar and vestibular disorders. Current Opinion In Neurology 27: 98-104.

12. Jen JC (2008) Recent advances in the genetics of recurrent vertigo and vestibulopathy. Current Opinion In Neurology 21: 3-7.

13. Teranishi M, Uchida Y, Nishio N, Kato K, Otake H, et al. (2013) Polymorphisms in genes involved in the free-radical process in patients with sudden sensorineural hearing loss and Meniere's disease. Free Radical Research 47: 498-506.

14. Teggi R, Colombo B, Trimarchi M, Bianco M, Manfredi A, et al. (2015) Altered Chromogranin A Circulating Levels in Meniere's Disease. Disease Markers: 1-6.

15. Kim BJ, Kim AR, Han KH, Rah YC, Hyun J, et al. (2016) Distinct vestibular phenotypes in DFNA9 families with COCH variants. European Archives Of Oto-Rhino-Laryngology 273: 2993-3002.

16. Ahmed S, Vorasubin N, Lopez IA, Hosokawa S, Ishiyama G, et al. (2013) The expression of glutamate aspartate transporter (GLAST) within the human cochlea and its distribution in various patient populations. Brain Res 1529: 134-142.

17. Ernst A (2011) [Perspectives in neurotology]. Laryngorhinootologie 90 Suppl 1: S35-43.

18. Ikezono T, Shindo S, Ishizaki M, Li L, Tomiyama S, et al. (2005) Expression of cochlin in the vestibular organ of rats. ORL J Otorhinolaryngol Relat Spec 67: 252-258.

19. Usami S, Takahashi K, Yuge I, Ohtsuka A, Namba A, et al. (2003) Mutations in the COCH gene are a frequent cause of autosomal dominant progressive cochleo-vestibular dysfunction, but not of Meniere's disease. Eur J Hum Genet 11: 744-748.

20. Lemaire FX, Feenstra L, Huygen PL, Fransen E, Devriendt K, et al. (2003) Progressive late-onset sensorineural hearing loss and vestibular impairment with vertigo (DFNA9/COCH): longitudinal analyses in a belgian family. Otol Neurotol 24: 743-748.

21. Verhagen WIM, Bom SJH, Fransen E, Van Camp G, Huygen PLM, et al. (2001) Hereditary cochleovestibular dysfunction due to a COCH gene mutation (DFNA9): a follow-up study of a family. Clinical Otolaryngology 26: 477-483.

22. Lopez-Escamez JA, Saenz-Lopez P, Acosta L, Moreno A, Gazquez I, et al. (2010) Association of a Functional Polymorphism of PTPN22 Encoding a Lymphoid Protein Phosphatase in Bilateral Meniere's Disease. Laryngoscope 120: 103-107.

23. Moller MN, Kirkeby S, Vikesa J, Nielsen FC, Caye-Thomasen P (2015) Gene expression demonstrates an immunological capacity of the human endolymphatic sac. Laryngoscope 125: E269-E275.

24. Frykholm C, Larsen HC, Dahl N, Klar J, Rask-Andersen H, et al. (2006) Familial Meniere's disease in five generations. Otology & Neurotology 27: 681-686.

25. Kesser BW, Hashisaki GT, Holt JR (2008) Gene Transfer in Human Vestibular Epithelia and the Prospects for Inner Ear Gene Therapy. Laryngoscope 118: 821-831.

26. Eppsteiner RW, Smith RJH (2011) Genetic disorders of the vestibular system. Current Opinion In Otolaryngology & Head And Neck Surgery 19: 397-402.

27. Frejo L, Giegling I, Teggi R, Lopez-Escamez JA, Rujescu D (2016) Genetics of vestibular disorders: pathophysiological insights. J Neurol 263 Suppl 1: S45-53.

28. Yeo SW, Park SN, Jeon EJ, Lee HY, Pyo CW, et al. (2002) Influence of human leukocyte antigen in the pathogenesis of Meniere's disease in the South Korean population. Acta Otolaryngol 122: 851-856.

29. Teranishi M, Uchida Y, Nishio N, Kato K, Otake H, et al. (2012) Polymorphisms in genes involved in oxidative stress response in patients with sudden sensorineural hearing loss and Meniere's disease in a Japanese population. DNA Cell Biol 31: 1555-1562.

30. Vrabec JT, Liu L, Li B, Leal SM (2008) Sequence variants in host cell factor C1 are associated with Meniere's disease. Otol Neurotol 29: 561-566.

31. Khorsandi MT, Amoli MM, Borghei H, Emami H, Amiri P, et al. (2011) Associations between HLA-C Alleles and Definite Meniere's Disease. Iranian Journal of Allergy Asthma and Immunology 10: 119-122.

32. Melchiorri L, Martini A, Rizzo R, Berto A, Adinolfi E, et al. (2002) Human leukocyte antigen-A, -B, -C and -DR alleles and soluble human leukocyte antigen class I serum level in Meniere's disease. Acta Otolaryngol Suppl: 26-29.

33. Lopez-Escamez JA, Lopez-Nevot A, Cortes R, Ramal L, Lopez-Nevot MA (2002) Expression of A, B, C and DR antigens in definite Meniere's disease in a Spanish population. Eur Arch Otorhinolaryngol 259: 347-350.

34. Bernstein JM, Shanahan TC, Schaffer FM (1996) Further observations on the role of the MHC genes and certain hearing disorders. Acta Otolaryngol 116: 666-671.

35. Koyama S, Mitsuishi Y, Bibee K, Watanabe I, Terasaki PI (1993) HLA associations with Meniere's disease. Acta Otolaryngol 113: 575-578.

36. Arweiler DJ, Jahnke K, Grosse-Wilde H (1995) [Meniere disease as an autosome dominant hereditary disease]. Laryngorhinootologie 74: 512-515.

37. Lopez-Escamez JA, Vilchez JR, Soto-Varela A, Santos-Perez S, Perez-Garrigues H, et al. (2007) HLA-DRB1*1101 allele may be associated with bilateral Meniere's disease in southern European population. Otol Neurotol 28: 891-895.

38. Koo JW, Oh SH, Chang SO, Park MH, Lim MJ, et al. (2003) Association of HLA-DR and type II collagen autoimmunity with Meniere's disease. Tissue Antigens 61: 99-103.

39. Meng X, Lian N, Yang Z, Liu C, Tang L (2001) [An association study of histocompatibility leukocyte antigen-class II with Meniere's disease]. Zhonghua Er Bi Yan Hou Ke Za Zhi 36: 25-27.

40. Kawaguchi S, Hagiwara A, Suzuki M (2008) Polymorphic analysis of the heat-shock protein 70 gene (HSPA1A) in Meniere's disease. Acta Otolaryngol 128: 1173-1177.

41. Gazquez I, Moreno A, Requena T, Ohmen J, Santos-Perez S, et al. (2013) Functional variants of MIF, INFG and TFNA genes are not associated with disease susceptibility or hearing loss progression in patients with Meniere's disease. Eur Arch Otorhinolaryngol 270: 1521-1529.

42. Furuta T, Teranishi M, Uchida Y, Nishio N, Kato K, et al. (2011) Association of interleukin-1 gene polymorphisms with sudden sensorineural hearing loss and Meniere's disease. Int J Immunogenet 38: 249-254.

43. Li YJ, Jin ZG, Xu XR (2016) Variants in the KCNE1 or KCNE3 gene and risk of Meniere's disease: A meta-analysis. J Vestib Res 25: 211-218.

44. Campbell CA, Della Santina CC, Meyer NC, Smith NB, Myrie OA, et al. (2010) Polymorphisms in KCNE1 or KCNE3 are not associated with Meniere disease in the Caucasian population. Am J Med Genet A 152a: 67-74.

45. Wang W, Kim HJ, Lee JH, Wong V, Sihn CR, et al. (2014) Functional significance of K+ channel beta-subunit KCNE3 in auditory neurons. J Biol Chem 289: 16802-16813.

46. Greco A, Gallo A, Fusconi M, Marinelli C, Macri GF, et al. (2012) Meniere's disease might be an autoimmune condition? Autoimmunity Reviews 11: 731-738.

47. Gazquez I, Moreno A, Aran I, Soto-Varela A, Santos S, et al. (2012) MICA-STR A.4 is associated with slower hearing loss progression in patients with Meniere's disease. Otol Neurotol 33: 223-229.

48. Yazdani N, Ashtiani MTK, Zarandy MM, Mohammadi SJ, Ghazavi H, et al. (2013) Association between MIF gene variation and Meniere's disease. International Journal of Immunogenetics 40: 488-491.

49. Pacheu-Grau D, Perez-Delgado L, Gomez-Diaz C, Fraile-Rodrigo J, Montoya J, et al. (2012) Mitochondrial ribosome and Meniere's disease: a pilot study. Eur Arch Otorhinolaryngol 269: 2003-2008.

50. Huang Y, Teranishi M, Uchida Y, Nishio N, Kato K, et al. (2013) Association between polymorphisms in genes encoding methylenetetrahydrofolate reductase and the risk of Meniere's disease. J Neurogenet 27: 5-10.

51. Kodama K, Kobayashi H, Abe R, Ohkawara A, Yoshii N, et al. (2001) A new case of alpha-N-acetylgalactosaminidase deficiency with angiokeratoma corporis diffusum, with Meniere's syndrome and without mental retardation. Br J Dermatol 144: 363-368.

52. Cabrera S, Sanchez E, Requena T, Martinez-Bueno M, Benitez J, et al. (2014) Intronic Variants in the NFKB1 Gene May Influence Hearing Forecast in Patients with Unilateral Sensorineural Hearing Loss in Meniere's Disease. Plos One 9: e112171.

53. Ito T, Li X, Kurima K, Choi BY, Wangemann P, et al. (2014) Slc26a4-insufficiency causes fluctuating hearing loss and stria vascularis dysfunction. Neurobiol Dis 66: 53-65.

54. Gazquez I, Lopez-Escamez JA, Moreno A, Campbell CA, Meyer NC, et al. (2011) Functional variants in NOS1 and NOS2A are not associated with progressive hearing loss in Meniere's disease in a European Caucasian population. DNA Cell Biol 30: 699-708.

55. Oki K, Nagata E, Ishiko A, Shimizu A, Tanaka K, et al. (2007) Novel mutation of the Notch3 gene in a Japanese patient with CADASIL. Eur J Neurol 14: 464-466.

56. Lopez-Escamez JA, Moreno A, Bernal M, Perez-Garrigues H, Santos-Perez S, et al. (2009) Poly(ADP-ribose) polymerase-1 (PARP-1) longer alleles spanning the promoter region may confer protection to bilateral Meniere's disease. Acta Otolaryngol 129: 1222-1225.

57. Yazdani N, Mojbafan M, Taleba M, Amiri P, Nejadian F, et al. (2015) Sex-specific association of RANTES gene -403 variant in Meniere's disease. Eur Arch Otorhinolaryngol 272: 2221-2225.

58. Teggi R, Zagato L, Delli Carpini S, Citterio L, Cassandro C, et al. (2017) Genetics of ion homeostasis in Meniere's Disease. Eur Arch Otorhinolaryngol 274: 757-763.

59. Stinckens C, Huygen PL, Joosten FB, Van Camp G, Otten B, et al. (2001) Fluctuant, progressive hearing loss associated with Meniere like vertigo in three patients with the Pendred syndrome. Int J Pediatr Otorhinolaryngol 61: 207-215.

60. Requena T, Gazquez I, Moreno A, Batuecas A, Aran I, et al. (2013) Allelic variants in TLR10 gene may influence bilateral affectation and clinical course of Meniere's disease. Immunogenetics 65: 345-355.

61. Traks T, Keermann M, Karelson M, Ratsep R, Reimann E, et al. (2015) Polymorphisms in Toll-like receptor genes are associated with vitiligo. Front Genet 6: 278.

62. Kitahara T, Maekawa C, Kizawa K, Kamakura T, Horii A, et al. (2011) Endolymphatic sac tumor with overexpression of V2 receptor mRNA and inner ear hydrops. Acta Otolaryngol 131: 951-957.

63. Choo D, Shotland L, Mastroianni M, Glenn G, van Waes C, et al. (2004) Endolymphatic sac tumors in von Hippel-Lindau disease. J Neurosurg 100: 480-487.
