# Supplementary material for: A network-based method using a random walk with restart algorithm and screening tests to identify novel genes associated with Menière's disease
Source: PLoS One. 2017 Aug 7;12(8):e0182592. doi: 10.1371/journal.pone.0182592 (PMC5546581; doi:10.1371/journal.pone.0182592)
Supplement: S2 Table — (DOCX) [file pone.0182592.s002.docx]

**S2 Table.** The 4,514 RWR genes with probabilities higher than 1E-05.

| **Ensembl ID** | **Gene symbol** | **Probability** | **P-value** |
| --- | --- | --- | --- |
| ENSP00000295897 | ALB | 4.28E-04 | 0.032 |
| ENSP00000348986 | INS-IGF2 | 4.00E-04 | 0.001 |
| ENSP00000258743 | IL6 | 3.62E-04 | 0.004 |
| ENSP00000328973 | TSPO | 3.61E-04 | <0.001 |
| ENSP00000344818 | UBC | 3.56E-04 | 0.948 |
| ENSP00000349467 | CALM1 | 3.28E-04 | 0.008 |
| ENSP00000011653 | CD4 | 3.11E-04 | 0.003 |
| ENSP00000272298 | CALM2 | 2.84E-04 | 0.009 |
| ENSP00000291295 | CALM3 | 2.80E-04 | 0.006 |
| ENSP00000264832 | ICAM1 | 2.49E-04 | <0.001 |
| ENSP00000270202 | AKT1 | 2.43E-04 | 0.029 |
| ENSP00000361359 | CD40 | 2.35E-04 | <0.001 |
| ENSP00000352312 | DNAH8 | 2.35E-04 | <0.001 |
| ENSP00000375881 | ALPP | 2.27E-04 | <0.001 |
| ENSP00000295453 | ALPPL2 | 2.22E-04 | <0.001 |
| ENSP00000361125 | VEGFA | 2.12E-04 | 0.075 |
| ENSP00000363965 | ALPL | 2.12E-04 | 0.005 |
| ENSP00000412237 | IL10 | 2.11E-04 | <0.001 |
| ENSP00000226730 | IL2 | 2.08E-04 | 0.012 |
| ENSP00000229239 | GAPDH | 2.07E-04 | 0.002 |
| ENSP00000365663 | NPPA | 2.05E-04 | <0.001 |
| ENSP00000296871 | CSF2 | 2.05E-04 | 0.004 |
| ENSP00000292303 | CCR5 | 2.01E-04 | <0.001 |
| ENSP00000332049 | CD86 | 2.00E-04 | <0.001 |
| ENSP00000269305 | TP53 | 1.99E-04 | 0.128 |
| ENSP00000290866 | ACE | 1.99E-04 | <0.001 |
| ENSP00000351697 | REV3L | 1.95E-04 | <0.001 |
| ENSP00000356438 | PTGS2 | 1.94E-04 | 0.007 |
| ENSP00000306245 | FOS | 1.92E-04 | 0.048 |
| ENSP00000350941 | SRC | 1.89E-04 | 0.079 |
| ENSP00000221930 | TGFB1 | 1.88E-04 | 0.009 |
| ENSP00000272190 | REN | 1.87E-04 | <0.001 |
| ENSP00000263321 | TYR | 1.84E-04 | 0.002 |
| ENSP00000368349 | POLA1 | 1.84E-04 | 0.009 |
| ENSP00000306512 | IL8 | 1.82E-04 | 0.005 |
| ENSP00000322570 | POLE | 1.82E-04 | 0.004 |
| ENSP00000260010 | TLR2 | 1.80E-04 | <0.001 |
| ENSP00000003084 | CFTR | 1.80E-04 | 0.01 |
| ENSP00000365435 | TNFRSF1B | 1.79E-04 | <0.001 |
| ENSP00000364114 | HLA-DRB5 | 1.77E-04 | 0.001 |
| ENSP00000294728 | VCAM1 | 1.75E-04 | <0.001 |
| ENSP00000221972 | CD79A | 1.74E-04 | 0.017 |
| ENSP00000340858 | B2M | 1.73E-04 | 0.076 |
| ENSP00000309845 | HRAS | 1.72E-04 | 0.17 |
| ENSP00000282561 | GJA1 | 1.72E-04 | 0.001 |
| ENSP00000365380 | FOXP3 | 1.71E-04 | 0.008 |
| ENSP00000388107 | UBA52 | 1.66E-04 | 0.188 |
| ENSP00000360266 | JUN | 1.64E-04 | 0.018 |
| ENSP00000263686 | SELP | 1.63E-04 | <0.001 |
| ENSP00000278385 | CD44 | 1.56E-04 | 0.002 |
| ENSP00000300589 | NOD2 | 1.53E-04 | 0.008 |
| ENSP00000312652 | LEP | 1.52E-04 | 0.067 |
| ENSP00000302961 | HSPA4 | 1.51E-04 | 0.005 |
| ENSP00000320866 | CALR | 1.49E-04 | 0.04 |
| ENSP00000264246 | CD80 | 1.49E-04 | 0.011 |
| ENSP00000412031 | HLA-DRB4 | 1.48E-04 | <0.001 |
| ENSP00000355627 | AGT | 1.48E-04 | 0.011 |
| ENSP00000382034 | HLA-DQB1 | 1.48E-04 | 0.006 |
| ENSP00000386884 | CXCR4 | 1.47E-04 | 0.01 |
| ENSP00000225831 | CCL2 | 1.46E-04 | 0.006 |
| ENSP00000342215 | KIR2DL3 | 1.46E-04 | 0.003 |
| ENSP00000331736 | SELE | 1.46E-04 | <0.001 |
| ENSP00000375608 | KIR3DL1 | 1.45E-04 | <0.001 |
| ENSP00000336769 | KIR2DL1 | 1.42E-04 | 0.001 |
| ENSP00000227507 | CCND1 | 1.42E-04 | 0.037 |
| ENSP00000318472 | NCAM1 | 1.41E-04 | 0.003 |
| ENSP00000369647 | AVP | 1.39E-04 | 0.004 |
| ENSP00000372734 | HLA-DQB1 | 1.38E-04 | <0.001 |
| ENSP00000349960 | ACTB | 1.38E-04 | 0.027 |
| ENSP00000369293 | IL2RA | 1.37E-04 | 0.003 |
| ENSP00000379625 | MYD88 | 1.37E-04 | 0.001 |
| ENSP00000311032 | CASP3 | 1.37E-04 | 0.006 |
| ENSP00000339393 | CCR6 | 1.37E-04 | <0.001 |
| ENSP00000346839 | FN1 | 1.36E-04 | 0.046 |
| ENSP00000302150 | PRL | 1.35E-04 | 0.005 |
| ENSP00000384273 | RELA | 1.34E-04 | 0.049 |
| ENSP00000247461 | CANX | 1.34E-04 | 0.01 |
| ENSP00000316328 | CIITA | 1.34E-04 | 0.003 |
| ENSP00000276927 | IFNA1 | 1.34E-04 | 0.004 |
| ENSP00000264708 | POMC | 1.33E-04 | 0.003 |
| ENSP00000215832 | MAPK1 | 1.32E-04 | 0.074 |
| ENSP00000329411 | IRF7 | 1.32E-04 | <0.001 |
| ENSP00000383645 | KIR3DS1 | 1.32E-04 | <0.001 |
| ENSP00000410046 | HLA-DRB4 | 1.32E-04 | <0.001 |
| ENSP00000273550 | FTH1 | 1.31E-04 | 0.003 |
| ENSP00000265715 | SLC26A4 | 1.31E-04 | <0.001 |
| ENSP00000155840 | KCNQ1 | 1.30E-04 | 0.008 |
| ENSP00000318770 | AQP11 | 1.30E-04 | <0.001 |
| ENSP00000361850 | PLAU | 1.29E-04 | 0.002 |
| ENSP00000228534 | IL23A | 1.29E-04 | 0.001 |
| ENSP00000270474 | PDE4A | 1.29E-04 | <0.001 |
| ENSP00000372607 | HLA-DRB3 | 1.29E-04 | 0.003 |
| ENSP00000259271 | GAD2 | 1.29E-04 | <0.001 |
| ENSP00000009530 | CD74 | 1.29E-04 | 0.024 |
| ENSP00000337825 | LCK | 1.28E-04 | 0.018 |
| ENSP00000361405 | MMP9 | 1.27E-04 | 0.008 |
| ENSP00000264657 | STAT3 | 1.26E-04 | 0.017 |
| ENSP00000416022 | HLA-DRB3 | 1.26E-04 | 0.001 |
| ENSP00000252486 | APOE | 1.25E-04 | 0.004 |
| ENSP00000246657 | CCR7 | 1.25E-04 | <0.001 |
| ENSP00000310036 | CD34 | 1.24E-04 | 0.008 |
| ENSP00000329967 | TBK1 | 1.24E-04 | 0.008 |
| ENSP00000331514 | ACTG1 | 1.23E-04 | 0.018 |
| ENSP00000302665 | IGF1 | 1.22E-04 | 0.011 |
| ENSP00000382025 | HLA-DQB1 | 1.22E-04 | 0.02 |
| ENSP00000320084 | CD276 | 1.21E-04 | 0.006 |
| ENSP00000363822 | AR | 1.21E-04 | 0.046 |
| ENSP00000396688 | C4A | 1.21E-04 | 0.006 |
| ENSP00000280357 | IL18 | 1.21E-04 | 0.002 |
| ENSP00000255030 | CRP | 1.21E-04 | 0.031 |
| ENSP00000265171 | EGF | 1.20E-04 | 0.123 |
| ENSP00000372815 | C4A | 1.20E-04 | 0.006 |
| ENSP00000410857 | HLA-DRB4 | 1.20E-04 | 0.001 |
| ENSP00000313419 | CD19 | 1.19E-04 | 0.019 |
| ENSP00000389288 | HLA-DPB1 | 1.19E-04 | 0.005 |
| ENSP00000226359 | AFP | 1.19E-04 | 0.013 |
| ENSP00000351671 | CCL20 | 1.19E-04 | 0.002 |
| ENSP00000296545 | IL15 | 1.19E-04 | <0.001 |
| ENSP00000224237 | VIM | 1.18E-04 | 0.017 |
| ENSP00000342952 | ADCY2 | 1.17E-04 | 0.01 |
| ENSP00000229794 | MAPK14 | 1.17E-04 | 0.04 |
| ENSP00000236850 | APOA1 | 1.16E-04 | 0.011 |
| ENSP00000364444 | C4A | 1.16E-04 | <0.001 |
| ENSP00000382018 | HLA-DQB1 | 1.16E-04 | 0.012 |
| ENSP00000253513 | IDO1 | 1.16E-04 | 0.001 |
| ENSP00000264998 | TF | 1.15E-04 | 0.029 |
| ENSP00000357981 | CTSS | 1.15E-04 | 0.023 |
| ENSP00000354394 | STAT1 | 1.15E-04 | 0.035 |
| ENSP00000353224 | TFRC | 1.14E-04 | 0.008 |
| ENSP00000349770 | IRF5 | 1.14E-04 | 0.001 |
| ENSP00000353483 | MAPK8 | 1.14E-04 | 0.025 |
| ENSP00000252444 | LDLR | 1.14E-04 | 0.008 |
| ENSP00000416330 | TGFBI | 1.14E-04 | 0.023 |
| ENSP00000249075 | LIF | 1.14E-04 | 0.006 |
| ENSP00000388662 | C4A | 1.14E-04 | <0.001 |
| ENSP00000262367 | CREBBP | 1.14E-04 | 0.084 |
| ENSP00000225474 | CSF3 | 1.13E-04 | 0.001 |
| ENSP00000343040 | HMGB1 | 1.13E-04 | <0.001 |
| ENSP00000356694 | FASLG | 1.13E-04 | 0.003 |
| ENSP00000396486 | HSPA1L | 1.13E-04 | 0.002 |
| ENSP00000412786 | C4B_2 | 1.12E-04 | <0.001 |
| ENSP00000269571 | ERBB2 | 1.12E-04 | 0.074 |
| ENSP00000287820 | PPARG | 1.12E-04 | 0.058 |
| ENSP00000231449 | IL4 | 1.12E-04 | 0.009 |
| ENSP00000340019 | HSPD1 | 1.12E-04 | <0.001 |
| ENSP00000292301 | CCR2 | 1.11E-04 | <0.001 |
| ENSP00000262629 | TYROBP | 1.11E-04 | 0.087 |
| ENSP00000277541 | NOTCH1 | 1.11E-04 | 0.096 |
| ENSP00000275493 | EGFR | 1.11E-04 | 0.325 |
| ENSP00000162749 | TNFRSF1A | 1.11E-04 | 0.011 |
| ENSP00000387699 | CREB1 | 1.11E-04 | 0.044 |
| ENSP00000307786 | CYCS | 1.10E-04 | 0.026 |
| ENSP00000262186 | KCNH2 | 1.10E-04 | 0.014 |
| ENSP00000252723 | EPO | 1.08E-04 | 0.004 |
| ENSP00000352064 | KLRC1 | 1.07E-04 | <0.001 |
| ENSP00000356346 | PTPRC | 1.07E-04 | 0.02 |
| ENSP00000245414 | IRF1 | 1.06E-04 | 0.006 |
| ENSP00000340011 | KIR2DS4 | 1.06E-04 | <0.001 |
| ENSP00000283635 | CD8A | 1.06E-04 | 0.013 |
| ENSP00000261267 | LYZ | 1.06E-04 | 0.034 |
| ENSP00000304697 | UBB | 1.06E-04 | 0.047 |
| ENSP00000324806 | GSK3B | 1.06E-04 | 0.026 |
| ENSP00000414196 | HLA-DPB1 | 1.06E-04 | 0.014 |
| ENSP00000241052 | CAT | 1.05E-04 | 0.009 |
| ENSP00000305651 | CXCL10 | 1.05E-04 | 0.002 |
| ENSP00000388001 | OAS1 | 1.05E-04 | 0.004 |
| ENSP00000265023 | KNG1 | 1.04E-04 | 0.01 |
| ENSP00000298125 | WDFY2 | 1.04E-04 | <0.001 |
| ENSP00000338130 | KLRD1 | 1.04E-04 | <0.001 |
| ENSP00000396538 | NFAT5 | 1.04E-04 | 0.036 |
| ENSP00000287497 | ITGAM | 1.03E-04 | 0.001 |
| ENSP00000263125 | PRKCQ | 1.03E-04 | 0.014 |
| ENSP00000225275 | MPO | 1.03E-04 | 0.006 |
| ENSP00000363548 | CXCL12 | 1.02E-04 | 0.016 |
| ENSP00000367207 | MYC | 1.02E-04 | 0.214 |
| ENSP00000359663 | CD40LG | 1.02E-04 | 0.001 |
| ENSP00000338018 | HIF1A | 1.01E-04 | 0.008 |
| ENSP00000369889 | COL2A1 | 1.01E-04 | 0.016 |
| ENSP00000358501 | CD58 | 1.00E-04 | 0.001 |
| ENSP00000352121 | PIK3CG | 9.93E-05 | 0.007 |
| ENSP00000332116 | PDE4B | 9.90E-05 | <0.001 |
| ENSP00000285379 | CA2 | 9.84E-05 | 0.015 |
| ENSP00000368450 | CD83 | 9.81E-05 | <0.001 |
| ENSP00000259206 | IL1RN | 9.78E-05 | 0.009 |
| ENSP00000348461 | RAC1 | 9.78E-05 | 0.196 |
| ENSP00000270142 | SOD1 | 9.76E-05 | 0.023 |
| ENSP00000281537 | TJP1 | 9.75E-05 | 0.018 |
| ENSP00000371067 | JAK2 | 9.74E-05 | 0.03 |
| ENSP00000363976 | HLA-DMA | 9.74E-05 | 0.021 |
| ENSP00000306697 | MRAP | 9.72E-05 | 0.004 |
| ENSP00000263253 | EP300 | 9.68E-05 | 0.114 |
| ENSP00000347988 | NDUFA5 | 9.67E-05 | 0.013 |
| ENSP00000189444 | NFKB2 | 9.63E-05 | 0.005 |
| ENSP00000375892 | AKT2 | 9.62E-05 | 0.032 |
| ENSP00000399309 | NEU1 | 9.59E-05 | 0.011 |
| ENSP00000228434 | CD69 | 9.55E-05 | 0.003 |
| ENSP00000325525 | KIR3DL2 | 9.52E-05 | <0.001 |
| ENSP00000358525 | NGF | 9.47E-05 | 0.111 |
| ENSP00000309757 | LPL | 9.39E-05 | 0.025 |
| ENSP00000369554 | IFNA2 | 9.36E-05 | 0.005 |
| ENSP00000320758 | NOS1 | 9.35E-05 | 0.009 |
| ENSP00000407674 | HLA-DPB1 | 9.34E-05 | 0.009 |
| ENSP00000373648 | KCNQ3 | 9.31E-05 | 0.007 |
| ENSP00000233242 | APOB | 9.31E-05 | 0.011 |
| ENSP00000229281 | C12orf57 | 9.31E-05 | 0.001 |
| ENSP00000226279 | CD38 | 9.30E-05 | 0.004 |
| ENSP00000390707 | HLA-E | 9.29E-05 | 0.006 |
| ENSP00000253408 | GFAP | 9.28E-05 | 0.021 |
| ENSP00000370381 | SLC12A1 | 9.24E-05 | 0.007 |
| ENSP00000364782 | NEU1 | 9.20E-05 | 0.011 |
| ENSP00000216797 | NFKBIA | 9.20E-05 | 0.023 |
| ENSP00000353472 | HLA-G | 9.20E-05 | <0.001 |
| ENSP00000263851 | IL7 | 9.14E-05 | 0.006 |
| ENSP00000283977 | PGM3 | 9.13E-05 | 0.011 |
| ENSP00000359998 | GSTA4 | 9.13E-05 | 0.003 |
| ENSP00000291582 | AIRE | 9.13E-05 | <0.001 |
| ENSP00000331602 | PRKCD | 9.11E-05 | 0.016 |
| ENSP00000356213 | VIP | 9.01E-05 | 0.005 |
| ENSP00000324648 | CYP2B6 | 9.00E-05 | 0.016 |
| ENSP00000315997 | LILRB1 | 8.95E-05 | 0.001 |
| ENSP00000250092 | CD68 | 8.94E-05 | 0.02 |
| ENSP00000335153 | HSP90AA1 | 8.94E-05 | 0.057 |
| ENSP00000296861 | TNFRSF21 | 8.93E-05 | <0.001 |
| ENSP00000263967 | PIK3CA | 8.92E-05 | 0.072 |
| ENSP00000339634 | KIR2DL4 | 8.87E-05 | <0.001 |
| ENSP00000284384 | PRKCA | 8.86E-05 | 0.058 |
| ENSP00000417404 | HFE | 8.86E-05 | 0.001 |
| ENSP00000357068 | KCNJ10 | 8.78E-05 | 0.018 |
| ENSP00000344192 | IL17A | 8.76E-05 | <0.001 |
| ENSP00000365048 | TNFSF13B | 8.76E-05 | 0.01 |
| ENSP00000409151 | HSPA1L | 8.73E-05 | 0.007 |
| ENSP00000387662 | GCG | 8.69E-05 | 0.091 |
| ENSP00000416561 | CFB | 8.68E-05 | <0.001 |
| ENSP00000347379 | OCLN | 8.68E-05 | 0.001 |
| ENSP00000353874 | TLR9 | 8.65E-05 | <0.001 |
| ENSP00000304283 | RAC3 | 8.64E-05 | 0.076 |
| ENSP00000295440 | NPPC | 8.63E-05 | <0.001 |
| ENSP00000263826 | AKT3 | 8.62E-05 | 0.017 |
| ENSP00000348273 | MBP | 8.61E-05 | 0.021 |
| ENSP00000220764 | DECR1 | 8.61E-05 | 0.029 |
| ENSP00000240618 | KLRK1 | 8.61E-05 | <0.001 |
| ENSP00000365651 | NPPB | 8.61E-05 | 0.006 |
| ENSP00000216968 | PROCR | 8.59E-05 | <0.001 |
| ENSP00000219070 | MMP2 | 8.55E-05 | 0.01 |
| ENSP00000360798 | EPS15 | 8.52E-05 | 0.037 |
| ENSP00000269280 | NLRP1 | 8.49E-05 | 0.003 |
| ENSP00000223095 | SERPINE1 | 8.49E-05 | 0.012 |
| ENSP00000368226 | GK | 8.48E-05 | <0.001 |
| ENSP00000216117 | HMOX1 | 8.43E-05 | 0.001 |
| ENSP00000357656 | FYN | 8.42E-05 | 0.231 |
| ENSP00000369581 | IFNB1 | 8.41E-05 | 0.01 |
| ENSP00000411940 | IFNA17 | 8.37E-05 | <0.001 |
| ENSP00000392398 | GPX5 | 8.36E-05 | 0.002 |
| ENSP00000372793 | LTA | 8.33E-05 | 0.008 |
| ENSP00000372295 | GJB2 | 8.32E-05 | 0.011 |
| ENSP00000308165 | CD36 | 8.32E-05 | 0.016 |
| ENSP00000261405 | VWF | 8.31E-05 | 0.04 |
| ENSP00000261769 | CDH1 | 8.31E-05 | 0.244 |
| ENSP00000263464 | BIRC3 | 8.28E-05 | 0.005 |
| ENSP00000416509 | LTA | 8.28E-05 | 0.001 |
| ENSP00000303208 | PCSK9 | 8.27E-05 | 0.005 |
| ENSP00000300305 | RUNX1 | 8.27E-05 | 0.055 |
| ENSP00000397331 | HLA-G | 8.26E-05 | <0.001 |
| ENSP00000200691 | MT3 | 8.22E-05 | <0.001 |
| ENSP00000339151 | IKBKB | 8.21E-05 | 0.01 |
| ENSP00000402694 | HLA-E | 8.21E-05 | 0.008 |
| ENSP00000303830 | INSR | 8.20E-05 | 0.051 |
| ENSP00000216341 | GZMB | 8.17E-05 | 0.022 |
| ENSP00000352035 | KCNQ2 | 8.17E-05 | 0.009 |
| ENSP00000407195 | TAPBP | 8.16E-05 | 0.03 |
| ENSP00000231509 | NR3C1 | 8.16E-05 | 0.034 |
| ENSP00000359424 | CHUK | 8.12E-05 | 0.015 |
| ENSP00000414303 | BDNF | 8.12E-05 | 0.075 |
| ENSP00000356087 | IKBKE | 8.12E-05 | 0.005 |
| ENSP00000387624 | HLA-G | 8.11E-05 | <0.001 |
| ENSP00000233946 | IL1R1 | 8.11E-05 | 0.015 |
| ENSP00000303242 | ITGB2 | 8.10E-05 | 0.037 |
| ENSP00000309124 | OLR1 | 8.06E-05 | <0.001 |
| ENSP00000287641 | SST | 8.06E-05 | 0.046 |
| ENSP00000304895 | IRS1 | 8.03E-05 | 0.035 |
| ENSP00000357283 | LMNA | 8.02E-05 | 0.041 |
| ENSP00000333298 | LAMP1 | 8.00E-05 | 0.031 |
| ENSP00000337144 | AQP12A | 7.95E-05 | 0.001 |
| ENSP00000310127 | IRF3 | 7.93E-05 | 0.018 |
| ENSP00000368174 | MCM8 | 7.93E-05 | 0.043 |
| ENSP00000369574 | IFNA21 | 7.92E-05 | <0.001 |
| ENSP00000271651 | CTSK | 7.92E-05 | 0.016 |
| ENSP00000234590 | ENO1 | 7.91E-05 | 0.013 |
| ENSP00000160262 | ICAM3 | 7.89E-05 | 0.005 |
| ENSP00000351273 | CASP8 | 7.89E-05 | 0.059 |
| ENSP00000239938 | EGR1 | 7.87E-05 | 0.02 |
| ENSP00000393087 | HSPA1B | 7.87E-05 | 0.023 |
| ENSP00000263461 | WDR11 | 7.83E-05 | <0.001 |
| ENSP00000320171 | PKM | 7.80E-05 | 0.029 |
| ENSP00000339933 | PKLR | 7.79E-05 | 0.013 |
| ENSP00000353452 | MYLK | 7.79E-05 | <0.001 |
| ENSP00000291890 | NCR1 | 7.79E-05 | <0.001 |
| ENSP00000372582 | HLA-DPB1 | 7.78E-05 | 0.003 |
| ENSP00000316136 | KCNJ1 | 7.77E-05 | 0.006 |
| ENSP00000264896 | SCARB2 | 7.76E-05 | 0.024 |
| ENSP00000395701 | TAPBP | 7.75E-05 | 0.009 |
| ENSP00000289153 | PIK3CB | 7.72E-05 | 0.027 |
| ENSP00000354932 | TLR1 | 7.71E-05 | 0.007 |
| ENSP00000340944 | PTPN11 | 7.71E-05 | 0.119 |
| ENSP00000261233 | IRAK3 | 7.70E-05 | <0.001 |
| ENSP00000362994 | TRAF1 | 7.70E-05 | 0.011 |
| ENSP00000200652 | SLC22A4 | 7.70E-05 | 0.005 |
| ENSP00000261693 | SCARB1 | 7.68E-05 | 0.024 |
| ENSP00000355330 | TGM2 | 7.65E-05 | 0.006 |
| ENSP00000312286 | PLA2G1B | 7.65E-05 | 0.033 |
| ENSP00000321345 | IL23R | 7.64E-05 | 0.022 |
| ENSP00000263621 | ELANE | 7.63E-05 | 0.021 |
| ENSP00000288266 | APPL1 | 7.62E-05 | 0.235 |
| ENSP00000382422 | HLA-DPB1 | 7.62E-05 | 0.003 |
| ENSP00000334145 | F3 | 7.61E-05 | 0.016 |
| ENSP00000391592 | PTPN6 | 7.61E-05 | 0.07 |
| ENSP00000262965 | TCF3 | 7.61E-05 | 0.055 |
| ENSP00000299855 | MMP3 | 7.59E-05 | <0.001 |
| ENSP00000346032 | ANXA2 | 7.59E-05 | 0.054 |
| ENSP00000013034 | NME1 | 7.58E-05 | 0.008 |
| ENSP00000366525 | FTL | 7.56E-05 | 0.011 |
| ENSP00000373091 | HLA-E | 7.54E-05 | 0.005 |
| ENSP00000284981 | APP | 7.54E-05 | 0.241 |
| ENSP00000333639 | IFNL2 | 7.54E-05 | 0.002 |
| ENSP00000354612 | PTGS1 | 7.53E-05 | 0.001 |
| ENSP00000308541 | F2 | 7.53E-05 | 0.112 |
| ENSP00000386331 | MYO7A | 7.53E-05 | 0.018 |
| ENSP00000354923 | DMD | 7.52E-05 | 0.054 |
| ENSP00000265709 | ANK1 | 7.51E-05 | 0.078 |
| ENSP00000229022 | VDR | 7.51E-05 | 0.025 |
| ENSP00000368683 | EDN1 | 7.49E-05 | 0.036 |
| ENSP00000358997 | IRAK1 | 7.49E-05 | 0.038 |
| ENSP00000349252 | ITGAL | 7.47E-05 | 0.01 |
| ENSP00000318480 | YME1L1 | 7.46E-05 | 0.036 |
| ENSP00000356832 | SGK1 | 7.42E-05 | 0.003 |
| ENSP00000356056 | DYNLT1 | 7.40E-05 | 0.007 |
| ENSP00000244741 | CDKN1A | 7.40E-05 | 0.167 |
| ENSP00000252321 | KCNA5 | 7.39E-05 | 0.016 |
| ENSP00000366563 | PIK3CD | 7.39E-05 | 0.039 |
| ENSP00000376886 | NME1-NME2 | 7.37E-05 | 0.003 |
| ENSP00000367851 | CYBB | 7.34E-05 | 0.003 |
| ENSP00000378546 | TAPBP | 7.33E-05 | 0.01 |
| ENSP00000263025 | MAPK3 | 7.33E-05 | 0.257 |
| ENSP00000358622 | IKBKG | 7.31E-05 | 0.099 |
| ENSP00000308610 | GPD2 | 7.31E-05 | 0.009 |
| ENSP00000303532 | DEFB4A | 7.30E-05 | 0.024 |
| ENSP00000263923 | KDR | 7.30E-05 | 0.011 |
| ENSP00000266557 | CD27 | 7.29E-05 | 0.015 |
| ENSP00000286758 | CXCL13 | 7.27E-05 | 0.012 |
| ENSP00000333685 | MAPK11 | 7.26E-05 | 0.049 |
| ENSP00000416599 | ZNRD1 | 7.26E-05 | 0.007 |
| ENSP00000361021 | PTEN | 7.26E-05 | 0.424 |
| ENSP00000296754 | ERAP1 | 7.24E-05 | 0.005 |
| ENSP00000283558 | ATP11A | 7.23E-05 | <0.001 |
| ENSP00000400175 | RHOA | 7.19E-05 | 0.092 |
| ENSP00000377218 | IRF2 | 7.18E-05 | 0.012 |
| ENSP00000264867 | PPARGC1A | 7.17E-05 | 0.009 |
| ENSP00000395337 | LDHA | 7.17E-05 | 0.018 |
| ENSP00000379110 | CXCL1 | 7.15E-05 | 0.018 |
| ENSP00000222823 | NOD1 | 7.14E-05 | 0.01 |
| ENSP00000397420 | HLA-E | 7.13E-05 | 0.004 |
| ENSP00000227758 | BIRC2 | 7.13E-05 | 0.012 |
| ENSP00000296930 | NPM1 | 7.11E-05 | 0.047 |
| ENSP00000382791 | GRIK1 | 7.10E-05 | 0.12 |
| ENSP00000397705 | HLA-F | 7.09E-05 | 0.024 |
| ENSP00000354558 | MTOR | 7.08E-05 | 0.12 |
| ENSP00000342681 | CD5 | 7.08E-05 | 0.042 |
| ENSP00000373023 | CDSN | 7.03E-05 | 0.042 |
| ENSP00000339007 | GRB2 | 7.02E-05 | 0.54 |
| ENSP00000259607 | CCL21 | 7.02E-05 | 0.002 |
| ENSP00000363435 | ITPR3 | 7.01E-05 | 0.022 |
| ENSP00000268296 | ITGAX | 7.01E-05 | 0.014 |
| ENSP00000296435 | CAMP | 6.98E-05 | 0.027 |
| ENSP00000370343 | IRF4 | 6.97E-05 | 0.007 |
| ENSP00000410076 | CASP1 | 6.96E-05 | 0.096 |
| ENSP00000381237 | HM13 | 6.90E-05 | 0.033 |
| ENSP00000353059 | APAF1 | 6.89E-05 | 0.06 |
| ENSP00000255409 | CHI3L1 | 6.86E-05 | 0.006 |
| ENSP00000268097 | HEXA | 6.85E-05 | 0.041 |
| ENSP00000233057 | EIF2AK2 | 6.85E-05 | 0.033 |
| ENSP00000308938 | PLG | 6.84E-05 | 0.108 |
| ENSP00000257979 | MIP | 6.84E-05 | 0.005 |
| ENSP00000229277 | ENO2 | 6.83E-05 | 0.035 |
| ENSP00000229708 | ULBP1 | 6.82E-05 | <0.001 |
| ENSP00000292807 | AP2M1 | 6.81E-05 | 0.051 |
| ENSP00000260630 | CYP1B1 | 6.80E-05 | 0.039 |
| ENSP00000295718 | PTPRN | 6.80E-05 | 0.025 |
| ENSP00000254325 | RFX1 | 6.79E-05 | 0.01 |
| ENSP00000052754 | DCN | 6.78E-05 | 0.105 |
| ENSP00000375629 | LILRB2 | 6.76E-05 | 0.029 |
| ENSP00000387477 | PRRC2A | 6.76E-05 | 0.025 |
| ENSP00000265431 | CALB1 | 6.75E-05 | 0.008 |
| ENSP00000362649 | HDAC1 | 6.74E-05 | 0.458 |
| ENSP00000296154 | CASR | 6.73E-05 | 0.036 |
| ENSP00000363804 | KLF4 | 6.73E-05 | 0.011 |
| ENSP00000309591 | PRKACA | 6.72E-05 | 0.102 |
| ENSP00000329357 | SP1 | 6.72E-05 | 0.115 |
| ENSP00000404619 | PRRC2A | 6.72E-05 | 0.023 |
| ENSP00000323036 | ACPP | 6.70E-05 | 0.052 |
| ENSP00000343325 | PKN1 | 6.70E-05 | 0.028 |
| ENSP00000360247 | CYP2J2 | 6.70E-05 | 0.024 |
| ENSP00000419945 | ERVW-1 | 6.69E-05 | 0.04 |
| ENSP00000370473 | IGFBP3 | 6.68E-05 | 0.012 |
| ENSP00000254322 | DNAJB1 | 6.66E-05 | 0.006 |
| ENSP00000307875 | B3GAT1 | 6.65E-05 | 0.014 |
| ENSP00000314458 | CDC42 | 6.64E-05 | 0.447 |
| ENSP00000363763 | EPHB2 | 6.63E-05 | 0.011 |
| ENSP00000362463 | GLO1 | 6.63E-05 | 0.023 |
| ENSP00000353731 | DPP4 | 6.62E-05 | 0.023 |
| ENSP00000372746 | HLA-DRA | 6.62E-05 | 0.025 |
| ENSP00000342070 | CTSB | 6.62E-05 | 0.027 |
| ENSP00000242152 | NPY | 6.62E-05 | 0.061 |
| ENSP00000246891 | CSN1S1 | 6.61E-05 | 0.334 |
| ENSP00000388386 | CDSN | 6.59E-05 | 0.031 |
| ENSP00000221466 | FCGRT | 6.59E-05 | 0.017 |
| ENSP00000399604 | CDSN | 6.59E-05 | 0.05 |
| ENSP00000368438 | PCNA | 6.58E-05 | 0.764 |
| ENSP00000364805 | HSPA1L | 6.56E-05 | 0.023 |
| ENSP00000352721 | DNM2 | 6.55E-05 | 0.04 |
| ENSP00000335620 | GSTA1 | 6.55E-05 | 0.02 |
| ENSP00000364076 | HLA-DQA2 | 6.53E-05 | 0.01 |
| ENSP00000282091 | PTH | 6.53E-05 | 0.09 |
| ENSP00000338072 | AVPR2 | 6.53E-05 | 0.063 |
| ENSP00000309503 | YWHAZ | 6.52E-05 | 0.197 |
| ENSP00000211122 | GSTA3 | 6.52E-05 | 0.018 |
| ENSP00000414905 | HLA-F | 6.51E-05 | 0.024 |
| ENSP00000420168 | GSTA2 | 6.49E-05 | 0.022 |
| ENSP00000276689 | NDUFB9 | 6.49E-05 | 0.017 |
| ENSP00000250151 | CCL4 | 6.49E-05 | 0.016 |
| ENSP00000315477 | CD209 | 6.48E-05 | 0.004 |
| ENSP00000273430 | AGTR1 | 6.47E-05 | 0.006 |
| ENSP00000370408 | CDX2 | 6.47E-05 | 0.018 |
| ENSP00000245479 | SOX9 | 6.45E-05 | 0.07 |
| ENSP00000368699 | ISG15 | 6.45E-05 | 0.026 |
| ENSP00000236959 | ATIC | 6.45E-05 | 0.052 |
| ENSP00000284240 | THY1 | 6.45E-05 | 0.008 |
| ENSP00000365465 | CDSN | 6.44E-05 | 0.027 |
| ENSP00000006053 | CX3CL1 | 6.43E-05 | 0.013 |
| ENSP00000223190 | NRF1 | 6.41E-05 | 0.008 |
| ENSP00000393355 | MICB | 6.41E-05 | <0.001 |
| ENSP00000256078 | KRAS | 6.41E-05 | 0.159 |
| ENSP00000224784 | ACTA2 | 6.40E-05 | 0.042 |
| ENSP00000351908 | MAP3K5 | 6.40E-05 | 0.026 |
| ENSP00000262160 | SMAD2 | 6.40E-05 | 0.205 |
| ENSP00000348831 | NUDT10 | 6.39E-05 | 0.014 |
| ENSP00000222248 | SLC5A5 | 6.38E-05 | 0.023 |
| ENSP00000405041 | POU5F1 | 6.38E-05 | 0.098 |
| ENSP00000028008 | RNASET2 | 6.37E-05 | 0.026 |
| ENSP00000354901 | CXCL9 | 6.37E-05 | 0.029 |
| ENSP00000295400 | TGFA | 6.37E-05 | 0.007 |
| ENSP00000358802 | KCNC4 | 6.37E-05 | 0.009 |
| ENSP00000362057 | NOX1 | 6.36E-05 | 0.011 |
| ENSP00000344352 | ATF3 | 6.36E-05 | 0.021 |
| ENSP00000395544 | NFKBIL1 | 6.35E-05 | <0.001 |
| ENSP00000245907 | C3 | 6.34E-05 | 0.058 |
| ENSP00000364898 | SYK | 6.34E-05 | 0.133 |
| ENSP00000352157 | MAPK10 | 6.34E-05 | 0.052 |
| ENSP00000394486 | NFKBIL1 | 6.33E-05 | <0.001 |
| ENSP00000268035 | IGF1R | 6.33E-05 | 0.047 |
| ENSP00000394842 | GCA | 6.32E-05 | 0.005 |
| ENSP00000364252 | PLA2G2A | 6.32E-05 | 0.072 |
| ENSP00000356946 | FCGR3A | 6.31E-05 | 0.045 |
| ENSP00000407561 | MICB | 6.30E-05 | <0.001 |
| ENSP00000268638 | IRF8 | 6.28E-05 | 0.01 |
| ENSP00000229402 | KLRB1 | 6.27E-05 | 0.014 |
| ENSP00000371327 | KLRC2 | 6.26E-05 | 0.006 |
| ENSP00000256010 | NTS | 6.25E-05 | 0.145 |
| ENSP00000362795 | CXCR3 | 6.24E-05 | 0.018 |
| ENSP00000264664 | FGF10 | 6.24E-05 | 0.035 |
| ENSP00000355812 | FGFR1OP | 6.23E-05 | 0.001 |
| ENSP00000346206 | TAP1 | 6.22E-05 | 0.044 |
| ENSP00000315768 | STAT2 | 6.21E-05 | 0.045 |
| ENSP00000375829 | LAD1 | 6.21E-05 | <0.001 |
| ENSP00000262445 | MAP2K4 | 6.21E-05 | 0.032 |
| ENSP00000328968 | SCN5A | 6.21E-05 | 0.005 |
| ENSP00000301974 | PTAFR | 6.20E-05 | 0.013 |
| ENSP00000264037 | TECTA | 6.19E-05 | 0.02 |
| ENSP00000216254 | ACO2 | 6.19E-05 | 0.008 |
| ENSP00000399298 | HLA-DPB1 | 6.19E-05 | 0.008 |
| ENSP00000365318 | NFKBIL1 | 6.18E-05 | <0.001 |
| ENSP00000238081 | YWHAQ | 6.18E-05 | 0.141 |
| ENSP00000228938 | MGP | 6.17E-05 | 0.05 |
| ENSP00000287295 | AIFM1 | 6.17E-05 | 0.036 |
| ENSP00000259808 | RIPK1 | 6.16E-05 | 0.018 |
| ENSP00000261170 | GUCY2C | 6.16E-05 | 0.035 |
| ENSP00000383142 | NFKBIL1 | 6.15E-05 | 0.002 |
| ENSP00000411611 | NFKBIL1 | 6.14E-05 | <0.001 |
| ENSP00000307235 | EIF2AK3 | 6.14E-05 | 0.014 |
| ENSP00000403495 | LTA | 6.13E-05 | 0.008 |
| ENSP00000301838 | FADD | 6.12E-05 | 0.022 |
| ENSP00000385269 | ELAVL1 | 6.11E-05 | 0.887 |
| ENSP00000365016 | IRS2 | 6.11E-05 | 0.026 |
| ENSP00000407133 | LTA | 6.10E-05 | 0.001 |
| ENSP00000416337 | LTA | 6.10E-05 | 0.001 |
| ENSP00000355988 | IRF6 | 6.10E-05 | 0.025 |
| ENSP00000408146 | HLA-DPB1 | 6.10E-05 | <0.001 |
| ENSP00000274026 | CCNA2 | 6.10E-05 | 0.04 |
| ENSP00000292199 | NLRX1 | 6.09E-05 | 0.016 |
| ENSP00000225655 | PFN1 | 6.09E-05 | 0.027 |
| ENSP00000263863 | GNLY | 6.08E-05 | 0.024 |
| ENSP00000241651 | MYOG | 6.07E-05 | 0.056 |
| ENSP00000327545 | NPTXR | 6.06E-05 | 0.016 |
| ENSP00000349893 | DLK2 | 6.06E-05 | <0.001 |
| ENSP00000356552 | MR1 | 6.06E-05 | <0.001 |
| ENSP00000256646 | NOTCH2 | 6.06E-05 | 0.038 |
| ENSP00000328150 | KCNJ12 | 6.05E-05 | 0.046 |
| ENSP00000366307 | THBD | 6.05E-05 | 0.004 |
| ENSP00000344460 | CBS | 6.05E-05 | 0.033 |
| ENSP00000264716 | FOSL2 | 6.05E-05 | 0.024 |
| ENSP00000219596 | MEFV | 6.03E-05 | 0.047 |
| ENSP00000350720 | SMARCA4 | 6.02E-05 | 0.139 |
| ENSP00000228307 | PXN | 6.01E-05 | 0.056 |
| ENSP00000255427 | CHIT1 | 6.01E-05 | 0.016 |
| ENSP00000220616 | TG | 6.00E-05 | 0.006 |
| ENSP00000351255 | STAT4 | 5.99E-05 | 0.028 |
| ENSP00000216194 | ADSL | 5.99E-05 | 0.036 |
| ENSP00000302707 | FPR1 | 5.99E-05 | 0.021 |
| ENSP00000315644 | TYMS | 5.98E-05 | 0.11 |
| ENSP00000384886 | IFI30 | 5.96E-05 | 0.016 |
| ENSP00000360973 | AGTR2 | 5.94E-05 | 0.003 |
| ENSP00000356771 | F5 | 5.94E-05 | 0.023 |
| ENSP00000231228 | IL12B | 5.94E-05 | 0.014 |
| ENSP00000355778 | H3F3A | 5.93E-05 | 0.357 |
| ENSP00000397587 | HLA-DPA1 | 5.93E-05 | 0.001 |
| ENSP00000393646 | HLA-DMB | 5.93E-05 | 0.013 |
| ENSP00000229319 | LDHB | 5.92E-05 | 0.018 |
| ENSP00000304236 | CD14 | 5.92E-05 | 0.04 |
| ENSP00000236147 | SELL | 5.92E-05 | 0.057 |
| ENSP00000330382 | PDGFB | 5.90E-05 | 0.044 |
| ENSP00000264498 | FGF2 | 5.89E-05 | 0.131 |
| ENSP00000395005 | MOG | 5.87E-05 | 0.008 |
| ENSP00000265800 | EPB49 | 5.87E-05 | 0.011 |
| ENSP00000356033 | TAGAP | 5.87E-05 | 0.044 |
| ENSP00000327336 | BGN | 5.86E-05 | 0.043 |
| ENSP00000319788 | NQO1 | 5.86E-05 | 0.004 |
| ENSP00000356320 | ULBP2 | 5.86E-05 | 0.007 |
| ENSP00000337915 | CYP3A4 | 5.85E-05 | 0.188 |
| ENSP00000391898 | MOG | 5.85E-05 | 0.006 |
| ENSP00000390785 | MOG | 5.85E-05 | 0.005 |
| ENSP00000366095 | MOG | 5.85E-05 | 0.005 |
| ENSP00000298171 | TSHR | 5.85E-05 | 0.034 |
| ENSP00000296585 | ITGA2 | 5.84E-05 | 0.03 |
| ENSP00000354566 | CD3E | 5.81E-05 | 0.036 |
| ENSP00000350512 | COPS5 | 5.81E-05 | 0.203 |
| ENSP00000332973 | SMAD3 | 5.80E-05 | 0.177 |
| ENSP00000357625 | BNIP3 | 5.80E-05 | 0.009 |
| ENSP00000354782 | CD247 | 5.80E-05 | 0.017 |
| ENSP00000392762 | DCT | 5.78E-05 | 0.015 |
| ENSP00000360968 | CYP4X1 | 5.78E-05 | 0.057 |
| ENSP00000226760 | WFS1 | 5.77E-05 | 0.004 |
| ENSP00000296271 | RHO | 5.77E-05 | 0.206 |
| ENSP00000330341 | SOCS3 | 5.76E-05 | 0.073 |
| ENSP00000245206 | GOT2 | 5.76E-05 | 0.117 |
| ENSP00000374467 | ABCC8 | 5.76E-05 | 0.017 |
| ENSP00000295598 | ATP1A1 | 5.75E-05 | 0.015 |
| ENSP00000381293 | NSF | 5.75E-05 | 0.088 |
| ENSP00000341940 | CAV3 | 5.75E-05 | 0.044 |
| ENSP00000366729 | TNFRSF9 | 5.75E-05 | 0.001 |
| ENSP00000284818 | LY96 | 5.74E-05 | 0.015 |
| ENSP00000296870 | IL3 | 5.74E-05 | 0.09 |
| ENSP00000218388 | TIMP1 | 5.73E-05 | 0.074 |
| ENSP00000363079 | MBL2 | 5.72E-05 | 0.033 |
| ENSP00000349959 | RICTOR | 5.72E-05 | 0.035 |
| ENSP00000297988 | AQP7 | 5.72E-05 | 0.016 |
| ENSP00000285930 | AKR1B1 | 5.71E-05 | 0.046 |
| ENSP00000290310 | KCNE2 | 5.70E-05 | 0.008 |
| ENSP00000248071 | KLF2 | 5.69E-05 | 0.012 |
| ENSP00000268704 | SPG7 | 5.69E-05 | 0.019 |
| ENSP00000356016 | CR1 | 5.69E-05 | 0.018 |
| ENSP00000364597 | PADI4 | 5.69E-05 | 0.008 |
| ENSP00000303276 | RNASE2 | 5.69E-05 | 0.044 |
| ENSP00000364801 | HSPA1B | 5.68E-05 | 0.016 |
| ENSP00000376445 | TIRAP | 5.68E-05 | 0.008 |
| ENSP00000247829 | TSPAN8 | 5.68E-05 | 0.01 |
| ENSP00000375391 | HSPA1B | 5.67E-05 | 0.016 |
| ENSP00000375399 | HSPA1B | 5.67E-05 | 0.016 |
| ENSP00000403530 | HSPA1B | 5.67E-05 | 0.016 |
| ENSP00000176195 | SCT | 5.66E-05 | 0.025 |
| ENSP00000357025 | CD48 | 5.65E-05 | 0.016 |
| ENSP00000353679 | MME | 5.65E-05 | 0.017 |
| ENSP00000268058 | PML | 5.65E-05 | 0.057 |
| ENSP00000280193 | VEGFC | 5.64E-05 | 0.017 |
| ENSP00000319635 | CXCR2 | 5.64E-05 | 0.032 |
| ENSP00000338548 | FGF1 | 5.64E-05 | 0.108 |
| ENSP00000335062 | PDCD1 | 5.63E-05 | 0.011 |
| ENSP00000330138 | SSTR3 | 5.63E-05 | 0.004 |
| ENSP00000297784 | TMC1 | 5.63E-05 | 0.036 |
| ENSP00000361202 | IRS4 | 5.62E-05 | 0.031 |
| ENSP00000341170 | PTN | 5.62E-05 | 0.032 |
| ENSP00000360312 | BSND | 5.61E-05 | 0.011 |
| ENSP00000381607 | GSTP1 | 5.61E-05 | 0.031 |
| ENSP00000361467 | DLG5 | 5.60E-05 | 0.01 |
| ENSP00000262065 | MMD | 5.59E-05 | 0.009 |
| ENSP00000239223 | DUSP1 | 5.58E-05 | 0.003 |
| ENSP00000300134 | STAT6 | 5.57E-05 | 0.046 |
| ENSP00000262539 | PTPN3 | 5.57E-05 | 0.031 |
| ENSP00000230568 | LY86 | 5.57E-05 | 0.057 |
| ENSP00000264335 | YWHAE | 5.57E-05 | 0.142 |
| ENSP00000353940 | PMEL | 5.57E-05 | 0.015 |
| ENSP00000379213 | PTHLH | 5.57E-05 | 0.051 |
| ENSP00000317714 | STX4 | 5.56E-05 | 0.037 |
| ENSP00000262735 | PPARA | 5.55E-05 | 0.144 |
| ENSP00000318355 | AQP10 | 5.54E-05 | 0.005 |
| ENSP00000243347 | TNFAIP6 | 5.54E-05 | 0.002 |
| ENSP00000318631 | HSD17B6 | 5.54E-05 | 0.109 |
| ENSP00000354111 | DNAJC5 | 5.54E-05 | 0.019 |
| ENSP00000008938 | PGLYRP1 | 5.53E-05 | 0.028 |
| ENSP00000285735 | RHOC | 5.52E-05 | 0.03 |
| ENSP00000221486 | RNASEH2A | 5.52E-05 | 0.167 |
| ENSP00000363092 | PRKG1 | 5.52E-05 | 0.099 |
| ENSP00000357013 | CD244 | 5.51E-05 | 0.014 |
| ENSP00000396308 | DHFR | 5.51E-05 | 0.036 |
| ENSP00000347427 | MINK1 | 5.51E-05 | 0.01 |
| ENSP00000248594 | PTPN12 | 5.51E-05 | 0.049 |
| ENSP00000360372 | CYP2C19 | 5.50E-05 | 0.111 |
| ENSP00000380073 | IRF9 | 5.50E-05 | 0.042 |
| ENSP00000321410 | MAPK9 | 5.49E-05 | 0.104 |
| ENSP00000290524 | RFX5 | 5.49E-05 | 0.027 |
| ENSP00000254810 | H3F3B | 5.49E-05 | 0.406 |
| ENSP00000387122 | CLEC16A | 5.48E-05 | 0.028 |
| ENSP00000293288 | BAX | 5.48E-05 | 0.082 |
| ENSP00000301141 | CYP2A6 | 5.48E-05 | 0.088 |
| ENSP00000362613 | CDX4 | 5.47E-05 | 0.012 |
| ENSP00000326432 | CCR8 | 5.47E-05 | 0.031 |
| ENSP00000220809 | PLAT | 5.47E-05 | 0.104 |
| ENSP00000286301 | CSF1R | 5.45E-05 | 0.208 |
| ENSP00000315011 | EDNRA | 5.44E-05 | 0.039 |
| ENSP00000219919 | AQP9 | 5.44E-05 | 0.004 |
| ENSP00000328511 | KCNA4 | 5.44E-05 | 0.035 |
| ENSP00000222982 | CYP3A5 | 5.44E-05 | 0.131 |
| ENSP00000333837 | MRPL12 | 5.43E-05 | 0.055 |
| ENSP00000401548 | TCF19 | 5.43E-05 | 0.039 |
| ENSP00000302812 | SP7 | 5.42E-05 | 0.019 |
| ENSP00000315130 | CLU | 5.42E-05 | 0.028 |
| ENSP00000261937 | FLT4 | 5.41E-05 | 0.017 |
| ENSP00000239940 | PFN2 | 5.41E-05 | 0.025 |
| ENSP00000278379 | SLC1A2 | 5.41E-05 | 0.008 |
| ENSP00000365572 | NME2 | 5.40E-05 | 0.022 |
| ENSP00000319591 | KCND3 | 5.40E-05 | 0.018 |
| ENSP00000222382 | CYP3A43 | 5.39E-05 | 0.126 |
| ENSP00000259089 | BLK | 5.36E-05 | 0.037 |
| ENSP00000355192 | CACNA1S | 5.36E-05 | 0.05 |
| ENSP00000352514 | RUNX2 | 5.36E-05 | 0.112 |
| ENSP00000258317 | NPL | 5.35E-05 | 0.033 |
| ENSP00000271348 | GJA5 | 5.35E-05 | 0.035 |
| ENSP00000269886 | SH3GL1 | 5.34E-05 | 0.171 |
| ENSP00000331172 | CD8B | 5.33E-05 | 0.028 |
| ENSP00000300161 | YWHAB | 5.33E-05 | 0.181 |
| ENSP00000386161 | WWOX | 5.33E-05 | 0.033 |
| ENSP00000314520 | KCNA2 | 5.33E-05 | 0.034 |
| ENSP00000217381 | SNTA1 | 5.33E-05 | 0.007 |
| ENSP00000236671 | CTSD | 5.32E-05 | 0.077 |
| ENSP00000299166 | NDUFB8 | 5.32E-05 | 0.026 |
| ENSP00000337088 | MEN1 | 5.32E-05 | 0.023 |
| ENSP00000329890 | DEFA5 | 5.31E-05 | 0.038 |
| ENSP00000331746 | CALCA | 5.31E-05 | 0.079 |
| ENSP00000348573 | AKAP9 | 5.30E-05 | 0.035 |
| ENSP00000225245 | CCL3 | 5.30E-05 | 0.008 |
| ENSP00000314949 | POLR2A | 5.30E-05 | 0.248 |
| ENSP00000340089 | TLR5 | 5.29E-05 | 0.02 |
| ENSP00000352608 | RYR1 | 5.29E-05 | 0.05 |
| ENSP00000372608 | HLA-DRA | 5.28E-05 | 0.025 |
| ENSP00000356399 | CFH | 5.28E-05 | 0.031 |
| ENSP00000220166 | CTSH | 5.28E-05 | 0.01 |
| ENSP00000394624 | OPRM1 | 5.28E-05 | 0.028 |
| ENSP00000231656 | CDX1 | 5.28E-05 | 0.016 |
| ENSP00000265773 | SMARCA2 | 5.28E-05 | 0.119 |
| ENSP00000265354 | SRF | 5.27E-05 | 0.266 |
| ENSP00000228280 | KITLG | 5.27E-05 | 0.196 |
| ENSP00000215479 | AMELY | 5.27E-05 | 0.05 |
| ENSP00000265969 | KCNC1 | 5.27E-05 | 0.028 |
| ENSP00000275015 | NFKBIE | 5.26E-05 | 0.007 |
| ENSP00000382362 | PHB2 | 5.26E-05 | 0.159 |
| ENSP00000357624 | MARCKS | 5.26E-05 | 0.007 |
| ENSP00000355675 | GJC2 | 5.26E-05 | 0.036 |
| ENSP00000355865 | PARK2 | 5.25E-05 | 0.252 |
| ENSP00000221515 | RETN | 5.25E-05 | 0.008 |
| ENSP00000262375 | DNAJA3 | 5.24E-05 | 0.021 |
| ENSP00000227378 | HSPA8 | 5.24E-05 | 0.113 |
| ENSP00000381599 | MX1 | 5.24E-05 | 0.04 |
| ENSP00000382697 | ROCK1 | 5.24E-05 | 0.022 |
| ENSP00000368880 | FOXO1 | 5.23E-05 | 0.035 |
| ENSP00000222390 | HGF | 5.23E-05 | 0.066 |
| ENSP00000353408 | MSN | 5.23E-05 | 0.06 |
| ENSP00000364694 | ASPN | 5.23E-05 | 0.08 |
| ENSP00000370571 | TH | 5.23E-05 | 0.116 |
| ENSP00000372881 | HSPA1L | 5.22E-05 | 0.016 |
| ENSP00000408347 | HSPA1L | 5.22E-05 | 0.016 |
| ENSP00000345708 | KCNJ11 | 5.22E-05 | 0.039 |
| ENSP00000229812 | STK38 | 5.21E-05 | 0.029 |
| ENSP00000348019 | SLC17A5 | 5.20E-05 | 0.011 |
| ENSP00000359425 | KCNQ5 | 5.20E-05 | 0.026 |
| ENSP00000402316 | TAP1 | 5.20E-05 | 0.035 |
| ENSP00000373477 | GPX3 | 5.20E-05 | 0.022 |
| ENSP00000353393 | F8 | 5.20E-05 | 0.067 |
| ENSP00000229264 | GNB3 | 5.20E-05 | 0.038 |
| ENSP00000301585 | ICT1 | 5.19E-05 | 0.128 |
| ENSP00000268182 | IQGAP1 | 5.19E-05 | 0.015 |
| ENSP00000278175 | ADM | 5.19E-05 | 0.033 |
| ENSP00000369213 | DDX58 | 5.19E-05 | 0.065 |
| ENSP00000363773 | C1QA | 5.18E-05 | 0.159 |
| ENSP00000257981 | KCNH3 | 5.18E-05 | 0.028 |
| ENSP00000297157 | RP9 | 5.17E-05 | 0.004 |
| ENSP00000263642 | IFIH1 | 5.17E-05 | 0.065 |
| ENSP00000000412 | M6PR | 5.17E-05 | 0.028 |
| ENSP00000338934 | EZR | 5.17E-05 | 0.147 |
| ENSP00000243457 | KCNJ2 | 5.17E-05 | 0.018 |
| ENSP00000262613 | SLC9A3R1 | 5.17E-05 | 0.026 |
| ENSP00000348234 | TAT | 5.17E-05 | 0.074 |
| ENSP00000296503 | HMGB2 | 5.15E-05 | 0.008 |
| ENSP00000408617 | HDAC9 | 5.15E-05 | 0.12 |
| ENSP00000345492 | SH2B3 | 5.15E-05 | 0.017 |
| ENSP00000281830 | KCNE4 | 5.15E-05 | <0.001 |
| ENSP00000371729 | SACS | 5.14E-05 | 0.014 |
| ENSP00000358786 | KCNA10 | 5.14E-05 | 0.012 |
| ENSP00000355136 | SORBS1 | 5.13E-05 | 0.008 |
| ENSP00000256649 | TRIM45 | 5.13E-05 | 0.007 |
| ENSP00000220849 | EIF3E | 5.13E-05 | 0.081 |
| ENSP00000274306 | GZMA | 5.12E-05 | 0.049 |
| ENSP00000313644 | MAP4K4 | 5.11E-05 | 0.009 |
| ENSP00000336832 | GJD3 | 5.11E-05 | 0.03 |
| ENSP00000274813 | MUT | 5.11E-05 | 0.094 |
| ENSP00000262999 | UCP1 | 5.10E-05 | 0.012 |
| ENSP00000228916 | SCNN1A | 5.10E-05 | 0.016 |
| ENSP00000267101 | ERBB3 | 5.09E-05 | 0.127 |
| ENSP00000212015 | SIRT1 | 5.09E-05 | 0.388 |
| ENSP00000316333 | CD55 | 5.07E-05 | 0.025 |
| ENSP00000215637 | MADCAM1 | 5.07E-05 | 0.015 |
| ENSP00000381822 | CDH23 | 5.07E-05 | 0.052 |
| ENSP00000306565 | ISG20 | 5.06E-05 | 0.006 |
| ENSP00000219660 | AQP8 | 5.05E-05 | 0.012 |
| ENSP00000261427 | UBE2K | 5.05E-05 | 0.102 |
| ENSP00000264818 | TYK2 | 5.05E-05 | 0.123 |
| ENSP00000305941 | USH2A | 5.04E-05 | 0.035 |
| ENSP00000354929 | NOTCH2NL | 5.04E-05 | 0.114 |
| ENSP00000346566 | CKAP5 | 5.04E-05 | 0.349 |
| ENSP00000222330 | GSK3A | 5.03E-05 | 0.037 |
| ENSP00000409378 | CHRM4 | 5.03E-05 | 0.024 |
| ENSP00000363641 | TXN | 5.03E-05 | 0.193 |
| ENSP00000356530 | RNASEL | 5.03E-05 | 0.021 |
| ENSP00000241125 | GJA3 | 5.02E-05 | 0.063 |
| ENSP00000356954 | CTGF | 5.02E-05 | 0.042 |
| ENSP00000370088 | AMELX | 5.01E-05 | 0.035 |
| ENSP00000383503 | ZNRD1 | 5.01E-05 | 0.017 |
| ENSP00000313967 | C1QB | 5.00E-05 | 0.138 |
| ENSP00000410071 | TUBB | 4.99E-05 | 0.049 |
| ENSP00000340811 | GJB5 | 4.99E-05 | 0.038 |
| ENSP00000404533 | HLA-DRA | 4.98E-05 | 0.024 |
| ENSP00000305372 | ADRB2 | 4.98E-05 | 0.06 |
| ENSP00000264110 | ATF2 | 4.98E-05 | 0.06 |
| ENSP00000254854 | GUCY2D | 4.98E-05 | 0.05 |
| ENSP00000410443 | HLA-DRA | 4.98E-05 | 0.026 |
| ENSP00000368966 | TRPC3 | 4.98E-05 | 0.047 |
| ENSP00000370880 | MLANA | 4.97E-05 | 0.021 |
| ENSP00000343505 | TNFSF13 | 4.97E-05 | 0.005 |
| ENSP00000252034 | ELN | 4.96E-05 | 0.125 |
| ENSP00000364217 | AGER | 4.96E-05 | 0.006 |
| ENSP00000274335 | PIK3R1 | 4.96E-05 | 0.361 |
| ENSP00000291700 | S100B | 4.95E-05 | 0.026 |
| ENSP00000318902 | FOXP1 | 4.95E-05 | 0.047 |
| ENSP00000307218 | NAT1 | 4.95E-05 | 0.022 |
| ENSP00000329380 | GP1BA | 4.95E-05 | 0.022 |
| ENSP00000417300 | TRBC2 | 4.95E-05 | 0.149 |
| ENSP00000328547 | DNMT3B | 4.95E-05 | 0.316 |
| ENSP00000312999 | GNAI2 | 4.94E-05 | 0.208 |
| ENSP00000261799 | PDGFRB | 4.94E-05 | 0.172 |
| ENSP00000289081 | FANCC | 4.94E-05 | 0.02 |
| ENSP00000357130 | SPTA1 | 4.94E-05 | 0.049 |
| ENSP00000368766 | ADRA1D | 4.93E-05 | 0.051 |
| ENSP00000346103 | GPX4 | 4.93E-05 | 0.037 |
| ENSP00000262570 | CHCHD3 | 4.93E-05 | 0.042 |
| ENSP00000216605 | MTHFD1 | 4.92E-05 | 0.017 |
| ENSP00000219700 | HMOX2 | 4.92E-05 | 0.084 |
| ENSP00000347184 | HTT | 4.92E-05 | 0.069 |
| ENSP00000254868 | CLEC10A | 4.91E-05 | 0.043 |
| ENSP00000405295 | HLA-DRA | 4.90E-05 | 0.022 |
| ENSP00000356708 | OLIG3 | 4.90E-05 | 0.06 |
| ENSP00000350132 | PLN | 4.90E-05 | 0.048 |
| ENSP00000220751 | RIPK2 | 4.90E-05 | 0.037 |
| ENSP00000261292 | LIPG | 4.90E-05 | 0.037 |
| ENSP00000348812 | PTPRD | 4.90E-05 | 0.081 |
| ENSP00000314709 | ZHX2 | 4.90E-05 | 0.056 |
| ENSP00000265724 | ABCB1 | 4.89E-05 | 0.126 |
| ENSP00000300289 | PDIA3 | 4.89E-05 | 0.061 |
| ENSP00000329623 | BCL2 | 4.89E-05 | 0.113 |
| ENSP00000349588 | ANK2 | 4.89E-05 | 0.003 |
| ENSP00000347948 | TNFRSF14 | 4.88E-05 | 0.048 |
| ENSP00000415793 | MUC5B | 4.88E-05 | 0.138 |
| ENSP00000261416 | HEXB | 4.88E-05 | 0.052 |
| ENSP00000354947 | CAPZA2 | 4.88E-05 | 0.057 |
| ENSP00000340292 | DLK1 | 4.87E-05 | 0.04 |
| ENSP00000297185 | HSPA9 | 4.87E-05 | 0.159 |
| ENSP00000323568 | SLC2A2 | 4.86E-05 | 0.029 |
| ENSP00000222307 | KXD1 | 4.86E-05 | 0.037 |
| ENSP00000329869 | TPO | 4.86E-05 | 0.009 |
| ENSP00000347046 | PDE5A | 4.86E-05 | <0.001 |
| ENSP00000378723 | HLA-DMB | 4.85E-05 | 0.007 |
| ENSP00000265421 | POLB | 4.85E-05 | 0.002 |
| ENSP00000280200 | CD226 | 4.84E-05 | 0.02 |
| ENSP00000339001 | TUBB | 4.84E-05 | 0.048 |
| ENSP00000396127 | RAN | 4.83E-05 | 0.609 |
| ENSP00000398890 | HLA-DMB | 4.83E-05 | 0.007 |
| ENSP00000347314 | SPG20 | 4.83E-05 | 0.011 |
| ENSP00000263754 | KAT2B | 4.83E-05 | 0.265 |
| ENSP00000415786 | SERPINE2 | 4.82E-05 | 0.022 |
| ENSP00000348307 | SIRPA | 4.80E-05 | 0.166 |
| ENSP00000276571 | CRH | 4.80E-05 | 0.08 |
| ENSP00000263734 | EPAS1 | 4.80E-05 | 0.021 |
| ENSP00000263168 | CAPZA1 | 4.80E-05 | 0.054 |
| ENSP00000306361 | FGA | 4.79E-05 | 0.065 |
| ENSP00000359504 | GBP1 | 4.79E-05 | 0.019 |
| ENSP00000371328 | KLRC3 | 4.79E-05 | 0.034 |
| ENSP00000370109 | PSIP1 | 4.79E-05 | 0.032 |
| ENSP00000240986 | GJA8 | 4.79E-05 | 0.087 |
| ENSP00000360869 | IFIT1 | 4.78E-05 | 0.053 |
| ENSP00000298942 | PTER | 4.78E-05 | 0.085 |
| ENSP00000401317 | TUBB | 4.78E-05 | 0.043 |
| ENSP00000402951 | HLA-DRA | 4.78E-05 | 0.014 |
| ENSP00000039007 | OTC | 4.77E-05 | 0.091 |
| ENSP00000348918 | SAA1 | 4.76E-05 | 0.009 |
| ENSP00000293330 | HCRT | 4.76E-05 | 0.042 |
| ENSP00000292401 | AZGP1 | 4.76E-05 | 0.031 |
| ENSP00000379908 | ICA1 | 4.76E-05 | 0.035 |
| ENSP00000261745 | NAA25 | 4.75E-05 | 0.01 |
| ENSP00000355645 | ACTA1 | 4.75E-05 | 0.236 |
| ENSP00000343023 | SP100 | 4.75E-05 | 0.029 |
| ENSP00000408534 | TNFAIP8 | 4.75E-05 | 0.001 |
| ENSP00000415941 | C4B | 4.74E-05 | 0.007 |
| ENSP00000405636 | ZNRD1 | 4.74E-05 | 0.023 |
| ENSP00000304822 | CSN3 | 4.74E-05 | 0.026 |
| ENSP00000237014 | TTR | 4.74E-05 | 0.078 |
| ENSP00000404817 | TRGC1 | 4.73E-05 | 0.084 |
| ENSP00000286627 | KCNMA1 | 4.72E-05 | 0.066 |
| ENSP00000357206 | NES | 4.71E-05 | 0.103 |
| ENSP00000261254 | CCND2 | 4.71E-05 | 0.084 |
| ENSP00000225964 | COL1A1 | 4.71E-05 | 0.235 |
| ENSP00000351314 | PSMB10 | 4.70E-05 | 0.044 |
| ENSP00000371372 | ATP12A | 4.70E-05 | 0.044 |
| ENSP00000261740 | TRPV4 | 4.70E-05 | 0.04 |
| ENSP00000170630 | IL4R | 4.69E-05 | 0.013 |
| ENSP00000318340 | AC026703.1 | 4.68E-05 | 0.018 |
| ENSP00000306124 | PRKCE | 4.68E-05 | 0.089 |
| ENSP00000347197 | C5AR1 | 4.68E-05 | 0.025 |
| ENSP00000364000 | COL5A2 | 4.68E-05 | 0.102 |
| ENSP00000366488 | PRKACG | 4.67E-05 | 0.112 |
| ENSP00000356329 | RAET1G | 4.67E-05 | <0.001 |
| ENSP00000262290 | LPO | 4.66E-05 | <0.001 |
| ENSP00000266646 | INHBE | 4.65E-05 | 0.111 |
| ENSP00000255226 | SLC14A2 | 4.65E-05 | 0.035 |
| ENSP00000272371 | OTOF | 4.65E-05 | 0.022 |
| ENSP00000339587 | DFNA5 | 4.65E-05 | 0.028 |
| ENSP00000344456 | CTNNB1 | 4.65E-05 | 0.732 |
| ENSP00000373298 | COLQ | 4.64E-05 | 0.071 |
| ENSP00000357494 | ROS1 | 4.64E-05 | 0.092 |
| ENSP00000311857 | PTPN2 | 4.64E-05 | 0.021 |
| ENSP00000291860 | KIR3DL3 | 4.63E-05 | 0.008 |
| ENSP00000354900 | GJB1 | 4.63E-05 | 0.049 |
| ENSP00000266376 | CACNA1C | 4.62E-05 | 0.097 |
| ENSP00000261652 | TNFRSF13B | 4.62E-05 | 0.014 |
| ENSP00000325663 | NFKBIZ | 4.62E-05 | 0.011 |
| ENSP00000341268 | TRADD | 4.62E-05 | 0.038 |
| ENSP00000288709 | MMEL1 | 4.62E-05 | 0.001 |
| ENSP00000350348 | GRM7 | 4.62E-05 | 0.026 |
| ENSP00000308928 | CYLD | 4.61E-05 | 0.079 |
| ENSP00000363827 | HSPG2 | 4.61E-05 | 0.173 |
| ENSP00000264033 | CBL | 4.60E-05 | 0.218 |
| ENSP00000261514 | CLCN3 | 4.60E-05 | 0.014 |
| ENSP00000314733 | TOLLIP | 4.60E-05 | 0.057 |
| ENSP00000338297 | IGF2 | 4.59E-05 | 0.113 |
| ENSP00000406878 | PSMB8 | 4.59E-05 | 0.109 |
| ENSP00000241261 | TNFSF10 | 4.59E-05 | 0.05 |
| ENSP00000295463 | ALPI | 4.57E-05 | 0.012 |
| ENSP00000262623 | ATP4A | 4.57E-05 | 0.029 |
| ENSP00000354859 | DRD2 | 4.56E-05 | 0.127 |
| ENSP00000347942 | RET | 4.56E-05 | 0.262 |
| ENSP00000253727 | NR1H2 | 4.56E-05 | 0.227 |
| ENSP00000221452 | RELB | 4.56E-05 | 0.035 |
| ENSP00000297436 | DEFA6 | 4.56E-05 | 0.067 |
| ENSP00000360891 | IFIT2 | 4.55E-05 | 0.058 |
| ENSP00000332468 | TRAF3 | 4.55E-05 | 0.061 |
| ENSP00000351706 | GK2 | 4.55E-05 | 0.019 |
| ENSP00000418001 | GK5 | 4.55E-05 | 0.016 |
| ENSP00000289429 | CD1A | 4.55E-05 | 0.045 |
| ENSP00000278070 | PPRC1 | 4.54E-05 | 0.004 |
| ENSP00000264381 | BCHE | 4.54E-05 | 0.043 |
| ENSP00000379500 | UEVLD | 4.54E-05 | 0.017 |
| ENSP00000321334 | LPA | 4.54E-05 | 0.029 |
| ENSP00000293276 | CCL15 | 4.54E-05 | 0.004 |
| ENSP00000308176 | BTK | 4.54E-05 | 0.107 |
| ENSP00000367959 | HTR2A | 4.54E-05 | 0.046 |
| ENSP00000359497 | GBP2 | 4.53E-05 | 0.02 |
| ENSP00000333193 | GJC1 | 4.53E-05 | 0.03 |
| ENSP00000350815 | NR3C2 | 4.53E-05 | 0.027 |
| ENSP00000320493 | TRIP10 | 4.53E-05 | 0.023 |
| ENSP00000223366 | GCK | 4.52E-05 | 0.031 |
| ENSP00000355153 | CDKN2A | 4.52E-05 | 0.422 |
| ENSP00000350937 | TES | 4.51E-05 | 0.105 |
| ENSP00000348793 | AFF3 | 4.51E-05 | 0.041 |
| ENSP00000324856 | STK11 | 4.50E-05 | 0.073 |
| ENSP00000265986 | IDE | 4.50E-05 | 0.17 |
| ENSP00000376966 | KCNC2 | 4.50E-05 | 0.03 |
| ENSP00000380227 | ITGA4 | 4.50E-05 | 0.117 |
| ENSP00000228438 | CLEC2B | 4.50E-05 | 0.021 |
| ENSP00000363081 | DKK1 | 4.50E-05 | 0.072 |
| ENSP00000234313 | PLEK | 4.50E-05 | 0.531 |
| ENSP00000305958 | STIP1 | 4.49E-05 | 0.025 |
| ENSP00000248923 | GGT1 | 4.49E-05 | 0.117 |
| ENSP00000338812 | C1QTNF6 | 4.49E-05 | 0.035 |
| ENSP00000400047 | POU5F1 | 4.48E-05 | 0.095 |
| ENSP00000248572 | GNGT1 | 4.48E-05 | 0.046 |
| ENSP00000349076 | ATN1 | 4.48E-05 | 0.22 |
| ENSP00000256458 | IRAK2 | 4.48E-05 | 0.053 |
| ENSP00000357727 | S100A9 | 4.48E-05 | 0.076 |
| ENSP00000230671 | SLC6A7 | 4.47E-05 | 0.27 |
| ENSP00000158762 | ACAP1 | 4.47E-05 | 0.037 |
| ENSP00000359539 | GOT1 | 4.47E-05 | 0.098 |
| ENSP00000407291 | RNF39 | 4.46E-05 | 0.008 |
| ENSP00000408539 | RNF39 | 4.46E-05 | 0.008 |
| ENSP00000265643 | GAL | 4.46E-05 | 0.054 |
| ENSP00000357103 | DARC | 4.46E-05 | 0.038 |
| ENSP00000248244 | TICAM1 | 4.45E-05 | 0.006 |
| ENSP00000323479 | B3GALNT1 | 4.44E-05 | 0.041 |
| ENSP00000359301 | MAGEA3 | 4.44E-05 | 0.081 |
| ENSP00000356671 | SERPINC1 | 4.44E-05 | 0.262 |
| ENSP00000355533 | RYR2 | 4.44E-05 | 0.079 |
| ENSP00000347858 | XIAP | 4.43E-05 | 0.062 |
| ENSP00000361824 | SPTAN1 | 4.43E-05 | 0.073 |
| ENSP00000234347 | PRTN3 | 4.43E-05 | 0.035 |
| ENSP00000252660 | MAS1 | 4.43E-05 | 0.068 |
| ENSP00000357838 | OAT | 4.43E-05 | 0.053 |
| ENSP00000221403 | DHDH | 4.42E-05 | 0.022 |
| ENSP00000359211 | DPYD | 4.42E-05 | 0.056 |
| ENSP00000359345 | RPL5 | 4.42E-05 | 0.231 |
| ENSP00000354652 | NPY1R | 4.42E-05 | 0.02 |
| ENSP00000342300 | NELFCD | 4.41E-05 | 0.018 |
| ENSP00000231188 | GRM6 | 4.41E-05 | 0.037 |
| ENSP00000352071 | CD163 | 4.40E-05 | 0.051 |
| ENSP00000375557 | POU5F1B | 4.40E-05 | 0.069 |
| ENSP00000351602 | FUT4 | 4.40E-05 | 0.074 |
| ENSP00000349709 | RAET1E | 4.40E-05 | 0.001 |
| ENSP00000358081 | BAG3 | 4.40E-05 | 0.018 |
| ENSP00000276570 | DNAJC5B | 4.40E-05 | 0.019 |
| ENSP00000355470 | CNST | 4.40E-05 | 0.02 |
| ENSP00000320709 | ADIPOQ | 4.39E-05 | 0.014 |
| ENSP00000217305 | PDYN | 4.39E-05 | 0.074 |
| ENSP00000228872 | CDKN1B | 4.39E-05 | 0.235 |
| ENSP00000358022 | MCL1 | 4.39E-05 | 0.241 |
| ENSP00000379566 | CCHCR1 | 4.39E-05 | 0.023 |
| ENSP00000255631 | HSPBP1 | 4.39E-05 | 0.019 |
| ENSP00000221130 | GSR | 4.38E-05 | 0.095 |
| ENSP00000264954 | GRPEL1 | 4.38E-05 | 0.041 |
| ENSP00000332124 | MRC1L1 | 4.37E-05 | 0.019 |
| ENSP00000365431 | TCF19 | 4.37E-05 | 0.018 |
| ENSP00000339399 | CRYZ | 4.37E-05 | 0.127 |
| ENSP00000266839 | MMAB | 4.37E-05 | 0.018 |
| ENSP00000293826 | TNFSF12-TNFSF13 | 4.37E-05 | 0.014 |
| ENSP00000343318 | B3GALT5 | 4.37E-05 | 0.033 |
| ENSP00000406872 | HLA-DQB2 | 4.37E-05 | 0.012 |
| ENSP00000341189 | PTK2 | 4.37E-05 | 0.165 |
| ENSP00000354130 | SOX10 | 4.36E-05 | 0.088 |
| ENSP00000294312 | FGF19 | 4.35E-05 | 0.1 |
| ENSP00000362082 | CCND3 | 4.35E-05 | 0.069 |
| ENSP00000381331 | HDAC2 | 4.35E-05 | 0.592 |
| ENSP00000188790 | FAP | 4.35E-05 | 0.004 |
| ENSP00000393566 | HLA-DPA1 | 4.35E-05 | 0.002 |
| ENSP00000296140 | CCR1 | 4.35E-05 | 0.08 |
| ENSP00000361965 | ADA | 4.35E-05 | 0.123 |
| ENSP00000409159 | HLA-DQB2 | 4.35E-05 | 0.015 |
| ENSP00000399259 | FCHSD1 | 4.35E-05 | 0.027 |
| ENSP00000276414 | GNRH1 | 4.34E-05 | 0.233 |
| ENSP00000302269 | VAV1 | 4.33E-05 | 0.204 |
| ENSP00000331327 | WT1 | 4.33E-05 | 0.111 |
| ENSP00000321106 | TAC1 | 4.33E-05 | 0.201 |
| ENSP00000345464 | NHLRC1 | 4.33E-05 | 0.016 |
| ENSP00000322170 | PFN4 | 4.33E-05 | 0.057 |
| ENSP00000263867 | CAPG | 4.33E-05 | 0.005 |
| ENSP00000350928 | GAD1 | 4.32E-05 | 0.189 |
| ENSP00000279441 | MMP10 | 4.32E-05 | 0.025 |
| ENSP00000379651 | LILRA6 | 4.32E-05 | 0.019 |
| ENSP00000274695 | CDKAL1 | 4.32E-05 | 0.035 |
| ENSP00000325120 | PGR | 4.31E-05 | 0.157 |
| ENSP00000359540 | CREG1 | 4.31E-05 | 0.029 |
| ENSP00000223642 | C5 | 4.31E-05 | 0.035 |
| ENSP00000357721 | S100A8 | 4.30E-05 | 0.076 |
| ENSP00000334564 | POLR3C | 4.30E-05 | 0.112 |
| ENSP00000244360 | RNF39 | 4.30E-05 | 0.008 |
| ENSP00000266970 | CDK2 | 4.30E-05 | 0.635 |
| ENSP00000405108 | HLA-DOB | 4.30E-05 | 0.032 |
| ENSP00000249071 | RAC2 | 4.30E-05 | 0.497 |
| ENSP00000295683 | CXCR1 | 4.29E-05 | 0.055 |
| ENSP00000381793 | GRB10 | 4.29E-05 | 0.069 |
| ENSP00000365012 | HCK | 4.29E-05 | 0.104 |
| ENSP00000263043 | ICK | 4.28E-05 | 0.155 |
| ENSP00000250018 | TPH1 | 4.28E-05 | 0.045 |
| ENSP00000334448 | GNG2 | 4.28E-05 | 0.22 |
| ENSP00000401149 | TAP1 | 4.28E-05 | 0.013 |
| ENSP00000395780 | HLA-DOB | 4.28E-05 | 0.031 |
| ENSP00000286380 | RAET1L | 4.28E-05 | 0.001 |
| ENSP00000334122 | FGF3 | 4.27E-05 | 0.033 |
| ENSP00000343477 | RUNX3 | 4.27E-05 | 0.057 |
| ENSP00000271450 | FCGR2A | 4.27E-05 | 0.07 |
| ENSP00000297435 | DEFA4 | 4.26E-05 | 0.068 |
| ENSP00000286452 | KIF5A | 4.26E-05 | 0.061 |
| ENSP00000226218 | SEBOX | 4.26E-05 | 0.118 |
| ENSP00000358092 | PRDM1 | 4.25E-05 | 0.032 |
| ENSP00000218721 | MLNR | 4.25E-05 | 0.009 |
| ENSP00000318212 | KCNH6 | 4.24E-05 | 0.039 |
| ENSP00000244769 | ATXN1 | 4.24E-05 | 0.295 |
| ENSP00000325875 | HSP90AB1 | 4.23E-05 | 0.069 |
| ENSP00000217426 | AHCY | 4.22E-05 | 0.028 |
| ENSP00000266086 | SLC5A4 | 4.22E-05 | 0.011 |
| ENSP00000309572 | TERT | 4.22E-05 | 0.102 |
| ENSP00000402406 | PSMB8 | 4.22E-05 | 0.039 |
| ENSP00000360626 | KCNG1 | 4.22E-05 | 0.047 |
| ENSP00000349275 | NRG1 | 4.21E-05 | 0.084 |
| ENSP00000300692 | CD3D | 4.21E-05 | 0.086 |
| ENSP00000352673 | ELF3 | 4.21E-05 | 0.108 |
| ENSP00000306884 | CXCL11 | 4.21E-05 | 0.021 |
| ENSP00000348602 | AMPH | 4.21E-05 | 0.114 |
| ENSP00000252483 | PVRL2 | 4.20E-05 | 0.045 |
| ENSP00000406797 | PSMB8 | 4.20E-05 | 0.067 |
| ENSP00000237527 | GHRH | 4.19E-05 | 0.123 |
| ENSP00000351497 | FCGR2B | 4.19E-05 | 0.03 |
| ENSP00000349577 | PRODH | 4.18E-05 | 0.088 |
| ENSP00000350415 | GJA9 | 4.18E-05 | 0.024 |
| ENSP00000311489 | SPTBN2 | 4.18E-05 | 0.069 |
| ENSP00000371798 | FSCN1 | 4.18E-05 | 0.047 |
| ENSP00000342278 | OAS2 | 4.18E-05 | 0.056 |
| ENSP00000230990 | HBEGF | 4.18E-05 | 0.106 |
| ENSP00000383199 | NEDD4L | 4.18E-05 | 0.095 |
| ENSP00000267436 | L2HGDH | 4.18E-05 | 0.023 |
| ENSP00000361423 | ABL1 | 4.17E-05 | 0.414 |
| ENSP00000347032 | PIP4K2C | 4.17E-05 | 0.025 |
| ENSP00000344479 | NR4A2 | 4.17E-05 | 0.049 |
| ENSP00000237596 | PKD2 | 4.17E-05 | 0.08 |
| ENSP00000391181 | CCHCR1 | 4.17E-05 | 0.024 |
| ENSP00000225983 | HDAC5 | 4.17E-05 | 0.109 |
| ENSP00000360217 | RHAG | 4.17E-05 | 0.027 |
| ENSP00000331827 | TNFAIP8L1 | 4.16E-05 | 0.011 |
| ENSP00000356713 | IFNGR1 | 4.16E-05 | 0.065 |
| ENSP00000304915 | IL13 | 4.16E-05 | 0.034 |
| ENSP00000001008 | FKBP4 | 4.15E-05 | 0.021 |
| ENSP00000344193 | RNASE1 | 4.15E-05 | 0.161 |
| ENSP00000255192 | BHMT2 | 4.15E-05 | 0.064 |
| ENSP00000223023 | WASL | 4.14E-05 | 0.099 |
| ENSP00000396320 | SCN4A | 4.14E-05 | 0.021 |
| ENSP00000257749 | BACH2 | 4.14E-05 | 0.055 |
| ENSP00000363071 | DES | 4.13E-05 | 0.211 |
| ENSP00000312673 | GH1 | 4.13E-05 | 0.032 |
| ENSP00000290378 | ACTC1 | 4.13E-05 | 0.158 |
| ENSP00000229134 | IL26 | 4.13E-05 | 0.019 |
| ENSP00000328236 | KNTC1 | 4.13E-05 | 0.473 |
| ENSP00000358784 | KCNA3 | 4.13E-05 | 0.034 |
| ENSP00000238607 | PGF | 4.12E-05 | 0.028 |
| ENSP00000227667 | APOC3 | 4.12E-05 | 0.097 |
| ENSP00000414817 | HLA-DMB | 4.11E-05 | 0.023 |
| ENSP00000273920 | ENOPH1 | 4.11E-05 | 0.065 |
| ENSP00000357731 | LOR | 4.11E-05 | 0.173 |
| ENSP00000205948 | APOH | 4.10E-05 | 0.087 |
| ENSP00000263360 | EED | 4.10E-05 | 0.288 |
| ENSP00000308815 | CCL19 | 4.10E-05 | 0.063 |
| ENSP00000362924 | GSN | 4.10E-05 | 0.077 |
| ENSP00000403721 | CYP21A2 | 4.10E-05 | 0.021 |
| ENSP00000340191 | FPR2 | 4.09E-05 | 0.07 |
| ENSP00000335657 | CCK | 4.09E-05 | 0.096 |
| ENSP00000366124 | CST3 | 4.09E-05 | 0.039 |
| ENSP00000382166 | CX3CR1 | 4.08E-05 | 0.036 |
| ENSP00000364979 | COL4A1 | 4.08E-05 | 0.1 |
| ENSP00000301633 | BIRC5 | 4.08E-05 | 0.397 |
| ENSP00000281834 | TNFSF4 | 4.07E-05 | 0.006 |
| ENSP00000333496 | KCND2 | 4.07E-05 | 0.056 |
| ENSP00000341828 | CHIA | 4.07E-05 | 0.019 |
| ENSP00000225844 | CCL13 | 4.07E-05 | 0.025 |
| ENSP00000329127 | PRKCH | 4.07E-05 | 0.054 |
| ENSP00000311469 | GSTM1 | 4.07E-05 | 0.074 |
| ENSP00000270861 | PLK4 | 4.07E-05 | 0.188 |
| ENSP00000266003 | MLN | 4.07E-05 | 0.019 |
| ENSP00000419425 | PPIA | 4.06E-05 | 0.094 |
| ENSP00000331358 | GAST | 4.06E-05 | 0.094 |
| ENSP00000276914 | PLIN2 | 4.06E-05 | 0.033 |
| ENSP00000201586 | SULT2B1 | 4.05E-05 | 0.215 |
| ENSP00000369981 | SH3GL2 | 4.04E-05 | 0.071 |
| ENSP00000389875 | PSORS1C1 | 4.04E-05 | 0.06 |
| ENSP00000356429 | RGS1 | 4.04E-05 | 0.088 |
| ENSP00000365175 | PRRC2A | 4.04E-05 | 0.062 |
| ENSP00000399968 | NCOA2 | 4.04E-05 | 0.253 |
| ENSP00000380150 | CARD11 | 4.03E-05 | 0.022 |
| ENSP00000360286 | RAE1 | 4.03E-05 | 0.179 |
| ENSP00000306920 | GLB1 | 4.03E-05 | 0.137 |
| ENSP00000406495 | TNXB | 4.03E-05 | 0.019 |
| ENSP00000360157 | FOXD3 | 4.03E-05 | 0.033 |
| ENSP00000377192 | G6PD | 4.03E-05 | 0.121 |
| ENSP00000264870 | F13A1 | 4.02E-05 | 0.055 |
| ENSP00000303507 | BCR | 4.02E-05 | 0.262 |
| ENSP00000387438 | TNXB | 4.02E-05 | 0.018 |
| ENSP00000326366 | PSEN1 | 4.02E-05 | 0.146 |
| ENSP00000389677 | TNXB | 4.02E-05 | 0.018 |
| ENSP00000248975 | YWHAH | 4.01E-05 | 0.155 |
| ENSP00000370115 | SERPINB1 | 4.01E-05 | 0.069 |
| ENSP00000364016 | PSMB8 | 4.00E-05 | 0.048 |
| ENSP00000365131 | BAG6 | 4.00E-05 | 0.051 |
| ENSP00000407942 | C4B | 3.99E-05 | 0.004 |
| ENSP00000251241 | DHX40 | 3.99E-05 | 0.043 |
| ENSP00000372726 | TAP2 | 3.99E-05 | 0.017 |
| ENSP00000233146 | MSH2 | 3.99E-05 | 0.497 |
| ENSP00000331111 | ZNRD1 | 3.99E-05 | 0.03 |
| ENSP00000402278 | C2 | 3.99E-05 | 0.032 |
| ENSP00000415363 | PRRC2A | 3.98E-05 | 0.059 |
| ENSP00000310305 | P2RY2 | 3.98E-05 | 0.056 |
| ENSP00000299367 | C2 | 3.97E-05 | 0.035 |
| ENSP00000272065 | ACP1 | 3.97E-05 | 0.031 |
| ENSP00000235933 | CD160 | 3.97E-05 | 0.017 |
| ENSP00000255040 | APCS | 3.97E-05 | 0.046 |
| ENSP00000292144 | CD3G | 3.97E-05 | 0.025 |
| ENSP00000222139 | EPOR | 3.96E-05 | 0.114 |
| ENSP00000349493 | GUCA2A | 3.95E-05 | 0.025 |
| ENSP00000345731 | DLG1 | 3.95E-05 | 0.137 |
| ENSP00000296122 | PPP1CB | 3.94E-05 | 0.363 |
| ENSP00000358301 | ADRB1 | 3.94E-05 | 0.115 |
| ENSP00000282397 | FLT1 | 3.94E-05 | 0.135 |
| ENSP00000230354 | TBP | 3.93E-05 | 0.31 |
| ENSP00000257904 | CDK4 | 3.93E-05 | 0.565 |
| ENSP00000342235 | ERBB4 | 3.93E-05 | 0.257 |
| ENSP00000359799 | DNAJB4 | 3.93E-05 | 0.014 |
| ENSP00000318687 | HSPH1 | 3.93E-05 | 0.037 |
| ENSP00000406965 | ABHD16A | 3.92E-05 | 0.064 |
| ENSP00000350283 | BRCA1 | 3.92E-05 | 0.788 |
| ENSP00000348170 | HP | 3.92E-05 | 0.108 |
| ENSP00000220003 | CSK | 3.91E-05 | 0.087 |
| ENSP00000306523 | INSM2 | 3.91E-05 | 0.004 |
| ENSP00000333657 | MX2 | 3.91E-05 | 0.039 |
| ENSP00000360502 | PDE6C | 3.91E-05 | 0.062 |
| ENSP00000318868 | SHMT1 | 3.91E-05 | 0.09 |
| ENSP00000324633 | DEFB103B | 3.91E-05 | 0.112 |
| ENSP00000221476 | CKM | 3.91E-05 | 0.045 |
| ENSP00000296223 | POLR2H | 3.90E-05 | 0.276 |
| ENSP00000372599 | TAP2 | 3.90E-05 | 0.02 |
| ENSP00000331817 | ALYREF | 3.90E-05 | 0.242 |
| ENSP00000315602 | CHRNA3 | 3.90E-05 | 0.048 |
| ENSP00000268171 | FURIN | 3.90E-05 | 0.353 |
| ENSP00000360216 | DNTT | 3.89E-05 | 0.052 |
| ENSP00000231004 | LOX | 3.89E-05 | 0.143 |
| ENSP00000323377 | EXOC3 | 3.88E-05 | 0.06 |
| ENSP00000352324 | ATXN3 | 3.88E-05 | 0.086 |
| ENSP00000360683 | PTPN1 | 3.88E-05 | 0.127 |
| ENSP00000358421 | HSD3B1 | 3.88E-05 | 0.1 |
| ENSP00000217407 | LBP | 3.88E-05 | 0.018 |
| ENSP00000272233 | RHOB | 3.88E-05 | 0.138 |
| ENSP00000268605 | NOL3 | 3.88E-05 | 0.048 |
| ENSP00000327850 | NFATC1 | 3.87E-05 | 0.038 |
| ENSP00000350003 | CCR3 | 3.86E-05 | 0.08 |
| ENSP00000262158 | SMAD7 | 3.86E-05 | 0.073 |
| ENSP00000378786 | HLA-DRA | 3.86E-05 | 0.042 |
| ENSP00000282096 | PDE3B | 3.86E-05 | 0.034 |
| ENSP00000312735 | POLR2B | 3.85E-05 | 0.489 |
| ENSP00000386341 | TICAM2 | 3.85E-05 | 0.009 |
| ENSP00000324827 | AKAP17A | 3.85E-05 | 0.013 |
| ENSP00000372547 | SRY | 3.85E-05 | 0.235 |
| ENSP00000244043 | PTGIS | 3.85E-05 | 0.008 |
| ENSP00000262888 | KCNN4 | 3.85E-05 | 0.025 |
| ENSP00000357158 | FCRL1 | 3.84E-05 | 0.02 |
| ENSP00000350005 | GIP | 3.84E-05 | 0.024 |
| ENSP00000368104 | BMP2 | 3.84E-05 | 0.328 |
| ENSP00000306330 | YWHAG | 3.83E-05 | 0.382 |
| ENSP00000409132 | HLA-G | 3.83E-05 | 0.039 |
| ENSP00000363480 | DLG3 | 3.83E-05 | 0.199 |
| ENSP00000281821 | EPHA4 | 3.82E-05 | 0.064 |
| ENSP00000278616 | ATM | 3.82E-05 | 0.596 |
| ENSP00000253699 | ZFYVE20 | 3.82E-05 | 0.022 |
| ENSP00000324248 | PENK | 3.82E-05 | 0.064 |
| ENSP00000257497 | ANXA1 | 3.82E-05 | 0.208 |
| ENSP00000342830 | RDX | 3.82E-05 | 0.096 |
| ENSP00000347409 | KEL | 3.81E-05 | 0.066 |
| ENSP00000357060 | ATP1A4 | 3.81E-05 | 0.037 |
| ENSP00000304783 | SLC26A5 | 3.81E-05 | 0.024 |
| ENSP00000356489 | EPM2A | 3.81E-05 | 0.016 |
| ENSP00000363840 | COL11A2 | 3.81E-05 | 0.066 |
| ENSP00000408860 | CYP21A2 | 3.81E-05 | 0.022 |
| ENSP00000363680 | EDA | 3.81E-05 | 0.298 |
| ENSP00000394942 | CYP21A2 | 3.80E-05 | 0.022 |
| ENSP00000271411 | POU2F1 | 3.80E-05 | 0.063 |
| ENSP00000227471 | UNC93B1 | 3.80E-05 | 0.045 |
| ENSP00000322439 | TUFM | 3.80E-05 | 0.159 |
| ENSP00000369127 | DNAJA1 | 3.80E-05 | 0.067 |
| ENSP00000255465 | CCNA1 | 3.80E-05 | 0.155 |
| ENSP00000314151 | KLK3 | 3.80E-05 | 0.186 |
| ENSP00000397598 | KY | 3.80E-05 | 0.024 |
| ENSP00000405965 | SUMO2 | 3.79E-05 | 0.879 |
| ENSP00000267843 | FGF7 | 3.79E-05 | 0.069 |
| ENSP00000261917 | HCN4 | 3.79E-05 | 0.041 |
| ENSP00000359042 | PRPF38B | 3.78E-05 | 0.214 |
| ENSP00000360806 | KCNB1 | 3.78E-05 | 0.063 |
| ENSP00000260356 | THBS1 | 3.77E-05 | 0.067 |
| ENSP00000261532 | ESRRB | 3.77E-05 | 0.063 |
| ENSP00000322788 | MMP1 | 3.77E-05 | 0.077 |
| ENSP00000325775 | GJC3 | 3.77E-05 | 0.041 |
| ENSP00000413625 | FNBP1 | 3.76E-05 | 0.044 |
| ENSP00000288071 | DDX19B | 3.76E-05 | 0.042 |
| ENSP00000357459 | ADAR | 3.76E-05 | 0.075 |
| ENSP00000339467 | RHOG | 3.76E-05 | 0.162 |
| ENSP00000408453 | HLA-DMB | 3.76E-05 | 0.047 |
| ENSP00000361465 | POLR1C | 3.76E-05 | 0.266 |
| ENSP00000332766 | NPB | 3.75E-05 | 0.001 |
| ENSP00000361777 | SET | 3.75E-05 | 0.339 |
| ENSP00000279593 | GRIN2B | 3.75E-05 | 0.24 |
| ENSP00000357167 | FCRL3 | 3.75E-05 | 0.03 |
| ENSP00000378890 | GCH1 | 3.75E-05 | 0.067 |
| ENSP00000364396 | TNXB | 3.75E-05 | 0.025 |
| ENSP00000284719 | OLA1 | 3.73E-05 | 0.065 |
| ENSP00000353622 | SIN3A | 3.73E-05 | 0.342 |
| ENSP00000215631 | GADD45B | 3.73E-05 | 0.026 |
| ENSP00000366006 | UBIAD1 | 3.73E-05 | 0.203 |
| ENSP00000395497 | GTF2H4 | 3.73E-05 | 0.02 |
| ENSP00000351410 | PRKAR1A | 3.73E-05 | 0.149 |
| ENSP00000400326 | DDX39B | 3.72E-05 | 0.14 |
| ENSP00000360876 | IFIT3 | 3.72E-05 | 0.119 |
| ENSP00000354581 | GPX6 | 3.72E-05 | 0.009 |
| ENSP00000329558 | GRPEL2 | 3.72E-05 | 0.055 |
| ENSP00000261707 | SLC6A4 | 3.72E-05 | 0.257 |
| ENSP00000343676 | GJA4 | 3.72E-05 | 0.058 |
| ENSP00000040663 | MRI1 | 3.72E-05 | 0.021 |
| ENSP00000365991 | DNAJC3 | 3.72E-05 | 0.036 |
| ENSP00000322191 | DEFB104B | 3.72E-05 | 0.048 |
| ENSP00000358430 | TECTB | 3.71E-05 | 0.037 |
| ENSP00000319248 | ZEB1 | 3.71E-05 | 0.045 |
| ENSP00000354490 | ATP1A2 | 3.71E-05 | 0.054 |
| ENSP00000215838 | TCN2 | 3.70E-05 | 0.026 |
| ENSP00000373169 | DRD3 | 3.70E-05 | 0.08 |
| ENSP00000361311 | TMEM53 | 3.70E-05 | 0.08 |
| ENSP00000308782 | GP6 | 3.70E-05 | 0.045 |
| ENSP00000357879 | PSMD4 | 3.70E-05 | 0.214 |
| ENSP00000275525 | IGFBP1 | 3.70E-05 | 0.193 |
| ENSP00000263233 | SYP | 3.70E-05 | 0.352 |
| ENSP00000346534 | SCN8A | 3.69E-05 | 0.061 |
| ENSP00000383746 | FCAMR | 3.69E-05 | 0.038 |
| ENSP00000369456 | ITPA | 3.69E-05 | 0.186 |
| ENSP00000379282 | ABHD16A | 3.69E-05 | 0.039 |
| ENSP00000300093 | PLK1 | 3.69E-05 | 0.588 |
| ENSP00000265729 | SRI | 3.68E-05 | 0.068 |
| ENSP00000217182 | EEF1A2 | 3.68E-05 | 0.026 |
| ENSP00000333994 | HBB | 3.68E-05 | 0.14 |
| ENSP00000005257 | RALA | 3.68E-05 | 0.189 |
| ENSP00000228928 | OAS3 | 3.68E-05 | 0.08 |
| ENSP00000344871 | MYO1F | 3.67E-05 | 0.35 |
| ENSP00000270538 | TIMM44 | 3.67E-05 | 0.048 |
| ENSP00000221413 | RUVBL2 | 3.67E-05 | 0.449 |
| ENSP00000332816 | PTK2B | 3.66E-05 | 0.126 |
| ENSP00000244241 | IL17C | 3.66E-05 | 0.062 |
| ENSP00000263331 | POLR1B | 3.65E-05 | 0.211 |
| ENSP00000266427 | ETV6 | 3.65E-05 | 0.306 |
| ENSP00000348634 | MYH6 | 3.65E-05 | 0.185 |
| ENSP00000389155 | ABHD16A | 3.65E-05 | 0.026 |
| ENSP00000306397 | UQCRFS1 | 3.65E-05 | 0.092 |
| ENSP00000373106 | RNF39 | 3.65E-05 | 0.072 |
| ENSP00000403576 | GNL1 | 3.64E-05 | 0.065 |
| ENSP00000409367 | GNL1 | 3.64E-05 | 0.065 |
| ENSP00000354554 | MT-CYB | 3.64E-05 | 0.13 |
| ENSP00000272167 | EPHX1 | 3.64E-05 | 0.058 |
| ENSP00000365272 | DLG2 | 3.64E-05 | 0.053 |
| ENSP00000348912 | ADAM33 | 3.64E-05 | 0.033 |
| ENSP00000329991 | IFNL1 | 3.64E-05 | 0.039 |
| ENSP00000227752 | IL10RA | 3.64E-05 | 0.093 |
| ENSP00000281317 | MMAA | 3.63E-05 | 0.039 |
| ENSP00000283936 | KCNJ16 | 3.63E-05 | 0.043 |
| ENSP00000257770 | NT5E | 3.63E-05 | 0.4 |
| ENSP00000231487 | SKP1 | 3.63E-05 | 0.632 |
| ENSP00000299402 | APBB1 | 3.63E-05 | 0.095 |
| ENSP00000399542 | DDX39B | 3.62E-05 | 0.063 |
| ENSP00000301788 | POLR2G | 3.62E-05 | 0.331 |
| ENSP00000370003 | BNIP3L | 3.62E-05 | 0.016 |
| ENSP00000222286 | GAPDHS | 3.62E-05 | 0.031 |
| ENSP00000215587 | POLR2E | 3.62E-05 | 0.532 |
| ENSP00000264834 | KLF1 | 3.62E-05 | 0.054 |
| ENSP00000303211 | ACHE | 3.62E-05 | 0.077 |
| ENSP00000353259 | CD300A | 3.62E-05 | 0.03 |
| ENSP00000343957 | IFNAR2 | 3.61E-05 | 0.067 |
| ENSP00000296026 | CXCL3 | 3.61E-05 | 0.038 |
| ENSP00000329418 | SOCS1 | 3.61E-05 | 0.092 |
| ENSP00000300061 | SCNN1G | 3.61E-05 | 0.052 |
| ENSP00000257570 | OASL | 3.61E-05 | 0.076 |
| ENSP00000222271 | COMP | 3.61E-05 | 0.294 |
| ENSP00000265814 | RUNX1T1 | 3.60E-05 | 0.308 |
| ENSP00000302564 | BCL2L1 | 3.60E-05 | 0.2 |
| ENSP00000293195 | FDXR | 3.60E-05 | 0.044 |
| ENSP00000354950 | PCDH15 | 3.59E-05 | 0.181 |
| ENSP00000257248 | GIF | 3.59E-05 | 0.023 |
| ENSP00000360262 | FGGY | 3.59E-05 | 0.03 |
| ENSP00000340937 | COL17A1 | 3.59E-05 | 0.072 |
| ENSP00000312288 | HADH | 3.59E-05 | 0.4 |
| ENSP00000296577 | ABCE1 | 3.59E-05 | 0.757 |
| ENSP00000354632 | MT-ATP6 | 3.59E-05 | 0.016 |
| ENSP00000274520 | IL9 | 3.59E-05 | 0.045 |
| ENSP00000261448 | CASQ2 | 3.58E-05 | 0.032 |
| ENSP00000261187 | SLC16A7 | 3.58E-05 | 0.024 |
| ENSP00000314508 | GBA | 3.58E-05 | 0.116 |
| ENSP00000340913 | TRPC6 | 3.58E-05 | 0.082 |
| ENSP00000200676 | CETP | 3.58E-05 | 0.018 |
| ENSP00000253861 | EXOC4 | 3.57E-05 | 0.085 |
| ENSP00000256999 | FOLH1 | 3.57E-05 | 0.041 |
| ENSP00000311545 | EMR1 | 3.57E-05 | 0.07 |
| ENSP00000247153 | CFP | 3.57E-05 | 0.034 |
| ENSP00000282486 | MBNL1 | 3.57E-05 | 0.141 |
| ENSP00000338983 | MUC1 | 3.56E-05 | 0.24 |
| ENSP00000216181 | MYH9 | 3.56E-05 | 0.191 |
| ENSP00000401632 | GSTT1 | 3.56E-05 | 0.076 |
| ENSP00000251595 | HBA2 | 3.56E-05 | 0.056 |
| ENSP00000280614 | CCRN4L | 3.56E-05 | 0.006 |
| ENSP00000318635 | SUMO4 | 3.55E-05 | 0.06 |
| ENSP00000398278 | PSORS1C1 | 3.55E-05 | 0.086 |
| ENSP00000290374 | GJD2 | 3.55E-05 | 0.033 |
| ENSP00000365806 | GNL1 | 3.55E-05 | 0.051 |
| ENSP00000302176 | ASPSCR1 | 3.55E-05 | 0.087 |
| ENSP00000363868 | ABCA1 | 3.55E-05 | 0.177 |
| ENSP00000350767 | HIST4H4 | 3.55E-05 | 0.234 |
| ENSP00000394290 | GNL1 | 3.55E-05 | 0.046 |
| ENSP00000296273 | RFC4 | 3.54E-05 | 0.76 |
| ENSP00000303231 | IL12A | 3.54E-05 | 0.012 |
| ENSP00000379475 | DDX39B | 3.54E-05 | 0.054 |
| ENSP00000164247 | KCNAB2 | 3.54E-05 | 0.103 |
| ENSP00000299767 | HSP90B1 | 3.54E-05 | 0.069 |
| ENSP00000318799 | BHLHE22 | 3.54E-05 | 0.05 |
| ENSP00000403852 | POLR2F | 3.53E-05 | 0.346 |
| ENSP00000265371 | NRP1 | 3.53E-05 | 0.085 |
| ENSP00000306157 | IL7R | 3.53E-05 | 0.041 |
| ENSP00000232501 | NPRL2 | 3.53E-05 | 0.098 |
| ENSP00000316244 | HTR1A | 3.52E-05 | 0.072 |
| ENSP00000296027 | CXCL5 | 3.52E-05 | 0.039 |
| ENSP00000274353 | BHMT | 3.52E-05 | 0.02 |
| ENSP00000345868 | GJB4 | 3.52E-05 | 0.023 |
| ENSP00000387930 | RNF39 | 3.52E-05 | 0.008 |
| ENSP00000263036 | OPTN | 3.52E-05 | 0.064 |
| ENSP00000244061 | RNF114 | 3.51E-05 | 0.027 |
| ENSP00000295113 | FRZB | 3.51E-05 | 0.151 |
| ENSP00000267996 | TPM1 | 3.51E-05 | 0.248 |
| ENSP00000350467 | NAT9 | 3.51E-05 | 0.055 |
| ENSP00000349085 | MAGEA1 | 3.50E-05 | 0.15 |
| ENSP00000346879 | NKX2-1 | 3.50E-05 | 0.13 |
| ENSP00000391681 | POU5F1 | 3.50E-05 | 0.189 |
| ENSP00000216802 | PSME2 | 3.49E-05 | 0.008 |
| ENSP00000323155 | DAND5 | 3.49E-05 | 0.146 |
| ENSP00000405306 | NCR3 | 3.49E-05 | 0.068 |
| ENSP00000389071 | NCR3 | 3.49E-05 | 0.067 |
| ENSP00000302234 | CCL11 | 3.48E-05 | 0.026 |
| ENSP00000417281 | MDM2 | 3.48E-05 | 0.567 |
| ENSP00000323280 | CD6 | 3.48E-05 | 0.037 |
| ENSP00000302079 | C3AR1 | 3.48E-05 | 0.12 |
| ENSP00000319308 | RGS5 | 3.48E-05 | 0.036 |
| ENSP00000397181 | RGS4 | 3.48E-05 | 0.066 |
| ENSP00000322460 | SCN4B | 3.48E-05 | 0.064 |
| ENSP00000260227 | MMP7 | 3.48E-05 | 0.078 |
| ENSP00000367034 | HIST1H4C | 3.48E-05 | 0.234 |
| ENSP00000262809 | ELL | 3.48E-05 | 0.196 |
| ENSP00000316032 | NUP98 | 3.47E-05 | 0.115 |
| ENSP00000381045 | UBE3A | 3.47E-05 | 0.284 |
| ENSP00000379457 | FAF1 | 3.47E-05 | 0.03 |
| ENSP00000271751 | KCNH1 | 3.47E-05 | 0.087 |
| ENSP00000245451 | BMP4 | 3.46E-05 | 0.652 |
| ENSP00000265372 | CREM | 3.46E-05 | 0.148 |
| ENSP00000370719 | ITSN1 | 3.46E-05 | 0.188 |
| ENSP00000363431 | NPY4R | 3.46E-05 | 0.043 |
| ENSP00000256442 | CCNB1 | 3.46E-05 | 0.733 |
| ENSP00000357892 | FAM24B | 3.46E-05 | 0.02 |
| ENSP00000378517 | SPP1 | 3.46E-05 | 0.097 |
| ENSP00000316152 | SFTPC | 3.46E-05 | 0.052 |
| ENSP00000308620 | RAG2 | 3.45E-05 | 0.11 |
| ENSP00000354511 | COMT | 3.45E-05 | 0.226 |
| ENSP00000354251 | NCKAP1 | 3.45E-05 | 0.047 |
| ENSP00000320516 | EHD1 | 3.45E-05 | 0.064 |
| ENSP00000273183 | STAC | 3.45E-05 | 0.04 |
| ENSP00000358548 | NRAS | 3.45E-05 | 0.634 |
| ENSP00000264731 | TP63 | 3.45E-05 | 0.285 |
| ENSP00000364831 | CLCNKB | 3.45E-05 | 0.027 |
| ENSP00000307713 | BDKRB2 | 3.45E-05 | 0.081 |
| ENSP00000304111 | ABCG4 | 3.45E-05 | 0.078 |
| ENSP00000274629 | KCNMB1 | 3.45E-05 | 0.025 |
| ENSP00000306279 | UMOD | 3.45E-05 | 0.06 |
| ENSP00000335311 | EDNRB | 3.44E-05 | 0.096 |
| ENSP00000219302 | NME3 | 3.44E-05 | 0.051 |
| ENSP00000415481 | PROM1 | 3.44E-05 | 0.086 |
| ENSP00000342082 | SLPI | 3.43E-05 | 0.049 |
| ENSP00000354159 | NLRC4 | 3.43E-05 | 0.039 |
| ENSP00000396915 | SCN1B | 3.43E-05 | 0.079 |
| ENSP00000233202 | SLC11A1 | 3.42E-05 | 0.064 |
| ENSP00000372721 | PSMB9 | 3.42E-05 | 0.085 |
| ENSP00000243050 | NR4A1 | 3.42E-05 | 0.099 |
| ENSP00000219548 | STUB1 | 3.42E-05 | 0.214 |
| ENSP00000294053 | CLPB | 3.42E-05 | 0.15 |
| ENSP00000299022 | LIPC | 3.41E-05 | 0.133 |
| ENSP00000333950 | FMN1 | 3.41E-05 | 0.091 |
| ENSP00000263774 | NDUFS3 | 3.41E-05 | 0.264 |
| ENSP00000264218 | NMU | 3.41E-05 | 0.138 |
| ENSP00000264010 | CTCF | 3.41E-05 | 0.17 |
| ENSP00000361366 | SFTPD | 3.41E-05 | 0.074 |
| ENSP00000261304 | GALC | 3.41E-05 | 0.099 |
| ENSP00000314897 | ANGPT2 | 3.41E-05 | 0.033 |
| ENSP00000358541 | SIKE1 | 3.40E-05 | 0.112 |
| ENSP00000351926 | AP2A1 | 3.40E-05 | 0.17 |
| ENSP00000333666 | ADI1 | 3.40E-05 | 0.02 |
| ENSP00000359727 | BAG2 | 3.40E-05 | 0.05 |
| ENSP00000358490 | CD2 | 3.40E-05 | 0.196 |
| ENSP00000356515 | UTRN | 3.40E-05 | 0.048 |
| ENSP00000360532 | CDC5L | 3.40E-05 | 0.501 |
| ENSP00000358511 | CNR1 | 3.40E-05 | 0.089 |
| ENSP00000355069 | PAX2 | 3.40E-05 | 0.406 |
| ENSP00000274364 | IQGAP2 | 3.39E-05 | 0.006 |
| ENSP00000337383 | NLRP3 | 3.39E-05 | 0.063 |
| ENSP00000298596 | STOX1 | 3.39E-05 | 0.046 |
| ENSP00000229195 | CNOT2 | 3.39E-05 | 0.062 |
| ENSP00000225698 | C1QBP | 3.39E-05 | 0.78 |
| ENSP00000244289 | LIPE | 3.38E-05 | 0.159 |
| ENSP00000312122 | SEC13 | 3.37E-05 | 0.171 |
| ENSP00000356969 | APOA2 | 3.37E-05 | 0.147 |
| ENSP00000295989 | CAND2 | 3.37E-05 | 0.02 |
| ENSP00000261203 | LIN7A | 3.37E-05 | 0.06 |
| ENSP00000274793 | PLA2G7 | 3.36E-05 | 0.079 |
| ENSP00000297848 | COL14A1 | 3.36E-05 | 0.301 |
| ENSP00000253083 | HIP1R | 3.36E-05 | 0.089 |
| ENSP00000280684 | KCNA6 | 3.36E-05 | 0.054 |
| ENSP00000370376 | DUT | 3.36E-05 | 0.133 |
| ENSP00000264972 | ZAP70 | 3.36E-05 | 0.08 |
| ENSP00000317955 | EEA1 | 3.35E-05 | 0.245 |
| ENSP00000310623 | ISCU | 3.35E-05 | 0.055 |
| ENSP00000388340 | CLINT1 | 3.35E-05 | 0.101 |
| ENSP00000339328 | PLAUR | 3.34E-05 | 0.231 |
| ENSP00000216160 | TAB1 | 3.34E-05 | 0.048 |
| ENSP00000221444 | KCNA7 | 3.34E-05 | 0.065 |
| ENSP00000347582 | CGB8 | 3.34E-05 | 0.02 |
| ENSP00000333667 | SHMT2 | 3.34E-05 | 0.152 |
| ENSP00000324740 | YES1 | 3.34E-05 | 0.361 |
| ENSP00000369654 | HBD | 3.33E-05 | 0.137 |
| ENSP00000348984 | SON | 3.33E-05 | 0.16 |
| ENSP00000308549 | ADORA1 | 3.33E-05 | 0.049 |
| ENSP00000313437 | MMP19 | 3.33E-05 | 0.003 |
| ENSP00000217244 | CSNK2A1 | 3.33E-05 | 0.4 |
| ENSP00000269485 | TNFRSF11A | 3.33E-05 | 0.094 |
| ENSP00000351490 | MAX | 3.32E-05 | 0.192 |
| ENSP00000299642 | CLEC3A | 3.32E-05 | 0.034 |
| ENSP00000312987 | HNF4A | 3.32E-05 | 0.389 |
| ENSP00000337632 | SARNP | 3.32E-05 | 0.044 |
| ENSP00000308895 | SLC19A1 | 3.32E-05 | 0.021 |
| ENSP00000250003 | MYOD1 | 3.31E-05 | 0.647 |
| ENSP00000317334 | TCP1 | 3.31E-05 | 0.575 |
| ENSP00000416293 | SLC2A1 | 3.30E-05 | 0.126 |
| ENSP00000338814 | BAG5 | 3.30E-05 | 0.04 |
| ENSP00000341208 | STAT5A | 3.30E-05 | 0.072 |
| ENSP00000355718 | DLL1 | 3.30E-05 | 0.217 |
| ENSP00000219252 | POLR2C | 3.30E-05 | 0.467 |
| ENSP00000216200 | PVALB | 3.30E-05 | 0.08 |
| ENSP00000391901 | PHF1 | 3.29E-05 | 0.132 |
| ENSP00000357152 | CD1C | 3.29E-05 | 0.012 |
| ENSP00000216336 | CTSG | 3.29E-05 | 0.117 |
| ENSP00000326031 | PPP1CA | 3.29E-05 | 0.796 |
| ENSP00000283243 | PLA2R1 | 3.29E-05 | 0.034 |
| ENSP00000296028 | PPBP | 3.28E-05 | 0.095 |
| ENSP00000369003 | TRPC4 | 3.28E-05 | 0.119 |
| ENSP00000367276 | CKAP2 | 3.28E-05 | 0.996 |
| ENSP00000283254 | SCN3A | 3.28E-05 | 0.075 |
| ENSP00000357643 | MKI67 | 3.28E-05 | 0.106 |
| ENSP00000336764 | OPRL1 | 3.28E-05 | 0.048 |
| ENSP00000360181 | SH2D1A | 3.27E-05 | 0.058 |
| ENSP00000340278 | PARK7 | 3.27E-05 | 0.202 |
| ENSP00000296456 | APEH | 3.27E-05 | 0.089 |
| ENSP00000302967 | HDAC3 | 3.27E-05 | 0.647 |
| ENSP00000343027 | GNAI1 | 3.27E-05 | 0.319 |
| ENSP00000263946 | PKP1 | 3.26E-05 | 0.038 |
| ENSP00000312988 | NFKBIB | 3.26E-05 | 0.055 |
| ENSP00000361173 | KCNE1L | 3.26E-05 | 0.043 |
| ENSP00000329212 | ATF7 | 3.26E-05 | 0.032 |
| ENSP00000211998 | VCL | 3.25E-05 | 0.303 |
| ENSP00000368632 | GATA3 | 3.25E-05 | 0.098 |
| ENSP00000331310 | MORF4L1 | 3.25E-05 | 0.75 |
| ENSP00000379330 | NFATC2 | 3.25E-05 | 0.085 |
| ENSP00000317337 | CD300LB | 3.25E-05 | 0.036 |
| ENSP00000288235 | MYO1E | 3.25E-05 | 0.091 |
| ENSP00000216223 | IL2RB | 3.25E-05 | 0.107 |
| ENSP00000334052 | LGMN | 3.25E-05 | 0.063 |
| ENSP00000331727 | KCNH7 | 3.24E-05 | 0.102 |
| ENSP00000292614 | POLR2J | 3.24E-05 | 0.118 |
| ENSP00000266088 | SLC5A1 | 3.24E-05 | 0.076 |
| ENSP00000336630 | ADORA2A | 3.24E-05 | 0.055 |
| ENSP00000328813 | KCNH8 | 3.24E-05 | 0.077 |
| ENSP00000365576 | UBQLN1 | 3.23E-05 | 0.182 |
| ENSP00000241453 | FLT3 | 3.23E-05 | 0.082 |
| ENSP00000349954 | CGB | 3.22E-05 | 0.016 |
| ENSP00000306185 | ANTXR2 | 3.22E-05 | 0.062 |
| ENSP00000251377 | LILRA2 | 3.22E-05 | 0.029 |
| ENSP00000357244 | CCT3 | 3.22E-05 | 0.38 |
| ENSP00000384806 | PDE4D | 3.22E-05 | 0.054 |
| ENSP00000231368 | LNPEP | 3.22E-05 | 0.058 |
| ENSP00000352401 | RPE | 3.21E-05 | 0.077 |
| ENSP00000264563 | IL11 | 3.21E-05 | 0.025 |
| ENSP00000219479 | NME4 | 3.21E-05 | 0.063 |
| ENSP00000324270 | OXTR | 3.21E-05 | 0.145 |
| ENSP00000246957 | TRAP1 | 3.20E-05 | 0.058 |
| ENSP00000360222 | TM2D1 | 3.20E-05 | 0.069 |
| ENSP00000363958 | BRD2 | 3.20E-05 | 0.165 |
| ENSP00000348069 | SREBF1 | 3.20E-05 | 0.132 |
| ENSP00000305422 | CEBPB | 3.20E-05 | 0.218 |
| ENSP00000370737 | GLDC | 3.19E-05 | 0.117 |
| ENSP00000334003 | INTU | 3.19E-05 | 0.245 |
| ENSP00000358617 | PHTF1 | 3.19E-05 | 0.177 |
| ENSP00000386444 | SAG | 3.19E-05 | 0.113 |
| ENSP00000294702 | GFI1 | 3.19E-05 | 0.046 |
| ENSP00000363640 | PHF1 | 3.19E-05 | 0.026 |
| ENSP00000252971 | MNX1 | 3.19E-05 | 0.472 |
| ENSP00000262741 | PIK3R3 | 3.19E-05 | 0.215 |
| ENSP00000330237 | CASP9 | 3.19E-05 | 0.175 |
| ENSP00000305416 | S1PR1 | 3.19E-05 | 0.061 |
| ENSP00000172229 | NGFR | 3.18E-05 | 0.204 |
| ENSP00000325822 | CYP11B2 | 3.18E-05 | 0.051 |
| ENSP00000301408 | CGB5 | 3.18E-05 | 0.009 |
| ENSP00000412297 | PPP1R11 | 3.18E-05 | 0.025 |
| ENSP00000249042 | TST | 3.18E-05 | 0.158 |
| ENSP00000381486 | NOTO | 3.18E-05 | 0.02 |
| ENSP00000323856 | PLEC | 3.18E-05 | 0.096 |
| ENSP00000379616 | MYH11 | 3.18E-05 | 0.257 |
| ENSP00000326699 | CLGN | 3.18E-05 | 0.116 |
| ENSP00000291842 | SHKBP1 | 3.18E-05 | 0.055 |
| ENSP00000330959 | IL1R2 | 3.18E-05 | 0.022 |
| ENSP00000379865 | ADHFE1 | 3.18E-05 | 0.123 |
| ENSP00000264606 | HDAC4 | 3.18E-05 | 0.268 |
| ENSP00000367891 | LGALS7 | 3.18E-05 | 0.27 |
| ENSP00000337797 | PIDD | 3.17E-05 | 0.095 |
| ENSP00000348258 | HIST1H4L | 3.17E-05 | 0.346 |
| ENSP00000322421 | HBA1 | 3.17E-05 | 0.061 |
| ENSP00000413234 | AP2A2 | 3.17E-05 | 0.09 |
| ENSP00000365466 | PSORS1C1 | 3.17E-05 | 0.107 |
| ENSP00000357651 | TUBE1 | 3.17E-05 | 0.011 |
| ENSP00000360125 | PGM1 | 3.17E-05 | 0.127 |
| ENSP00000346402 | RNH1 | 3.17E-05 | 0.06 |
| ENSP00000298902 | IFI27 | 3.17E-05 | 0.077 |
| ENSP00000358994 | MYO6 | 3.17E-05 | 0.155 |
| ENSP00000372136 | DEFA1B | 3.17E-05 | 0.111 |
| ENSP00000321326 | F2R | 3.16E-05 | 0.227 |
| ENSP00000342056 | CS | 3.16E-05 | 0.164 |
| ENSP00000355896 | TGFB2 | 3.16E-05 | 0.115 |
| ENSP00000312126 | ADCY4 | 3.16E-05 | 0.428 |
| ENSP00000265175 | SEC24B | 3.16E-05 | 0.317 |
| ENSP00000261623 | CYBA | 3.15E-05 | 0.04 |
| ENSP00000357794 | TCHH | 3.15E-05 | 0.097 |
| ENSP00000367124 | SLC3A2 | 3.15E-05 | 0.125 |
| ENSP00000374455 | SQSTM1 | 3.15E-05 | 0.258 |
| ENSP00000354522 | TOP1 | 3.15E-05 | 0.377 |
| ENSP00000366267 | GPC5 | 3.15E-05 | 0.193 |
| ENSP00000396813 | PSMB9 | 3.15E-05 | 0.073 |
| ENSP00000247668 | TRAF2 | 3.15E-05 | 0.214 |
| ENSP00000269141 | CDH2 | 3.15E-05 | 0.36 |
| ENSP00000246949 | DNASE1 | 3.15E-05 | 0.034 |
| ENSP00000309103 | BAD | 3.14E-05 | 0.05 |
| ENSP00000295137 | ACTG2 | 3.14E-05 | 0.126 |
| ENSP00000341551 | SMAD4 | 3.14E-05 | 0.744 |
| ENSP00000296702 | TCERG1 | 3.14E-05 | 0.195 |
| ENSP00000306881 | SEC23A | 3.14E-05 | 0.131 |
| ENSP00000264009 | HSF4 | 3.14E-05 | 0.076 |
| ENSP00000316176 | UBE2N | 3.13E-05 | 0.324 |
| ENSP00000317985 | ROCK2 | 3.13E-05 | 0.135 |
| ENSP00000225402 | AATF | 3.13E-05 | 0.616 |
| ENSP00000233893 | HSPE1 | 3.13E-05 | 0.127 |
| ENSP00000362608 | PIM1 | 3.12E-05 | 0.014 |
| ENSP00000236938 | FCRLA | 3.12E-05 | 0.047 |
| ENSP00000345195 | UBQLN2 | 3.12E-05 | 0.161 |
| ENSP00000390497 | TRIM10 | 3.11E-05 | 0.029 |
| ENSP00000376024 | SNX9 | 3.11E-05 | 0.097 |
| ENSP00000315654 | KCNG2 | 3.11E-05 | 0.056 |
| ENSP00000345974 | ENOSF1 | 3.11E-05 | 0.06 |
| ENSP00000329507 | CD300C | 3.11E-05 | 0.026 |
| ENSP00000386187 | IFITM1 | 3.11E-05 | 0.085 |
| ENSP00000358795 | NEURL | 3.11E-05 | 0.046 |
| ENSP00000354451 | IQGAP3 | 3.11E-05 | 0.019 |
| ENSP00000248935 | GSTT1 | 3.11E-05 | 0.047 |
| ENSP00000297450 | ANGPT1 | 3.10E-05 | 0.07 |
| ENSP00000225992 | PPY | 3.10E-05 | 0.09 |
| ENSP00000215567 | TECR | 3.10E-05 | 0.205 |
| ENSP00000267079 | MAP3K12 | 3.09E-05 | 0.006 |
| ENSP00000306190 | SLAMF1 | 3.09E-05 | 0.036 |
| ENSP00000358815 | NEURL1B | 3.09E-05 | 0.015 |
| ENSP00000395598 | TRIM10 | 3.09E-05 | 0.032 |
| ENSP00000254066 | RARA | 3.09E-05 | 0.393 |
| ENSP00000292174 | CXCR5 | 3.09E-05 | 0.181 |
| ENSP00000296509 | MAD2L1 | 3.09E-05 | 0.901 |
| ENSP00000364649 | SDHB | 3.09E-05 | 0.191 |
| ENSP00000317272 | MET | 3.09E-05 | 0.312 |
| ENSP00000323929 | A2M | 3.08E-05 | 0.206 |
| ENSP00000362296 | POU3F4 | 3.08E-05 | 0.044 |
| ENSP00000293778 | CXCL16 | 3.08E-05 | 0.057 |
| ENSP00000353198 | PYY | 3.08E-05 | 0.074 |
| ENSP00000264613 | CP | 3.08E-05 | 0.068 |
| ENSP00000267169 | DIABLO | 3.08E-05 | 0.028 |
| ENSP00000412027 | PSMB9 | 3.08E-05 | 0.084 |
| ENSP00000401272 | GPANK1 | 3.08E-05 | 0.131 |
| ENSP00000245932 | VASP | 3.07E-05 | 0.182 |
| ENSP00000259895 | GTF2H4 | 3.07E-05 | 0.046 |
| ENSP00000352516 | DNMT1 | 3.07E-05 | 0.412 |
| ENSP00000300035 | KIAA0101 | 3.07E-05 | 0.16 |
| ENSP00000273951 | GC | 3.07E-05 | 0.036 |
| ENSP00000397073 | TRIM10 | 3.07E-05 | 0.012 |
| ENSP00000305603 | FUT3 | 3.07E-05 | 0.226 |
| ENSP00000216274 | RIPK3 | 3.07E-05 | 0.023 |
| ENSP00000278833 | ROM1 | 3.07E-05 | 0.206 |
| ENSP00000324124 | POLR2L | 3.07E-05 | 0.417 |
| ENSP00000396251 | GTF2H4 | 3.07E-05 | 0.046 |
| ENSP00000229307 | NANOG | 3.06E-05 | 0.467 |
| ENSP00000314425 | IL12RB1 | 3.06E-05 | 0.048 |
| ENSP00000289352 | HIST1H4H | 3.06E-05 | 0.444 |
| ENSP00000303315 | JUNB | 3.06E-05 | 0.182 |
| ENSP00000262839 | TRPC5 | 3.06E-05 | 0.155 |
| ENSP00000391879 | TRIM26 | 3.06E-05 | 0.032 |
| ENSP00000222553 | NAMPT | 3.06E-05 | 0.074 |
| ENSP00000415328 | TRIM26 | 3.06E-05 | 0.025 |
| ENSP00000352980 | HIST1H4A | 3.06E-05 | 0.414 |
| ENSP00000312326 | AOC3 | 3.06E-05 | 0.048 |
| ENSP00000043402 | RTN4R | 3.06E-05 | 0.088 |
| ENSP00000322957 | PAK7 | 3.06E-05 | 0.061 |
| ENSP00000316338 | BAIAP2 | 3.06E-05 | 0.093 |
| ENSP00000324302 | STXBP6 | 3.06E-05 | 0.025 |
| ENSP00000359552 | PKN2 | 3.05E-05 | 0.05 |
| ENSP00000367830 | PRKCZ | 3.05E-05 | 0.172 |
| ENSP00000228347 | POLR3B | 3.05E-05 | 0.243 |
| ENSP00000221859 | POLR2I | 3.05E-05 | 0.399 |
| ENSP00000279544 | KLRF1 | 3.05E-05 | 0.027 |
| ENSP00000354822 | XAF1 | 3.05E-05 | 0.133 |
| ENSP00000310216 | KLRC4 | 3.05E-05 | 0.024 |
| ENSP00000252945 | CYP2E1 | 3.04E-05 | 0.218 |
| ENSP00000365891 | WAS | 3.04E-05 | 0.322 |
| ENSP00000344549 | CARD14 | 3.04E-05 | 0.029 |
| ENSP00000363397 | UGCG | 3.04E-05 | 0.129 |
| ENSP00000302719 | KCNAB3 | 3.04E-05 | 0.096 |
| ENSP00000362014 | DNM1 | 3.04E-05 | 0.126 |
| ENSP00000297261 | SHH | 3.04E-05 | 0.865 |
| ENSP00000315070 | GJD4 | 3.04E-05 | 0.089 |
| ENSP00000366974 | HIST1H4F | 3.04E-05 | 0.419 |
| ENSP00000265113 | SLC1A3 | 3.03E-05 | 0.067 |
| ENSP00000276072 | TAF1 | 3.03E-05 | 0.247 |
| ENSP00000318585 | BACE1 | 3.03E-05 | 0.329 |
| ENSP00000320951 | DEFB103A | 3.03E-05 | 0.097 |
| ENSP00000370125 | HMGN1 | 3.03E-05 | 0.027 |
| ENSP00000278282 | SCGB1A1 | 3.03E-05 | 0.129 |
| ENSP00000262407 | ITGA2B | 3.02E-05 | 0.3 |
| ENSP00000338019 | DNAJB2 | 3.02E-05 | 0.03 |
| ENSP00000360248 | ENTPD1 | 3.02E-05 | 0.183 |
| ENSP00000363512 | ALOX5 | 3.02E-05 | 0.149 |
| ENSP00000381823 | SEC24A | 3.02E-05 | 0.114 |
| ENSP00000308452 | KRT17 | 3.02E-05 | 0.069 |
| ENSP00000369843 | EPHX2 | 3.01E-05 | 0.114 |
| ENSP00000278353 | HSD17B12 | 3.01E-05 | 0.085 |
| ENSP00000343204 | JAK1 | 3.01E-05 | 0.332 |
| ENSP00000392726 | POM121L2 | 3.01E-05 | 0.037 |
| ENSP00000357470 | IL6R | 3.01E-05 | 0.041 |
| ENSP00000405934 | ITPR1 | 3.01E-05 | 0.259 |
| ENSP00000320025 | CACNB2 | 3.00E-05 | 0.083 |
| ENSP00000259881 | PSORS1C1 | 3.00E-05 | 0.116 |
| ENSP00000262177 | DNAJB6 | 3.00E-05 | 0.033 |
| ENSP00000353624 | HIST1H4E | 3.00E-05 | 0.31 |
| ENSP00000262958 | GNA15 | 3.00E-05 | 0.224 |
| ENSP00000366581 | HIST1H4B | 3.00E-05 | 0.332 |
| ENSP00000350967 | ELP2 | 2.99E-05 | 0.094 |
| ENSP00000302486 | MAP2K1 | 2.99E-05 | 0.226 |
| ENSP00000347329 | SEC31A | 2.99E-05 | 0.18 |
| ENSP00000222002 | SULT2A1 | 2.99E-05 | 0.099 |
| ENSP00000272645 | POLR2D | 2.99E-05 | 0.293 |
| ENSP00000302896 | RPS9 | 2.99E-05 | 0.289 |
| ENSP00000358358 | GJA10 | 2.99E-05 | 0.028 |
| ENSP00000358153 | HIST2H4B | 2.99E-05 | 0.296 |
| ENSP00000280551 | SEC24D | 2.99E-05 | 0.108 |
| ENSP00000358162 | HIST2H4A | 2.99E-05 | 0.32 |
| ENSP00000347168 | HIST1H4J | 2.99E-05 | 0.294 |
| ENSP00000343282 | HIST1H4D | 2.99E-05 | 0.298 |
| ENSP00000346316 | HIST1H4I | 2.99E-05 | 0.3 |
| ENSP00000350159 | HIST1H4K | 2.98E-05 | 0.298 |
| ENSP00000360025 | GADD45A | 2.98E-05 | 0.165 |
| ENSP00000406288 | CHD8 | 2.98E-05 | 0.085 |
| ENSP00000337773 | NQO2 | 2.98E-05 | 0.031 |
| ENSP00000268876 | UNC45B | 2.98E-05 | 0.042 |
| ENSP00000335084 | PPP1CC | 2.98E-05 | 0.758 |
| ENSP00000321845 | SEC24C | 2.98E-05 | 0.135 |
| ENSP00000372126 | DEFA1 | 2.98E-05 | 0.114 |
| ENSP00000207870 | XYLB | 2.98E-05 | 0.021 |
| ENSP00000318884 | FMN2 | 2.97E-05 | 0.079 |
| ENSP00000255266 | PDE6A | 2.97E-05 | 0.101 |
| ENSP00000359532 | NTSR1 | 2.96E-05 | 0.42 |
| ENSP00000363115 | FGR | 2.96E-05 | 0.36 |
| ENSP00000305873 | CRTC2 | 2.96E-05 | 0.031 |
| ENSP00000206544 | SLC22A17 | 2.96E-05 | 0.014 |
| ENSP00000314030 | DNAJA2 | 2.96E-05 | 0.071 |
| ENSP00000259915 | POU5F1 | 2.96E-05 | 0.157 |
| ENSP00000371376 | TLR6 | 2.95E-05 | 0.02 |
| ENSP00000401980 | MAVS | 2.95E-05 | 0.07 |
| ENSP00000264265 | LXN | 2.95E-05 | 0.164 |
| ENSP00000262746 | PRDX1 | 2.95E-05 | 0.213 |
| ENSP00000007516 | NDUFAB1 | 2.95E-05 | 0.618 |
| ENSP00000246115 | S1PR4 | 2.95E-05 | 0.261 |
| ENSP00000263119 | CABIN1 | 2.95E-05 | 0.048 |
| ENSP00000200307 | CCL7 | 2.94E-05 | 0.025 |
| ENSP00000254722 | SERPINF1 | 2.94E-05 | 0.068 |
| ENSP00000372817 | STK19 | 2.94E-05 | 0.094 |
| ENSP00000292476 | CPSF4 | 2.93E-05 | 0.17 |
| ENSP00000369050 | CYP1A1 | 2.93E-05 | 0.28 |
| ENSP00000351170 | GDA | 2.93E-05 | 0.035 |
| ENSP00000268389 | IGSF6 | 2.93E-05 | 0.233 |
| ENSP00000317674 | APOL1 | 2.93E-05 | 0.038 |
| ENSP00000302569 | CLEC7A | 2.93E-05 | 0.065 |
| ENSP00000369129 | DSP | 2.93E-05 | 0.1 |
| ENSP00000279242 | MRPL49 | 2.93E-05 | 0.325 |
| ENSP00000371554 | SOD3 | 2.93E-05 | 0.055 |
| ENSP00000355512 | OPN3 | 2.92E-05 | 0.171 |
| ENSP00000369858 | ALOX5AP | 2.92E-05 | 0.127 |
| ENSP00000369820 | PIGA | 2.92E-05 | 0.137 |
| ENSP00000361433 | EXOSC2 | 2.92E-05 | 0.132 |
| ENSP00000219235 | CCL22 | 2.92E-05 | 0.058 |
| ENSP00000310998 | NAGPA | 2.92E-05 | 0.205 |
| ENSP00000420295 | PDE6B | 2.91E-05 | 0.135 |
| ENSP00000278568 | PAK1 | 2.91E-05 | 0.266 |
| ENSP00000251973 | CARD10 | 2.91E-05 | 0.036 |
| ENSP00000310928 | PPARD | 2.91E-05 | 0.13 |
| ENSP00000333268 | CSH1 | 2.91E-05 | 0.068 |
| ENSP00000282541 | GPD1L | 2.91E-05 | 0.133 |
| ENSP00000236826 | MMP8 | 2.91E-05 | 0.048 |
| ENSP00000419851 | GMPS | 2.91E-05 | 0.652 |
| ENSP00000195649 | SNAP91 | 2.91E-05 | 0.072 |
| ENSP00000393744 | PSMB9 | 2.91E-05 | 0.08 |
| ENSP00000354476 | SREBF2 | 2.91E-05 | 0.17 |
| ENSP00000356290 | MTHFD1L | 2.90E-05 | 0.111 |
| ENSP00000385721 | GGT2 | 2.90E-05 | 0.12 |
| ENSP00000363993 | PSMB9 | 2.90E-05 | 0.082 |
| ENSP00000359490 | GBP4 | 2.89E-05 | 0.132 |
| ENSP00000249647 | SNAP23 | 2.89E-05 | 0.152 |
| ENSP00000361562 | CTSA | 2.89E-05 | 0.15 |
| ENSP00000391798 | STK19 | 2.89E-05 | 0.107 |
| ENSP00000357153 | CD1D | 2.88E-05 | 0.076 |
| ENSP00000357038 | SLAMF6 | 2.88E-05 | 0.075 |
| ENSP00000293860 | POLR3K | 2.88E-05 | 0.134 |
| ENSP00000307863 | U2AF2 | 2.88E-05 | 0.287 |
| ENSP00000417257 | FNDC3A | 2.87E-05 | 0.329 |
| ENSP00000352011 | CACNA1G | 2.87E-05 | 0.122 |
| ENSP00000327943 | SLC5A2 | 2.87E-05 | 0.055 |
| ENSP00000420588 | TFAM | 2.87E-05 | 0.125 |
| ENSP00000350914 | BDH1 | 2.86E-05 | 0.071 |
| ENSP00000355133 | NOS1AP | 2.86E-05 | 0.042 |
| ENSP00000345656 | VAPA | 2.86E-05 | 0.126 |
| ENSP00000371236 | GART | 2.86E-05 | 0.419 |
| ENSP00000254436 | TRIM21 | 2.86E-05 | 0.119 |
| ENSP00000357058 | CASQ1 | 2.86E-05 | 0.052 |
| ENSP00000261826 | P2RX7 | 2.86E-05 | 0.054 |
| ENSP00000295962 | ABHD6 | 2.86E-05 | 0.163 |
| ENSP00000345206 | RBPJ | 2.85E-05 | 0.354 |
| ENSP00000401802 | PSMC6 | 2.85E-05 | 0.836 |
| ENSP00000321810 | TRIT1 | 2.85E-05 | 0.11 |
| ENSP00000376076 | SUMO1 | 2.85E-05 | 0.789 |
| ENSP00000417587 | PEG10 | 2.84E-05 | 0.086 |
| ENSP00000263857 | POLR1A | 2.84E-05 | 0.268 |
| ENSP00000384708 | FSHR | 2.84E-05 | 0.168 |
| ENSP00000296511 | ANXA5 | 2.84E-05 | 0.051 |
| ENSP00000413572 | NLE1 | 2.84E-05 | 0.37 |
| ENSP00000339804 | TSLP | 2.84E-05 | 0.053 |
| ENSP00000361446 | POLR3A | 2.84E-05 | 0.217 |
| ENSP00000353500 | CLEC4C | 2.84E-05 | 0.012 |
| ENSP00000234091 | ID2 | 2.84E-05 | 0.183 |
| ENSP00000245441 | NIN | 2.84E-05 | 0.32 |
| ENSP00000222305 | USF2 | 2.84E-05 | 0.027 |
| ENSP00000267163 | RB1 | 2.83E-05 | 0.718 |
| ENSP00000216027 | HSCB | 2.83E-05 | 0.039 |
| ENSP00000358126 | VPS45 | 2.83E-05 | 0.089 |
| ENSP00000333656 | SIGIRR | 2.83E-05 | 0.013 |
| ENSP00000219244 | CCL17 | 2.82E-05 | 0.067 |
| ENSP00000367029 | POLR1E | 2.82E-05 | 0.228 |
| ENSP00000340820 | MAPT | 2.82E-05 | 0.406 |
| ENSP00000239849 | TNFSF11 | 2.82E-05 | 0.082 |
| ENSP00000194530 | STRADB | 2.82E-05 | 0.071 |
| ENSP00000356505 | NCF2 | 2.82E-05 | 0.194 |
| ENSP00000307900 | GLUL | 2.81E-05 | 0.189 |
| ENSP00000240304 | LUC7L3 | 2.81E-05 | 0.227 |
| ENSP00000321259 | TALDO1 | 2.81E-05 | 0.156 |
| ENSP00000054668 | UTS2 | 2.81E-05 | 0.177 |
| ENSP00000260302 | MMP13 | 2.81E-05 | 0.08 |
| ENSP00000302517 | HLA-DRB3 | 2.81E-05 | 0.031 |
| ENSP00000305824 | KCNS3 | 2.81E-05 | 0.104 |
| ENSP00000349595 | ATP2A1 | 2.81E-05 | 0.174 |
| ENSP00000376623 | CSH2 | 2.80E-05 | 0.07 |
| ENSP00000222115 | HAS1 | 2.80E-05 | 0.126 |
| ENSP00000263182 | BBOX1 | 2.80E-05 | 0.383 |
| ENSP00000410512 | HLA-DQB2 | 2.80E-05 | 0.04 |
| ENSP00000215904 | PDXP | 2.80E-05 | 0.195 |
| ENSP00000415183 | MUC2 | 2.80E-05 | 0.29 |
| ENSP00000391938 | HLA-DQB2 | 2.80E-05 | 0.041 |
| ENSP00000278927 | ESAM | 2.80E-05 | 0.126 |
| ENSP00000311405 | ADCY6 | 2.80E-05 | 0.352 |
| ENSP00000353820 | CYP2D6 | 2.80E-05 | 0.117 |
| ENSP00000314420 | PER1 | 2.79E-05 | 0.13 |
| ENSP00000260985 | IDH1 | 2.79E-05 | 0.136 |
| ENSP00000339992 | MYB | 2.79E-05 | 0.551 |
| ENSP00000393887 | AHSG | 2.79E-05 | 0.374 |
| ENSP00000340330 | KAT5 | 2.79E-05 | 0.308 |
| ENSP00000243077 | LRP1 | 2.79E-05 | 0.062 |
| ENSP00000227266 | CTSC | 2.79E-05 | 0.091 |
| ENSP00000413254 | RPH3A | 2.79E-05 | 0.079 |
| ENSP00000364094 | ITGB1 | 2.79E-05 | 0.48 |
| ENSP00000249364 | CALU | 2.78E-05 | 0.059 |
| ENSP00000332565 | SULT4A1 | 2.78E-05 | 0.214 |
| ENSP00000351789 | PELI1 | 2.78E-05 | 0.085 |
| ENSP00000240662 | KCNJ8 | 2.78E-05 | 0.023 |
| ENSP00000246041 | AP5S1 | 2.78E-05 | 0.066 |
| ENSP00000266718 | LUM | 2.77E-05 | 0.287 |
| ENSP00000329243 | KRT7 | 2.77E-05 | 0.101 |
| ENSP00000338260 | APOL4 | 2.77E-05 | 0.025 |
| ENSP00000355747 | PSEN2 | 2.77E-05 | 0.169 |
| ENSP00000339801 | IDS | 2.77E-05 | 0.409 |
| ENSP00000358966 | IMPG1 | 2.76E-05 | 0.028 |
| ENSP00000384026 | HMGA2 | 2.76E-05 | 0.182 |
| ENSP00000324173 | HSPA5 | 2.76E-05 | 0.383 |
| ENSP00000243786 | INHA | 2.76E-05 | 0.053 |
| ENSP00000390948 | CYFIP2 | 2.76E-05 | 0.111 |
| ENSP00000370445 | HTN3 | 2.76E-05 | 0.206 |
| ENSP00000218176 | KCND1 | 2.76E-05 | 0.106 |
| ENSP00000261207 | PPP1R12A | 2.76E-05 | 0.544 |
| ENSP00000252818 | JUND | 2.76E-05 | 0.175 |
| ENSP00000320509 | BANK1 | 2.75E-05 | 0.088 |
| ENSP00000264162 | LCT | 2.75E-05 | 0.304 |
| ENSP00000317145 | GDNF | 2.75E-05 | 0.271 |
| ENSP00000258080 | HTRA2 | 2.75E-05 | 0.121 |
| ENSP00000293218 | UNK | 2.75E-05 | 0.004 |
| ENSP00000287727 | ZFYVE9 | 2.75E-05 | 0.243 |
| ENSP00000261833 | CIT | 2.75E-05 | 0.034 |
| ENSP00000253023 | UBE2M | 2.75E-05 | 0.141 |
| ENSP00000237380 | MED28 | 2.75E-05 | 0.035 |
| ENSP00000312262 | ADRBK1 | 2.75E-05 | 0.266 |
| ENSP00000328023 | SRPR | 2.75E-05 | 0.117 |
| ENSP00000249066 | APOL2 | 2.74E-05 | 0.046 |
| ENSP00000242480 | EGR2 | 2.74E-05 | 0.235 |
| ENSP00000369497 | BRCA2 | 2.74E-05 | 0.288 |
| ENSP00000337761 | RAB27A | 2.74E-05 | 0.36 |
| ENSP00000321203 | TNIP2 | 2.74E-05 | 0.023 |
| ENSP00000327070 | MDH2 | 2.74E-05 | 0.484 |
| ENSP00000356430 | RGS18 | 2.74E-05 | 0.26 |
| ENSP00000331103 | IP6K2 | 2.74E-05 | 0.05 |
| ENSP00000305071 | RFXANK | 2.74E-05 | 0.107 |
| ENSP00000263593 | SIAE | 2.74E-05 | 0.199 |
| ENSP00000225519 | SHPK | 2.74E-05 | 0.036 |
| ENSP00000312027 | CD7 | 2.73E-05 | 0.079 |
| ENSP00000304592 | FASN | 2.73E-05 | 0.314 |
| ENSP00000400104 | RXRB | 2.73E-05 | 0.048 |
| ENSP00000262461 | SLC12A2 | 2.73E-05 | 0.084 |
| ENSP00000368253 | NR0B1 | 2.73E-05 | 0.204 |
| ENSP00000263645 | CD81 | 2.73E-05 | 0.124 |
| ENSP00000053867 | GRN | 2.73E-05 | 0.124 |
| ENSP00000046794 | LCP2 | 2.73E-05 | 0.286 |
| ENSP00000364893 | ARHGEF7 | 2.72E-05 | 0.091 |
| ENSP00000312435 | DAG1 | 2.72E-05 | 0.306 |
| ENSP00000274606 | NHP2 | 2.72E-05 | 0.878 |
| ENSP00000209540 | OR1I1 | 2.72E-05 | 0.114 |
| ENSP00000260818 | DNAJC13 | 2.72E-05 | 0.04 |
| ENSP00000377372 | GAP43 | 2.72E-05 | 0.067 |
| ENSP00000277549 | CACNA1B | 2.72E-05 | 0.122 |
| ENSP00000374791 | IGKV6-21 | 2.72E-05 | 0.007 |
| ENSP00000240488 | MND1 | 2.71E-05 | 0.31 |
| ENSP00000399155 | TUBB | 2.71E-05 | 0.093 |
| ENSP00000232014 | BCL6 | 2.71E-05 | 0.34 |
| ENSP00000347244 | ITSN2 | 2.71E-05 | 0.175 |
| ENSP00000256119 | CA1 | 2.71E-05 | 0.026 |
| ENSP00000391685 | TRIM31 | 2.71E-05 | 0.119 |
| ENSP00000388322 | HHLA1 | 2.71E-05 | 0.058 |
| ENSP00000220959 | UBR5 | 2.71E-05 | 0.407 |
| ENSP00000385057 | APOBEC3G | 2.71E-05 | 0.112 |
| ENSP00000319377 | NLRP12 | 2.71E-05 | 0.074 |
| ENSP00000372991 | LTA | 2.71E-05 | 0.102 |
| ENSP00000369519 | MTAP | 2.70E-05 | 0.167 |
| ENSP00000262027 | MARS | 2.70E-05 | 0.212 |
| ENSP00000368401 | PAX6 | 2.70E-05 | 0.335 |
| ENSP00000339292 | CLDN14 | 2.70E-05 | 0.092 |
| ENSP00000272227 | PDIA6 | 2.70E-05 | 0.145 |
| ENSP00000377047 | PTPRZ1 | 2.70E-05 | 0.052 |
| ENSP00000205890 | MYO15A | 2.69E-05 | 0.067 |
| ENSP00000378132 | IRX5 | 2.69E-05 | 0.066 |
| ENSP00000398677 | TRIM31 | 2.69E-05 | 0.118 |
| ENSP00000248070 | EPS15L1 | 2.69E-05 | 0.048 |
| ENSP00000356785 | NME7 | 2.69E-05 | 0.138 |
| ENSP00000377783 | PROS1 | 2.69E-05 | 0.051 |
| ENSP00000231790 | MLH1 | 2.69E-05 | 0.339 |
| ENSP00000339527 | FOXO3 | 2.69E-05 | 0.064 |
| ENSP00000371234 | CYS1 | 2.69E-05 | 0.014 |
| ENSP00000164227 | BCL3 | 2.69E-05 | 0.106 |
| ENSP00000248598 | FGL2 | 2.68E-05 | 0.163 |
| ENSP00000304414 | CXCR6 | 2.68E-05 | 0.162 |
| ENSP00000336790 | ATF4 | 2.68E-05 | 0.319 |
| ENSP00000312002 | HAVCR2 | 2.68E-05 | 0.056 |
| ENSP00000242057 | AHR | 2.67E-05 | 0.144 |
| ENSP00000327255 | PPM1A | 2.67E-05 | 0.215 |
| ENSP00000366013 | GNB2L1 | 2.67E-05 | 0.621 |
| ENSP00000342513 | IFI6 | 2.66E-05 | 0.071 |
| ENSP00000355731 | CDC42BPA | 2.66E-05 | 0.065 |
| ENSP00000397351 | GRID2IP | 2.66E-05 | 0.029 |
| ENSP00000307272 | RPTOR | 2.66E-05 | 0.142 |
| ENSP00000360560 | TCTE1 | 2.66E-05 | 0.034 |
| ENSP00000265077 | VCAN | 2.66E-05 | 0.278 |
| ENSP00000262238 | YY1 | 2.66E-05 | 0.495 |
| ENSP00000353793 | WDHD1 | 2.65E-05 | 0.458 |
| ENSP00000271588 | HMCN1 | 2.65E-05 | 0.08 |
| ENSP00000264552 | UBE2S | 2.65E-05 | 0.379 |
| ENSP00000360215 | ZBP1 | 2.65E-05 | 0.046 |
| ENSP00000354575 | LSR | 2.65E-05 | 0.027 |
| ENSP00000297323 | ADCY1 | 2.65E-05 | 0.398 |
| ENSP00000410294 | FGFR2 | 2.65E-05 | 0.458 |
| ENSP00000258091 | CCT7 | 2.65E-05 | 0.599 |
| ENSP00000245539 | MRPS7 | 2.65E-05 | 0.41 |
| ENSP00000327890 | IL3RA | 2.65E-05 | 0.028 |
| ENSP00000009180 | CD9 | 2.65E-05 | 0.13 |
| ENSP00000316786 | HSD11B2 | 2.64E-05 | 0.056 |
| ENSP00000258418 | CAB39 | 2.64E-05 | 0.391 |
| ENSP00000316460 | FYB | 2.64E-05 | 0.128 |
| ENSP00000299421 | ILK | 2.64E-05 | 0.261 |
| ENSP00000358814 | AHCYL1 | 2.63E-05 | 0.069 |
| ENSP00000299333 | SCN3B | 2.63E-05 | 0.062 |
| ENSP00000350198 | SSTR2 | 2.63E-05 | 0.081 |
| ENSP00000300055 | PLIN1 | 2.63E-05 | 0.21 |
| ENSP00000265026 | MAP3K13 | 2.63E-05 | 0.015 |
| ENSP00000381031 | EWSR1 | 2.63E-05 | 0.509 |
| ENSP00000231454 | IL5 | 2.63E-05 | 0.074 |
| ENSP00000342889 | POLR2K | 2.62E-05 | 0.092 |
| ENSP00000264005 | LCAT | 2.62E-05 | 0.133 |
| ENSP00000263056 | MAP3K8 | 2.62E-05 | 0.072 |
| ENSP00000267890 | TTBK2 | 2.62E-05 | 0.035 |
| ENSP00000339960 | KCNJ5 | 2.62E-05 | 0.058 |
| ENSP00000345571 | E2F1 | 2.62E-05 | 0.262 |
| ENSP00000265310 | TRPV5 | 2.62E-05 | 0.083 |
| ENSP00000240100 | DUSP4 | 2.62E-05 | 0.076 |
| ENSP00000244711 | MEA1 | 2.61E-05 | 0.175 |
| ENSP00000216392 | PYGL | 2.61E-05 | 0.373 |
| ENSP00000406229 | FAT1 | 2.61E-05 | 0.064 |
| ENSP00000302216 | ATOH1 | 2.61E-05 | 0.099 |
| ENSP00000252593 | BST2 | 2.61E-05 | 0.111 |
| ENSP00000354568 | BRDT | 2.61E-05 | 0.081 |
| ENSP00000263640 | ACVR1 | 2.61E-05 | 0.153 |
| ENSP00000315931 | AHCYL2 | 2.61E-05 | 0.046 |
| ENSP00000224600 | RBP3 | 2.61E-05 | 0.059 |
| ENSP00000392270 | STRAP | 2.61E-05 | 0.058 |
| ENSP00000361926 | CNPY3 | 2.61E-05 | 0.029 |
| ENSP00000336655 | STRADA | 2.61E-05 | 0.056 |
| ENSP00000362299 | ENG | 2.61E-05 | 0.148 |
| ENSP00000334267 | C5orf38 | 2.61E-05 | 0.079 |
| ENSP00000305244 | IRX1 | 2.60E-05 | 0.061 |
| ENSP00000369703 | TUBB2A | 2.60E-05 | 0.172 |
| ENSP00000347507 | MYH7 | 2.60E-05 | 0.208 |
| ENSP00000342560 | HRH3 | 2.60E-05 | 0.088 |
| ENSP00000355560 | TBCE | 2.60E-05 | 0.164 |
| ENSP00000340409 | SMPD1 | 2.60E-05 | 0.157 |
| ENSP00000328207 | TNFRSF18 | 2.60E-05 | 0.028 |
| ENSP00000356433 | UST | 2.60E-05 | 0.275 |
| ENSP00000411532 | TOP2A | 2.60E-05 | 0.71 |
| ENSP00000263201 | CDC45 | 2.60E-05 | 0.665 |
| ENSP00000311449 | RAB6A | 2.60E-05 | 0.235 |
| ENSP00000302830 | MAEA | 2.60E-05 | 0.194 |
| ENSP00000296882 | GJB7 | 2.59E-05 | 0.036 |
| ENSP00000301825 | ENTPD3 | 2.59E-05 | 0.136 |
| ENSP00000262865 | BPI | 2.59E-05 | 0.077 |
| ENSP00000366828 | POLR3F | 2.59E-05 | 0.149 |
| ENSP00000377083 | ALDH1L1 | 2.59E-05 | 0.099 |
| ENSP00000216214 | FAM118A | 2.59E-05 | 0.044 |
| ENSP00000360561 | ENTPD8 | 2.59E-05 | 0.136 |
| ENSP00000343782 | ADRB3 | 2.59E-05 | 0.122 |
| ENSP00000280772 | ANK3 | 2.59E-05 | 0.11 |
| ENSP00000382595 | PAICS | 2.59E-05 | 0.814 |
| ENSP00000339867 | CLCN1 | 2.59E-05 | 0.044 |
| ENSP00000176183 | DRD4 | 2.58E-05 | 0.246 |
| ENSP00000264938 | SLC9A3 | 2.58E-05 | 0.112 |
| ENSP00000250495 | NEDD8 | 2.58E-05 | 0.298 |
| ENSP00000300108 | TAC3 | 2.58E-05 | 0.086 |
| ENSP00000358335 | MAP3K7 | 2.57E-05 | 0.378 |
| ENSP00000335281 | DEFB105B | 2.57E-05 | 0.069 |
| ENSP00000363869 | VSIG4 | 2.57E-05 | 0.174 |
| ENSP00000347836 | UBE2H | 2.57E-05 | 0.428 |
| ENSP00000355370 | CNTF | 2.57E-05 | 0.175 |
| ENSP00000381891 | ERG | 2.57E-05 | 0.117 |
| ENSP00000234961 | OPRD1 | 2.57E-05 | 0.159 |
| ENSP00000393007 | TCF19 | 2.56E-05 | 0.06 |
| ENSP00000321427 | KCNH5 | 2.56E-05 | 0.174 |
| ENSP00000358310 | ITGA10 | 2.55E-05 | 0.197 |
| ENSP00000297469 | GPER | 2.55E-05 | 0.059 |
| ENSP00000405229 | TRIM27 | 2.55E-05 | 0.101 |
| ENSP00000402065 | POLR2J2 | 2.55E-05 | 0.2 |
| ENSP00000370912 | TEC | 2.55E-05 | 0.128 |
| ENSP00000358674 | UBL4A | 2.55E-05 | 0.094 |
| ENSP00000379933 | TPI1 | 2.55E-05 | 0.335 |
| ENSP00000362824 | OGT | 2.55E-05 | 0.351 |
| ENSP00000364794 | IARS | 2.54E-05 | 0.607 |
| ENSP00000304467 | THOP1 | 2.54E-05 | 0.095 |
| ENSP00000335074 | GHRL | 2.54E-05 | 0.076 |
| ENSP00000367787 | LIG3 | 2.54E-05 | 0.146 |
| ENSP00000379612 | TAX1BP1 | 2.54E-05 | 0.083 |
| ENSP00000328359 | DEFA3 | 2.54E-05 | 0.122 |
| ENSP00000381590 | ATXN7 | 2.54E-05 | 0.089 |
| ENSP00000341680 | DTNBP1 | 2.54E-05 | 0.209 |
| ENSP00000303088 | POLR3D | 2.54E-05 | 0.148 |
| ENSP00000358867 | GNAI3 | 2.54E-05 | 0.548 |
| ENSP00000301905 | PBK | 2.53E-05 | 0.527 |
| ENSP00000357292 | UBQLN4 | 2.53E-05 | 0.371 |
| ENSP00000293897 | SSTR5 | 2.53E-05 | 0.1 |
| ENSP00000368645 | POLR2J3 | 2.53E-05 | 0.171 |
| ENSP00000395473 | MAN2B1 | 2.53E-05 | 0.133 |
| ENSP00000294954 | LHCGR | 2.53E-05 | 0.166 |
| ENSP00000409346 | CISH | 2.53E-05 | 0.09 |
| ENSP00000348762 | LSS | 2.53E-05 | 0.376 |
| ENSP00000264723 | CSPG5 | 2.53E-05 | 0.203 |
| ENSP00000305107 | GIMAP8 | 2.52E-05 | 0.099 |
| ENSP00000387040 | FAT3 | 2.52E-05 | 0.066 |
| ENSP00000009589 | RPS20 | 2.52E-05 | 0.493 |
| ENSP00000319060 | CAMK2G | 2.52E-05 | 0.248 |
| ENSP00000362282 | MOCS1 | 2.52E-05 | 0.097 |
| ENSP00000371514 | KCNV2 | 2.51E-05 | 0.205 |
| ENSP00000410635 | C6orf100 | 2.51E-05 | 0.149 |
| ENSP00000407879 | MYH14 | 2.51E-05 | 0.043 |
| ENSP00000338629 | RERE | 2.51E-05 | 0.238 |
| ENSP00000218099 | F9 | 2.51E-05 | 0.131 |
| ENSP00000386259 | NEB | 2.51E-05 | 0.205 |
| ENSP00000369816 | SHBG | 2.51E-05 | 0.235 |
| ENSP00000368169 | DVL1 | 2.50E-05 | 0.441 |
| ENSP00000364271 | DFNB59 | 2.50E-05 | 0.083 |
| ENSP00000257857 | CD63 | 2.50E-05 | 0.21 |
| ENSP00000374372 | SPTB | 2.50E-05 | 0.074 |
| ENSP00000320376 | P2RY13 | 2.50E-05 | 0.098 |
| ENSP00000262101 | MSR1 | 2.50E-05 | 0.099 |
| ENSP00000394134 | C6orf100 | 2.50E-05 | 0.141 |
| ENSP00000396675 | C6orf100 | 2.50E-05 | 0.142 |
| ENSP00000244007 | PLCG1 | 2.50E-05 | 0.586 |
| ENSP00000362570 | FNDC5 | 2.50E-05 | 0.11 |
| ENSP00000267549 | GPR65 | 2.50E-05 | 0.352 |
| ENSP00000297404 | KCNV1 | 2.50E-05 | 0.202 |
| ENSP00000345575 | DNAJB12 | 2.49E-05 | 0.115 |
| ENSP00000309595 | C10orf2 | 2.49E-05 | 0.119 |
| ENSP00000290219 | IFNGR2 | 2.49E-05 | 0.071 |
| ENSP00000354961 | MT-ND4 | 2.49E-05 | 0.104 |
| ENSP00000308208 | MMP14 | 2.49E-05 | 0.129 |
| ENSP00000260008 | FHDC1 | 2.49E-05 | 0.064 |
| ENSP00000327290 | ITGA11 | 2.49E-05 | 0.146 |
| ENSP00000259631 | CCL27 | 2.49E-05 | 0.12 |
| ENSP00000296029 | PF4 | 2.49E-05 | 0.092 |
| ENSP00000303452 | TRH | 2.49E-05 | 0.305 |
| ENSP00000307432 | SUCLG2 | 2.49E-05 | 0.166 |
| ENSP00000302051 | ECEL1 | 2.48E-05 | 0.117 |
| ENSP00000331574 | PDE1A | 2.48E-05 | 0.096 |
| ENSP00000369677 | EMILIN1 | 2.48E-05 | 0.274 |
| ENSP00000295082 | KCNF1 | 2.48E-05 | 0.146 |
| ENSP00000240922 | NAA50 | 2.48E-05 | 0.677 |
| ENSP00000348545 | CGB7 | 2.48E-05 | 0.017 |
| ENSP00000306099 | FGB | 2.48E-05 | 0.143 |
| ENSP00000244137 | PEPD | 2.48E-05 | 0.141 |
| ENSP00000173898 | TRO | 2.48E-05 | 0.219 |
| ENSP00000252505 | ALLC | 2.48E-05 | 0.092 |
| ENSP00000235329 | MFN2 | 2.48E-05 | 0.124 |
| ENSP00000217961 | STS | 2.48E-05 | 0.576 |
| ENSP00000307694 | KCNS1 | 2.47E-05 | 0.185 |
| ENSP00000362690 | NR5A1 | 2.47E-05 | 0.299 |
| ENSP00000257264 | TCN1 | 2.47E-05 | 0.057 |
| ENSP00000274255 | SKP2 | 2.47E-05 | 0.52 |
| ENSP00000169298 | ST6GAL1 | 2.47E-05 | 0.083 |
| ENSP00000242208 | INHBA | 2.47E-05 | 0.093 |
| ENSP00000340211 | CORO1B | 2.47E-05 | 0.102 |
| ENSP00000359290 | DR1 | 2.47E-05 | 0.166 |
| ENSP00000355237 | CDC42BPB | 2.46E-05 | 0.071 |
| ENSP00000305355 | PRKCB | 2.46E-05 | 0.313 |
| ENSP00000354813 | MT-ND5 | 2.46E-05 | 0.109 |
| ENSP00000243253 | SEC61A1 | 2.46E-05 | 0.436 |
| ENSP00000353824 | PIM3 | 2.46E-05 | 0.024 |
| ENSP00000363689 | ID3 | 2.46E-05 | 0.09 |
| ENSP00000304127 | KCNG3 | 2.46E-05 | 0.145 |
| ENSP00000377617 | MTHFD2 | 2.46E-05 | 0.183 |
| ENSP00000351957 | PDE3A | 2.46E-05 | 0.059 |
| ENSP00000355941 | TATDN3 | 2.46E-05 | 0.048 |
| ENSP00000287936 | HMGCR | 2.45E-05 | 0.266 |
| ENSP00000347596 | EFEMP1 | 2.45E-05 | 0.052 |
| ENSP00000264661 | KCNH4 | 2.45E-05 | 0.129 |
| ENSP00000240139 | PPP3CC | 2.45E-05 | 0.145 |
| ENSP00000358730 | ADORA3 | 2.45E-05 | 0.083 |
| ENSP00000356999 | USF1 | 2.45E-05 | 0.11 |
| ENSP00000254122 | FSHB | 2.45E-05 | 0.09 |
| ENSP00000405798 | CDK16 | 2.44E-05 | 0.211 |
| ENSP00000278916 | CHEK1 | 2.44E-05 | 0.851 |
| ENSP00000312129 | KCNG4 | 2.44E-05 | 0.115 |
| ENSP00000255945 | GIMAP4 | 2.44E-05 | 0.15 |
| ENSP00000370543 | SLC5A3 | 2.44E-05 | 0.032 |
| ENSP00000287042 | KCNS2 | 2.44E-05 | 0.124 |
| ENSP00000248444 | VIL1 | 2.44E-05 | 0.143 |
| ENSP00000085219 | CD22 | 2.44E-05 | 0.185 |
| ENSP00000384554 | CLDN5 | 2.44E-05 | 0.31 |
| ENSP00000245919 | FOSB | 2.44E-05 | 0.166 |
| ENSP00000274496 | YIPF5 | 2.44E-05 | 0.168 |
| ENSP00000295624 | NAV1 | 2.44E-05 | 0.075 |
| ENSP00000344115 | CDH5 | 2.43E-05 | 0.258 |
| ENSP00000297350 | TNFRSF11B | 2.43E-05 | 0.064 |
| ENSP00000347345 | POLR3H | 2.43E-05 | 0.187 |
| ENSP00000222005 | CDC37 | 2.43E-05 | 0.124 |
| ENSP00000372853 | C2 | 2.43E-05 | 0.061 |
| ENSP00000292896 | HBE1 | 2.43E-05 | 0.179 |
| ENSP00000307940 | EEF2 | 2.43E-05 | 0.545 |
| ENSP00000247470 | PYCARD | 2.43E-05 | 0.164 |
| ENSP00000332151 | DSE | 2.42E-05 | 0.241 |
| ENSP00000363330 | CD52 | 2.42E-05 | 0.141 |
| ENSP00000299663 | CLEC4E | 2.42E-05 | 0.084 |
| ENSP00000357066 | ARG1 | 2.42E-05 | 0.118 |
| ENSP00000227251 | CRYAB | 2.42E-05 | 0.193 |
| ENSP00000359976 | CTH | 2.42E-05 | 0.101 |
| ENSP00000267377 | SSTR1 | 2.42E-05 | 0.078 |
| ENSP00000248553 | HSPB1 | 2.42E-05 | 0.25 |
| ENSP00000391676 | JAK3 | 2.42E-05 | 0.266 |
| ENSP00000356791 | DPT | 2.42E-05 | 0.08 |
| ENSP00000286355 | ADCY8 | 2.42E-05 | 0.432 |
| ENSP00000300060 | ANPEP | 2.42E-05 | 0.167 |
| ENSP00000251496 | NCAPG | 2.41E-05 | 0.542 |
| ENSP00000376410 | INF2 | 2.41E-05 | 0.025 |
| ENSP00000361548 | MPL | 2.41E-05 | 0.198 |
| ENSP00000381876 | DAAM2 | 2.41E-05 | 0.057 |
| ENSP00000345676 | 1-Mar | 2.41E-05 | 0.076 |
| ENSP00000222812 | STX1A | 2.41E-05 | 0.436 |
| ENSP00000269576 | KRT10 | 2.41E-05 | 0.049 |
| ENSP00000358456 | F8A1 | 2.41E-05 | 0.06 |
| ENSP00000252674 | MLLT1 | 2.41E-05 | 0.401 |
| ENSP00000265191 | NME5 | 2.40E-05 | 0.207 |
| ENSP00000306968 | CDCA7 | 2.40E-05 | 0.309 |
| ENSP00000358903 | CYP17A1 | 2.40E-05 | 0.172 |
| ENSP00000215909 | LGALS1 | 2.40E-05 | 0.171 |
| ENSP00000286332 | TAB2 | 2.40E-05 | 0.135 |
| ENSP00000276449 | STAR | 2.40E-05 | 0.119 |
| ENSP00000266481 | DNM1L | 2.40E-05 | 0.435 |
| ENSP00000226207 | MYH1 | 2.40E-05 | 0.209 |
| ENSP00000270349 | SLC6A3 | 2.40E-05 | 0.489 |
| ENSP00000301396 | PELP1 | 2.40E-05 | 0.061 |
| ENSP00000334051 | GNAL | 2.40E-05 | 0.638 |
| ENSP00000318297 | RUVBL1 | 2.40E-05 | 0.664 |
| ENSP00000259254 | GYPC | 2.40E-05 | 0.134 |
| ENSP00000233813 | IGFBP5 | 2.39E-05 | 0.101 |
| ENSP00000342626 | EYA1 | 2.39E-05 | 0.2 |
| ENSP00000260682 | CYP2C9 | 2.39E-05 | 0.283 |
| ENSP00000234488 | CLCN6 | 2.39E-05 | 0.067 |
| ENSP00000319984 | CHRM2 | 2.39E-05 | 0.235 |
| ENSP00000360034 | SERBP1 | 2.39E-05 | 0.366 |
| ENSP00000361507 | HYI | 2.39E-05 | 0.062 |
| ENSP00000261303 | PSMC1 | 2.39E-05 | 0.248 |
| ENSP00000360871 | FCN1 | 2.39E-05 | 0.14 |
| ENSP00000368730 | PDE7A | 2.39E-05 | 0.037 |
| ENSP00000412309 | SLC14A1 | 2.39E-05 | 0.012 |
| ENSP00000291539 | PDE9A | 2.38E-05 | 0.05 |
| ENSP00000262502 | SLC12A3 | 2.38E-05 | 0.11 |
| ENSP00000299853 | POLR3E | 2.38E-05 | 0.152 |
| ENSP00000247182 | SIX1 | 2.38E-05 | 0.152 |
| ENSP00000265165 | LEF1 | 2.38E-05 | 0.387 |
| ENSP00000295228 | INHBB | 2.38E-05 | 0.054 |
| ENSP00000260408 | ADAM10 | 2.38E-05 | 0.224 |
| ENSP00000261531 | SNW1 | 2.38E-05 | 0.702 |
| ENSP00000332225 | PMCH | 2.38E-05 | 0.281 |
| ENSP00000416658 | NME6 | 2.38E-05 | 0.067 |
| ENSP00000365312 | ABI1 | 2.38E-05 | 0.168 |
| ENSP00000271915 | KCNN3 | 2.38E-05 | 0.076 |
| ENSP00000299847 | CHRFAM7A | 2.38E-05 | 0.093 |
| ENSP00000265174 | PAPSS1 | 2.38E-05 | 0.252 |
| ENSP00000283256 | SCN2A | 2.38E-05 | 0.165 |
| ENSP00000354532 | PNP | 2.38E-05 | 0.113 |
| ENSP00000350878 | S1PR3 | 2.38E-05 | 0.125 |
| ENSP00000265132 | AMBP | 2.37E-05 | 0.576 |
| ENSP00000228476 | DAO | 2.37E-05 | 0.111 |
| ENSP00000300659 | NFATC3 | 2.37E-05 | 0.075 |
| ENSP00000251968 | TSG101 | 2.37E-05 | 0.368 |
| ENSP00000294724 | AGL | 2.37E-05 | 0.351 |
| ENSP00000382423 | MAP3K1 | 2.37E-05 | 0.223 |
| ENSP00000302530 | BUB1 | 2.37E-05 | 0.821 |
| ENSP00000288135 | KIT | 2.37E-05 | 0.295 |
| ENSP00000252338 | FAM155B | 2.37E-05 | 0.322 |
| ENSP00000378577 | CLDN3 | 2.37E-05 | 0.202 |
| ENSP00000255476 | RFXAP | 2.37E-05 | 0.049 |
| ENSP00000253004 | ASS1 | 2.36E-05 | 0.134 |
| ENSP00000265131 | TNC | 2.36E-05 | 0.185 |
| ENSP00000358320 | POLR3GL | 2.36E-05 | 0.144 |
| ENSP00000355537 | ACTN2 | 2.36E-05 | 0.585 |
| ENSP00000330393 | LEPR | 2.36E-05 | 0.181 |
| ENSP00000358404 | TCF7L2 | 2.36E-05 | 0.309 |
| ENSP00000220562 | EXTL3 | 2.36E-05 | 0.25 |
| ENSP00000361287 | MAT1A | 2.36E-05 | 0.205 |
| ENSP00000335203 | ATPIF1 | 2.36E-05 | 0.055 |
| ENSP00000311453 | PDE8A | 2.36E-05 | 0.088 |
| ENSP00000382058 | POLR3G | 2.36E-05 | 0.097 |
| ENSP00000333122 | NR4A3 | 2.36E-05 | 0.057 |
| ENSP00000265572 | OPRK1 | 2.36E-05 | 0.15 |
| ENSP00000234111 | ODC1 | 2.35E-05 | 0.166 |
| ENSP00000336775 | SYNM | 2.35E-05 | 0.102 |
| ENSP00000306490 | CHRM1 | 2.35E-05 | 0.174 |
| ENSP00000363779 | IKBKAP | 2.35E-05 | 0.348 |
| ENSP00000278193 | LIN7C | 2.35E-05 | 0.373 |
| ENSP00000262366 | GLIS2 | 2.35E-05 | 0.08 |
| ENSP00000262493 | GNAO1 | 2.35E-05 | 0.332 |
| ENSP00000347495 | WARS | 2.35E-05 | 0.414 |
| ENSP00000343313 | ATG5 | 2.34E-05 | 0.194 |
| ENSP00000366843 | ATXN2 | 2.34E-05 | 0.288 |
| ENSP00000363019 | UBE2D1 | 2.34E-05 | 0.288 |
| ENSP00000245552 | NT5C | 2.34E-05 | 0.426 |
| ENSP00000327513 | CSF1 | 2.34E-05 | 0.047 |
| ENSP00000379364 | MOB1A | 2.34E-05 | 0.13 |
| ENSP00000354621 | SMURF1 | 2.34E-05 | 0.281 |
| ENSP00000345161 | SFTPB | 2.34E-05 | 0.134 |
| ENSP00000249749 | DLL4 | 2.34E-05 | 0.133 |
| ENSP00000308361 | P2RY14 | 2.34E-05 | 0.167 |
| ENSP00000367064 | CUBN | 2.34E-05 | 0.069 |
| ENSP00000345344 | CTSL1 | 2.34E-05 | 0.201 |
| ENSP00000347689 | PDE4C | 2.34E-05 | 0.095 |
| ENSP00000359609 | LMBRD1 | 2.33E-05 | 0.114 |
| ENSP00000343745 | DICER1 | 2.33E-05 | 0.507 |
| ENSP00000360687 | PTGDS | 2.33E-05 | 0.189 |
| ENSP00000287600 | PDE6D | 2.33E-05 | 0.034 |
| ENSP00000243052 | PDE1B | 2.33E-05 | 0.081 |
| ENSP00000361275 | PLK3 | 2.33E-05 | 0.131 |
| ENSP00000386541 | PSMD14 | 2.33E-05 | 0.865 |
| ENSP00000222254 | PIK3R2 | 2.33E-05 | 0.494 |
| ENSP00000277865 | GLUD1 | 2.33E-05 | 0.233 |
| ENSP00000379485 | PDE1C | 2.33E-05 | 0.079 |
| ENSP00000267814 | SORD | 2.32E-05 | 0.097 |
| ENSP00000266025 | TMEM115 | 2.32E-05 | 0.116 |
| ENSP00000267953 | BCL2A1 | 2.32E-05 | 0.102 |
| ENSP00000221421 | LHB | 2.32E-05 | 0.078 |
| ENSP00000262294 | TRIM37 | 2.32E-05 | 0.339 |
| ENSP00000250378 | CMA1 | 2.32E-05 | 0.115 |
| ENSP00000296464 | HSPA4L | 2.32E-05 | 0.195 |
| ENSP00000314348 | DDX10 | 2.32E-05 | 0.252 |
| ENSP00000230321 | MDFI | 2.32E-05 | 0.241 |
| ENSP00000334910 | PDE2A | 2.32E-05 | 0.094 |
| ENSP00000410674 | NOTCH4 | 2.31E-05 | 0.18 |
| ENSP00000362888 | ADO | 2.31E-05 | 0.151 |
| ENSP00000387281 | ADRA2B | 2.31E-05 | 0.095 |
| ENSP00000351755 | LPAR1 | 2.31E-05 | 0.276 |
| ENSP00000164139 | PYGM | 2.31E-05 | 0.549 |
| ENSP00000227163 | SPI1 | 2.31E-05 | 0.138 |
| ENSP00000334364 | DEFB106B | 2.31E-05 | 0.245 |
| ENSP00000203407 | UQCRC1 | 2.31E-05 | 0.529 |
| ENSP00000019103 | SCTR | 2.31E-05 | 0.132 |
| ENSP00000362413 | PGK1 | 2.31E-05 | 0.808 |
| ENSP00000336868 | CENPA | 2.31E-05 | 0.398 |
| ENSP00000251453 | RPS16 | 2.31E-05 | 0.427 |
| ENSP00000357711 | S100A7 | 2.30E-05 | 0.141 |
| ENSP00000338862 | BRIX1 | 2.30E-05 | 0.913 |
| ENSP00000268668 | NDUFB10 | 2.30E-05 | 0.32 |
| ENSP00000337128 | EDN3 | 2.30E-05 | 0.154 |
| ENSP00000276062 | NDUFB11 | 2.30E-05 | 0.468 |
| ENSP00000352798 | COL18A1 | 2.30E-05 | 0.261 |
| ENSP00000078429 | GNA11 | 2.30E-05 | 0.29 |
| ENSP00000364310 | H2AFX | 2.30E-05 | 0.773 |
| ENSP00000267082 | ITGB7 | 2.30E-05 | 0.358 |
| ENSP00000365747 | PQBP1 | 2.30E-05 | 0.098 |
| ENSP00000286063 | PDE11A | 2.30E-05 | 0.048 |
| ENSP00000369400 | NHS | 2.30E-05 | 0.321 |
| ENSP00000347198 | SRGAP1 | 2.29E-05 | 0.044 |
| ENSP00000351997 | MAP2K6 | 2.29E-05 | 0.211 |
| ENSP00000318195 | NCL | 2.29E-05 | 0.624 |
| ENSP00000203786 | ARHGAP4 | 2.29E-05 | 0.247 |
| ENSP00000406209 | EPN1 | 2.29E-05 | 0.083 |
| ENSP00000254958 | JAG1 | 2.29E-05 | 0.176 |
| ENSP00000310771 | GRB7 | 2.29E-05 | 0.162 |
| ENSP00000296099 | UCN | 2.28E-05 | 0.03 |
| ENSP00000319910 | EIF3M | 2.28E-05 | 0.17 |
| ENSP00000352561 | CALCR | 2.28E-05 | 0.218 |
| ENSP00000318142 | FUT7 | 2.28E-05 | 0.104 |
| ENSP00000296130 | CLEC3B | 2.28E-05 | 0.078 |
| ENSP00000302955 | RRM2 | 2.28E-05 | 0.653 |
| ENSP00000260795 | FGFR3 | 2.28E-05 | 0.332 |
| ENSP00000410815 | CFB | 2.28E-05 | 0.016 |
| ENSP00000242576 | UNG | 2.28E-05 | 0.23 |
| ENSP00000374902 | TRBV6-9 | 2.28E-05 | 0.044 |
| ENSP00000367714 | HES5 | 2.28E-05 | 0.222 |
| ENSP00000273353 | MYH15 | 2.28E-05 | 0.103 |
| ENSP00000281030 | THRSP | 2.28E-05 | 0.057 |
| ENSP00000351209 | EPHA2 | 2.28E-05 | 0.306 |
| ENSP00000266395 | PDE6H | 2.28E-05 | 0.045 |
| ENSP00000259075 | TANK | 2.28E-05 | 0.073 |
| ENSP00000384665 | LPAR2 | 2.27E-05 | 0.286 |
| ENSP00000226317 | CXCL6 | 2.27E-05 | 0.111 |
| ENSP00000311095 | CYP4A11 | 2.27E-05 | 0.168 |
| ENSP00000345689 | RAB5C | 2.27E-05 | 0.189 |
| ENSP00000277480 | LCN2 | 2.27E-05 | 0.27 |
| ENSP00000252029 | TYMP | 2.27E-05 | 0.278 |
| ENSP00000412292 | DEPDC1 | 2.27E-05 | 0.238 |
| ENSP00000360966 | SLC2A6 | 2.27E-05 | 0.075 |
| ENSP00000259726 | CDSN | 2.27E-05 | 0.119 |
| ENSP00000342374 | SNRPD2 | 2.27E-05 | 0.56 |
| ENSP00000308137 | FOLR1 | 2.27E-05 | 0.058 |
| ENSP00000245903 | CD70 | 2.27E-05 | 0.045 |
| ENSP00000369218 | RBM17 | 2.27E-05 | 0.072 |
| ENSP00000378306 | PPP3CB | 2.27E-05 | 0.232 |
| ENSP00000370842 | IL33 | 2.26E-05 | 0.07 |
| ENSP00000360797 | CARD9 | 2.26E-05 | 0.097 |
| ENSP00000374855 | IGLC1 | 2.26E-05 | 0.067 |
| ENSP00000251871 | MED17 | 2.26E-05 | 0.187 |
| ENSP00000299694 | BEAN1 | 2.26E-05 | 0.019 |
| ENSP00000310661 | PDE7B | 2.26E-05 | 0.047 |
| ENSP00000360141 | GNAS | 2.26E-05 | 0.713 |
| ENSP00000392466 | LDB1 | 2.26E-05 | 0.227 |
| ENSP00000291906 | PKN3 | 2.26E-05 | 0.041 |
| ENSP00000272163 | LBR | 2.26E-05 | 0.255 |
| ENSP00000258455 | MRPS9 | 2.26E-05 | 0.346 |
| ENSP00000282141 | CRYGC | 2.26E-05 | 0.072 |
| ENSP00000307706 | MT1E | 2.26E-05 | 0.1 |
| ENSP00000278572 | RPS3 | 2.26E-05 | 0.709 |
| ENSP00000291532 | TMPRSS3 | 2.25E-05 | 0.165 |
| ENSP00000329384 | IL22 | 2.25E-05 | 0.048 |
| ENSP00000318128 | BLOC1S4 | 2.25E-05 | 0.139 |
| ENSP00000348472 | PXK | 2.25E-05 | 0.099 |
| ENSP00000359285 | CHRNA4 | 2.25E-05 | 0.173 |
| ENSP00000352835 | MB | 2.25E-05 | 0.157 |
| ENSP00000359787 | IFI44L | 2.25E-05 | 0.159 |
| ENSP00000388794 | CD177 | 2.25E-05 | 0.042 |
| ENSP00000401321 | NOTCH4 | 2.25E-05 | 0.145 |
| ENSP00000375086 | CCL25 | 2.25E-05 | 0.12 |
| ENSP00000372449 | RP11-408E5.4 | 2.25E-05 | 0.06 |
| ENSP00000301037 | SGK494 | 2.25E-05 | 0.095 |
| ENSP00000403817 | NACA | 2.25E-05 | 0.214 |
| ENSP00000364163 | NOTCH4 | 2.25E-05 | 0.143 |
| ENSP00000266085 | TIMP3 | 2.25E-05 | 0.158 |
| ENSP00000064724 | CLDN11 | 2.25E-05 | 0.253 |
| ENSP00000356436 | PLA2G4A | 2.25E-05 | 0.305 |
| ENSP00000387262 | IMMT | 2.25E-05 | 0.328 |
| ENSP00000221418 | ECH1 | 2.24E-05 | 0.137 |
| ENSP00000305426 | TUB | 2.24E-05 | 0.104 |
| ENSP00000264487 | AREG | 2.24E-05 | 0.062 |
| ENSP00000270625 | RPS11 | 2.24E-05 | 0.249 |
| ENSP00000260118 | GGH | 2.24E-05 | 0.172 |
| ENSP00000375730 | RPL13A | 2.24E-05 | 0.216 |
| ENSP00000408581 | TMEM132D | 2.24E-05 | 0.07 |
| ENSP00000252699 | ACTN4 | 2.24E-05 | 0.498 |
| ENSP00000351015 | NF1 | 2.24E-05 | 0.372 |
| ENSP00000419361 | ADCY5 | 2.24E-05 | 0.502 |
| ENSP00000350425 | APOA4 | 2.24E-05 | 0.081 |
| ENSP00000352842 | PFKM | 2.24E-05 | 0.248 |
| ENSP00000386881 | DYSF | 2.24E-05 | 0.112 |
| ENSP00000302728 | GUSB | 2.23E-05 | 0.148 |
| ENSP00000336591 | PPP2R2B | 2.23E-05 | 0.406 |
| ENSP00000256592 | TSHB | 2.23E-05 | 0.06 |
| ENSP00000361554 | TIE1 | 2.23E-05 | 0.279 |
| ENSP00000338345 | SNCA | 2.23E-05 | 0.393 |
| ENSP00000264399 | PRKG2 | 2.23E-05 | 0.123 |
| ENSP00000372751 | NOTCH4 | 2.23E-05 | 0.134 |
| ENSP00000325448 | KARS | 2.23E-05 | 0.513 |
| ENSP00000233997 | AZU1 | 2.23E-05 | 0.105 |
| ENSP00000262033 | PTGES3 | 2.23E-05 | 0.462 |
| ENSP00000389207 | NOTCH4 | 2.23E-05 | 0.132 |
| ENSP00000238682 | TGFB3 | 2.23E-05 | 0.198 |
| ENSP00000287538 | ZIC3 | 2.23E-05 | 0.225 |
| ENSP00000367992 | ESD | 2.22E-05 | 0.232 |
| ENSP00000307859 | SLC22A18 | 2.22E-05 | 0.162 |
| ENSP00000370227 | AREGB | 2.22E-05 | 0.061 |
| ENSP00000171887 | TNS1 | 2.22E-05 | 0.25 |
| ENSP00000340396 | GBP5 | 2.22E-05 | 0.408 |
| ENSP00000266304 | TEF | 2.22E-05 | 0.305 |
| ENSP00000375921 | PAX3 | 2.22E-05 | 0.38 |
| ENSP00000371138 | FKBP1A | 2.22E-05 | 0.292 |
| ENSP00000319610 | NPAS3 | 2.22E-05 | 0.117 |
| ENSP00000294339 | TAL1 | 2.22E-05 | 0.185 |
| ENSP00000215781 | OSM | 2.21E-05 | 0.138 |
| ENSP00000350491 | PRIM1 | 2.21E-05 | 0.598 |
| ENSP00000347847 | MKL1 | 2.21E-05 | 0.032 |
| ENSP00000253799 | AOC2 | 2.21E-05 | 0.274 |
| ENSP00000239462 | TNN | 2.21E-05 | 0.203 |
| ENSP00000260270 | FDX1 | 2.21E-05 | 0.058 |
| ENSP00000270139 | IFNAR1 | 2.21E-05 | 0.124 |
| ENSP00000293362 | PSME3 | 2.21E-05 | 0.156 |
| ENSP00000005226 | USH1C | 2.21E-05 | 0.147 |
| ENSP00000358727 | GSTO1 | 2.21E-05 | 0.285 |
| ENSP00000345008 | FBLN5 | 2.20E-05 | 0.091 |
| ENSP00000315878 | MS4A6A | 2.20E-05 | 0.232 |
| ENSP00000354912 | MAGEE1 | 2.20E-05 | 0.124 |
| ENSP00000322159 | ATG4C | 2.20E-05 | 0.444 |
| ENSP00000361592 | ERMAP | 2.20E-05 | 0.024 |
| ENSP00000333769 | BSG | 2.20E-05 | 0.274 |
| ENSP00000070846 | PKP2 | 2.20E-05 | 0.09 |
| ENSP00000352575 | INPP5D | 2.20E-05 | 0.328 |
| ENSP00000264156 | MCM6 | 2.20E-05 | 0.702 |
| ENSP00000301653 | KRT16 | 2.19E-05 | 0.105 |
| ENSP00000263409 | LIFR | 2.19E-05 | 0.083 |
| ENSP00000250377 | KIAA0391 | 2.19E-05 | 0.031 |
| ENSP00000367872 | GNB1 | 2.19E-05 | 0.275 |
| ENSP00000386796 | SCN7A | 2.19E-05 | 0.043 |
| ENSP00000340093 | NAPEPLD | 2.19E-05 | 0.063 |
| ENSP00000340210 | CD59 | 2.19E-05 | 0.161 |
| ENSP00000264316 | TXK | 2.19E-05 | 0.064 |
| ENSP00000361894 | GNMT | 2.19E-05 | 0.153 |
| ENSP00000200453 | PPP1R15A | 2.19E-05 | 0.193 |
| ENSP00000290122 | CELA3A | 2.19E-05 | 0.144 |
| ENSP00000309968 | ADAM17 | 2.19E-05 | 0.177 |
| ENSP00000247970 | PIN1 | 2.18E-05 | 0.488 |
| ENSP00000226578 | MANBA | 2.18E-05 | 0.105 |
| ENSP00000262768 | TIMP2 | 2.18E-05 | 0.167 |
| ENSP00000300184 | MS4A7 | 2.18E-05 | 0.231 |
| ENSP00000314620 | MS4A1 | 2.18E-05 | 0.141 |
| ENSP00000361381 | SLC6A9 | 2.18E-05 | 0.388 |
| ENSP00000344254 | FXYD5 | 2.18E-05 | 0.281 |
| ENSP00000192788 | UHRF1BP1 | 2.18E-05 | 0.063 |
| ENSP00000332812 | PTGDR2 | 2.18E-05 | 0.129 |
| ENSP00000345681 | GATA2 | 2.17E-05 | 0.483 |
| ENSP00000267803 | DUOXA1 | 2.17E-05 | 0.199 |
| ENSP00000264995 | MRPL3 | 2.17E-05 | 0.859 |
| ENSP00000310880 | MAL | 2.17E-05 | 0.032 |
| ENSP00000346291 | MYO18A | 2.17E-05 | 0.129 |
| ENSP00000299092 | GPR176 | 2.17E-05 | 0.074 |
| ENSP00000307046 | SDC2 | 2.17E-05 | 0.485 |
| ENSP00000303575 | NUDT9 | 2.17E-05 | 0.116 |
| ENSP00000345580 | SLC25A10 | 2.17E-05 | 0.152 |
| ENSP00000419692 | RXRA | 2.16E-05 | 0.585 |
| ENSP00000263816 | LRP2 | 2.16E-05 | 0.17 |
| ENSP00000234626 | CDC7 | 2.16E-05 | 0.654 |
| ENSP00000315774 | NDUFS8 | 2.16E-05 | 0.61 |
| ENSP00000296097 | DNAJC5G | 2.16E-05 | 0.058 |
| ENSP00000310447 | GLS2 | 2.16E-05 | 0.215 |
| ENSP00000340454 | RAP1GDS1 | 2.16E-05 | 0.087 |
| ENSP00000347810 | DEFB107B | 2.16E-05 | 0.073 |
| ENSP00000284523 | WNT3A | 2.16E-05 | 0.534 |
| ENSP00000216225 | RBX1 | 2.16E-05 | 0.573 |
| ENSP00000313983 | WHSC1L1 | 2.16E-05 | 0.144 |
| ENSP00000268231 | 12-Sep | 2.15E-05 | 0.08 |
| ENSP00000248564 | GNG11 | 2.15E-05 | 0.095 |
| ENSP00000000442 | ESRRA | 2.15E-05 | 0.147 |
| ENSP00000372155 | PSME1 | 2.15E-05 | 0.148 |
| ENSP00000262304 | PKD1 | 2.15E-05 | 0.251 |
| ENSP00000386165 | CEBPD | 2.15E-05 | 0.371 |
| ENSP00000341243 | RBPJL | 2.15E-05 | 0.207 |
| ENSP00000304229 | HINT1 | 2.15E-05 | 0.297 |
| ENSP00000299293 | FRS2 | 2.15E-05 | 0.406 |
| ENSP00000221459 | LIN7B | 2.15E-05 | 0.035 |
| ENSP00000320940 | NCOA1 | 2.15E-05 | 0.328 |
| ENSP00000311997 | NEFH | 2.15E-05 | 0.3 |
| ENSP00000312455 | CFLAR | 2.15E-05 | 0.103 |
| ENSP00000295108 | NEUROD1 | 2.15E-05 | 0.238 |
| ENSP00000356248 | PTPN7 | 2.15E-05 | 0.11 |
| ENSP00000371341 | TNK2 | 2.15E-05 | 0.146 |
| ENSP00000366593 | TMEM201 | 2.15E-05 | 0.125 |
| ENSP00000340671 | DDIT3 | 2.15E-05 | 0.23 |
| ENSP00000359531 | GTF2B | 2.15E-05 | 0.623 |
| ENSP00000233336 | TTL | 2.14E-05 | 0.084 |
| ENSP00000274289 | PLK2 | 2.14E-05 | 0.242 |
| ENSP00000251849 | RAF1 | 2.14E-05 | 0.368 |
| ENSP00000252519 | ACE2 | 2.14E-05 | 0.1 |
| ENSP00000243924 | PI3 | 2.14E-05 | 0.29 |
| ENSP00000285850 | SLC7A7 | 2.14E-05 | 0.283 |
| ENSP00000313875 | CD46 | 2.14E-05 | 0.195 |
| ENSP00000273588 | AMT | 2.14E-05 | 0.08 |
| ENSP00000363603 | FUCA1 | 2.14E-05 | 0.064 |
| ENSP00000271324 | CD53 | 2.14E-05 | 0.336 |
| ENSP00000364028 | ECE1 | 2.14E-05 | 0.018 |
| ENSP00000216962 | PYGB | 2.13E-05 | 0.473 |
| ENSP00000227665 | APOA5 | 2.13E-05 | 0.233 |
| ENSP00000334198 | CACNA1H | 2.13E-05 | 0.179 |
| ENSP00000342087 | FHIT | 2.13E-05 | 0.247 |
| ENSP00000328169 | JAG2 | 2.13E-05 | 0.154 |
| ENSP00000265333 | VDAC1 | 2.13E-05 | 0.254 |
| ENSP00000349049 | KDM1A | 2.13E-05 | 0.226 |
| ENSP00000359778 | ELTD1 | 2.13E-05 | 0.426 |
| ENSP00000367001 | DPP6 | 2.13E-05 | 0.099 |
| ENSP00000222543 | TFPI2 | 2.13E-05 | 0.158 |
| ENSP00000294016 | ADCY9 | 2.13E-05 | 0.492 |
| ENSP00000341030 | CSN2 | 2.13E-05 | 0.066 |
| ENSP00000283928 | JAZF1 | 2.13E-05 | 0.194 |
| ENSP00000340361 | ACVR2B | 2.12E-05 | 0.161 |
| ENSP00000324105 | ENO3 | 2.12E-05 | 0.381 |
| ENSP00000272641 | NXPH2 | 2.12E-05 | 0.058 |
| ENSP00000402240 | KIAA1432 | 2.12E-05 | 0.138 |
| ENSP00000359643 | LPAR3 | 2.12E-05 | 0.24 |
| ENSP00000359790 | DST | 2.12E-05 | 0.195 |
| ENSP00000355167 | HORMAD1 | 2.12E-05 | 0.286 |
| ENSP00000332591 | NPY2R | 2.12E-05 | 0.109 |
| ENSP00000350894 | SERPINH1 | 2.11E-05 | 0.193 |
| ENSP00000416448 | HLA-DOA | 2.11E-05 | 0.025 |
| ENSP00000344782 | GFI1B | 2.11E-05 | 0.194 |
| ENSP00000410390 | HLA-DOB | 2.11E-05 | 0.027 |
| ENSP00000269980 | BCKDHA | 2.11E-05 | 0.181 |
| ENSP00000209728 | CDC6 | 2.11E-05 | 0.852 |
| ENSP00000265362 | SEMA3A | 2.11E-05 | 0.177 |
| ENSP00000233190 | NDUFS1 | 2.11E-05 | 0.218 |
| ENSP00000196551 | RPS5 | 2.11E-05 | 0.611 |
| ENSP00000324775 | TMIE | 2.11E-05 | 0.047 |
| ENSP00000353344 | ETS2 | 2.11E-05 | 0.164 |
| ENSP00000356925 | FCRLB | 2.11E-05 | 0.051 |
| ENSP00000276079 | NONO | 2.10E-05 | 0.386 |
| ENSP00000272928 | CXCR7 | 2.10E-05 | 0.11 |
| ENSP00000356024 | CR2 | 2.10E-05 | 0.056 |
| ENSP00000303252 | ZNF804A | 2.10E-05 | 0.109 |
| ENSP00000228140 | RPS13 | 2.10E-05 | 0.626 |
| ENSP00000343314 | FXYD1 | 2.10E-05 | 0.076 |
| ENSP00000334490 | ZDHHC8 | 2.10E-05 | 0.182 |
| ENSP00000319279 | MALT1 | 2.10E-05 | 0.064 |
| ENSP00000244766 | NRN1 | 2.10E-05 | 0.072 |
| ENSP00000291670 | FTCD | 2.10E-05 | 0.416 |
| ENSP00000245817 | TNFSF9 | 2.10E-05 | 0.052 |
| ENSP00000368244 | CHGB | 2.10E-05 | 0.107 |
| ENSP00000313681 | SPHK1 | 2.10E-05 | 0.253 |
| ENSP00000308576 | RHOD | 2.10E-05 | 0.246 |
| ENSP00000361382 | SFTPA1 | 2.09E-05 | 0.15 |
| ENSP00000254235 | ADCY7 | 2.09E-05 | 0.464 |
| ENSP00000292377 | GPC2 | 2.09E-05 | 0.144 |
| ENSP00000390018 | C6orf10 | 2.09E-05 | 0.219 |
| ENSP00000263431 | PRKCG | 2.09E-05 | 0.364 |
| ENSP00000278823 | MTA2 | 2.09E-05 | 0.457 |
| ENSP00000344666 | NF2 | 2.09E-05 | 0.399 |
| ENSP00000302139 | SYNPO | 2.09E-05 | 0.108 |
| ENSP00000249269 | PMPCB | 2.08E-05 | 0.45 |
| ENSP00000409403 | LRTOMT | 2.08E-05 | 0.03 |
| ENSP00000285407 | KLF10 | 2.08E-05 | 0.208 |
| ENSP00000249016 | MCHR1 | 2.08E-05 | 0.387 |
| ENSP00000225577 | RPS6KB1 | 2.08E-05 | 0.467 |
| ENSP00000262030 | ATP5B | 2.08E-05 | 0.61 |
| ENSP00000356700 | C1orf105 | 2.08E-05 | 0.053 |
| ENSP00000365103 | DAOA | 2.08E-05 | 0.067 |
| ENSP00000301790 | HRASLS5 | 2.08E-05 | 0.173 |
| ENSP00000327801 | P4HB | 2.08E-05 | 0.432 |
| ENSP00000247170 | DAAM1 | 2.07E-05 | 0.133 |
| ENSP00000245407 | SLC22A5 | 2.07E-05 | 0.104 |
| ENSP00000260956 | SSB | 2.07E-05 | 0.758 |
| ENSP00000345751 | SCNN1B | 2.07E-05 | 0.047 |
| ENSP00000332643 | NDN | 2.07E-05 | 0.243 |
| ENSP00000368914 | PSTPIP1 | 2.07E-05 | 0.312 |
| ENSP00000254351 | SDC1 | 2.07E-05 | 0.358 |
| ENSP00000263239 | DDX18 | 2.06E-05 | 0.768 |
| ENSP00000332293 | ASCL2 | 2.06E-05 | 0.15 |
| ENSP00000380514 | HAGH | 2.06E-05 | 0.074 |
| ENSP00000233114 | MDH1 | 2.06E-05 | 0.502 |
| ENSP00000269243 | MYH10 | 2.06E-05 | 0.256 |
| ENSP00000253107 | PPAN | 2.06E-05 | 0.644 |
| ENSP00000388910 | NFATC4 | 2.06E-05 | 0.076 |
| ENSP00000254963 | HSPA12B | 2.06E-05 | 0.089 |
| ENSP00000362873 | NDUFA8 | 2.06E-05 | 0.448 |
| ENSP00000263087 | ITGAE | 2.06E-05 | 0.076 |
| ENSP00000270517 | ECSIT | 2.06E-05 | 0.103 |
| ENSP00000234420 | MSH6 | 2.06E-05 | 0.744 |
| ENSP00000174621 | TRPV1 | 2.06E-05 | 0.346 |
| ENSP00000256759 | FST | 2.06E-05 | 0.405 |
| ENSP00000376322 | HOXD13 | 2.05E-05 | 0.139 |
| ENSP00000347294 | EYA4 | 2.05E-05 | 0.181 |
| ENSP00000373347 | SRGAP3 | 2.05E-05 | 0.172 |
| ENSP00000339377 | NPY5R | 2.05E-05 | 0.118 |
| ENSP00000230431 | DNPH1 | 2.05E-05 | 0.102 |
| ENSP00000316950 | MYEF2 | 2.05E-05 | 0.186 |
| ENSP00000310219 | HSPA6 | 2.05E-05 | 0.11 |
| ENSP00000380308 | DYNC1I2 | 2.05E-05 | 0.437 |
| ENSP00000295731 | IHH | 2.05E-05 | 0.711 |
| ENSP00000248114 | GFER | 2.05E-05 | 0.502 |
| ENSP00000308236 | COMMD1 | 2.05E-05 | 0.111 |
| ENSP00000263390 | MED26 | 2.05E-05 | 0.121 |
| ENSP00000303147 | MAT2A | 2.05E-05 | 0.171 |
| ENSP00000377954 | LARS | 2.05E-05 | 0.412 |
| ENSP00000418447 | PPP2CA | 2.05E-05 | 0.814 |
| ENSP00000337854 | HEXDC | 2.05E-05 | 0.032 |
| ENSP00000275428 | GGCT | 2.05E-05 | 0.146 |
| ENSP00000221566 | SGTA | 2.05E-05 | 0.179 |
| ENSP00000354691 | FCRL5 | 2.04E-05 | 0.088 |
| ENSP00000260102 | MRPL15 | 2.04E-05 | 0.616 |
| ENSP00000301764 | DDB1 | 2.04E-05 | 0.724 |
| ENSP00000268383 | CDR2 | 2.04E-05 | 0.267 |
| ENSP00000004921 | CCL18 | 2.04E-05 | 0.138 |
| ENSP00000310189 | MOB1B | 2.04E-05 | 0.075 |
| ENSP00000371393 | PGM2 | 2.03E-05 | 0.139 |
| ENSP00000225603 | CBX1 | 2.03E-05 | 0.247 |
| ENSP00000254108 | FUS | 2.03E-05 | 0.462 |
| ENSP00000364320 | MRTO4 | 2.03E-05 | 0.804 |
| ENSP00000265260 | PCNP | 2.03E-05 | 0.126 |
| ENSP00000354687 | MT-ND1 | 2.03E-05 | 0.128 |
| ENSP00000274192 | SRD5A1 | 2.03E-05 | 0.159 |
| ENSP00000177694 | TBX21 | 2.03E-05 | 0.186 |
| ENSP00000221448 | SNRNP70 | 2.03E-05 | 0.411 |
| ENSP00000354927 | MAP3K3 | 2.03E-05 | 0.162 |
| ENSP00000358031 | C1orf138 | 2.02E-05 | 0.023 |
| ENSP00000381333 | CRHR1 | 2.02E-05 | 0.115 |
| ENSP00000251472 | MAST1 | 2.02E-05 | 0.125 |
| ENSP00000353826 | LONP1 | 2.02E-05 | 0.262 |
| ENSP00000282406 | PLEKHH2 | 2.02E-05 | 0.093 |
| ENSP00000337212 | SERPINB7 | 2.02E-05 | 0.074 |
| ENSP00000261205 | SYT1 | 2.02E-05 | 0.369 |
| ENSP00000348888 | PIGR | 2.01E-05 | 0.117 |
| ENSP00000345530 | NEDD4 | 2.01E-05 | 0.414 |
| ENSP00000326238 | CAPZA3 | 2.01E-05 | 0.104 |
| ENSP00000234170 | CEBPZ | 2.01E-05 | 0.687 |
| ENSP00000271452 | NUF2 | 2.01E-05 | 0.706 |
| ENSP00000289902 | FCER1G | 2.01E-05 | 0.411 |
| ENSP00000374990 | IGHG1 | 2.01E-05 | 0.305 |
| ENSP00000377265 | TFAP2B | 2.01E-05 | 0.166 |
| ENSP00000324422 | ZYX | 2.01E-05 | 0.233 |
| ENSP00000253452 | COX4I1 | 2.01E-05 | 0.33 |
| ENSP00000349887 | ASNA1 | 2.01E-05 | 0.27 |
| ENSP00000341479 | KCNJ14 | 2.01E-05 | 0.08 |
| ENSP00000256857 | GRP | 2.01E-05 | 0.312 |
| ENSP00000240652 | IAPP | 2.01E-05 | 0.245 |
| ENSP00000385610 | MEX3C | 2.00E-05 | 0.053 |
| ENSP00000410626 | PHRF1 | 2.00E-05 | 0.111 |
| ENSP00000345023 | GSTO2 | 2.00E-05 | 0.198 |
| ENSP00000080059 | HDAC7 | 2.00E-05 | 0.161 |
| ENSP00000334650 | KCNK18 | 2.00E-05 | 0.08 |
| ENSP00000398655 | ITK | 2.00E-05 | 0.241 |
| ENSP00000341821 | FPR3 | 2.00E-05 | 0.201 |
| ENSP00000328251 | MINA | 2.00E-05 | 0.17 |
| ENSP00000309565 | UQCRH | 2.00E-05 | 0.397 |
| ENSP00000327025 | RORC | 2.00E-05 | 0.186 |
| ENSP00000216465 | GSTZ1 | 2.00E-05 | 0.191 |
| ENSP00000379204 | BMP7 | 2.00E-05 | 0.454 |
| ENSP00000298852 | PSMC3 | 2.00E-05 | 0.761 |
| ENSP00000369757 | RPS6 | 2.00E-05 | 0.807 |
| ENSP00000359563 | CDR1 | 1.99E-05 | 0.317 |
| ENSP00000310596 | LSM1 | 1.99E-05 | 0.396 |
| ENSP00000387219 | NFU1 | 1.99E-05 | 0.322 |
| ENSP00000372718 | HLA-DMB | 1.99E-05 | 0.011 |
| ENSP00000292035 | MED27 | 1.99E-05 | 0.099 |
| ENSP00000340347 | TCF7 | 1.99E-05 | 0.275 |
| ENSP00000364554 | SCN1A | 1.99E-05 | 0.145 |
| ENSP00000227451 | DTX4 | 1.99E-05 | 0.109 |
| ENSP00000302100 | LGALS4 | 1.99E-05 | 0.459 |
| ENSP00000411321 | HLA-DMB | 1.99E-05 | 0.032 |
| ENSP00000351113 | FRYL | 1.99E-05 | 0.09 |
| ENSP00000314343 | MED29 | 1.99E-05 | 0.236 |
| ENSP00000363571 | MUSK | 1.99E-05 | 0.299 |
| ENSP00000302811 | MTNR1A | 1.99E-05 | 0.225 |
| ENSP00000263635 | TANC1 | 1.99E-05 | 0.099 |
| ENSP00000357753 | IVL | 1.98E-05 | 0.447 |
| ENSP00000332371 | COL7A1 | 1.98E-05 | 0.192 |
| ENSP00000322579 | PHF23 | 1.98E-05 | 0.105 |
| ENSP00000298556 | HPRT1 | 1.98E-05 | 0.36 |
| ENSP00000248673 | ZFP36 | 1.98E-05 | 0.238 |
| ENSP00000360718 | RAB3B | 1.98E-05 | 0.255 |
| ENSP00000357789 | FLG | 1.98E-05 | 0.433 |
| ENSP00000347979 | FAS | 1.98E-05 | 0.098 |
| ENSP00000233957 | IL18R1 | 1.98E-05 | 0.084 |
| ENSP00000384675 | SOS1 | 1.98E-05 | 0.603 |
| ENSP00000209875 | CBX5 | 1.98E-05 | 0.518 |
| ENSP00000282030 | SETBP1 | 1.98E-05 | 0.079 |
| ENSP00000307188 | ASL | 1.98E-05 | 0.13 |
| ENSP00000413720 | CDKN1C | 1.98E-05 | 0.344 |
| ENSP00000280326 | CCT5 | 1.97E-05 | 0.52 |
| ENSP00000413471 | HLA-DMB | 1.97E-05 | 0.011 |
| ENSP00000366061 | NMS | 1.97E-05 | 0.323 |
| ENSP00000230882 | GHR | 1.97E-05 | 0.195 |
| ENSP00000315147 | ISYNA1 | 1.97E-05 | 0.132 |
| ENSP00000379531 | TMC5 | 1.97E-05 | 0.23 |
| ENSP00000304501 | ADORA2B | 1.97E-05 | 0.164 |
| ENSP00000281708 | FBXW7 | 1.97E-05 | 0.405 |
| ENSP00000375066 | HCAR2 | 1.97E-05 | 0.176 |
| ENSP00000312649 | PPARGC1B | 1.97E-05 | 0.119 |
| ENSP00000375809 | ERCC2 | 1.97E-05 | 0.381 |
| ENSP00000242577 | DYNLL1 | 1.97E-05 | 0.511 |
| ENSP00000280155 | ADRA2A | 1.97E-05 | 0.209 |
| ENSP00000304133 | SCG2 | 1.97E-05 | 0.12 |
| ENSP00000258874 | MTHFS | 1.97E-05 | 0.123 |
| ENSP00000271636 | CGN | 1.97E-05 | 0.224 |
| ENSP00000299300 | CCT2 | 1.97E-05 | 0.911 |
| ENSP00000333203 | SERPINA5 | 1.96E-05 | 0.164 |
| ENSP00000346148 | PRKAA1 | 1.96E-05 | 0.187 |
| ENSP00000267950 | ETFA | 1.96E-05 | 0.439 |
| ENSP00000282276 | MARS2 | 1.96E-05 | 0.088 |
| ENSP00000306561 | OGG1 | 1.96E-05 | 0.224 |
| ENSP00000350911 | CTSE | 1.96E-05 | 0.256 |
| ENSP00000271532 | FCRL4 | 1.96E-05 | 0.093 |
| ENSP00000262519 | SETD1A | 1.96E-05 | 0.237 |
| ENSP00000262506 | CSNK2A2 | 1.96E-05 | 0.502 |
| ENSP00000406463 | DNAJC7 | 1.96E-05 | 0.098 |
| ENSP00000282892 | MED21 | 1.96E-05 | 0.417 |
| ENSP00000261558 | AP5M1 | 1.96E-05 | 0.126 |
| ENSP00000373684 | STK38L | 1.96E-05 | 0.096 |
| ENSP00000211287 | MAPK13 | 1.96E-05 | 0.46 |
| ENSP00000303500 | PAH | 1.95E-05 | 0.232 |
| ENSP00000351430 | GYPE | 1.95E-05 | 0.066 |
| ENSP00000351379 | PFN3 | 1.95E-05 | 0.063 |
| ENSP00000289473 | NCF1 | 1.95E-05 | 0.313 |
| ENSP00000401504 | HLA-DOA | 1.95E-05 | 0.109 |
| ENSP00000301019 | CDT1 | 1.95E-05 | 0.773 |
| ENSP00000321445 | MSC | 1.95E-05 | 0.051 |
| ENSP00000372713 | HLA-DOA | 1.95E-05 | 0.078 |
| ENSP00000297268 | COL1A2 | 1.95E-05 | 0.64 |
| ENSP00000227868 | PDHX | 1.95E-05 | 0.478 |
| ENSP00000376436 | ETS1 | 1.95E-05 | 0.432 |
| ENSP00000329448 | NUTM1 | 1.95E-05 | 0.094 |
| ENSP00000360316 | DHCR24 | 1.94E-05 | 0.288 |
| ENSP00000388872 | CLUH | 1.94E-05 | 0.077 |
| ENSP00000222145 | RASIP1 | 1.94E-05 | 0.155 |
| ENSP00000356734 | PRRX1 | 1.94E-05 | 0.106 |
| ENSP00000263798 | TYRO3 | 1.94E-05 | 0.166 |
| ENSP00000334061 | HDAC6 | 1.94E-05 | 0.546 |
| ENSP00000297518 | CDK5 | 1.94E-05 | 0.778 |
| ENSP00000373570 | TYRP1 | 1.94E-05 | 0.23 |
| ENSP00000367605 | CCL3L3 | 1.93E-05 | 0.157 |
| ENSP00000412283 | IER3 | 1.93E-05 | 0.128 |
| ENSP00000265563 | PRKAR2A | 1.93E-05 | 0.208 |
| ENSP00000268379 | UQCRC2 | 1.93E-05 | 0.54 |
| ENSP00000312185 | ELMO1 | 1.93E-05 | 0.076 |
| ENSP00000416320 | NTM | 1.93E-05 | 0.255 |
| ENSP00000356425 | UCHL5 | 1.93E-05 | 0.808 |
| ENSP00000336687 | CBX3 | 1.93E-05 | 0.528 |
| ENSP00000377840 | CACNB1 | 1.93E-05 | 0.165 |
| ENSP00000216629 | BDKRB1 | 1.93E-05 | 0.307 |
| ENSP00000265857 | GET4 | 1.93E-05 | 0.18 |
| ENSP00000408411 | LAMP2 | 1.93E-05 | 0.523 |
| ENSP00000312081 | SSH3 | 1.93E-05 | 0.034 |
| ENSP00000362036 | MRPS16 | 1.93E-05 | 0.514 |
| ENSP00000386439 | SYNC | 1.93E-05 | 0.041 |
| ENSP00000264424 | GUCY1B3 | 1.93E-05 | 0.244 |
| ENSP00000379158 | CAST | 1.93E-05 | 0.14 |
| ENSP00000295588 | POGLUT1 | 1.93E-05 | 0.051 |
| ENSP00000287482 | SASS6 | 1.93E-05 | 0.069 |
| ENSP00000311028 | RPS14 | 1.92E-05 | 0.526 |
| ENSP00000306497 | KCNJ4 | 1.92E-05 | 0.062 |
| ENSP00000313420 | PRKDC | 1.92E-05 | 0.586 |
| ENSP00000367545 | TP73 | 1.92E-05 | 0.392 |
| ENSP00000339027 | RPLP0 | 1.92E-05 | 0.627 |
| ENSP00000409910 | HLA-E | 1.92E-05 | 0.04 |
| ENSP00000346643 | HKDC1 | 1.92E-05 | 0.181 |
| ENSP00000375022 | IGHV3-30 | 1.92E-05 | 0.184 |
| ENSP00000392028 | CHD7 | 1.92E-05 | 0.231 |
| ENSP00000406250 | HLA-DPA1 | 1.92E-05 | 0.065 |
| ENSP00000335307 | DEFB106A | 1.92E-05 | 0.302 |
| ENSP00000363941 | HLA-DPA1 | 1.92E-05 | 0.012 |
| ENSP00000363708 | BMPR2 | 1.92E-05 | 0.322 |
| ENSP00000293379 | ITGA5 | 1.92E-05 | 0.363 |
| ENSP00000252996 | TAF4 | 1.92E-05 | 0.237 |
| ENSP00000357986 | PLEKHA1 | 1.91E-05 | 0.107 |
| ENSP00000376097 | URI1 | 1.91E-05 | 0.354 |
| ENSP00000301843 | CTTN | 1.91E-05 | 0.431 |
| ENSP00000232003 | HRG | 1.91E-05 | 0.196 |
| ENSP00000331871 | RHD | 1.91E-05 | 0.117 |
| ENSP00000266544 | NDUFA9 | 1.91E-05 | 0.321 |
| ENSP00000378735 | DNAJC6 | 1.91E-05 | 0.346 |
| ENSP00000392330 | BCAP31 | 1.91E-05 | 0.291 |
| ENSP00000355471 | TFB2M | 1.91E-05 | 0.594 |
| ENSP00000248272 | GAN | 1.91E-05 | 0.2 |
| ENSP00000348635 | AMICA1 | 1.90E-05 | 0.376 |
| ENSP00000265983 | HPX | 1.90E-05 | 0.275 |
| ENSP00000329797 | CADM1 | 1.90E-05 | 0.136 |
| ENSP00000397139 | HLA-DPA1 | 1.90E-05 | 0.02 |
| ENSP00000363998 | ITCH | 1.90E-05 | 0.262 |
| ENSP00000365817 | HLA-E | 1.90E-05 | 0.09 |
| ENSP00000263317 | NOX4 | 1.90E-05 | 0.093 |
| ENSP00000234071 | PROC | 1.90E-05 | 0.163 |
| ENSP00000357980 | HTRA1 | 1.90E-05 | 0.169 |
| ENSP00000293308 | KRT8 | 1.90E-05 | 0.185 |
| ENSP00000342538 | STMN4 | 1.90E-05 | 0.081 |
| ENSP00000334458 | GATA4 | 1.89E-05 | 0.707 |
| ENSP00000255008 | SSTR4 | 1.89E-05 | 0.142 |
| ENSP00000275198 | TAAR6 | 1.89E-05 | 0.078 |
| ENSP00000355922 | CENPF | 1.89E-05 | 0.686 |
| ENSP00000358211 | HSPA12A | 1.89E-05 | 0.08 |
| ENSP00000345393 | MAFF | 1.89E-05 | 0.143 |
| ENSP00000257430 | APC | 1.89E-05 | 0.459 |
| ENSP00000377311 | FDX1L | 1.89E-05 | 0.089 |
| ENSP00000327758 | NKX2-5 | 1.89E-05 | 0.322 |
| ENSP00000244869 | EREG | 1.89E-05 | 0.056 |
| ENSP00000264930 | SLC12A7 | 1.89E-05 | 0.095 |
| ENSP00000417970 | FAM120B | 1.88E-05 | 0.015 |
| ENSP00000216911 | AURKA | 1.88E-05 | 0.898 |
| ENSP00000378323 | PPP3CA | 1.88E-05 | 0.401 |
| ENSP00000335306 | DPPA4 | 1.88E-05 | 0.441 |
| ENSP00000369312 | IL15RA | 1.88E-05 | 0.066 |
| ENSP00000288840 | SMAD6 | 1.88E-05 | 0.081 |
| ENSP00000230658 | ISL1 | 1.88E-05 | 0.491 |
| ENSP00000255381 | MYH4 | 1.88E-05 | 0.219 |
| ENSP00000405838 | HLA-DPA1 | 1.88E-05 | 0.008 |
| ENSP00000358963 | HTR1B | 1.88E-05 | 0.222 |
| ENSP00000333142 | PLA2G6 | 1.88E-05 | 0.328 |
| ENSP00000396704 | TOP2B | 1.88E-05 | 0.844 |
| ENSP00000254480 | SMARCC1 | 1.88E-05 | 0.445 |
| ENSP00000372170 | MSX1 | 1.88E-05 | 0.447 |
| ENSP00000252172 | MYH13 | 1.87E-05 | 0.145 |
| ENSP00000352603 | AP4M1 | 1.87E-05 | 0.15 |
| ENSP00000305260 | GNB2 | 1.87E-05 | 0.336 |
| ENSP00000258729 | IGF2BP3 | 1.87E-05 | 0.374 |
| ENSP00000337697 | DCX | 1.87E-05 | 0.384 |
| ENSP00000261015 | WDR12 | 1.87E-05 | 0.954 |
| ENSP00000322450 | NDUFV1 | 1.87E-05 | 0.26 |
| ENSP00000363489 | GDF5 | 1.87E-05 | 0.183 |
| ENSP00000278715 | HMBS | 1.87E-05 | 0.377 |
| ENSP00000284292 | NRGN | 1.87E-05 | 0.131 |
| ENSP00000302324 | RNASE3 | 1.87E-05 | 0.129 |
| ENSP00000386200 | FOXP2 | 1.87E-05 | 0.317 |
| ENSP00000367366 | PCDHA5 | 1.86E-05 | 0.06 |
| ENSP00000411096 | ABCA13 | 1.86E-05 | 0.169 |
| ENSP00000354791 | DCTN1 | 1.86E-05 | 0.592 |
| ENSP00000364709 | F10 | 1.86E-05 | 0.417 |
| ENSP00000329312 | IGLL1 | 1.86E-05 | 0.254 |
| ENSP00000293371 | DCD | 1.86E-05 | 0.131 |
| ENSP00000235628 | NT5C1A | 1.86E-05 | 0.478 |
| ENSP00000356520 | DHX9 | 1.86E-05 | 0.451 |
| ENSP00000357150 | CD1B | 1.86E-05 | 0.041 |
| ENSP00000264637 | THRA | 1.86E-05 | 0.301 |
| ENSP00000418379 | TAF1L | 1.86E-05 | 0.216 |
| ENSP00000381282 | VIMP | 1.86E-05 | 0.255 |
| ENSP00000317159 | CYC1 | 1.86E-05 | 0.42 |
| ENSP00000280362 | PTS | 1.85E-05 | 0.376 |
| ENSP00000359640 | MCOLN2 | 1.85E-05 | 0.18 |
| ENSP00000269582 | PNMT | 1.85E-05 | 0.097 |
| ENSP00000219150 | CORO1A | 1.85E-05 | 0.429 |
| ENSP00000322924 | HTR1F | 1.85E-05 | 0.178 |
| ENSP00000245503 | MYH2 | 1.85E-05 | 0.312 |
| ENSP00000244709 | TREM1 | 1.85E-05 | 0.157 |
| ENSP00000225430 | RPL19 | 1.85E-05 | 0.441 |
| ENSP00000405533 | CCL4L1 | 1.85E-05 | 0.089 |
| ENSP00000398930 | SGCE | 1.85E-05 | 0.238 |
| ENSP00000401513 | STRC | 1.85E-05 | 0.089 |
| ENSP00000264497 | IL21 | 1.85E-05 | 0.041 |
| ENSP00000268712 | NCOR1 | 1.85E-05 | 0.65 |
| ENSP00000296518 | GUCY1A3 | 1.85E-05 | 0.357 |
| ENSP00000245046 | EMC3 | 1.85E-05 | 0.068 |
| ENSP00000334002 | HAP1 | 1.85E-05 | 0.077 |
| ENSP00000355231 | BECN1 | 1.85E-05 | 0.403 |
| ENSP00000255262 | NMUR2 | 1.84E-05 | 0.5 |
| ENSP00000313661 | HTR1D | 1.84E-05 | 0.161 |
| ENSP00000384369 | METTL15 | 1.84E-05 | 0.079 |
| ENSP00000353508 | MAP2 | 1.84E-05 | 0.344 |
| ENSP00000364699 | SDHD | 1.84E-05 | 0.316 |
| ENSP00000342307 | FOXM1 | 1.84E-05 | 0.644 |
| ENSP00000262630 | ZBTB32 | 1.84E-05 | 0.224 |
| ENSP00000351732 | TMC2 | 1.84E-05 | 0.086 |
| ENSP00000368767 | ASPH | 1.84E-05 | 0.086 |
| ENSP00000366404 | TRIM27 | 1.84E-05 | 0.112 |
| ENSP00000229758 | FBXO5 | 1.84E-05 | 0.576 |
| ENSP00000301774 | BEST1 | 1.83E-05 | 0.151 |
| ENSP00000348551 | NCOR2 | 1.83E-05 | 0.494 |
| ENSP00000232564 | GNB4 | 1.83E-05 | 0.214 |
| ENSP00000281543 | GUF1 | 1.83E-05 | 0.15 |
| ENSP00000359423 | MTM1 | 1.83E-05 | 0.447 |
| ENSP00000257118 | PHC2 | 1.83E-05 | 0.161 |
| ENSP00000258105 | MRPL53 | 1.83E-05 | 0.084 |
| ENSP00000362361 | CDK9 | 1.83E-05 | 0.436 |
| ENSP00000262345 | IL12RB2 | 1.83E-05 | 0.078 |
| ENSP00000386722 | FCHSD2 | 1.83E-05 | 0.131 |
| ENSP00000290953 | AGRP | 1.83E-05 | 0.208 |
| ENSP00000353219 | NFIX | 1.83E-05 | 0.21 |
| ENSP00000360492 | NDUFA1 | 1.83E-05 | 0.391 |
| ENSP00000258796 | TTYH3 | 1.83E-05 | 0.155 |
| ENSP00000264025 | PVRL1 | 1.83E-05 | 0.156 |
| ENSP00000264932 | SDHA | 1.83E-05 | 0.185 |
| ENSP00000316772 | HMHA1 | 1.83E-05 | 0.369 |
| ENSP00000037502 | MYOC | 1.83E-05 | 0.176 |
| ENSP00000364700 | OMD | 1.83E-05 | 0.226 |
| ENSP00000365227 | AIF1 | 1.83E-05 | 0.092 |
| ENSP00000303754 | PPID | 1.83E-05 | 0.149 |
| ENSP00000262887 | XRCC1 | 1.83E-05 | 0.236 |
| ENSP00000244336 | CEACAM8 | 1.82E-05 | 0.045 |
| ENSP00000262873 | MYH7B | 1.82E-05 | 0.129 |
| ENSP00000265598 | LAMP3 | 1.82E-05 | 0.023 |
| ENSP00000294117 | GNG3 | 1.82E-05 | 0.176 |
| ENSP00000252229 | MICB | 1.82E-05 | 0.073 |
| ENSP00000332771 | CLCNKA | 1.82E-05 | 0.059 |
| ENSP00000300682 | ENGASE | 1.82E-05 | 0.059 |
| ENSP00000357697 | S100A2 | 1.82E-05 | 0.161 |
| ENSP00000373006 | MICB | 1.82E-05 | 0.075 |
| ENSP00000344524 | PPP1R9A | 1.82E-05 | 0.155 |
| ENSP00000368552 | SIRT5 | 1.82E-05 | 0.25 |
| ENSP00000351885 | PPP2R4 | 1.82E-05 | 0.484 |
| ENSP00000345193 | SHANK2 | 1.82E-05 | 0.227 |
| ENSP00000281043 | MYCN | 1.81E-05 | 0.279 |
| ENSP00000280190 | WDR17 | 1.81E-05 | 0.181 |
| ENSP00000229332 | CLEC4A | 1.81E-05 | 0.178 |
| ENSP00000329374 | SERPINA7 | 1.81E-05 | 0.213 |
| ENSP00000296387 | CLDN19 | 1.81E-05 | 0.135 |
| ENSP00000314214 | VAMP2 | 1.81E-05 | 0.437 |
| ENSP00000345512 | SEMA6A | 1.81E-05 | 0.127 |
| ENSP00000379339 | RPS29 | 1.81E-05 | 0.493 |
| ENSP00000361699 | CTPS1 | 1.81E-05 | 0.656 |
| ENSP00000407092 | MICB | 1.81E-05 | 0.066 |
| ENSP00000384192 | RASGRP3 | 1.81E-05 | 0.191 |
| ENSP00000419923 | KLF6 | 1.81E-05 | 0.165 |
| ENSP00000237858 | GLRX | 1.81E-05 | 0.109 |
| ENSP00000055077 | RFC2 | 1.81E-05 | 0.684 |
| ENSP00000257254 | APLNR | 1.81E-05 | 0.228 |
| ENSP00000364589 | CUL4A | 1.81E-05 | 0.33 |
| ENSP00000261023 | ITGAV | 1.81E-05 | 0.431 |
| ENSP00000373854 | ITGAD | 1.81E-05 | 0.033 |
| ENSP00000373614 | SELPLG | 1.81E-05 | 0.244 |
| ENSP00000360441 | RHOXF2 | 1.81E-05 | 0.558 |
| ENSP00000351894 | NCOA6 | 1.81E-05 | 0.368 |
| ENSP00000326804 | CUL1 | 1.81E-05 | 0.737 |
| ENSP00000315383 | ASPRV1 | 1.81E-05 | 0.106 |
| ENSP00000352665 | ATP2C1 | 1.81E-05 | 0.221 |
| ENSP00000312029 | UCP2 | 1.81E-05 | 0.203 |
| ENSP00000289779 | F11R | 1.81E-05 | 0.216 |
| ENSP00000309052 | CATSPER1 | 1.81E-05 | 0.228 |
| ENSP00000340477 | TMEM179 | 1.81E-05 | 0.027 |
| ENSP00000222673 | OGDH | 1.81E-05 | 0.481 |
| ENSP00000344936 | PTTG1 | 1.80E-05 | 0.493 |
| ENSP00000298690 | RNASE7 | 1.80E-05 | 0.048 |
| ENSP00000262418 | SLC4A1 | 1.80E-05 | 0.287 |
| ENSP00000392787 | TRIM27 | 1.80E-05 | 0.072 |
| ENSP00000275603 | CCT6A | 1.80E-05 | 0.745 |
| ENSP00000356789 | ATP1B1 | 1.80E-05 | 0.129 |
| ENSP00000354107 | SLC6A6 | 1.80E-05 | 0.415 |
| ENSP00000317992 | NOC2L | 1.80E-05 | 0.784 |
| ENSP00000257879 | ITGA7 | 1.80E-05 | 0.344 |
| ENSP00000377141 | ARRB1 | 1.80E-05 | 0.354 |
| ENSP00000363193 | MED12 | 1.80E-05 | 0.626 |
| ENSP00000364133 | TGFBR1 | 1.80E-05 | 0.52 |
| ENSP00000318057 | EGR3 | 1.80E-05 | 0.203 |
| ENSP00000265440 | TFEC | 1.79E-05 | 0.061 |
| ENSP00000318921 | NOSTRIN | 1.79E-05 | 0.052 |
| ENSP00000299084 | SPRED1 | 1.79E-05 | 0.06 |
| ENSP00000408395 | RBFOX3 | 1.79E-05 | 0.153 |
| ENSP00000352162 | ELAVL3 | 1.79E-05 | 0.178 |
| ENSP00000393693 | LTB | 1.79E-05 | 0.054 |
| ENSP00000356953 | SDHC | 1.79E-05 | 0.283 |
| ENSP00000390120 | LILRA6 | 1.79E-05 | 0.313 |
| ENSP00000264228 | SRD5A3 | 1.79E-05 | 0.146 |
| ENSP00000005340 | DVL2 | 1.79E-05 | 0.472 |
| ENSP00000295987 | SYN1 | 1.78E-05 | 0.527 |
| ENSP00000308716 | INHBC | 1.78E-05 | 0.046 |
| ENSP00000263636 | LY75 | 1.78E-05 | 0.121 |
| ENSP00000265970 | PIK3C2A | 1.78E-05 | 0.432 |
| ENSP00000272748 | KIAA1715 | 1.78E-05 | 0.088 |
| ENSP00000369419 | STEAP4 | 1.78E-05 | 0.133 |
| ENSP00000377958 | CCT4 | 1.78E-05 | 0.768 |
| ENSP00000394033 | KCNK2 | 1.78E-05 | 0.117 |
| ENSP00000360811 | LHX3 | 1.78E-05 | 0.15 |
| ENSP00000367615 | APRT | 1.78E-05 | 0.831 |
| ENSP00000338606 | SMUG1 | 1.78E-05 | 0.253 |
| ENSP00000348877 | GPI | 1.78E-05 | 0.573 |
| ENSP00000345083 | MAP2K3 | 1.77E-05 | 0.284 |
| ENSP00000345984 | TRAF3IP2 | 1.77E-05 | 0.081 |
| ENSP00000160740 | CIC | 1.77E-05 | 0.099 |
| ENSP00000343428 | GPR18 | 1.77E-05 | 0.421 |
| ENSP00000295256 | HPGDS | 1.77E-05 | 0.216 |
| ENSP00000310275 | BANF1 | 1.77E-05 | 0.656 |
| ENSP00000320147 | EZH2 | 1.77E-05 | 0.721 |
| ENSP00000331897 | IDH2 | 1.77E-05 | 0.451 |
| ENSP00000336740 | LIMK1 | 1.77E-05 | 0.186 |
| ENSP00000216714 | APEX1 | 1.77E-05 | 0.557 |
| ENSP00000284486 | FAM167A | 1.77E-05 | 0.142 |
| ENSP00000312304 | TPMT | 1.77E-05 | 0.052 |
| ENSP00000353608 | DSC3 | 1.77E-05 | 0.268 |
| ENSP00000300209 | METTL21B | 1.77E-05 | 0.096 |
| ENSP00000294785 | NCSTN | 1.77E-05 | 0.182 |
| ENSP00000310244 | RASGRP1 | 1.77E-05 | 0.234 |
| ENSP00000285814 | MKI67IP | 1.77E-05 | 0.938 |
| ENSP00000262904 | E2F3 | 1.76E-05 | 0.165 |
| ENSP00000167586 | KRT14 | 1.76E-05 | 0.229 |
| ENSP00000272644 | GPR17 | 1.76E-05 | 0.347 |
| ENSP00000325527 | FBN1 | 1.76E-05 | 0.518 |
| ENSP00000261208 | HAL | 1.76E-05 | 0.211 |
| ENSP00000250457 | EGLN3 | 1.76E-05 | 0.174 |
| ENSP00000377446 | SUCLG1 | 1.76E-05 | 0.601 |
| ENSP00000355520 | RGS7 | 1.76E-05 | 0.245 |
| ENSP00000357998 | SEC63 | 1.76E-05 | 0.337 |
| ENSP00000314129 | TFE3 | 1.76E-05 | 0.226 |
| ENSP00000320503 | EDC3 | 1.75E-05 | 0.112 |
| ENSP00000294413 | RHCE | 1.75E-05 | 0.15 |
| ENSP00000367301 | NDP | 1.75E-05 | 0.278 |
| ENSP00000395102 | LTB | 1.75E-05 | 0.055 |
| ENSP00000169551 | TIMM21 | 1.75E-05 | 0.241 |
| ENSP00000349142 | ATP5C1 | 1.75E-05 | 0.546 |
| ENSP00000265728 | DBF4 | 1.75E-05 | 0.536 |
| ENSP00000382323 | OTOG | 1.75E-05 | 0.092 |
| ENSP00000351486 | NTRK1 | 1.75E-05 | 0.283 |
| ENSP00000349437 | IGF2R | 1.75E-05 | 0.381 |
| ENSP00000358563 | DKC1 | 1.75E-05 | 0.953 |
| ENSP00000301522 | PRDX2 | 1.74E-05 | 0.198 |
| ENSP00000368314 | NRCAM | 1.74E-05 | 0.273 |
| ENSP00000396843 | NFIC | 1.74E-05 | 0.256 |
| ENSP00000278968 | TAGLN | 1.74E-05 | 0.325 |
| ENSP00000250448 | FOXA1 | 1.74E-05 | 0.361 |
| ENSP00000359729 | SLC9A6 | 1.74E-05 | 0.429 |
| ENSP00000328173 | C1S | 1.74E-05 | 0.304 |
| ENSP00000256682 | ARF3 | 1.73E-05 | 0.475 |
| ENSP00000258749 | AOAH | 1.73E-05 | 0.236 |
| ENSP00000315955 | FOXA2 | 1.73E-05 | 0.531 |
| ENSP00000356545 | CACNA1E | 1.73E-05 | 0.296 |
| ENSP00000284811 | TCEB1 | 1.73E-05 | 0.715 |
| ENSP00000234701 | CLCA1 | 1.73E-05 | 0.21 |
| ENSP00000317659 | GUK1 | 1.73E-05 | 0.612 |
| ENSP00000363676 | RPL11 | 1.73E-05 | 0.832 |
| ENSP00000346037 | RPLP1 | 1.73E-05 | 0.479 |
| ENSP00000266000 | DAXX | 1.73E-05 | 0.287 |
| ENSP00000081029 | MRPS35 | 1.73E-05 | 0.294 |
| ENSP00000417132 | BAP1 | 1.73E-05 | 0.251 |
| ENSP00000330343 | SUMO3 | 1.73E-05 | 0.498 |
| ENSP00000233156 | TFPI | 1.73E-05 | 0.054 |
| ENSP00000256495 | BHLHE40 | 1.73E-05 | 0.226 |
| ENSP00000333194 | RGS19 | 1.73E-05 | 0.406 |
| ENSP00000268124 | POLG | 1.73E-05 | 0.184 |
| ENSP00000300651 | MED1 | 1.72E-05 | 0.249 |
| ENSP00000415480 | TRIM15 | 1.72E-05 | 0.052 |
| ENSP00000305924 | PPP4R4 | 1.72E-05 | 0.139 |
| ENSP00000259667 | HINT2 | 1.72E-05 | 0.291 |
| ENSP00000264036 | MCAM | 1.72E-05 | 0.061 |
| ENSP00000348205 | ATP5G1 | 1.72E-05 | 0.421 |
| ENSP00000378965 | SNTB1 | 1.72E-05 | 0.085 |
| ENSP00000233627 | NDUFS7 | 1.72E-05 | 0.362 |
| ENSP00000343348 | VMAC | 1.72E-05 | 0.042 |
| ENSP00000349486 | SCRIB | 1.72E-05 | 0.295 |
| ENSP00000292180 | FLAD1 | 1.71E-05 | 0.439 |
| ENSP00000293328 | STAT5B | 1.71E-05 | 0.286 |
| ENSP00000269260 | ARRB2 | 1.71E-05 | 0.838 |
| ENSP00000349003 | PTGER3 | 1.71E-05 | 0.181 |
| ENSP00000357112 | AIM2 | 1.71E-05 | 0.08 |
| ENSP00000217971 | PGRMC1 | 1.71E-05 | 0.443 |
| ENSP00000358158 | HIST2H2AA3 | 1.71E-05 | 0.491 |
| ENSP00000301908 | PNOC | 1.71E-05 | 0.23 |
| ENSP00000314067 | PAK2 | 1.71E-05 | 0.403 |
| ENSP00000319851 | CHDH | 1.71E-05 | 0.128 |
| ENSP00000358812 | PDCD11 | 1.71E-05 | 0.802 |
| ENSP00000357540 | FUOM | 1.71E-05 | 0.108 |
| ENSP00000378033 | KCNK4 | 1.71E-05 | 0.103 |
| ENSP00000355518 | FH | 1.71E-05 | 0.262 |
| ENSP00000366306 | SPRY2 | 1.70E-05 | 0.312 |
| ENSP00000304858 | ORMDL3 | 1.70E-05 | 0.244 |
| ENSP00000290100 | EPB41 | 1.70E-05 | 0.283 |
| ENSP00000369038 | EEF1E1 | 1.70E-05 | 0.853 |
| ENSP00000377007 | CENPN | 1.70E-05 | 0.63 |
| ENSP00000269321 | ARHGDIA | 1.70E-05 | 0.486 |
| ENSP00000318646 | RPS15A | 1.70E-05 | 0.509 |
| ENSP00000324101 | CD151 | 1.70E-05 | 0.126 |
| ENSP00000302846 | PTGER4 | 1.70E-05 | 0.243 |
| ENSP00000256383 | EIF2S1 | 1.70E-05 | 0.776 |
| ENSP00000348068 | SERPINA1 | 1.70E-05 | 0.284 |
| ENSP00000216479 | AHSA1 | 1.70E-05 | 0.411 |
| ENSP00000381250 | APOF | 1.70E-05 | 0.353 |
| ENSP00000332549 | GRIN2A | 1.70E-05 | 0.403 |
| ENSP00000228606 | CYP27B1 | 1.70E-05 | 0.146 |
| ENSP00000222266 | PSENEN | 1.70E-05 | 0.238 |
| ENSP00000356370 | CRB1 | 1.70E-05 | 0.299 |
| ENSP00000345873 | SLC26A3 | 1.70E-05 | 0.191 |
| ENSP00000370372 | NLN | 1.69E-05 | 0.124 |
| ENSP00000380280 | FGFR1 | 1.69E-05 | 0.701 |
| ENSP00000363851 | EDA2R | 1.69E-05 | 0.439 |
| ENSP00000262946 | UQCR11 | 1.69E-05 | 0.559 |
| ENSP00000415805 | AIF1 | 1.69E-05 | 0.106 |
| ENSP00000377941 | ACTN1 | 1.69E-05 | 0.521 |
| ENSP00000291481 | HAPLN4 | 1.69E-05 | 0.055 |
| ENSP00000367173 | RNF207 | 1.69E-05 | 0.14 |
| ENSP00000314441 | METTL1 | 1.69E-05 | 0.395 |
| ENSP00000261191 | ASUN | 1.69E-05 | 0.612 |
| ENSP00000366248 | CORT | 1.69E-05 | 0.139 |
| ENSP00000290399 | SIM2 | 1.69E-05 | 0.316 |
| ENSP00000296777 | CARTPT | 1.69E-05 | 0.187 |
| ENSP00000264501 | KIAA1109 | 1.69E-05 | 0.135 |
| ENSP00000378394 | PSAP | 1.69E-05 | 0.207 |
| ENSP00000355629 | COG2 | 1.69E-05 | 0.163 |
| ENSP00000339906 | PAX4 | 1.69E-05 | 0.163 |
| ENSP00000391249 | 9-Sep | 1.69E-05 | 0.127 |
| ENSP00000220478 | SCG3 | 1.69E-05 | 0.081 |
| ENSP00000275169 | GPR6 | 1.69E-05 | 0.101 |
| ENSP00000375872 | ATG16L1 | 1.69E-05 | 0.242 |
| ENSP00000359719 | PRKACB | 1.69E-05 | 0.375 |
| ENSP00000351905 | TGFBR2 | 1.69E-05 | 0.49 |
| ENSP00000263629 | MTIF2 | 1.69E-05 | 0.724 |
| ENSP00000261465 | HSD11B1 | 1.68E-05 | 0.148 |
| ENSP00000372912 | LY6G6F | 1.68E-05 | 0.003 |
| ENSP00000358165 | FCGR1A | 1.68E-05 | 0.181 |
| ENSP00000300151 | MRPL16 | 1.68E-05 | 0.554 |
| ENSP00000349365 | IL27 | 1.68E-05 | 0.047 |
| ENSP00000386896 | ITGA6 | 1.68E-05 | 0.358 |
| ENSP00000264658 | FBXL20 | 1.68E-05 | 0.174 |
| ENSP00000351049 | PAK4 | 1.68E-05 | 0.223 |
| ENSP00000288943 | DUSP2 | 1.68E-05 | 0.133 |
| ENSP00000268150 | MFGE8 | 1.68E-05 | 0.205 |
| ENSP00000351682 | CNDP1 | 1.68E-05 | 0.183 |
| ENSP00000319141 | CYBRD1 | 1.68E-05 | 0.109 |
| ENSP00000317580 | NEUROG1 | 1.68E-05 | 0.299 |
| ENSP00000364204 | PINK1 | 1.68E-05 | 0.161 |
| ENSP00000324122 | PRPF31 | 1.68E-05 | 0.569 |
| ENSP00000305877 | NMUR1 | 1.68E-05 | 0.31 |
| ENSP00000368350 | TPT1 | 1.68E-05 | 0.475 |
| ENSP00000375009 | IGHV3-9 | 1.68E-05 | 0.233 |
| ENSP00000225174 | PPIF | 1.68E-05 | 0.129 |
| ENSP00000350336 | DSCR8 | 1.68E-05 | 0.481 |
| ENSP00000215565 | NDUFB7 | 1.68E-05 | 0.547 |
| ENSP00000315700 | CHAF1B | 1.67E-05 | 0.641 |
| ENSP00000298223 | FOLR2 | 1.67E-05 | 0.062 |
| ENSP00000270509 | FBN3 | 1.67E-05 | 0.109 |
| ENSP00000310572 | PSMC5 | 1.67E-05 | 0.747 |
| ENSP00000299964 | NNMT | 1.67E-05 | 0.114 |
| ENSP00000356070 | MAPKAPK2 | 1.67E-05 | 0.215 |
| ENSP00000320184 | MRPS23 | 1.67E-05 | 0.791 |
| ENSP00000373487 | KRT18 | 1.67E-05 | 0.208 |
| ENSP00000339095 | RPS7 | 1.67E-05 | 0.734 |
| ENSP00000324172 | ATP2B2 | 1.67E-05 | 0.152 |
| ENSP00000367934 | UQCRQ | 1.67E-05 | 0.614 |
| ENSP00000295600 | MITF | 1.67E-05 | 0.364 |
| ENSP00000357047 | MED23 | 1.66E-05 | 0.375 |
| ENSP00000289575 | SLCO2B1 | 1.66E-05 | 0.364 |
| ENSP00000281806 | MCHR2 | 1.66E-05 | 0.344 |
| ENSP00000246551 | HCST | 1.66E-05 | 0.378 |
| ENSP00000338191 | SNTB2 | 1.66E-05 | 0.092 |
| ENSP00000368190 | NPHS1 | 1.66E-05 | 0.31 |
| ENSP00000404623 | DAXX | 1.66E-05 | 0.262 |
| ENSP00000373884 | RYR3 | 1.66E-05 | 0.164 |
| ENSP00000227155 | CD82 | 1.66E-05 | 0.34 |
| ENSP00000410481 | LTB | 1.66E-05 | 0.072 |
| ENSP00000379839 | GNE | 1.66E-05 | 0.412 |
| ENSP00000330070 | NPW | 1.66E-05 | 0.216 |
| ENSP00000313059 | WNK1 | 1.66E-05 | 0.255 |
| ENSP00000282957 | CPB1 | 1.66E-05 | 0.232 |
| ENSP00000342385 | PTGES | 1.66E-05 | 0.096 |
| ENSP00000279259 | FAU | 1.66E-05 | 0.71 |
| ENSP00000370989 | CD274 | 1.66E-05 | 0.149 |
| ENSP00000393557 | SLC4A4 | 1.66E-05 | 0.152 |
| ENSP00000420040 | KCNIP2 | 1.66E-05 | 0.104 |
| ENSP00000251808 | GRHL2 | 1.66E-05 | 0.161 |
| ENSP00000358770 | SLK | 1.65E-05 | 0.498 |
| ENSP00000340691 | EIF4EBP1 | 1.65E-05 | 0.398 |
| ENSP00000347169 | NUMB | 1.65E-05 | 0.455 |
| ENSP00000332194 | HIST2H2AC | 1.65E-05 | 0.631 |
| ENSP00000358865 | INA | 1.65E-05 | 0.113 |
| ENSP00000355124 | KRT19 | 1.65E-05 | 0.231 |
| ENSP00000305480 | FEN1 | 1.65E-05 | 0.852 |
| ENSP00000379888 | RPS8 | 1.65E-05 | 0.645 |
| ENSP00000327048 | MAF | 1.65E-05 | 0.32 |
| ENSP00000225371 | EPX | 1.65E-05 | 0.033 |
| ENSP00000386229 | RGS14 | 1.65E-05 | 0.218 |
| ENSP00000260950 | MSTN | 1.65E-05 | 0.32 |
| ENSP00000264634 | WNT5A | 1.65E-05 | 0.286 |
| ENSP00000202831 | SLC24A6 | 1.65E-05 | 0.099 |
| ENSP00000357113 | IFI16 | 1.64E-05 | 0.151 |
| ENSP00000271234 | FNBP1L | 1.64E-05 | 0.154 |
| ENSP00000375018 | IGHV3-23 | 1.64E-05 | 0.325 |
| ENSP00000266744 | ASCL1 | 1.64E-05 | 0.387 |
| ENSP00000379704 | 3-Sep | 1.64E-05 | 0.096 |
| ENSP00000412566 | SNRPB | 1.64E-05 | 0.781 |
| ENSP00000270288 | WTIP | 1.64E-05 | 0.059 |
| ENSP00000329287 | SNN | 1.64E-05 | 0.154 |
| ENSP00000293275 | CCL16 | 1.64E-05 | 0.179 |
| ENSP00000373810 | NOP16 | 1.64E-05 | 0.315 |
| ENSP00000353415 | PRKAR1B | 1.63E-05 | 0.205 |
| ENSP00000419952 | KCNAB1 | 1.63E-05 | 0.147 |
| ENSP00000300408 | PHB | 1.63E-05 | 0.206 |
| ENSP00000332695 | RARG | 1.63E-05 | 0.204 |
| ENSP00000366246 | GPC6 | 1.63E-05 | 0.303 |
| ENSP00000332139 | CFD | 1.63E-05 | 0.221 |
| ENSP00000241600 | MRPS2 | 1.63E-05 | 0.496 |
| ENSP00000256906 | HRH4 | 1.63E-05 | 0.193 |
| ENSP00000299601 | LEO1 | 1.63E-05 | 0.566 |
| ENSP00000346294 | S100A4 | 1.63E-05 | 0.371 |
| ENSP00000315659 | DNMBP | 1.63E-05 | 0.142 |
| ENSP00000307288 | MCM7 | 1.63E-05 | 0.89 |
| ENSP00000362208 | MRPS15 | 1.63E-05 | 0.282 |
| ENSP00000274547 | GABRB2 | 1.63E-05 | 0.183 |
| ENSP00000332887 | UQCR10 | 1.63E-05 | 0.432 |
| ENSP00000363277 | RPS6KA1 | 1.63E-05 | 0.192 |
| ENSP00000374069 | PTPRN2 | 1.62E-05 | 0.37 |
| ENSP00000298139 | WRN | 1.62E-05 | 0.574 |
| ENSP00000377793 | PRC1 | 1.62E-05 | 0.843 |
| ENSP00000359151 | DBT | 1.62E-05 | 0.321 |
| ENSP00000309117 | RBFOX1 | 1.62E-05 | 0.267 |
| ENSP00000363643 | P2RY4 | 1.62E-05 | 0.249 |
| ENSP00000362116 | PGC | 1.62E-05 | 0.142 |
| ENSP00000259512 | DERL1 | 1.62E-05 | 0.353 |
| ENSP00000244458 | PACSIN1 | 1.62E-05 | 0.258 |
| ENSP00000231061 | SPARC | 1.62E-05 | 0.534 |
| ENSP00000251074 | NUP37 | 1.62E-05 | 0.929 |
| ENSP00000265565 | SCAP | 1.62E-05 | 0.116 |
| ENSP00000291009 | PIP | 1.62E-05 | 0.222 |
| ENSP00000289707 | SLAMF8 | 1.62E-05 | 0.29 |
| ENSP00000294304 | LRP5 | 1.62E-05 | 0.425 |
| ENSP00000307508 | CALB2 | 1.62E-05 | 0.283 |
| ENSP00000367059 | ESPN | 1.62E-05 | 0.243 |
| ENSP00000261464 | TRAF5 | 1.62E-05 | 0.173 |
| ENSP00000301729 | ECI1 | 1.61E-05 | 0.373 |
| ENSP00000255390 | SCO1 | 1.61E-05 | 0.094 |
| ENSP00000331944 | AEN | 1.61E-05 | 0.523 |
| ENSP00000222304 | HAMP | 1.61E-05 | 0.195 |
| ENSP00000254301 | LGALS3 | 1.61E-05 | 0.234 |
| ENSP00000290551 | BTG2 | 1.61E-05 | 0.156 |
| ENSP00000299766 | MC4R | 1.61E-05 | 0.38 |
| ENSP00000343619 | HOXA9 | 1.61E-05 | 0.305 |
| ENSP00000228918 | LTBR | 1.61E-05 | 0.137 |
| ENSP00000219476 | TSC2 | 1.61E-05 | 0.509 |
| ENSP00000338358 | SERPINB6 | 1.61E-05 | 0.104 |
| ENSP00000305464 | APLN | 1.61E-05 | 0.283 |
| ENSP00000298841 | SERPINA4 | 1.61E-05 | 0.188 |
| ENSP00000337194 | PRPF4B | 1.61E-05 | 0.808 |
| ENSP00000346050 | RPS3A | 1.61E-05 | 0.387 |
| ENSP00000351446 | WDR5 | 1.61E-05 | 0.515 |
| ENSP00000159060 | NOX3 | 1.61E-05 | 0.047 |
| ENSP00000278947 | SCN2B | 1.61E-05 | 0.192 |
| ENSP00000361508 | PLTP | 1.61E-05 | 0.136 |
| ENSP00000320130 | DYNC1I1 | 1.60E-05 | 0.346 |
| ENSP00000408012 | CCHCR1 | 1.60E-05 | 0.21 |
| ENSP00000253496 | F12 | 1.60E-05 | 0.159 |
| ENSP00000320813 | DEFB104A | 1.60E-05 | 0.171 |
| ENSP00000354416 | CCL28 | 1.60E-05 | 0.171 |
| ENSP00000362446 | ZNF79 | 1.60E-05 | 0.125 |
| ENSP00000332326 | TNFAIP2 | 1.60E-05 | 0.062 |
| ENSP00000321853 | SERPINF2 | 1.60E-05 | 0.465 |
| ENSP00000282074 | SPC25 | 1.60E-05 | 0.716 |
| ENSP00000261366 | LMNB1 | 1.60E-05 | 0.662 |
| ENSP00000367608 | CA9 | 1.60E-05 | 0.145 |
| ENSP00000346067 | RPSA | 1.60E-05 | 0.361 |
| ENSP00000311856 | SLC25A30 | 1.60E-05 | 0.03 |
| ENSP00000370259 | GABPB1 | 1.60E-05 | 0.119 |
| ENSP00000288937 | MRPL17 | 1.60E-05 | 0.432 |
| ENSP00000336543 | SNURF | 1.60E-05 | 0.699 |
| ENSP00000297313 | RGS20 | 1.60E-05 | 0.256 |
| ENSP00000249041 | GALR3 | 1.60E-05 | 0.368 |
| ENSP00000358155 | HIST2H2AA4 | 1.60E-05 | 0.551 |
| ENSP00000262193 | PSMB1 | 1.59E-05 | 0.624 |
| ENSP00000303279 | IARS2 | 1.59E-05 | 0.616 |
| ENSP00000357726 | S100A12 | 1.59E-05 | 0.189 |
| ENSP00000386069 | ADRA2C | 1.59E-05 | 0.215 |
| ENSP00000360616 | GRIN1 | 1.59E-05 | 0.418 |
| ENSP00000290541 | PSMB4 | 1.59E-05 | 0.853 |
| ENSP00000339845 | DROSHA | 1.59E-05 | 0.438 |
| ENSP00000225728 | MED31 | 1.59E-05 | 0.28 |
| ENSP00000291294 | PTGIR | 1.59E-05 | 0.217 |
| ENSP00000257915 | TFCP2 | 1.59E-05 | 0.2 |
| ENSP00000368924 | TFAP2A | 1.59E-05 | 0.285 |
| ENSP00000255039 | HAPLN2 | 1.59E-05 | 0.061 |
| ENSP00000256001 | ACTR3B | 1.59E-05 | 0.073 |
| ENSP00000362058 | NDUFS5 | 1.59E-05 | 0.539 |
| ENSP00000216951 | GSS | 1.59E-05 | 0.251 |
| ENSP00000279387 | PPP4C | 1.59E-05 | 0.493 |
| ENSP00000292907 | COX7A1 | 1.59E-05 | 0.47 |
| ENSP00000215375 | ATP5D | 1.59E-05 | 0.814 |
| ENSP00000366901 | FAM216A | 1.59E-05 | 0.216 |
| ENSP00000347088 | LARGE | 1.59E-05 | 0.34 |
| ENSP00000222214 | GCDH | 1.58E-05 | 0.194 |
| ENSP00000254940 | NIP7 | 1.58E-05 | 0.905 |
| ENSP00000369317 | KRT6A | 1.58E-05 | 0.137 |
| ENSP00000295956 | FLNB | 1.58E-05 | 0.297 |
| ENSP00000350616 | DDC | 1.58E-05 | 0.338 |
| ENSP00000361475 | NCS1 | 1.58E-05 | 0.263 |
| ENSP00000285039 | MYO5B | 1.58E-05 | 0.269 |
| ENSP00000311596 | ZNF699 | 1.58E-05 | 0.106 |
| ENSP00000264741 | ITGA9 | 1.58E-05 | 0.344 |
| ENSP00000358596 | DUSP5 | 1.58E-05 | 0.131 |
| ENSP00000300404 | B4GALNT2 | 1.58E-05 | 0.305 |
| ENSP00000357301 | RXFP4 | 1.58E-05 | 0.231 |
| ENSP00000386096 | MYO18B | 1.58E-05 | 0.125 |
| ENSP00000262018 | SGCA | 1.58E-05 | 0.214 |
| ENSP00000281243 | QDPR | 1.58E-05 | 0.184 |
| ENSP00000331288 | TMEM173 | 1.58E-05 | 0.178 |
| ENSP00000313070 | PPIP5K2 | 1.58E-05 | 0.357 |
| ENSP00000399388 | TCF19 | 1.57E-05 | 0.125 |
| ENSP00000242317 | DNAI1 | 1.57E-05 | 0.371 |
| ENSP00000299727 | GALR1 | 1.57E-05 | 0.197 |
| ENSP00000260665 | LRPPRC | 1.57E-05 | 0.486 |
| ENSP00000364895 | ZBTB17 | 1.57E-05 | 0.13 |
| ENSP00000322323 | FAM20C | 1.57E-05 | 0.201 |
| ENSP00000296051 | HPS3 | 1.57E-05 | 0.155 |
| ENSP00000249923 | COPB1 | 1.57E-05 | 0.756 |
| ENSP00000376609 | GRK5 | 1.57E-05 | 0.294 |
| ENSP00000272462 | MALL | 1.57E-05 | 0.146 |
| ENSP00000361043 | RAD54L | 1.57E-05 | 0.586 |
| ENSP00000337103 | CHAT | 1.57E-05 | 0.412 |
| ENSP00000371451 | DNAJC21 | 1.57E-05 | 0.271 |
| ENSP00000329468 | ADAP2 | 1.57E-05 | 0.361 |
| ENSP00000217893 | TAF9 | 1.57E-05 | 0.596 |
| ENSP00000311740 | NDUFA11 | 1.57E-05 | 0.354 |
| ENSP00000285381 | CA3 | 1.57E-05 | 0.082 |
| ENSP00000331040 | OLIG2 | 1.57E-05 | 0.436 |
| ENSP00000359506 | FMR1 | 1.57E-05 | 0.729 |
| ENSP00000328472 | S1PR5 | 1.57E-05 | 0.265 |
| ENSP00000351665 | CLIP1 | 1.57E-05 | 0.67 |
| ENSP00000264245 | ARHGAP31 | 1.57E-05 | 0.138 |
| ENSP00000263980 | SLC9A1 | 1.57E-05 | 0.227 |
| ENSP00000295206 | EN1 | 1.57E-05 | 0.332 |
| ENSP00000364731 | F7 | 1.57E-05 | 0.205 |
| ENSP00000264554 | SHC2 | 1.57E-05 | 0.27 |
| ENSP00000302166 | KCNK9 | 1.57E-05 | 0.173 |
| ENSP00000368538 | TNFRSF4 | 1.56E-05 | 0.094 |
| ENSP00000330284 | NPBWR1 | 1.56E-05 | 0.278 |
| ENSP00000376652 | EVL | 1.56E-05 | 0.368 |
| ENSP00000371872 | SLC35G5 | 1.56E-05 | 0.07 |
| ENSP00000386029 | GABRR2 | 1.56E-05 | 0.05 |
| ENSP00000270223 | DMWD | 1.56E-05 | 0.135 |
| ENSP00000406157 | PAPSS2 | 1.56E-05 | 0.26 |
| ENSP00000272317 | RPS27A | 1.56E-05 | 0.787 |
| ENSP00000276416 | BIN3 | 1.56E-05 | 0.174 |
| ENSP00000353864 | PAK3 | 1.56E-05 | 0.532 |
| ENSP00000327268 | NDUFV2 | 1.56E-05 | 0.444 |
| ENSP00000352606 | HAPLN3 | 1.56E-05 | 0.065 |
| ENSP00000364512 | G6PC2 | 1.56E-05 | 0.074 |
| ENSP00000307259 | P2RY12 | 1.56E-05 | 0.167 |
| ENSP00000321656 | CDC25C | 1.56E-05 | 0.739 |
| ENSP00000358099 | RGS10 | 1.56E-05 | 0.208 |
| ENSP00000354335 | MPEG1 | 1.56E-05 | 0.247 |
| ENSP00000300900 | CA4 | 1.56E-05 | 0.265 |
| ENSP00000284154 | GRAP | 1.56E-05 | 0.331 |
| ENSP00000318089 | LPP | 1.56E-05 | 0.191 |
| ENSP00000276594 | PRDM14 | 1.56E-05 | 0.204 |
| ENSP00000339720 | NDUFA4 | 1.56E-05 | 0.506 |
| ENSP00000303727 | CHRNA7 | 1.56E-05 | 0.233 |
| ENSP00000328708 | RXFP3 | 1.56E-05 | 0.217 |
| ENSP00000383953 | RGS6 | 1.56E-05 | 0.214 |
| ENSP00000287907 | HTR5A | 1.56E-05 | 0.179 |
| ENSP00000326981 | IMP3 | 1.55E-05 | 0.806 |
| ENSP00000354158 | ERC1 | 1.55E-05 | 0.292 |
| ENSP00000362948 | MED18 | 1.55E-05 | 0.263 |
| ENSP00000297347 | MED30 | 1.55E-05 | 0.226 |
| ENSP00000332454 | RIPK4 | 1.55E-05 | 0.206 |
| ENSP00000350592 | MAGEA12 | 1.55E-05 | 0.245 |
| ENSP00000002165 | FUCA2 | 1.55E-05 | 0.038 |
| ENSP00000280700 | NGLY1 | 1.55E-05 | 0.271 |
| ENSP00000322909 | FHL2 | 1.55E-05 | 0.306 |
| ENSP00000274311 | PELO | 1.55E-05 | 0.396 |
| ENSP00000323588 | SOX2 | 1.55E-05 | 0.62 |
| ENSP00000230056 | GMNN | 1.55E-05 | 0.86 |
| ENSP00000320447 | NR2C2 | 1.55E-05 | 0.136 |
| ENSP00000247191 | DLGAP5 | 1.55E-05 | 0.632 |
| ENSP00000374014 | TM6SF2 | 1.55E-05 | 0.046 |
| ENSP00000375893 | GPR55 | 1.55E-05 | 0.257 |
| ENSP00000344218 | C1orf162 | 1.55E-05 | 0.348 |
| ENSP00000300591 | LOXHD1 | 1.55E-05 | 0.072 |
| ENSP00000258886 | IREB2 | 1.55E-05 | 0.206 |
| ENSP00000282470 | SPARCL1 | 1.55E-05 | 0.293 |
| ENSP00000405890 | PBX1 | 1.55E-05 | 0.347 |
| ENSP00000329684 | GALR2 | 1.55E-05 | 0.172 |
| ENSP00000297562 | AP5Z1 | 1.55E-05 | 0.187 |
| ENSP00000354486 | GOLGA4 | 1.55E-05 | 0.389 |
| ENSP00000253381 | DEFB118 | 1.54E-05 | 0.187 |
| ENSP00000262626 | HPN | 1.54E-05 | 0.206 |
| ENSP00000307766 | HTR1E | 1.54E-05 | 0.192 |
| ENSP00000200181 | ITGB4 | 1.54E-05 | 0.344 |
| ENSP00000323076 | NDUFAF3 | 1.54E-05 | 0.133 |
| ENSP00000342850 | SERPINA6 | 1.54E-05 | 0.094 |
| ENSP00000298838 | PACSIN3 | 1.54E-05 | 0.209 |
| ENSP00000263552 | TBXAS1 | 1.54E-05 | 0.268 |
| ENSP00000267868 | RAD51 | 1.54E-05 | 0.955 |
| ENSP00000404450 | LTB | 1.54E-05 | 0.066 |
| ENSP00000346001 | RPL3 | 1.54E-05 | 0.701 |
| ENSP00000298910 | LRRK2 | 1.54E-05 | 0.386 |
| ENSP00000342007 | CYP1A2 | 1.54E-05 | 0.527 |
| ENSP00000337340 | MED19 | 1.54E-05 | 0.211 |
| ENSP00000261783 | ARG2 | 1.54E-05 | 0.169 |
| ENSP00000259456 | HEMGN | 1.54E-05 | 0.243 |
| ENSP00000014930 | HEBP1 | 1.54E-05 | 0.156 |
| ENSP00000325526 | WNT11 | 1.54E-05 | 0.331 |
| ENSP00000302790 | XPO6 | 1.54E-05 | 0.093 |
| ENSP00000341838 | TNNI3 | 1.54E-05 | 0.454 |
| ENSP00000384690 | MMD2 | 1.54E-05 | 0.149 |
| ENSP00000367930 | OXER1 | 1.54E-05 | 0.226 |
| ENSP00000368727 | XDH | 1.54E-05 | 0.174 |
| ENSP00000286398 | SMC2 | 1.53E-05 | 0.882 |
| ENSP00000342557 | IL4I1 | 1.53E-05 | 0.298 |
| ENSP00000193391 | IMPG2 | 1.53E-05 | 0.077 |
| ENSP00000362335 | SAR1A | 1.53E-05 | 0.543 |
| ENSP00000290341 | IGF2BP1 | 1.53E-05 | 0.607 |
| ENSP00000265028 | DNAJB11 | 1.53E-05 | 0.126 |
| ENSP00000332668 | TSKU | 1.53E-05 | 0.165 |
| ENSP00000356243 | UBE2T | 1.53E-05 | 0.778 |
| ENSP00000322716 | GLYR1 | 1.53E-05 | 0.076 |
| ENSP00000253247 | NOL11 | 1.53E-05 | 0.79 |
| ENSP00000354826 | CALD1 | 1.53E-05 | 0.338 |
| ENSP00000204615 | THPO | 1.53E-05 | 0.188 |
| ENSP00000249636 | PIAS1 | 1.53E-05 | 0.484 |
| ENSP00000252809 | GDF15 | 1.53E-05 | 0.199 |
| ENSP00000325660 | CNTN1 | 1.53E-05 | 0.209 |
| ENSP00000358783 | NPBWR2 | 1.53E-05 | 0.146 |
| ENSP00000368030 | ATAD3A | 1.53E-05 | 0.209 |
| ENSP00000241337 | GSTM2 | 1.53E-05 | 0.231 |
| ENSP00000264257 | IL1RL2 | 1.53E-05 | 0.093 |
| ENSP00000336829 | FGG | 1.53E-05 | 0.347 |
| ENSP00000370589 | NOP56 | 1.52E-05 | 0.846 |
| ENSP00000354168 | TSSK6 | 1.52E-05 | 0.121 |
| ENSP00000263088 | PLD2 | 1.52E-05 | 0.499 |
| ENSP00000354676 | OSTC | 1.52E-05 | 0.162 |
| ENSP00000322486 | OTOS | 1.52E-05 | 0.047 |
| ENSP00000259037 | NDUFB5 | 1.52E-05 | 0.79 |
| ENSP00000253934 | TMEM204 | 1.52E-05 | 0.451 |
| ENSP00000363157 | TNFSF15 | 1.52E-05 | 0.216 |
| ENSP00000292427 | CYP11B1 | 1.52E-05 | 0.245 |
| ENSP00000266735 | SNRPF | 1.52E-05 | 0.878 |
| ENSP00000262584 | RPL8 | 1.52E-05 | 0.775 |
| ENSP00000287908 | STEAP2 | 1.52E-05 | 0.067 |
| ENSP00000401303 | SHC1 | 1.52E-05 | 0.467 |
| ENSP00000301457 | NDUFA7 | 1.52E-05 | 0.371 |
| ENSP00000354728 | MT-ND4L | 1.51E-05 | 0.108 |
| ENSP00000171111 | KEAP1 | 1.51E-05 | 0.292 |
| ENSP00000377486 | MRPL19 | 1.51E-05 | 0.698 |
| ENSP00000225927 | NAGLU | 1.51E-05 | 0.144 |
| ENSP00000253024 | TRIM28 | 1.51E-05 | 0.781 |
| ENSP00000174618 | MNT | 1.51E-05 | 0.419 |
| ENSP00000251582 | ADAMTS2 | 1.51E-05 | 0.155 |
| ENSP00000265395 | HIBADH | 1.51E-05 | 0.118 |
| ENSP00000346045 | RPS17 | 1.51E-05 | 0.479 |
| ENSP00000216037 | XBP1 | 1.51E-05 | 0.33 |
| ENSP00000377384 | PSMC3IP | 1.51E-05 | 0.204 |
| ENSP00000338774 | BCL11A | 1.51E-05 | 0.178 |
| ENSP00000349259 | SPTBN1 | 1.51E-05 | 0.301 |
| ENSP00000342136 | CREB3 | 1.51E-05 | 0.094 |
| ENSP00000324890 | CD28 | 1.51E-05 | 0.1 |
| ENSP00000342222 | TRPV2 | 1.51E-05 | 0.125 |
| ENSP00000269385 | CBX8 | 1.51E-05 | 0.328 |
| ENSP00000365439 | HNRNPK | 1.51E-05 | 0.83 |
| ENSP00000221996 | CRX | 1.51E-05 | 0.344 |
| ENSP00000362344 | FPGS | 1.51E-05 | 0.181 |
| ENSP00000261733 | ALDH2 | 1.50E-05 | 0.34 |
| ENSP00000344668 | KRIT1 | 1.50E-05 | 0.313 |
| ENSP00000353072 | ATP2A3 | 1.50E-05 | 0.172 |
| ENSP00000234677 | SARS | 1.50E-05 | 0.322 |
| ENSP00000256178 | LYVE1 | 1.50E-05 | 0.316 |
| ENSP00000298510 | PRDX3 | 1.50E-05 | 0.403 |
| ENSP00000341835 | MYOCD | 1.50E-05 | 0.243 |
| ENSP00000340088 | THEG | 1.50E-05 | 0.382 |
| ENSP00000369427 | RBBP7 | 1.50E-05 | 0.844 |
| ENSP00000300574 | CRK | 1.50E-05 | 0.877 |
| ENSP00000320291 | OSBPL1A | 1.50E-05 | 0.163 |
| ENSP00000302046 | RNASE6 | 1.50E-05 | 0.084 |
| ENSP00000368801 | WBP4 | 1.50E-05 | 0.27 |
| ENSP00000263657 | PNO1 | 1.50E-05 | 0.807 |
| ENSP00000302625 | AXIN2 | 1.50E-05 | 0.537 |
| ENSP00000295797 | PRKCI | 1.50E-05 | 0.374 |
| ENSP00000401445 | ERN1 | 1.50E-05 | 0.461 |
| ENSP00000209668 | ADH1A | 1.50E-05 | 0.412 |
| ENSP00000296677 | F2RL1 | 1.50E-05 | 0.479 |
| ENSP00000363880 | CUL2 | 1.50E-05 | 0.533 |
| ENSP00000351407 | ARNT | 1.49E-05 | 0.301 |
| ENSP00000354995 | ABCG1 | 1.49E-05 | 0.405 |
| ENSP00000383977 | TEK | 1.49E-05 | 0.159 |
| ENSP00000256733 | SAA2 | 1.49E-05 | 0.076 |
| ENSP00000285599 | MAN2B2 | 1.49E-05 | 0.083 |
| ENSP00000054666 | VAMP3 | 1.49E-05 | 0.556 |
| ENSP00000302111 | MGMT | 1.49E-05 | 0.456 |
| ENSP00000249499 | HOXD9 | 1.49E-05 | 0.332 |
| ENSP00000386378 | CD207 | 1.49E-05 | 0.067 |
| ENSP00000356903 | UAP1 | 1.49E-05 | 0.098 |
| ENSP00000307080 | EGLN2 | 1.49E-05 | 0.211 |
| ENSP00000367923 | SUCLA2 | 1.49E-05 | 0.74 |
| ENSP00000321674 | 4-Sep | 1.48E-05 | 0.159 |
| ENSP00000319651 | DMRTA1 | 1.48E-05 | 0.232 |
| ENSP00000252242 | KRT5 | 1.48E-05 | 0.359 |
| ENSP00000349967 | CCRL2 | 1.48E-05 | 0.082 |
| ENSP00000329915 | PSMG1 | 1.48E-05 | 0.718 |
| ENSP00000351631 | FAM76B | 1.48E-05 | 0.374 |
| ENSP00000270586 | PSMB6 | 1.48E-05 | 0.784 |
| ENSP00000291527 | TFF1 | 1.48E-05 | 0.368 |
| ENSP00000336888 | SLC44A2 | 1.48E-05 | 0.078 |
| ENSP00000300406 | GNGT2 | 1.48E-05 | 0.246 |
| ENSP00000398290 | NDUFA3 | 1.48E-05 | 0.269 |
| ENSP00000300935 | RAB8A | 1.48E-05 | 0.606 |
| ENSP00000368686 | E2F4 | 1.48E-05 | 0.316 |
| ENSP00000366915 | KLF5 | 1.48E-05 | 0.13 |
| ENSP00000359546 | SPANXD | 1.48E-05 | 0.414 |
| ENSP00000258145 | GNS | 1.48E-05 | 0.167 |
| ENSP00000216124 | ARSA | 1.48E-05 | 0.298 |
| ENSP00000217423 | CST4 | 1.48E-05 | 0.138 |
| ENSP00000386171 | ESRRG | 1.47E-05 | 0.215 |
| ENSP00000352408 | BABAM1 | 1.47E-05 | 0.358 |
| ENSP00000314813 | OAZ1 | 1.47E-05 | 0.406 |
| ENSP00000220514 | OIP5 | 1.47E-05 | 0.544 |
| ENSP00000264734 | CLDN16 | 1.47E-05 | 0.252 |
| ENSP00000376822 | STEAP3 | 1.47E-05 | 0.092 |
| ENSP00000397297 | NTF3 | 1.47E-05 | 0.312 |
| ENSP00000259874 | IER3 | 1.47E-05 | 0.126 |
| ENSP00000304668 | HARS | 1.47E-05 | 0.303 |
| ENSP00000354499 | MT-CO1 | 1.47E-05 | 0.278 |
| ENSP00000297770 | CPA6 | 1.47E-05 | 0.11 |
| ENSP00000300215 | EPB42 | 1.47E-05 | 0.234 |
| ENSP00000360483 | TMEM48 | 1.47E-05 | 0.685 |
| ENSP00000380252 | NFE2L2 | 1.47E-05 | 0.449 |
| ENSP00000354677 | GPX7 | 1.47E-05 | 0.454 |
| ENSP00000261349 | LRP6 | 1.47E-05 | 0.386 |
| ENSP00000263270 | AP2S1 | 1.47E-05 | 0.577 |
| ENSP00000219255 | PARD6A | 1.47E-05 | 0.453 |
| ENSP00000252102 | NDUFA2 | 1.46E-05 | 0.503 |
| ENSP00000257860 | PRPH | 1.46E-05 | 0.287 |
| ENSP00000245323 | EFNB2 | 1.46E-05 | 0.452 |
| ENSP00000371532 | VLDLR | 1.46E-05 | 0.185 |
| ENSP00000350844 | PAX5 | 1.46E-05 | 0.553 |
| ENSP00000274137 | NDUFS6 | 1.46E-05 | 0.354 |
| ENSP00000333919 | BTLA | 1.46E-05 | 0.081 |
| ENSP00000304408 | COL3A1 | 1.46E-05 | 0.705 |
| ENSP00000216862 | CYP24A1 | 1.46E-05 | 0.226 |
| ENSP00000255389 | PEMT | 1.46E-05 | 0.264 |
| ENSP00000256447 | CD180 | 1.46E-05 | 0.1 |
| ENSP00000322180 | DSCC1 | 1.46E-05 | 0.511 |
| ENSP00000310226 | RAB1B | 1.46E-05 | 0.725 |
| ENSP00000311402 | SLC4A2 | 1.46E-05 | 0.176 |
| ENSP00000287322 | BAG4 | 1.46E-05 | 0.137 |
| ENSP00000223321 | PSMA2 | 1.46E-05 | 0.978 |
| ENSP00000221975 | RPS19 | 1.46E-05 | 0.639 |
| ENSP00000354982 | MT-CO3 | 1.46E-05 | 0.277 |
| ENSP00000245185 | MT2A | 1.46E-05 | 0.154 |
| ENSP00000340896 | ASH2L | 1.46E-05 | 0.473 |
| ENSP00000252490 | APOC4 | 1.45E-05 | 0.25 |
| ENSP00000378130 | CFI | 1.45E-05 | 0.394 |
| ENSP00000368544 | VIT | 1.45E-05 | 0.097 |
| ENSP00000260731 | KIF11 | 1.45E-05 | 0.925 |
| ENSP00000258682 | CAMK2B | 1.45E-05 | 0.408 |
| ENSP00000257600 | DTX1 | 1.45E-05 | 0.117 |
| ENSP00000253099 | MRPL4 | 1.45E-05 | 0.716 |
| ENSP00000270221 | EMP3 | 1.45E-05 | 0.247 |
| ENSP00000238112 | CPSF3 | 1.45E-05 | 0.878 |
| ENSP00000384432 | ICOSLG | 1.45E-05 | 0.203 |
| ENSP00000258955 | RSAD1 | 1.45E-05 | 0.134 |
| ENSP00000349359 | SASH3 | 1.45E-05 | 0.31 |
| ENSP00000257566 | TBX3 | 1.45E-05 | 0.292 |
| ENSP00000355580 | KCNK1 | 1.45E-05 | 0.174 |
| ENSP00000225893 | HNF1B | 1.45E-05 | 0.421 |
| ENSP00000306548 | MRPL13 | 1.45E-05 | 0.89 |
| ENSP00000403536 | GAMT | 1.45E-05 | 0.449 |
| ENSP00000352400 | NUP214 | 1.45E-05 | 0.402 |
| ENSP00000246554 | COX6B1 | 1.45E-05 | 0.408 |
| ENSP00000416463 | RASGRP4 | 1.44E-05 | 0.139 |
| ENSP00000387019 | UXS1 | 1.44E-05 | 0.171 |
| ENSP00000222308 | FKBP8 | 1.44E-05 | 0.269 |
| ENSP00000324742 | IL32 | 1.44E-05 | 0.052 |
| ENSP00000279804 | CTF1 | 1.44E-05 | 0.086 |
| ENSP00000345789 | MUM1 | 1.44E-05 | 0.045 |
| ENSP00000271889 | CREB3L4 | 1.44E-05 | 0.142 |
| ENSP00000410649 | MAS1L | 1.44E-05 | 0.11 |
| ENSP00000264515 | RBBP5 | 1.44E-05 | 0.418 |
| ENSP00000364858 | CARD16 | 1.44E-05 | 0.122 |
| ENSP00000314029 | SENP3 | 1.44E-05 | 0.155 |
| ENSP00000261879 | APH1B | 1.44E-05 | 0.145 |
| ENSP00000352358 | TRPV6 | 1.44E-05 | 0.229 |
| ENSP00000301407 | CGB1 | 1.43E-05 | 0.028 |
| ENSP00000252268 | DPF2 | 1.43E-05 | 0.121 |
| ENSP00000221431 | SARS2 | 1.43E-05 | 0.329 |
| ENSP00000352527 | KCNK5 | 1.43E-05 | 0.073 |
| ENSP00000316121 | CDCA8 | 1.43E-05 | 0.747 |
| ENSP00000222693 | CAV2 | 1.43E-05 | 0.152 |
| ENSP00000267845 | HDC | 1.43E-05 | 0.192 |
| ENSP00000269216 | GATA6 | 1.43E-05 | 0.506 |
| ENSP00000327583 | RANBP1 | 1.43E-05 | 0.715 |
| ENSP00000262948 | MAP2K2 | 1.43E-05 | 0.431 |
| ENSP00000274576 | GLRA1 | 1.43E-05 | 0.169 |
| ENSP00000281081 | NUBPL | 1.43E-05 | 0.213 |
| ENSP00000348298 | LCOR | 1.43E-05 | 0.163 |
| ENSP00000362592 | RBBP4 | 1.42E-05 | 0.698 |
| ENSP00000268957 | TOB1 | 1.42E-05 | 0.26 |
| ENSP00000362576 | YARS | 1.42E-05 | 0.747 |
| ENSP00000225916 | KAT2A | 1.42E-05 | 0.549 |
| ENSP00000306606 | ADH1B | 1.42E-05 | 0.457 |
| ENSP00000359629 | CRTAC1 | 1.42E-05 | 0.099 |
| ENSP00000314414 | AP2B1 | 1.42E-05 | 0.426 |
| ENSP00000309830 | RPL38 | 1.42E-05 | 0.559 |
| ENSP00000247866 | NDUFB2 | 1.42E-05 | 0.483 |
| ENSP00000258052 | SMPD2 | 1.42E-05 | 0.222 |
| ENSP00000251642 | DHX58 | 1.42E-05 | 0.3 |
| ENSP00000368646 | PRDX4 | 1.42E-05 | 0.548 |
| ENSP00000294800 | FCGR3B | 1.42E-05 | 0.245 |
| ENSP00000371432 | PRLR | 1.42E-05 | 0.181 |
| ENSP00000356350 | PCMT1 | 1.42E-05 | 0.488 |
| ENSP00000320239 | TRPM7 | 1.42E-05 | 0.228 |
| ENSP00000386935 | UAP1L1 | 1.42E-05 | 0.045 |
| ENSP00000262241 | RCOR1 | 1.41E-05 | 0.229 |
| ENSP00000296513 | ADAD1 | 1.41E-05 | 0.141 |
| ENSP00000262320 | AXIN1 | 1.41E-05 | 0.811 |
| ENSP00000296684 | NDUFS4 | 1.41E-05 | 0.439 |
| ENSP00000228740 | LTA4H | 1.41E-05 | 0.161 |
| ENSP00000357668 | ADAM12 | 1.41E-05 | 0.141 |
| ENSP00000346537 | SMOC2 | 1.41E-05 | 0.485 |
| ENSP00000317997 | DUOX1 | 1.41E-05 | 0.032 |
| ENSP00000311873 | EXO1 | 1.41E-05 | 0.875 |
| ENSP00000256997 | ACP2 | 1.41E-05 | 0.053 |
| ENSP00000329466 | SIRT7 | 1.41E-05 | 0.72 |
| ENSP00000333982 | NDEL1 | 1.41E-05 | 0.416 |
| ENSP00000307369 | SHE | 1.41E-05 | 0.121 |
| ENSP00000303997 | GTPBP2 | 1.41E-05 | 0.168 |
| ENSP00000378635 | DRP2 | 1.41E-05 | 0.361 |
| ENSP00000306275 | KCNK3 | 1.41E-05 | 0.142 |
| ENSP00000380414 | DEK | 1.41E-05 | 0.772 |
| ENSP00000280187 | GPM6A | 1.41E-05 | 0.119 |
| ENSP00000264128 | SLC25A24 | 1.41E-05 | 0.11 |
| ENSP00000363944 | LAS1L | 1.41E-05 | 0.435 |
| ENSP00000294507 | LAPTM5 | 1.40E-05 | 0.498 |
| ENSP00000222399 | LAMB1 | 1.40E-05 | 0.214 |
| ENSP00000217429 | FAM83D | 1.40E-05 | 0.287 |
| ENSP00000340983 | LRRC25 | 1.40E-05 | 0.286 |
| ENSP00000316879 | EIF4G1 | 1.40E-05 | 0.794 |
| ENSP00000409605 | TRIM15 | 1.40E-05 | 0.14 |
| ENSP00000334330 | DEFB105A | 1.40E-05 | 0.191 |
| ENSP00000221847 | EBI3 | 1.40E-05 | 0.089 |
| ENSP00000395772 | GNL3 | 1.40E-05 | 0.881 |
| ENSP00000394494 | IFNA13 | 1.40E-05 | 0.16 |
| ENSP00000310301 | SP3 | 1.40E-05 | 0.32 |
| ENSP00000394863 | PPT1 | 1.40E-05 | 0.287 |
| ENSP00000259469 | RPL35 | 1.40E-05 | 0.781 |
| ENSP00000401397 | DDR1 | 1.40E-05 | 0.243 |
| ENSP00000233969 | SLC9A2 | 1.40E-05 | 0.113 |
| ENSP00000215574 | CDC34 | 1.40E-05 | 0.518 |
| ENSP00000374354 | EXOSC8 | 1.40E-05 | 0.767 |
| ENSP00000342343 | MED22 | 1.40E-05 | 0.2 |
| ENSP00000249750 | ALDH1A2 | 1.40E-05 | 0.411 |
| ENSP00000366482 | FXN | 1.40E-05 | 0.321 |
| ENSP00000273857 | CORIN | 1.40E-05 | 0.075 |
| ENSP00000348283 | WWP2 | 1.40E-05 | 0.188 |
| ENSP00000330737 | NDUFA12 | 1.40E-05 | 0.573 |
| ENSP00000301411 | NTF4 | 1.40E-05 | 0.126 |
| ENSP00000287598 | BUB1B | 1.40E-05 | 0.933 |
| ENSP00000268802 | NOB1 | 1.40E-05 | 0.645 |
| ENSP00000356919 | ATF6 | 1.40E-05 | 0.429 |
| ENSP00000402608 | CPS1 | 1.40E-05 | 0.772 |
| ENSP00000331066 | OLIG1 | 1.40E-05 | 0.196 |
| ENSP00000364277 | FGD1 | 1.40E-05 | 0.527 |
| ENSP00000389184 | MARK2 | 1.39E-05 | 0.328 |
| ENSP00000010338 | TRAF3IP3 | 1.39E-05 | 0.445 |
| ENSP00000361066 | NCOA3 | 1.39E-05 | 0.537 |
| ENSP00000364188 | DDOST | 1.39E-05 | 0.608 |
| ENSP00000358857 | EMD | 1.39E-05 | 0.68 |
| ENSP00000412673 | GABRR1 | 1.39E-05 | 0.121 |
| ENSP00000276431 | TNFRSF10B | 1.39E-05 | 0.234 |
| ENSP00000348944 | RSPO1 | 1.39E-05 | 0.2 |
| ENSP00000299328 | TAZ | 1.39E-05 | 0.216 |
| ENSP00000376848 | PRIMA1 | 1.39E-05 | 0.175 |
| ENSP00000351777 | VCP | 1.39E-05 | 0.753 |
| ENSP00000322396 | RRS1 | 1.39E-05 | 0.788 |
| ENSP00000349687 | GM2A | 1.39E-05 | 0.169 |
| ENSP00000342554 | STX2 | 1.39E-05 | 0.322 |
| ENSP00000263377 | BRD4 | 1.39E-05 | 0.201 |
| ENSP00000355751 | THBS2 | 1.39E-05 | 0.436 |
| ENSP00000262971 | PIAS4 | 1.38E-05 | 0.236 |
| ENSP00000376823 | C7orf49 | 1.38E-05 | 0.118 |
| ENSP00000355890 | EPRS | 1.38E-05 | 0.828 |
| ENSP00000386134 | NGDN | 1.38E-05 | 0.632 |
| ENSP00000391498 | KCNK16 | 1.38E-05 | 0.107 |
| ENSP00000244296 | PSG1 | 1.38E-05 | 0.063 |
| ENSP00000319531 | GCSH | 1.38E-05 | 0.541 |
| ENSP00000369460 | ELAVL2 | 1.38E-05 | 0.255 |
| ENSP00000347834 | FBXL3 | 1.38E-05 | 0.293 |
| ENSP00000403447 | NOTCH4 | 1.38E-05 | 0.362 |
| ENSP00000319231 | PHLDA2 | 1.38E-05 | 0.176 |
| ENSP00000296946 | T | 1.38E-05 | 0.272 |
| ENSP00000304689 | THAP11 | 1.38E-05 | 0.106 |
| ENSP00000328364 | MAFA | 1.38E-05 | 0.09 |
| ENSP00000302441 | NANP | 1.38E-05 | 0.046 |
| ENSP00000356448 | TPR | 1.38E-05 | 0.812 |
| ENSP00000342144 | EVC2 | 1.38E-05 | 0.223 |
| ENSP00000265717 | PRKAR2B | 1.38E-05 | 0.439 |
| ENSP00000344223 | MBTPS1 | 1.37E-05 | 0.141 |
| ENSP00000349629 | LRBA | 1.37E-05 | 0.18 |
| ENSP00000283646 | RPIA | 1.37E-05 | 0.342 |
| ENSP00000395653 | SLC46A1 | 1.37E-05 | 0.203 |
| ENSP00000317310 | CABP1 | 1.37E-05 | 0.064 |
| ENSP00000398224 | TRIM15 | 1.37E-05 | 0.121 |
| ENSP00000405963 | RASGRF1 | 1.37E-05 | 0.33 |
| ENSP00000406037 | KAT8 | 1.37E-05 | 0.206 |
| ENSP00000352839 | NPSR1 | 1.37E-05 | 0.674 |
| ENSP00000384442 | CDK11A | 1.37E-05 | 0.265 |
| ENSP00000223641 | SEC61B | 1.37E-05 | 0.453 |
| ENSP00000402338 | RPF2 | 1.37E-05 | 0.888 |
| ENSP00000298767 | WAPAL | 1.37E-05 | 0.389 |
| ENSP00000226193 | RCVRN | 1.37E-05 | 0.383 |
| ENSP00000247207 | HSPA2 | 1.37E-05 | 0.375 |
| ENSP00000380378 | PAFAH1B1 | 1.37E-05 | 0.759 |
| ENSP00000054950 | RCN1 | 1.37E-05 | 0.4 |
| ENSP00000402060 | PVR | 1.37E-05 | 0.22 |
| ENSP00000357555 | RPS27 | 1.37E-05 | 0.4 |
| ENSP00000376776 | DBH | 1.37E-05 | 0.194 |
| ENSP00000418082 | RPL37A | 1.36E-05 | 0.61 |
| ENSP00000414187 | CPEB1 | 1.36E-05 | 0.667 |
| ENSP00000365884 | TRIM15 | 1.36E-05 | 0.103 |
| ENSP00000300456 | SLC27A4 | 1.36E-05 | 0.31 |
| ENSP00000332455 | KPNA2 | 1.36E-05 | 0.876 |
| ENSP00000315465 | DSCAML1 | 1.36E-05 | 0.211 |
| ENSP00000411683 | TRIM15 | 1.36E-05 | 0.1 |
| ENSP00000311816 | REST | 1.36E-05 | 0.431 |
| ENSP00000356000 | PLXNA2 | 1.36E-05 | 0.282 |
| ENSP00000356001 | MRPL18 | 1.36E-05 | 0.545 |
| ENSP00000338864 | RASGRP2 | 1.36E-05 | 0.32 |
| ENSP00000379823 | COL4A3 | 1.36E-05 | 0.357 |
| ENSP00000250111 | ATP1B2 | 1.36E-05 | 0.197 |
| ENSP00000294517 | ADC | 1.36E-05 | 0.12 |
| ENSP00000231749 | ZMYND10 | 1.36E-05 | 0.344 |
| ENSP00000219599 | CRYM | 1.36E-05 | 0.246 |
| ENSP00000348722 | PUS7 | 1.36E-05 | 0.563 |
| ENSP00000229335 | AICDA | 1.36E-05 | 0.437 |
| ENSP00000364660 | C2 | 1.36E-05 | 0.109 |
| ENSP00000349393 | LIG4 | 1.36E-05 | 0.49 |
| ENSP00000344844 | HAVCR1 | 1.36E-05 | 0.113 |
| ENSP00000249504 | HOXD11 | 1.35E-05 | 0.248 |
| ENSP00000239243 | MSX2 | 1.35E-05 | 0.514 |
| ENSP00000229214 | KRR1 | 1.35E-05 | 0.907 |
| ENSP00000360268 | ALDH18A1 | 1.35E-05 | 0.49 |
| ENSP00000265441 | WNT2 | 1.35E-05 | 0.249 |
| ENSP00000408295 | RFC5 | 1.35E-05 | 0.911 |
| ENSP00000339479 | NT5C2 | 1.35E-05 | 0.479 |
| ENSP00000355955 | NENF | 1.35E-05 | 0.099 |
| ENSP00000326767 | MED25 | 1.35E-05 | 0.214 |
| ENSP00000282146 | KCNK13 | 1.35E-05 | 0.098 |
| ENSP00000229179 | NUP107 | 1.35E-05 | 0.899 |
| ENSP00000311876 | GNPDA1 | 1.35E-05 | 0.203 |
| ENSP00000361949 | PABPC4 | 1.35E-05 | 0.217 |
| ENSP00000283249 | ITGB6 | 1.35E-05 | 0.337 |
| ENSP00000308450 | CDC20 | 1.35E-05 | 0.959 |
| ENSP00000305692 | GAA | 1.35E-05 | 0.212 |
| ENSP00000265080 | RASGRF2 | 1.35E-05 | 0.406 |
| ENSP00000230510 | TTK | 1.35E-05 | 0.79 |
| ENSP00000221138 | PPP2CB | 1.35E-05 | 0.68 |
| ENSP00000278888 | MS4A2 | 1.35E-05 | 0.095 |
| ENSP00000265447 | ANXA11 | 1.35E-05 | 0.182 |
| ENSP00000360171 | TENM1 | 1.35E-05 | 0.722 |
| ENSP00000282050 | ATP5A1 | 1.34E-05 | 0.645 |
| ENSP00000229243 | ACRBP | 1.34E-05 | 0.313 |
| ENSP00000262643 | CCNE1 | 1.34E-05 | 0.768 |
| ENSP00000231572 | RARS | 1.34E-05 | 0.863 |
| ENSP00000362103 | POU3F1 | 1.34E-05 | 0.187 |
| ENSP00000301242 | PPP1R14A | 1.34E-05 | 0.208 |
| ENSP00000308753 | TMOD3 | 1.34E-05 | 0.081 |
| ENSP00000263382 | ASF1B | 1.34E-05 | 0.849 |
| ENSP00000294066 | MAP4K2 | 1.34E-05 | 0.077 |
| ENSP00000261523 | RORA | 1.34E-05 | 0.354 |
| ENSP00000294489 | PDPN | 1.34E-05 | 0.248 |
| ENSP00000366244 | ARAF | 1.34E-05 | 0.42 |
| ENSP00000338010 | IL21R | 1.34E-05 | 0.103 |
| ENSP00000388996 | AP1M1 | 1.34E-05 | 0.4 |
| ENSP00000254227 | NR0B2 | 1.34E-05 | 0.49 |
| ENSP00000264773 | KCNN2 | 1.34E-05 | 0.264 |
| ENSP00000362777 | ATOH7 | 1.34E-05 | 0.142 |
| ENSP00000237889 | NDUFB3 | 1.34E-05 | 0.517 |
| ENSP00000382004 | CTNND1 | 1.34E-05 | 0.657 |
| ENSP00000264669 | FASTKD3 | 1.34E-05 | 0.175 |
| ENSP00000357906 | TNFAIP8L2 | 1.34E-05 | 0.337 |
| ENSP00000355011 | ILF2 | 1.33E-05 | 0.898 |
| ENSP00000301732 | ABCA3 | 1.33E-05 | 0.363 |
| ENSP00000184266 | NDUFB4 | 1.33E-05 | 0.298 |
| ENSP00000262848 | PRKX | 1.33E-05 | 0.458 |
| ENSP00000370938 | CDK8 | 1.33E-05 | 0.597 |
| ENSP00000222573 | ITGB8 | 1.33E-05 | 0.339 |
| ENSP00000354718 | AKAP13 | 1.33E-05 | 0.224 |
| ENSP00000294338 | PDZK1IP1 | 1.33E-05 | 0.247 |
| ENSP00000323880 | FOXJ1 | 1.33E-05 | 0.308 |
| ENSP00000303706 | CDC25A | 1.33E-05 | 0.864 |
| ENSP00000262105 | MCM4 | 1.33E-05 | 0.923 |
| ENSP00000161559 | CEACAM1 | 1.33E-05 | 0.113 |
| ENSP00000261837 | GNB5 | 1.33E-05 | 0.272 |
| ENSP00000228841 | MYL2 | 1.33E-05 | 0.464 |
| ENSP00000263610 | BARHL1 | 1.33E-05 | 0.143 |
| ENSP00000368881 | KIN | 1.33E-05 | 0.087 |
| ENSP00000300873 | GNG8 | 1.33E-05 | 0.146 |
| ENSP00000360576 | NDOR1 | 1.33E-05 | 0.165 |
| ENSP00000255224 | SYT4 | 1.33E-05 | 0.242 |
| ENSP00000361151 | CEL | 1.33E-05 | 0.384 |
| ENSP00000360922 | STAU1 | 1.33E-05 | 0.282 |
| ENSP00000381412 | CAMK2A | 1.33E-05 | 0.442 |
| ENSP00000346694 | HNRNPA2B1 | 1.33E-05 | 0.772 |
| ENSP00000367196 | GLIPR2 | 1.33E-05 | 0.185 |
| ENSP00000357123 | MNDA | 1.33E-05 | 0.334 |
| ENSP00000330633 | CNTN2 | 1.32E-05 | 0.284 |
| ENSP00000345997 | DMPK | 1.32E-05 | 0.213 |
| ENSP00000317333 | NEUROG2 | 1.32E-05 | 0.329 |
| ENSP00000296181 | ITGB5 | 1.32E-05 | 0.449 |
| ENSP00000230323 | TFEB | 1.32E-05 | 0.171 |
| ENSP00000237612 | ABCG2 | 1.32E-05 | 0.663 |
| ENSP00000234396 | ATP6V1B1 | 1.32E-05 | 0.273 |
| ENSP00000319974 | BBX | 1.32E-05 | 0.109 |
| ENSP00000299198 | CKB | 1.32E-05 | 0.219 |
| ENSP00000258381 | SP110 | 1.32E-05 | 0.39 |
| ENSP00000256594 | GSTM3 | 1.32E-05 | 0.252 |
| ENSP00000367446 | EXT1 | 1.32E-05 | 0.335 |
| ENSP00000299502 | SERPINB2 | 1.32E-05 | 0.143 |
| ENSP00000312709 | TAF7 | 1.32E-05 | 0.562 |
| ENSP00000252825 | HRC | 1.32E-05 | 0.294 |
| ENSP00000305152 | PLP1 | 1.32E-05 | 0.357 |
| ENSP00000273153 | CSRNP1 | 1.32E-05 | 0.124 |
| ENSP00000242872 | CENPK | 1.32E-05 | 0.737 |
| ENSP00000301071 | TUBA1A | 1.32E-05 | 0.643 |
| ENSP00000355325 | PSMB5 | 1.32E-05 | 0.853 |
| ENSP00000345809 | ZNF197 | 1.32E-05 | 0.014 |
| ENSP00000280097 | HNMT | 1.32E-05 | 0.257 |
| ENSP00000379607 | KNCN | 1.31E-05 | 0.831 |
| ENSP00000284245 | C16orf74 | 1.31E-05 | 0.006 |
| ENSP00000333938 | NEXN | 1.31E-05 | 0.17 |
| ENSP00000229854 | MCM3 | 1.31E-05 | 0.919 |
| ENSP00000355566 | TOMM20 | 1.31E-05 | 0.839 |
| ENSP00000375035 | IGHV5-51 | 1.31E-05 | 0.178 |
| ENSP00000303942 | GP9 | 1.31E-05 | 0.107 |
| ENSP00000360021 | GNG12 | 1.31E-05 | 0.19 |
| ENSP00000331698 | KCNJ15 | 1.31E-05 | 0.134 |
| ENSP00000261458 | HHAT | 1.31E-05 | 0.137 |
| ENSP00000248150 | GNG13 | 1.31E-05 | 0.223 |
| ENSP00000364784 | C6orf48 | 1.31E-05 | 0.005 |
| ENSP00000338157 | ZBTB16 | 1.31E-05 | 0.414 |
| ENSP00000332716 | CCDC28A | 1.31E-05 | 0.069 |
| ENSP00000301761 | SDHAF2 | 1.31E-05 | 0.195 |
| ENSP00000367214 | GRXCR2 | 1.31E-05 | 0.052 |
| ENSP00000309087 | PLAC8L1 | 1.31E-05 | 0.056 |
| ENSP00000386759 | SETD2 | 1.31E-05 | 0.718 |
| ENSP00000314193 | WDR75 | 1.31E-05 | 0.852 |
| ENSP00000265140 | ANKRD32 | 1.31E-05 | 0.246 |
| ENSP00000329093 | TPH2 | 1.31E-05 | 0.131 |
| ENSP00000246792 | RRAS | 1.31E-05 | 0.561 |
| ENSP00000265304 | SSBP1 | 1.31E-05 | 0.508 |
| ENSP00000311837 | SNTG2 | 1.31E-05 | 0.198 |
| ENSP00000381340 | GGT5 | 1.30E-05 | 0.316 |
| ENSP00000264670 | NSUN2 | 1.30E-05 | 0.377 |
| ENSP00000370373 | FKBP1B | 1.30E-05 | 0.391 |
| ENSP00000308227 | HMGA1 | 1.30E-05 | 0.195 |
| ENSP00000240328 | TBX2 | 1.30E-05 | 0.212 |
| ENSP00000389305 | GOLGA6L4 | 1.30E-05 | 0.054 |
| ENSP00000378856 | LGALS9 | 1.30E-05 | 0.136 |
| ENSP00000262306 | TCEB2 | 1.30E-05 | 0.52 |
| ENSP00000357440 | HSF2 | 1.30E-05 | 0.226 |
| ENSP00000044462 | PSMA4 | 1.30E-05 | 0.97 |
| ENSP00000325425 | MAT2B | 1.30E-05 | 0.142 |
| ENSP00000354560 | KIFAP3 | 1.30E-05 | 0.444 |
| ENSP00000346440 | TCF4 | 1.30E-05 | 0.379 |
| ENSP00000376765 | PIAS3 | 1.30E-05 | 0.275 |
| ENSP00000346810 | PLAGL1 | 1.30E-05 | 0.17 |
| ENSP00000339428 | SOCS2 | 1.30E-05 | 0.295 |
| ENSP00000363018 | RPL10A | 1.30E-05 | 0.585 |
| ENSP00000216218 | ST13 | 1.29E-05 | 0.272 |
| ENSP00000222644 | MPP6 | 1.29E-05 | 0.374 |
| ENSP00000246914 | WNK4 | 1.29E-05 | 0.227 |
| ENSP00000408910 | DCTN2 | 1.29E-05 | 0.603 |
| ENSP00000293525 | KRT86 | 1.29E-05 | 0.128 |
| ENSP00000256897 | CCNH | 1.29E-05 | 0.667 |
| ENSP00000265462 | PRDX5 | 1.29E-05 | 0.316 |
| ENSP00000364037 | TEX10 | 1.29E-05 | 0.338 |
| ENSP00000304736 | ELOVL6 | 1.29E-05 | 0.565 |
| ENSP00000319096 | RAP2B | 1.29E-05 | 0.439 |
| ENSP00000349803 | ZBTB20 | 1.29E-05 | 0.127 |
| ENSP00000263525 | TNR | 1.29E-05 | 0.185 |
| ENSP00000369237 | SLC6A2 | 1.29E-05 | 0.619 |
| ENSP00000395535 | MECP2 | 1.29E-05 | 0.922 |
| ENSP00000349320 | CACNA2D1 | 1.29E-05 | 0.244 |
| ENSP00000346886 | GABPA | 1.29E-05 | 0.288 |
| ENSP00000310170 | FOSL1 | 1.29E-05 | 0.402 |
| ENSP00000283006 | CENPH | 1.29E-05 | 0.742 |
| ENSP00000187397 | ARPP21 | 1.29E-05 | 0.524 |
| ENSP00000353475 | CLDN7 | 1.29E-05 | 0.441 |
| ENSP00000264553 | GZMM | 1.29E-05 | 0.194 |
| ENSP00000259457 | PSMB7 | 1.29E-05 | 0.593 |
| ENSP00000308774 | BMX | 1.28E-05 | 0.061 |
| ENSP00000350199 | AP1B1 | 1.28E-05 | 0.359 |
| ENSP00000343392 | XRCC3 | 1.28E-05 | 0.453 |
| ENSP00000345001 | CRTC1 | 1.28E-05 | 0.259 |
| ENSP00000309504 | SLC26A7 | 1.28E-05 | 0.195 |
| ENSP00000311117 | DBI | 1.28E-05 | 0.168 |
| ENSP00000354876 | MT-CO2 | 1.28E-05 | 0.432 |
| ENSP00000264663 | NNT | 1.28E-05 | 0.407 |
| ENSP00000346921 | AK2 | 1.28E-05 | 0.438 |
| ENSP00000353654 | COL4A2 | 1.28E-05 | 0.334 |
| ENSP00000297689 | NFIL3 | 1.28E-05 | 0.294 |
| ENSP00000340200 | GLYAT | 1.28E-05 | 0.23 |
| ENSP00000298316 | ARF6 | 1.28E-05 | 0.723 |
| ENSP00000320604 | FAXDC2 | 1.28E-05 | 0.708 |
| ENSP00000265734 | CDK6 | 1.28E-05 | 0.655 |
| ENSP00000238788 | TMEM214 | 1.28E-05 | 0.304 |
| ENSP00000269122 | CLTC | 1.28E-05 | 0.822 |
| ENSP00000361867 | SEMG1 | 1.28E-05 | 0.166 |
| ENSP00000257829 | NAT10 | 1.28E-05 | 0.846 |
| ENSP00000284984 | ADAMTS1 | 1.28E-05 | 0.229 |
| ENSP00000278407 | SERPING1 | 1.28E-05 | 0.478 |
| ENSP00000365256 | CLCN5 | 1.28E-05 | 0.456 |
| ENSP00000325690 | CARM1 | 1.28E-05 | 0.738 |
| ENSP00000300737 | STIM1 | 1.28E-05 | 0.159 |
| ENSP00000410715 | SFRP4 | 1.28E-05 | 0.518 |
| ENSP00000167218 | PDCD2 | 1.28E-05 | 0.283 |
| ENSP00000371594 | GNG7 | 1.28E-05 | 0.283 |
| ENSP00000346196 | NDUFV3 | 1.28E-05 | 0.196 |
| ENSP00000366347 | NKX2-2 | 1.28E-05 | 0.448 |
| ENSP00000203629 | LAG3 | 1.28E-05 | 0.058 |
| ENSP00000367623 | HSPA14 | 1.28E-05 | 0.418 |
| ENSP00000250244 | AP1M2 | 1.27E-05 | 0.369 |
| ENSP00000376250 | CCDC50 | 1.27E-05 | 0.148 |
| ENSP00000366682 | IRG1 | 1.27E-05 | 0.14 |
| ENSP00000012443 | PPP5C | 1.27E-05 | 0.224 |
| ENSP00000261183 | OSBPL8 | 1.27E-05 | 0.487 |
| ENSP00000251287 | HCN2 | 1.27E-05 | 0.21 |
| ENSP00000359675 | GNG5 | 1.27E-05 | 0.2 |
| ENSP00000340507 | TRIM24 | 1.27E-05 | 0.361 |
| ENSP00000344609 | BTG3 | 1.27E-05 | 0.323 |
| ENSP00000393393 | RPL35A | 1.27E-05 | 0.631 |
| ENSP00000354525 | MRPL24 | 1.27E-05 | 0.65 |
| ENSP00000226021 | CACNG1 | 1.27E-05 | 0.37 |
| ENSP00000321260 | COX8A | 1.27E-05 | 0.361 |
| ENSP00000310006 | NR1D2 | 1.27E-05 | 0.34 |
| ENSP00000372313 | MSLN | 1.27E-05 | 0.123 |
| ENSP00000246166 | FNTB | 1.27E-05 | 0.184 |
| ENSP00000322524 | DCTPP1 | 1.27E-05 | 0.551 |
| ENSP00000385720 | ARHGAP35 | 1.27E-05 | 0.332 |
| ENSP00000300413 | SNRPD1 | 1.27E-05 | 0.951 |
| ENSP00000260361 | NDUFAF1 | 1.27E-05 | 0.273 |
| ENSP00000390600 | SCN10A | 1.27E-05 | 0.182 |
| ENSP00000248901 | CYTH4 | 1.27E-05 | 0.422 |
| ENSP00000264187 | NID1 | 1.27E-05 | 0.425 |
| ENSP00000265631 | SLC25A13 | 1.27E-05 | 0.123 |
| ENSP00000295066 | DPY30 | 1.26E-05 | 0.855 |
| ENSP00000411177 | MRPL22 | 1.26E-05 | 0.761 |
| ENSP00000344537 | TAF6 | 1.26E-05 | 0.392 |
| ENSP00000363768 | C1QC | 1.26E-05 | 0.535 |
| ENSP00000417899 | MECOM | 1.26E-05 | 0.377 |
| ENSP00000414906 | DCTN4 | 1.26E-05 | 0.182 |
| ENSP00000357040 | VANGL2 | 1.26E-05 | 0.393 |
| ENSP00000217901 | IDH3G | 1.26E-05 | 0.514 |
| ENSP00000320543 | EPN2 | 1.26E-05 | 0.249 |
| ENSP00000301740 | SRRM2 | 1.26E-05 | 0.501 |
| ENSP00000272348 | SNRPG | 1.26E-05 | 0.938 |
| ENSP00000332353 | PTCH1 | 1.26E-05 | 0.833 |
| ENSP00000337736 | AKAP1 | 1.26E-05 | 0.285 |
| ENSP00000356480 | RNF2 | 1.26E-05 | 0.541 |
| ENSP00000256509 | CHL1 | 1.26E-05 | 0.157 |
| ENSP00000357461 | CHRNB2 | 1.26E-05 | 0.327 |
| ENSP00000321988 | SULT1A1 | 1.26E-05 | 0.164 |
| ENSP00000264674 | MECOM | 1.26E-05 | 0.402 |
| ENSP00000369299 | TRIM22 | 1.26E-05 | 0.44 |
| ENSP00000355556 | GNG4 | 1.26E-05 | 0.18 |
| ENSP00000296417 | H2AFZ | 1.26E-05 | 0.992 |
| ENSP00000381648 | PIAS2 | 1.26E-05 | 0.369 |
| ENSP00000288207 | CCNB2 | 1.26E-05 | 0.851 |
| ENSP00000312299 | FRG2C | 1.26E-05 | 0.019 |
| ENSP00000384730 | FRG2C | 1.26E-05 | 0.019 |
| ENSP00000344155 | ABCA2 | 1.25E-05 | 0.109 |
| ENSP00000308901 | MRPL45 | 1.25E-05 | 0.219 |
| ENSP00000262776 | LGALS3BP | 1.25E-05 | 0.247 |
| ENSP00000408697 | NUGGC | 1.25E-05 | 0.005 |
| ENSP00000358497 | RNGTT | 1.25E-05 | 0.598 |
| ENSP00000353246 | MAK16 | 1.25E-05 | 0.832 |
| ENSP00000243776 | CHPF | 1.25E-05 | 0.403 |
| ENSP00000352257 | XRCC6 | 1.25E-05 | 0.77 |
| ENSP00000287022 | UQCRB | 1.25E-05 | 0.379 |
| ENSP00000355296 | LDB3 | 1.25E-05 | 0.548 |
| ENSP00000358105 | APH1A | 1.25E-05 | 0.224 |
| ENSP00000345445 | SAMM50 | 1.25E-05 | 0.435 |
| ENSP00000380460 | PLAA | 1.25E-05 | 0.443 |
| ENSP00000260404 | PAK6 | 1.25E-05 | 0.253 |
| ENSP00000255688 | RARRES3 | 1.25E-05 | 0.227 |
| ENSP00000311962 | GGA2 | 1.25E-05 | 0.359 |
| ENSP00000313169 | NPHP1 | 1.25E-05 | 0.41 |
| ENSP00000321805 | RIT2 | 1.25E-05 | 0.137 |
| ENSP00000292644 | PSMC2 | 1.25E-05 | 0.967 |
| ENSP00000369176 | NDUFB6 | 1.25E-05 | 0.401 |
| ENSP00000005587 | SKAP2 | 1.25E-05 | 0.123 |
| ENSP00000353094 | SDF4 | 1.25E-05 | 0.315 |
| ENSP00000331057 | TCF12 | 1.25E-05 | 0.472 |
| ENSP00000361626 | YBX1 | 1.25E-05 | 0.71 |
| ENSP00000290810 | NAE1 | 1.24E-05 | 0.923 |
| ENSP00000216271 | HDAC10 | 1.24E-05 | 0.203 |
| ENSP00000290765 | GSTT2B | 1.24E-05 | 0.267 |
| ENSP00000348827 | THRB | 1.24E-05 | 0.486 |
| ENSP00000352336 | PLCG2 | 1.24E-05 | 0.594 |
| ENSP00000232458 | ECT2 | 1.24E-05 | 0.723 |
| ENSP00000344874 | GUCY1A2 | 1.24E-05 | 0.246 |
| ENSP00000317904 | GYS1 | 1.24E-05 | 0.299 |
| ENSP00000347754 | HTR3A | 1.24E-05 | 0.361 |
| ENSP00000358737 | ATP5F1 | 1.24E-05 | 0.807 |
| ENSP00000387261 | PSTPIP2 | 1.24E-05 | 0.083 |
| ENSP00000321070 | ME2 | 1.24E-05 | 0.285 |
| ENSP00000345096 | IMPDH1 | 1.24E-05 | 0.423 |
| ENSP00000234310 | PPP3R1 | 1.24E-05 | 0.456 |
| ENSP00000252711 | NDUFA10 | 1.24E-05 | 0.321 |
| ENSP00000272369 | MEIS1 | 1.24E-05 | 0.443 |
| ENSP00000302476 | HSPB2 | 1.24E-05 | 0.368 |
| ENSP00000230582 | PRSS16 | 1.24E-05 | 0.17 |
| ENSP00000261921 | LOXL1 | 1.24E-05 | 0.34 |
| ENSP00000363411 | GNG10 | 1.24E-05 | 0.196 |
| ENSP00000254193 | SNRPA1 | 1.24E-05 | 0.92 |
| ENSP00000301634 | TK1 | 1.24E-05 | 0.779 |
| ENSP00000233084 | DDX1 | 1.24E-05 | 0.841 |
| ENSP00000343741 | ATR | 1.23E-05 | 0.788 |
| ENSP00000304290 | CHRNB1 | 1.23E-05 | 0.148 |
| ENSP00000390475 | CXXC1 | 1.23E-05 | 0.329 |
| ENSP00000355046 | MT-ND2 | 1.23E-05 | 0.184 |
| ENSP00000365877 | SUV39H1 | 1.23E-05 | 0.813 |
| ENSP00000349508 | CHD4 | 1.23E-05 | 0.501 |
| ENSP00000339621 | LCN6 | 1.23E-05 | 0.184 |
| ENSP00000259253 | UGGT1 | 1.23E-05 | 0.158 |
| ENSP00000181839 | CDK13 | 1.23E-05 | 0.333 |
| ENSP00000377470 | CNP | 1.23E-05 | 0.071 |
| ENSP00000362329 | PPA1 | 1.23E-05 | 0.814 |
| ENSP00000376849 | CASP5 | 1.23E-05 | 0.207 |
| ENSP00000333157 | GH2 | 1.23E-05 | 0.051 |
| ENSP00000286548 | GNAQ | 1.23E-05 | 0.633 |
| ENSP00000366410 | NMNAT1 | 1.23E-05 | 0.342 |
| ENSP00000397552 | ACTL6A | 1.23E-05 | 0.951 |
| ENSP00000357905 | DMBT1 | 1.23E-05 | 0.241 |
| ENSP00000221496 | AMH | 1.22E-05 | 0.343 |
| ENSP00000316054 | DVL3 | 1.22E-05 | 0.546 |
| ENSP00000227880 | SLC15A3 | 1.22E-05 | 0.615 |
| ENSP00000420714 | MRPS14 | 1.22E-05 | 0.487 |
| ENSP00000410758 | LSM5 | 1.22E-05 | 0.849 |
| ENSP00000408005 | SLC9A3R2 | 1.22E-05 | 0.367 |
| ENSP00000366898 | HIF3A | 1.22E-05 | 0.108 |
| ENSP00000215727 | SERPIND1 | 1.22E-05 | 0.502 |
| ENSP00000384169 | FBLN2 | 1.22E-05 | 0.201 |
| ENSP00000368927 | EIF1AX | 1.22E-05 | 0.785 |
| ENSP00000365528 | PTPRH | 1.22E-05 | 0.246 |
| ENSP00000270233 | BCAM | 1.22E-05 | 0.249 |
| ENSP00000354119 | LAT | 1.22E-05 | 0.177 |
| ENSP00000300175 | SCG5 | 1.22E-05 | 0.321 |
| ENSP00000215071 | PSMD8 | 1.22E-05 | 0.869 |
| ENSP00000361818 | SDC4 | 1.22E-05 | 0.408 |
| ENSP00000349640 | MCRS1 | 1.21E-05 | 0.339 |
| ENSP00000331614 | IKZF1 | 1.21E-05 | 0.28 |
| ENSP00000386104 | CPE | 1.21E-05 | 0.296 |
| ENSP00000371471 | RSAD2 | 1.21E-05 | 0.424 |
| ENSP00000236979 | TNP1 | 1.21E-05 | 0.433 |
| ENSP00000388566 | CASP4 | 1.21E-05 | 0.174 |
| ENSP00000407779 | NRM | 1.21E-05 | 0.267 |
| ENSP00000335392 | SPATA12 | 1.21E-05 | 0.023 |
| ENSP00000367309 | MAOB | 1.21E-05 | 0.369 |
| ENSP00000373539 | STAB2 | 1.21E-05 | 0.12 |
| ENSP00000355601 | EGLN1 | 1.21E-05 | 0.234 |
| ENSP00000230340 | BYSL | 1.21E-05 | 0.911 |
| ENSP00000383330 | KCNJ6 | 1.21E-05 | 0.233 |
| ENSP00000256103 | PMP2 | 1.21E-05 | 0.103 |
| ENSP00000363284 | POLE3 | 1.21E-05 | 0.34 |
| ENSP00000260049 | IL18BP | 1.21E-05 | 0.078 |
| ENSP00000359793 | PTGFR | 1.21E-05 | 0.33 |
| ENSP00000359489 | AFF2 | 1.21E-05 | 0.562 |
| ENSP00000367198 | LECT1 | 1.21E-05 | 0.156 |
| ENSP00000265388 | TNPO3 | 1.21E-05 | 0.24 |
| ENSP00000256104 | FABP4 | 1.21E-05 | 0.507 |
| ENSP00000336762 | ANG | 1.21E-05 | 0.13 |
| ENSP00000355721 | SNAP47 | 1.21E-05 | 0.151 |
| ENSP00000303522 | TACR1 | 1.20E-05 | 0.427 |
| ENSP00000261881 | TIPIN | 1.20E-05 | 0.827 |
| ENSP00000385361 | VPREB1 | 1.20E-05 | 0.251 |
| ENSP00000215095 | STX1B | 1.20E-05 | 0.229 |
| ENSP00000348108 | KHDRBS3 | 1.20E-05 | 0.501 |
| ENSP00000363377 | FOXO4 | 1.20E-05 | 0.504 |
| ENSP00000320295 | TUBB3 | 1.20E-05 | 0.55 |
| ENSP00000199764 | CEACAM6 | 1.20E-05 | 0.238 |
| ENSP00000360412 | NOC3L | 1.20E-05 | 0.945 |
| ENSP00000311344 | PPP2R1B | 1.20E-05 | 0.407 |
| ENSP00000377303 | RENBP | 1.20E-05 | 0.315 |
| ENSP00000259953 | NRM | 1.20E-05 | 0.227 |
| ENSP00000367498 | LRRC47 | 1.20E-05 | 0.161 |
| ENSP00000259365 | TMOD1 | 1.20E-05 | 0.254 |
| ENSP00000221801 | FBL | 1.20E-05 | 0.927 |
| ENSP00000328181 | NOG | 1.20E-05 | 0.702 |
| ENSP00000354206 | ZNF219 | 1.20E-05 | 0.159 |
| ENSP00000340736 | GYG1 | 1.20E-05 | 0.254 |
| ENSP00000236051 | EBNA1BP2 | 1.20E-05 | 0.818 |
| ENSP00000354643 | DIO1 | 1.20E-05 | 0.113 |
| ENSP00000025008 | RB1CC1 | 1.20E-05 | 0.806 |
| ENSP00000389894 | NRM | 1.20E-05 | 0.348 |
| ENSP00000251372 | LILRA1 | 1.20E-05 | 0.085 |
| ENSP00000351650 | NUDT3 | 1.20E-05 | 0.13 |
| ENSP00000335675 | PRELID2 | 1.20E-05 | 0.211 |
| ENSP00000276925 | CDKN2B | 1.20E-05 | 0.384 |
| ENSP00000362153 | GNL2 | 1.19E-05 | 0.858 |
| ENSP00000353910 | FUT8 | 1.19E-05 | 0.2 |
| ENSP00000304051 | RNF139 | 1.19E-05 | 0.227 |
| ENSP00000295542 | DCST1 | 1.19E-05 | 0.151 |
| ENSP00000253362 | BPIFA2 | 1.19E-05 | 0.202 |
| ENSP00000401435 | VPS53 | 1.19E-05 | 0.321 |
| ENSP00000348099 | PDLIM7 | 1.19E-05 | 0.401 |
| ENSP00000296161 | DTX3L | 1.19E-05 | 0.326 |
| ENSP00000356331 | NR5A2 | 1.19E-05 | 0.36 |
| ENSP00000262710 | ACIN1 | 1.19E-05 | 0.222 |
| ENSP00000319897 | FAM214B | 1.19E-05 | 0.154 |
| ENSP00000292599 | MAML1 | 1.19E-05 | 0.175 |
| ENSP00000298999 | R3HCC1L | 1.19E-05 | 0.057 |
| ENSP00000300026 | PPIB | 1.19E-05 | 0.528 |
| ENSP00000340989 | SFN | 1.19E-05 | 0.471 |
| ENSP00000222247 | RPL18A | 1.19E-05 | 0.793 |
| ENSP00000281031 | NDUFC2 | 1.19E-05 | 0.329 |
| ENSP00000249396 | SIRT2 | 1.19E-05 | 0.678 |
| ENSP00000299798 | SLC9A5 | 1.19E-05 | 0.229 |
| ENSP00000409285 | NRM | 1.19E-05 | 0.294 |
| ENSP00000397755 | NRM | 1.19E-05 | 0.292 |
| ENSP00000304791 | KLK7 | 1.19E-05 | 0.321 |
| ENSP00000363559 | EIF6 | 1.19E-05 | 0.873 |
| ENSP00000351409 | CELF1 | 1.18E-05 | 0.243 |
| ENSP00000246662 | KRT9 | 1.18E-05 | 0.195 |
| ENSP00000318916 | MARCO | 1.18E-05 | 0.402 |
| ENSP00000300738 | RRM1 | 1.18E-05 | 0.957 |
| ENSP00000261173 | ATP2B1 | 1.18E-05 | 0.296 |
| ENSP00000296490 | WDR82 | 1.18E-05 | 0.456 |
| ENSP00000352273 | DIO3 | 1.18E-05 | 0.189 |
| ENSP00000321929 | NME9 | 1.18E-05 | 0.291 |
| ENSP00000265276 | GPAM | 1.18E-05 | 0.312 |
| ENSP00000405812 | SLC4A8 | 1.18E-05 | 0.172 |
| ENSP00000333275 | NR2C1 | 1.18E-05 | 0.257 |
| ENSP00000341625 | CYB5A | 1.18E-05 | 0.408 |
| ENSP00000347839 | RAB11FIP2 | 1.18E-05 | 0.245 |
| ENSP00000333934 | TRMT11 | 1.18E-05 | 0.773 |
| ENSP00000397956 | IER3 | 1.18E-05 | 0.199 |
| ENSP00000373509 | C11orf31 | 1.18E-05 | 0.297 |
| ENSP00000266041 | ITIH4 | 1.18E-05 | 0.371 |
| ENSP00000302935 | IL16 | 1.18E-05 | 0.137 |
| ENSP00000349547 | RASSF1 | 1.18E-05 | 0.581 |
| ENSP00000284727 | ATP5G3 | 1.18E-05 | 0.664 |
| ENSP00000339186 | GRAP2 | 1.18E-05 | 0.244 |
| ENSP00000262457 | INVS | 1.18E-05 | 0.358 |
| ENSP00000228682 | GLI1 | 1.18E-05 | 0.706 |
| ENSP00000357748 | BCCIP | 1.18E-05 | 0.921 |
| ENSP00000335357 | CDKN3 | 1.18E-05 | 0.837 |
| ENSP00000356395 | CFHR3 | 1.18E-05 | 0.156 |
| ENSP00000369349 | KRT81 | 1.18E-05 | 0.124 |
| ENSP00000304707 | SLN | 1.17E-05 | 0.105 |
| ENSP00000234798 | TPSG1 | 1.17E-05 | 0.251 |
| ENSP00000291232 | TNFRSF13C | 1.17E-05 | 0.157 |
| ENSP00000372326 | FECH | 1.17E-05 | 0.525 |
| ENSP00000317376 | MRPS11 | 1.17E-05 | 0.595 |
| ENSP00000280979 | AKAP6 | 1.17E-05 | 0.273 |
| ENSP00000370201 | TAF9 | 1.17E-05 | 0.828 |
| ENSP00000318158 | MRPS24 | 1.17E-05 | 0.547 |
| ENSP00000290536 | M1AP | 1.17E-05 | 0.172 |
| ENSP00000324549 | CYFIP1 | 1.17E-05 | 0.408 |
| ENSP00000412045 | TXNRD1 | 1.17E-05 | 0.379 |
| ENSP00000380727 | TMEM213 | 1.17E-05 | 0.025 |
| ENSP00000216185 | TXN2 | 1.17E-05 | 0.43 |
| ENSP00000356540 | STX11 | 1.17E-05 | 0.207 |
| ENSP00000377640 | RPL24 | 1.17E-05 | 0.829 |
| ENSP00000288602 | BRAF | 1.17E-05 | 0.338 |
| ENSP00000357858 | BUB3 | 1.17E-05 | 0.988 |
| ENSP00000377978 | CCL4L2 | 1.17E-05 | 0.12 |
| ENSP00000310129 | PSMD2 | 1.17E-05 | 0.753 |
| ENSP00000370968 | MYBBP1A | 1.17E-05 | 0.631 |
| ENSP00000369566 | IFNA10 | 1.17E-05 | 0.075 |
| ENSP00000352097 | SPTSSB | 1.17E-05 | 0.233 |
| ENSP00000323264 | MARVELD2 | 1.17E-05 | 0.278 |
| ENSP00000363124 | PHF20 | 1.17E-05 | 0.3 |
| ENSP00000215591 | POLRMT | 1.16E-05 | 0.401 |
| ENSP00000357823 | MRPL9 | 1.16E-05 | 0.37 |
| ENSP00000286479 | NAT2 | 1.16E-05 | 0.164 |
| ENSP00000321584 | IMPDH2 | 1.16E-05 | 0.729 |
| ENSP00000246081 | OTOR | 1.16E-05 | 0.013 |
| ENSP00000358188 | NBPF15 | 1.16E-05 | 0.031 |
| ENSP00000284320 | TOMM70A | 1.16E-05 | 0.576 |
| ENSP00000263735 | EPCAM | 1.16E-05 | 0.33 |
| ENSP00000356382 | F13B | 1.16E-05 | 0.294 |
| ENSP00000260433 | CYP19A1 | 1.16E-05 | 0.525 |
| ENSP00000358106 | PREP | 1.16E-05 | 0.203 |
| ENSP00000317087 | 8-Mar | 1.16E-05 | 0.132 |
| ENSP00000362410 | MAFB | 1.16E-05 | 0.444 |
| ENSP00000351767 | COL20A1 | 1.16E-05 | 0.287 |
| ENSP00000353581 | HIST1H3E | 1.16E-05 | 0.286 |
| ENSP00000295927 | PTX3 | 1.16E-05 | 0.124 |
| ENSP00000381129 | GRK4 | 1.16E-05 | 0.107 |
| ENSP00000352565 | HAND2 | 1.16E-05 | 0.344 |
| ENSP00000310701 | TEAD2 | 1.16E-05 | 0.245 |
| ENSP00000242719 | RNF11 | 1.16E-05 | 0.191 |
| ENSP00000279247 | CAPN1 | 1.16E-05 | 0.416 |
| ENSP00000377148 | AP1G1 | 1.16E-05 | 0.421 |
| ENSP00000326581 | PEG3 | 1.16E-05 | 0.221 |
| ENSP00000222725 | LFNG | 1.16E-05 | 0.37 |
| ENSP00000260363 | KIF23 | 1.16E-05 | 0.919 |
| ENSP00000366453 | TJP2 | 1.16E-05 | 0.379 |
| ENSP00000264260 | IL18RAP | 1.16E-05 | 0.131 |
| ENSP00000262854 | HUWE1 | 1.16E-05 | 0.663 |
| ENSP00000272418 | MRPS5 | 1.16E-05 | 0.201 |
| ENSP00000368124 | CAMK1D | 1.15E-05 | 0.201 |
| ENSP00000242776 | DDX39A | 1.15E-05 | 0.742 |
| ENSP00000338343 | SGCD | 1.15E-05 | 0.318 |
| ENSP00000311888 | MGAT1 | 1.15E-05 | 0.264 |
| ENSP00000370936 | E2F6 | 1.15E-05 | 0.272 |
| ENSP00000246062 | MKKS | 1.15E-05 | 0.55 |
| ENSP00000310405 | XCR1 | 1.15E-05 | 0.421 |
| ENSP00000416110 | RPS18 | 1.15E-05 | 0.502 |
| ENSP00000253303 | RGN | 1.15E-05 | 0.509 |
| ENSP00000235382 | RGS2 | 1.15E-05 | 0.572 |
| ENSP00000240617 | PLBD1 | 1.15E-05 | 0.2 |
| ENSP00000371483 | SLC34A2 | 1.15E-05 | 0.219 |
| ENSP00000262487 | ISM1 | 1.15E-05 | 0.252 |
| ENSP00000232424 | HES1 | 1.15E-05 | 0.219 |
| ENSP00000320246 | SLC9A9 | 1.15E-05 | 0.283 |
| ENSP00000303153 | COL22A1 | 1.15E-05 | 0.285 |
| ENSP00000238688 | SLIRP | 1.15E-05 | 0.731 |
| ENSP00000377833 | ANXA4 | 1.15E-05 | 0.204 |
| ENSP00000310440 | CHMP2A | 1.15E-05 | 0.39 |
| ENSP00000359393 | HMGB3 | 1.15E-05 | 0.412 |
| ENSP00000304006 | PAFAH1B2 | 1.15E-05 | 0.127 |
| ENSP00000262053 | ATF1 | 1.15E-05 | 0.419 |
| ENSP00000274376 | RASA1 | 1.15E-05 | 0.851 |
| ENSP00000324897 | UBE2I | 1.15E-05 | 0.934 |
| ENSP00000258962 | SRSF1 | 1.15E-05 | 0.973 |
| ENSP00000305138 | FAM195A | 1.15E-05 | 0.134 |
| ENSP00000372169 | CACNA2D4 | 1.14E-05 | 0.099 |
| ENSP00000252456 | CNN1 | 1.14E-05 | 0.263 |
| ENSP00000353154 | NFASC | 1.14E-05 | 0.28 |
| ENSP00000283147 | BMP6 | 1.14E-05 | 0.348 |
| ENSP00000346027 | RPL21 | 1.14E-05 | 0.363 |
| ENSP00000359074 | L1CAM | 1.14E-05 | 0.534 |
| ENSP00000257290 | PDGFRA | 1.14E-05 | 0.604 |
| ENSP00000347408 | AP3M1 | 1.14E-05 | 0.202 |
| ENSP00000317780 | COX5A | 1.14E-05 | 0.537 |
| ENSP00000296805 | GFM2 | 1.14E-05 | 0.3 |
| ENSP00000235310 | MAD2L2 | 1.14E-05 | 0.333 |
| ENSP00000412394 | MAML2 | 1.14E-05 | 0.167 |
| ENSP00000260058 | CREBZF | 1.14E-05 | 0.095 |
| ENSP00000265500 | NDUFC1 | 1.14E-05 | 0.302 |
| ENSP00000396452 | HDHD1 | 1.14E-05 | 0.337 |
| ENSP00000348564 | CDAN1 | 1.14E-05 | 0.185 |
| ENSP00000257745 | MLL5 | 1.14E-05 | 0.529 |
| ENSP00000272542 | SLC20A1 | 1.14E-05 | 0.301 |
| ENSP00000312664 | CASP2 | 1.14E-05 | 0.278 |
| ENSP00000380444 | MYO9B | 1.14E-05 | 0.341 |
| ENSP00000313391 | DAB2 | 1.14E-05 | 0.347 |
| ENSP00000242257 | FTSJ2 | 1.14E-05 | 0.341 |
| ENSP00000332111 | EFCAB13 | 1.14E-05 | 0.061 |
| ENSP00000256343 | CATSPERB | 1.13E-05 | 0.133 |
| ENSP00000358696 | PLXNA3 | 1.13E-05 | 0.391 |
| ENSP00000275815 | EPHA1 | 1.13E-05 | 0.166 |
| ENSP00000359024 | NOLC1 | 1.13E-05 | 0.807 |
| ENSP00000347041 | FMOD | 1.13E-05 | 0.645 |
| ENSP00000314004 | ANAPC2 | 1.13E-05 | 0.325 |
| ENSP00000258494 | ALDH1L2 | 1.13E-05 | 0.246 |
| ENSP00000349957 | ANAPC11 | 1.13E-05 | 0.562 |
| ENSP00000005178 | PDK4 | 1.13E-05 | 0.225 |
| ENSP00000322898 | EBF1 | 1.13E-05 | 0.565 |
| ENSP00000223862 | RLN1 | 1.13E-05 | 0.079 |
| ENSP00000219905 | MGA | 1.13E-05 | 0.08 |
| ENSP00000007722 | ITGA3 | 1.13E-05 | 0.476 |
| ENSP00000255380 | CHRM3 | 1.13E-05 | 0.524 |
| ENSP00000363031 | THEMIS2 | 1.13E-05 | 0.47 |
| ENSP00000250454 | EAPP | 1.13E-05 | 0.236 |
| ENSP00000355206 | MT-ND3 | 1.13E-05 | 0.14 |
| ENSP00000247005 | GDF1 | 1.13E-05 | 0.24 |
| ENSP00000341885 | RPS2 | 1.13E-05 | 0.503 |
| ENSP00000364902 | POFUT1 | 1.13E-05 | 0.309 |
| ENSP00000369558 | IFNA6 | 1.12E-05 | 0.067 |
| ENSP00000377770 | NDUFC1 | 1.12E-05 | 0.272 |
| ENSP00000010299 | FAM76A | 1.12E-05 | 0.11 |
| ENSP00000315112 | RNF31 | 1.12E-05 | 0.135 |
| ENSP00000308024 | PCSK1 | 1.12E-05 | 0.364 |
| ENSP00000370223 | IDH3B | 1.12E-05 | 0.583 |
| ENSP00000332247 | ATP6V0A2 | 1.12E-05 | 0.383 |
| ENSP00000310568 | KCNK10 | 1.12E-05 | 0.213 |
| ENSP00000410994 | BRD2 | 1.12E-05 | 0.235 |
| ENSP00000301364 | TSR1 | 1.12E-05 | 0.931 |
| ENSP00000339004 | FOXG1 | 1.12E-05 | 0.366 |
| ENSP00000084795 | RPL18 | 1.12E-05 | 0.77 |
| ENSP00000340684 | MAOA | 1.12E-05 | 0.416 |
| ENSP00000380156 | RPL32 | 1.12E-05 | 0.396 |
| ENSP00000246151 | PITHD1 | 1.12E-05 | 0.065 |
| ENSP00000245912 | TNFSF14 | 1.12E-05 | 0.222 |
| ENSP00000330787 | NDUFB1 | 1.12E-05 | 0.212 |
| ENSP00000351632 | FAM3D | 1.12E-05 | 0.248 |
| ENSP00000370395 | SGTB | 1.12E-05 | 0.171 |
| ENSP00000300688 | ATP5L | 1.12E-05 | 0.791 |
| ENSP00000398736 | CYR61 | 1.12E-05 | 0.344 |
| ENSP00000381717 | UBE2D2 | 1.12E-05 | 0.72 |
| ENSP00000331201 | HGS | 1.12E-05 | 0.763 |
| ENSP00000231498 | NUP155 | 1.12E-05 | 0.791 |
| ENSP00000304803 | NKRF | 1.12E-05 | 0.396 |
| ENSP00000353739 | PCMTD1 | 1.12E-05 | 0.334 |
| ENSP00000258424 | COX5B | 1.12E-05 | 0.505 |
| ENSP00000245157 | BBS2 | 1.12E-05 | 0.419 |
| ENSP00000304891 | ATP6V1E2 | 1.12E-05 | 0.335 |
| ENSP00000373404 | MRPL2 | 1.12E-05 | 0.622 |
| ENSP00000013807 | ERCC1 | 1.12E-05 | 0.565 |
| ENSP00000411397 | MARK3 | 1.12E-05 | 0.29 |
| ENSP00000324804 | PPP2R1A | 1.11E-05 | 0.904 |
| ENSP00000268122 | RHCG | 1.11E-05 | 0.236 |
| ENSP00000282441 | YAP1 | 1.11E-05 | 0.505 |
| ENSP00000295974 | APBB2 | 1.11E-05 | 0.115 |
| ENSP00000304102 | COPS6 | 1.11E-05 | 0.775 |
| ENSP00000370588 | CD99 | 1.11E-05 | 0.24 |
| ENSP00000360730 | IL13RA1 | 1.11E-05 | 0.457 |
| ENSP00000333802 | ZNF599 | 1.11E-05 | 0.022 |
| ENSP00000355001 | POU3F3 | 1.11E-05 | 0.236 |
| ENSP00000370316 | GPR143 | 1.11E-05 | 0.305 |
| ENSP00000317619 | LACC1 | 1.11E-05 | 0.279 |
| ENSP00000331504 | FES | 1.11E-05 | 0.283 |
| ENSP00000309690 | DMXL1 | 1.11E-05 | 0.645 |
| ENSP00000315476 | EXOSC4 | 1.11E-05 | 0.615 |
| ENSP00000291525 | TFF3 | 1.11E-05 | 0.254 |
| ENSP00000238044 | C2orf40 | 1.11E-05 | 0.189 |
| ENSP00000355775 | MIXL1 | 1.11E-05 | 0.129 |
| ENSP00000344220 | PDPK1 | 1.10E-05 | 0.244 |
| ENSP00000233331 | INO80B | 1.10E-05 | 0.128 |
| ENSP00000005284 | CACNG3 | 1.10E-05 | 0.34 |
| ENSP00000281923 | MGAT5 | 1.10E-05 | 0.538 |
| ENSP00000335382 | DEFB128 | 1.10E-05 | 0.159 |
| ENSP00000354340 | UBA3 | 1.10E-05 | 0.841 |
| ENSP00000293422 | MYL6 | 1.10E-05 | 0.425 |
| ENSP00000371040 | RLN2 | 1.10E-05 | 0.059 |
| ENSP00000268314 | ARMC5 | 1.10E-05 | 0.099 |
| ENSP00000375682 | KLK8 | 1.10E-05 | 0.135 |
| ENSP00000338272 | HEY1 | 1.10E-05 | 0.363 |
| ENSP00000356379 | ASPM | 1.10E-05 | 0.909 |
| ENSP00000414110 | ZNRD1 | 1.10E-05 | 0.063 |
| ENSP00000296412 | ADH5 | 1.10E-05 | 0.674 |
| ENSP00000258960 | NMT1 | 1.10E-05 | 0.279 |
| ENSP00000316357 | USP9X | 1.10E-05 | 0.628 |
| ENSP00000318822 | BID | 1.10E-05 | 0.183 |
| ENSP00000390131 | NCR3 | 1.10E-05 | 0.315 |
| ENSP00000337065 | CXCL14 | 1.10E-05 | 0.218 |
| ENSP00000053468 | MRPS10 | 1.10E-05 | 0.316 |
| ENSP00000299752 | CDH16 | 1.10E-05 | 0.175 |
| ENSP00000325863 | MRE11A | 1.10E-05 | 0.881 |
| ENSP00000274545 | GABRA6 | 1.10E-05 | 0.19 |
| ENSP00000363500 | CLIC4 | 1.10E-05 | 0.135 |
| ENSP00000348657 | NCAPG2 | 1.10E-05 | 0.855 |
| ENSP00000379108 | MTHFD2L | 1.10E-05 | 0.306 |
| ENSP00000372023 | CHEK2 | 1.10E-05 | 0.949 |
| ENSP00000364864 | KIF3B | 1.10E-05 | 0.49 |
| ENSP00000299314 | GNPTAB | 1.09E-05 | 0.216 |
| ENSP00000255189 | DMGDH | 1.09E-05 | 0.357 |
| ENSP00000418823 | FTO | 1.09E-05 | 0.364 |
| ENSP00000401371 | TIA1 | 1.09E-05 | 0.91 |
| ENSP00000333779 | EVI2B | 1.09E-05 | 0.369 |
| ENSP00000262419 | KANSL1 | 1.09E-05 | 0.267 |
| ENSP00000250699 | CHRNA10 | 1.09E-05 | 0.087 |
| ENSP00000296802 | NSA2 | 1.09E-05 | 0.91 |
| ENSP00000354478 | DLX1 | 1.09E-05 | 0.424 |
| ENSP00000236957 | EEF1B2 | 1.09E-05 | 0.89 |
| ENSP00000348089 | ERCC6 | 1.09E-05 | 0.454 |
| ENSP00000336701 | RAD51C | 1.09E-05 | 0.844 |
| ENSP00000263817 | ABCB11 | 1.09E-05 | 0.508 |
| ENSP00000265651 | FBXO3 | 1.09E-05 | 0.215 |
| ENSP00000410396 | CMSS1 | 1.09E-05 | 0.846 |
| ENSP00000344259 | UBE2L3 | 1.09E-05 | 0.481 |
| ENSP00000390329 | CACNA2D2 | 1.09E-05 | 0.212 |
| ENSP00000320768 | RCAN1 | 1.09E-05 | 0.34 |
| ENSP00000287766 | SLC6A1 | 1.09E-05 | 0.696 |
| ENSP00000354665 | MT-ND6 | 1.09E-05 | 0.155 |
| ENSP00000362298 | SGPL1 | 1.09E-05 | 0.48 |
| ENSP00000222256 | RAB3A | 1.09E-05 | 0.454 |
| ENSP00000347581 | AGK | 1.09E-05 | 0.199 |
| ENSP00000260643 | PREB | 1.09E-05 | 0.498 |
| ENSP00000355899 | RRP15 | 1.09E-05 | 0.892 |
| ENSP00000319370 | KCNMB3 | 1.09E-05 | 0.203 |
| ENSP00000288422 | TAB3 | 1.09E-05 | 0.176 |
| ENSP00000369154 | SMAD9 | 1.09E-05 | 0.344 |
| ENSP00000221978 | NKG7 | 1.09E-05 | 0.29 |
| ENSP00000337838 | RTN4 | 1.08E-05 | 0.573 |
| ENSP00000411851 | C17orf49 | 1.08E-05 | 0.123 |
| ENSP00000340799 | SP6 | 1.08E-05 | 0.227 |
| ENSP00000305714 | BMP1 | 1.08E-05 | 0.451 |
| ENSP00000254488 | SLC6A11 | 1.08E-05 | 0.573 |
| ENSP00000367637 | MRS2 | 1.08E-05 | 0.582 |
| ENSP00000219789 | CDIPT | 1.08E-05 | 0.678 |
| ENSP00000252891 | NUMBL | 1.08E-05 | 0.257 |
| ENSP00000260443 | RSL24D1 | 1.08E-05 | 0.991 |
| ENSP00000391457 | INO80C | 1.08E-05 | 0.149 |
| ENSP00000262219 | ANXA13 | 1.08E-05 | 0.222 |
| ENSP00000355361 | CD47 | 1.08E-05 | 0.387 |
| ENSP00000265070 | GOLPH3 | 1.08E-05 | 0.835 |
| ENSP00000246895 | STATH | 1.08E-05 | 0.167 |
| ENSP00000370962 | GGT6 | 1.08E-05 | 0.319 |
| ENSP00000354006 | TRPM6 | 1.08E-05 | 0.181 |
| ENSP00000342156 | NCR3 | 1.08E-05 | 0.193 |
| ENSP00000340328 | NYX | 1.08E-05 | 0.302 |
| ENSP00000341117 | SDSL | 1.08E-05 | 0.114 |
| ENSP00000334115 | OR10T2 | 1.08E-05 | 0.421 |
| ENSP00000330054 | EEF1A1 | 1.08E-05 | 0.842 |
| ENSP00000323046 | EXOSC3 | 1.08E-05 | 0.363 |
| ENSP00000380598 | BLOC1S5 | 1.08E-05 | 0.152 |
| ENSP00000380349 | CAPN3 | 1.08E-05 | 0.349 |
| ENSP00000304185 | GTSF1 | 1.08E-05 | 0.445 |
| ENSP00000289448 | HMHB1 | 1.08E-05 | 0.089 |
| ENSP00000356602 | VTA1 | 1.08E-05 | 0.418 |
| ENSP00000286428 | VBP1 | 1.08E-05 | 0.973 |
| ENSP00000260116 | TTPA | 1.08E-05 | 0.122 |
| ENSP00000296674 | RPS23 | 1.07E-05 | 0.797 |
| ENSP00000348074 | CLEC9A | 1.07E-05 | 0.061 |
| ENSP00000356975 | ADAMTS4 | 1.07E-05 | 0.222 |
| ENSP00000354871 | PPEF1 | 1.07E-05 | 0.279 |
| ENSP00000261714 | BLMH | 1.07E-05 | 0.312 |
| ENSP00000265164 | CASP6 | 1.07E-05 | 0.317 |
| ENSP00000362688 | EIF3I | 1.07E-05 | 0.906 |
| ENSP00000305653 | LETM1 | 1.07E-05 | 0.215 |
| ENSP00000370767 | GP2 | 1.07E-05 | 0.244 |
| ENSP00000338387 | HLCS | 1.07E-05 | 0.396 |
| ENSP00000243326 | RIF1 | 1.07E-05 | 0.295 |
| ENSP00000262932 | CNPY4 | 1.07E-05 | 0.025 |
| ENSP00000341692 | DAP3 | 1.07E-05 | 0.11 |
| ENSP00000311127 | VEGFB | 1.07E-05 | 0.2 |
| ENSP00000330005 | RGMA | 1.07E-05 | 0.24 |
| ENSP00000301050 | CACNB3 | 1.07E-05 | 0.237 |
| ENSP00000246548 | UBA2 | 1.07E-05 | 0.963 |
| ENSP00000297578 | SLC25A32 | 1.07E-05 | 0.184 |
| ENSP00000307889 | RPL13 | 1.07E-05 | 0.698 |
| ENSP00000326227 | GANC | 1.07E-05 | 0.23 |
| ENSP00000352219 | BCS1L | 1.07E-05 | 0.563 |
| ENSP00000367408 | CASK | 1.06E-05 | 0.934 |
| ENSP00000267197 | SETD1B | 1.06E-05 | 0.287 |
| ENSP00000348163 | PLS3 | 1.06E-05 | 0.428 |
| ENSP00000367378 | GPR34 | 1.06E-05 | 0.118 |
| ENSP00000326342 | ELMOD2 | 1.06E-05 | 0.172 |
| ENSP00000328269 | HMG20B | 1.06E-05 | 0.068 |
| ENSP00000233954 | IL1RL1 | 1.06E-05 | 0.205 |
| ENSP00000265335 | RAD50 | 1.06E-05 | 0.864 |
| ENSP00000338964 | GGT7 | 1.06E-05 | 0.384 |
| ENSP00000308733 | GPR151 | 1.06E-05 | 0.196 |
| ENSP00000371278 | CDC37L1 | 1.06E-05 | 0.082 |
| ENSP00000420298 | CD200 | 1.06E-05 | 0.107 |
| ENSP00000329482 | CCSER1 | 1.06E-05 | 0.205 |
| ENSP00000362068 | TAF8 | 1.06E-05 | 0.358 |
| ENSP00000359783 | IFI44 | 1.06E-05 | 0.385 |
| ENSP00000408094 | CLIC1 | 1.06E-05 | 0.416 |
| ENSP00000285848 | OXA1L | 1.06E-05 | 0.276 |
| ENSP00000310088 | PTMS | 1.06E-05 | 0.156 |
| ENSP00000248996 | GNAZ | 1.06E-05 | 0.462 |
| ENSP00000365280 | ID1 | 1.06E-05 | 0.434 |
| ENSP00000249776 | KNSTRN | 1.06E-05 | 0.549 |
| ENSP00000317473 | RABGGTB | 1.06E-05 | 0.839 |
| ENSP00000274764 | HIST1H2BA | 1.05E-05 | 0.473 |
| ENSP00000376544 | CD79B | 1.05E-05 | 0.397 |
| ENSP00000312741 | CAMKK2 | 1.05E-05 | 0.226 |
| ENSP00000225426 | PSMB3 | 1.05E-05 | 0.819 |
| ENSP00000285900 | GRIA1 | 1.05E-05 | 0.6 |
| ENSP00000253792 | ACLY | 1.05E-05 | 0.695 |
| ENSP00000324292 | FBF1 | 1.05E-05 | 0.1 |
| ENSP00000405738 | ESRP1 | 1.05E-05 | 0.353 |
| ENSP00000346120 | DDX21 | 1.05E-05 | 0.928 |
| ENSP00000382688 | KDM5A | 1.05E-05 | 0.307 |
| ENSP00000366673 | CLN5 | 1.05E-05 | 0.111 |
| ENSP00000410910 | MUSTN1 | 1.05E-05 | 0.099 |
| ENSP00000414980 | TCF19 | 1.05E-05 | 0.05 |
| ENSP00000367570 | PHF11 | 1.05E-05 | 0.421 |
| ENSP00000339740 | CAMK2D | 1.05E-05 | 0.45 |
| ENSP00000355583 | MLK4 | 1.05E-05 | 0.085 |
| ENSP00000295736 | SLC4A7 | 1.05E-05 | 0.236 |
| ENSP00000325312 | METAP2 | 1.05E-05 | 0.927 |
| ENSP00000377914 | CIAPIN1 | 1.05E-05 | 0.145 |
| ENSP00000370330 | ERBB2IP | 1.05E-05 | 0.482 |
| ENSP00000252543 | RPL36 | 1.05E-05 | 0.731 |
| ENSP00000413845 | BRD2 | 1.05E-05 | 0.213 |
| ENSP00000382342 | ABCC1 | 1.05E-05 | 0.443 |
| ENSP00000354737 | ECI2 | 1.05E-05 | 0.304 |
| ENSP00000216832 | PNN | 1.05E-05 | 0.865 |
| ENSP00000281701 | NVL | 1.05E-05 | 0.512 |
| ENSP00000265560 | USP4 | 1.05E-05 | 0.378 |
| ENSP00000377865 | RPL23 | 1.05E-05 | 0.847 |
| ENSP00000204961 | EFNB1 | 1.04E-05 | 0.756 |
| ENSP00000012134 | HIVEP2 | 1.04E-05 | 0.168 |
| ENSP00000230050 | RPS12 | 1.04E-05 | 0.573 |
| ENSP00000261868 | EIF3J | 1.04E-05 | 0.861 |
| ENSP00000351669 | MANEA | 1.04E-05 | 0.228 |
| ENSP00000261991 | RPS6KA5 | 1.04E-05 | 0.278 |
| ENSP00000362353 | GLP1R | 1.04E-05 | 0.35 |
| ENSP00000379401 | ODAM | 1.04E-05 | 0.107 |
| ENSP00000252622 | LSM7 | 1.04E-05 | 0.782 |
| ENSP00000225792 | DDX5 | 1.04E-05 | 0.887 |
| ENSP00000366829 | RBM10 | 1.04E-05 | 0.721 |
| ENSP00000318176 | PRKRA | 1.04E-05 | 0.218 |
| ENSP00000261537 | MIB1 | 1.04E-05 | 0.23 |
| ENSP00000342793 | PLD1 | 1.04E-05 | 0.563 |
| ENSP00000414808 | PPP1R11 | 1.04E-05 | 0.031 |
| ENSP00000354739 | RPL12 | 1.04E-05 | 0.774 |
| ENSP00000287038 | RPL30 | 1.04E-05 | 0.823 |
| ENSP00000403393 | BRD2 | 1.04E-05 | 0.285 |
| ENSP00000320488 | NAP1L5 | 1.04E-05 | 0.222 |
| ENSP00000414321 | RPS24 | 1.04E-05 | 0.813 |
| ENSP00000388842 | POU5F1 | 1.04E-05 | 0.135 |
| ENSP00000357255 | BGLAP | 1.04E-05 | 0.464 |
| ENSP00000263620 | ARID3A | 1.03E-05 | 0.147 |
| ENSP00000378288 | MYLK3 | 1.03E-05 | 0.177 |
| ENSP00000387768 | HLA-DQA2 | 1.03E-05 | 0.251 |
| ENSP00000300403 | TPX2 | 1.03E-05 | 0.85 |
| ENSP00000171757 | P2RY10 | 1.03E-05 | 0.435 |
| ENSP00000280892 | EIF4E | 1.03E-05 | 0.871 |
| ENSP00000226413 | GNRHR | 1.03E-05 | 0.537 |
| ENSP00000251993 | KIAA0930 | 1.03E-05 | 0.241 |
| ENSP00000368798 | MBTPS2 | 1.03E-05 | 0.73 |
| ENSP00000378324 | DNAJA4 | 1.03E-05 | 0.306 |
| ENSP00000314776 | MBLAC2 | 1.03E-05 | 0.054 |
| ENSP00000251390 | LILRA3 | 1.03E-05 | 0.205 |
| ENSP00000241256 | GHSR | 1.03E-05 | 0.398 |
| ENSP00000286713 | STOM | 1.03E-05 | 0.186 |
| ENSP00000275874 | RAB19 | 1.03E-05 | 0.359 |
| ENSP00000338562 | STX3 | 1.03E-05 | 0.347 |
| ENSP00000357429 | FABP7 | 1.03E-05 | 0.258 |
| ENSP00000264824 | LYL1 | 1.03E-05 | 0.14 |
| ENSP00000357844 | SLC16A10 | 1.03E-05 | 0.281 |
| ENSP00000306138 | GRM5 | 1.03E-05 | 0.286 |
| ENSP00000218758 | ACP5 | 1.03E-05 | 0.456 |
| ENSP00000343126 | ARID3B | 1.03E-05 | 0.145 |
| ENSP00000352852 | MDK | 1.03E-05 | 0.477 |
| ENSP00000369634 | PRPF40B | 1.03E-05 | 0.211 |
| ENSP00000356015 | ACAT2 | 1.03E-05 | 0.5 |
| ENSP00000403628 | CCHCR1 | 1.03E-05 | 0.174 |
| ENSP00000328858 | PYCR1 | 1.03E-05 | 0.271 |
| ENSP00000256443 | CDK7 | 1.02E-05 | 0.696 |
| ENSP00000361943 | HEYL | 1.02E-05 | 0.168 |
| ENSP00000292535 | CUX1 | 1.02E-05 | 0.285 |
| ENSP00000371505 | CLEC6A | 1.02E-05 | 0.117 |
| ENSP00000365682 | TLE1 | 1.02E-05 | 0.494 |
| ENSP00000311528 | GPR162 | 1.02E-05 | 0.2 |
| ENSP00000358203 | NBPF20 | 1.02E-05 | 0.223 |
| ENSP00000229330 | HCFC2 | 1.02E-05 | 0.227 |
| ENSP00000349465 | PICK1 | 1.02E-05 | 0.435 |
| ENSP00000260619 | THUMPD2 | 1.02E-05 | 0.5 |
| ENSP00000287025 | MTERFD1 | 1.02E-05 | 0.748 |
| ENSP00000164024 | CELSR3 | 1.02E-05 | 0.315 |
| ENSP00000368831 | SNX2 | 1.02E-05 | 0.807 |
| ENSP00000339795 | RPL7 | 1.02E-05 | 0.703 |
| ENSP00000226209 | MYH3 | 1.02E-05 | 0.256 |
| ENSP00000353074 | HIST1H2BF | 1.02E-05 | 0.567 |
| ENSP00000361725 | ENDOG | 1.02E-05 | 0.378 |
| ENSP00000376472 | STT3A | 1.02E-05 | 0.391 |
| ENSP00000253339 | LATS1 | 1.02E-05 | 0.455 |
| ENSP00000338868 | PHF8 | 1.02E-05 | 0.811 |
| ENSP00000343463 | MAP3K2 | 1.02E-05 | 0.2 |
| ENSP00000321744 | HIST1H2BC | 1.02E-05 | 0.564 |
| ENSP00000314407 | CA8 | 1.02E-05 | 0.186 |
| ENSP00000290200 | IL10RB | 1.02E-05 | 0.255 |
| ENSP00000366460 | PLXDC2 | 1.02E-05 | 0.382 |
| ENSP00000263437 | NLRP2 | 1.02E-05 | 0.167 |
| ENSP00000359291 | ZNF185 | 1.02E-05 | 0.108 |
| ENSP00000346693 | ELOVL2 | 1.02E-05 | 0.347 |
| ENSP00000363055 | ZWINT | 1.01E-05 | 0.677 |
| ENSP00000334681 | DEFB107A | 1.01E-05 | 0.098 |
| ENSP00000327214 | ATP13A2 | 1.01E-05 | 0.435 |
| ENSP00000419038 | GFM1 | 1.01E-05 | 0.309 |
| ENSP00000217515 | TXNL1 | 1.01E-05 | 0.41 |
| ENSP00000265016 | BST1 | 1.01E-05 | 0.14 |
| ENSP00000265723 | ABCB4 | 1.01E-05 | 0.455 |
| ENSP00000360031 | RRP12 | 1.01E-05 | 0.695 |
| ENSP00000336741 | DHX15 | 1.01E-05 | 0.993 |
| ENSP00000368245 | CXorf21 | 1.01E-05 | 0.141 |
| ENSP00000337393 | UXT | 1.01E-05 | 0.653 |
| ENSP00000382193 | MYBPC3 | 1.01E-05 | 0.376 |
| ENSP00000254079 | PPP1R1B | 1.01E-05 | 0.353 |
| ENSP00000359693 | FKBP1C | 1.01E-05 | 0.346 |
| ENSP00000365441 | CACNA1F | 1.01E-05 | 0.566 |
| ENSP00000362105 | UTP11L | 1.01E-05 | 0.618 |
| ENSP00000327647 | CRADD | 1.01E-05 | 0.248 |
| ENSP00000265148 | CENPE | 1.01E-05 | 0.787 |
| ENSP00000302021 | MUC7 | 1.01E-05 | 0.324 |
| ENSP00000295743 | EOMES | 1.01E-05 | 0.37 |
| ENSP00000327468 | KLHDC8B | 1.01E-05 | 0.063 |
| ENSP00000374552 | RNF216 | 1.01E-05 | 0.113 |
| ENSP00000368189 | ARID3C | 1.01E-05 | 0.04 |
| ENSP00000304845 | UGT1A1 | 1.01E-05 | 0.486 |
| ENSP00000291565 | PDXK | 1.01E-05 | 0.198 |
| ENSP00000261980 | VSX2 | 1.01E-05 | 0.334 |
| ENSP00000317379 | GLS | 1.01E-05 | 0.375 |
| ENSP00000258787 | MYO1G | 1.01E-05 | 0.217 |
| ENSP00000274031 | SETD7 | 1.00E-05 | 0.223 |
| ENSP00000243082 | HOXC11 | 1.00E-05 | 0.14 |
| ENSP00000342026 | PRDX6 | 1.00E-05 | 0.281 |
| ENSP00000361141 | OPN4 | 1.00E-05 | 0.55 |
| ENSP00000279488 | DUSP6 | 1.00E-05 | 0.319 |
| ENSP00000264714 | PPM1G | 1.00E-05 | 0.734 |
| ENSP00000298472 | SLC18A2 | 1.00E-05 | 0.323 |
| ENSP00000362255 | TSPO2 | 1.00E-05 | 0.055 |
| ENSP00000349490 | MFNG | 1.00E-05 | 0.141 |
| ENSP00000262551 | OGN | 1.00E-05 | 0.371 |
| ENSP00000367893 | SEPHS1 | 1.00E-05 | 0.336 |
| ENSP00000380855 | PDCD1LG2 | 1.00E-05 | 0.054 |
| ENSP00000352834 | MYO1C | 1.00E-05 | 0.507 |
| ENSP00000216259 | PMM1 | 1.00E-05 | 0.266 |
| ENSP00000322608 | GAS7 | 1.00E-05 | 0.326 |
| ENSP00000303129 | VAT1L | 1.00E-05 | 0.115 |
| ENSP00000263918 | STRN | 1.00E-05 | 0.201 |
| ENSP00000264051 | NGEF | 1.00E-05 | 0.21 |
| ENSP00000251772 | PLXNA1 | 1.00E-05 | 0.266 |
| ENSP00000311083 | CKS1B | 1.00E-05 | 0.827 |
