# Supplementary material for: A network-based method using a random walk with restart algorithm and screening tests to identify novel genes associated with Menière's disease
Source: PLoS One. 2017 Aug 7;12(8):e0182592. doi: 10.1371/journal.pone.0182592 (PMC5546581; doi:10.1371/journal.pone.0182592)
Supplement: S3 Table — (DOCX) [file pone.0182592.s003.docx]

**S3 Table.** The 1,069 candidate genes with permutation FDRs less than 0.05.

| **Ensembl ID** | **Gene symbol** | **Probability** | **P-value** | **MIS** | **MFS** |
| --- | --- | --- | --- | --- | --- |
| ENSP00000292303 | CCR5 | 2.01E-04 | <0.001 | 999 | 0.841 |
| ENSP00000332049 | CD86 | 2.00E-04 | <0.001 | 999 | 0.812 |
| ENSP00000240618 | KLRK1 | 8.61E-05 | <0.001 | 997 | 0.612 |
| ENSP00000365435 | TNFRSF1B | 1.79E-04 | <0.001 | 996 | 0.794 |
| ENSP00000365663 | NPPA | 2.05E-04 | <0.001 | 994 | 0.707 |
| ENSP00000295440 | NPPC | 8.63E-05 | <0.001 | 994 | 0.502 |
| ENSP00000375608 | KIR3DL1 | 1.45E-04 | <0.001 | 988 | 0.542 |
| ENSP00000270474 | PDE4A | 1.29E-04 | <0.001 | 982 | 0.679 |
| ENSP00000264832 | ICAM1 | 2.49E-04 | <0.001 | 977 | 0.835 |
| ENSP00000412237 | IL10 | 2.11E-04 | <0.001 | 976 | 0.851 |
| ENSP00000339393 | CCR6 | 1.37E-04 | <0.001 | 974 | 0.782 |
| ENSP00000347046 | PDE5A | 4.86E-05 | <0.001 | 973 | 0.529 |
| ENSP00000246657 | CCR7 | 1.25E-04 | <0.001 | 971 | 0.799 |
| ENSP00000343040 | HMGB1 | 1.13E-04 | <0.001 | 970 | 0.592 |
| ENSP00000361359 | CD40 | 2.35E-04 | <0.001 | 969 | 0.839 |
| ENSP00000294728 | VCAM1 | 1.75E-04 | <0.001 | 968 | 0.819 |
| ENSP00000331736 | SELE | 1.46E-04 | <0.001 | 967 | 0.766 |
| ENSP00000383645 | KIR3DS1 | 1.32E-04 | <0.001 | 966 | 0.572 |
| ENSP00000260010 | TLR2 | 1.80E-04 | <0.001 | 964 | 0.888 |
| ENSP00000340019 | HSPD1 | 1.12E-04 | <0.001 | 963 | 0.445 |
| ENSP00000292301 | CCR2 | 1.11E-04 | <0.001 | 962 | 0.765 |
| ENSP00000325525 | KIR3DL2 | 9.52E-05 | <0.001 | 962 | 0.436 |
| ENSP00000340011 | KIR2DS4 | 1.06E-04 | <0.001 | 961 | 0.439 |
| ENSP00000290866 | ACE | 1.99E-04 | <0.001 | 957 | 0.759 |
| ENSP00000375881 | ALPP | 2.27E-04 | <0.001 | 948 | 0.733 |
| ENSP00000295453 | ALPPL2 | 2.22E-04 | <0.001 | 948 | 0.732 |
| ENSP00000272190 | REN | 1.87E-04 | <0.001 | 947 | 0.769 |
| ENSP00000296545 | IL15 | 1.19E-04 | <0.001 | 935 | 0.767 |
| ENSP00000299855 | MMP3 | 7.59E-05 | <0.001 | 929 | 0.726 |
| ENSP00000372734 | HLA-DQB1 | 1.38E-04 | <0.001 | 928 | 0.770 |
| ENSP00000368450 | CD83 | 9.81E-05 | <0.001 | 927 | 0.761 |
| ENSP00000353874 | TLR9 | 8.65E-05 | <0.001 | 927 | 0.895 |
| ENSP00000261233 | IRAK3 | 7.70E-05 | <0.001 | 919 | 0.768 |
| ENSP00000329411 | IRF7 | 1.32E-04 | <0.001 | 917 | 0.806 |
| ENSP00000344192 | IL17A | 8.76E-05 | <0.001 | 913 | 0.790 |
| ENSP00000353452 | MYLK | 7.79E-05 | <0.001 | 911 | 0.629 |
| ENSP00000352064 | KLRC1 | 1.07E-04 | <0.001 | 907 | 0.501 |
| ENSP00000393355 | MICB | 6.41E-05 | <0.001 | 907 | 0.685 |
| ENSP00000351697 | REV3L | 1.95E-04 | <0.001 | 905 | 0.728 |
| ENSP00000332116 | PDE4B | 9.90E-05 | <0.001 | 904 | 0.298 |
| ENSP00000338130 | KLRD1 | 1.04E-04 | <0.001 | 902 | 0.569 |
| ENSP00000309124 | OLR1 | 8.06E-05 | <0.001 | 902 | 0.558 |
| ENSP00000339634 | KIR2DL4 | 8.87E-05 | <0.001 | 899 | 0.384 |
| ENSP00000216968 | PROCR | 8.59E-05 | <0.001 | 890 | 0.347 |
| ENSP00000369574 | IFNA21 | 7.92E-05 | <0.001 | 886 | 0.731 |
| ENSP00000411940 | IFNA17 | 8.37E-05 | <0.001 | 885 | 0.714 |
| ENSP00000412031 | HLA-DRB4 | 1.48E-04 | <0.001 | 852 | 0.000 |
| ENSP00000263686 | SELP | 1.63E-04 | <0.001 | 836 | 0.677 |
| ENSP00000328973 | TSPO | 3.61E-04 | <0.001 | 833 | 0.684 |
| ENSP00000259271 | GAD2 | 1.29E-04 | <0.001 | 824 | 0.365 |
| ENSP00000281830 | KCNE4 | 5.15E-05 | <0.001 | 778 | 0.677 |
| ENSP00000352312 | DNAH8 | 2.35E-04 | <0.001 | 752 | 0.609 |
| ENSP00000318770 | AQP11 | 1.30E-04 | <0.001 | 752 | 0.633 |
| ENSP00000262290 | LPO | 4.66E-05 | <0.001 | 671 | 0.432 |
| ENSP00000410046 | HLA-DRB4 | 1.32E-04 | <0.001 | 669 | 0.000 |
| ENSP00000397331 | HLA-G | 8.26E-05 | <0.001 | 653 | 0.647 |
| ENSP00000364444 | C4A | 1.16E-04 | <0.001 | 609 | 0.420 |
| ENSP00000200691 | MT3 | 8.22E-05 | <0.001 | 609 | 0.708 |
| ENSP00000368226 | GK | 8.48E-05 | <0.001 | 600 | 0.333 |
| ENSP00000388662 | C4A | 1.14E-04 | <0.001 | 567 | 0.420 |
| ENSP00000412786 | C4B_2 | 1.12E-04 | <0.001 | 565 | 0.437 |
| ENSP00000353472 | HLA-G | 9.20E-05 | <0.001 | 552 | 0.647 |
| ENSP00000356552 | MR1 | 6.06E-05 | <0.001 | 552 | 0.177 |
| ENSP00000387624 | HLA-G | 8.11E-05 | <0.001 | 551 | 0.647 |
| ENSP00000229708 | ULBP1 | 6.82E-05 | <0.001 | 543 | 0.557 |
| ENSP00000265715 | SLC26A4 | 1.31E-04 | <0.001 | 540 | 0.494 |
| ENSP00000298125 | WDFY2 | 1.04E-04 | <0.001 | 540 | 0.725 |
| ENSP00000263461 | WDR11 | 7.83E-05 | <0.001 | 540 | 0.216 |
| ENSP00000283558 | ATP11A | 7.23E-05 | <0.001 | 514 | 0.485 |
| ENSP00000375829 | LAD1 | 6.21E-05 | <0.001 | 497 | 0.267 |
| ENSP00000291890 | NCR1 | 7.79E-05 | <0.001 | 467 | 0.588 |
| ENSP00000296861 | TNFRSF21 | 8.93E-05 | <0.001 | 461 | 0.318 |
| ENSP00000416561 | CFB | 8.68E-05 | <0.001 | 434 | 0.513 |
| ENSP00000291582 | AIRE | 9.13E-05 | <0.001 | 430 | 0.390 |
| ENSP00000407561 | MICB | 6.30E-05 | <0.001 | 426 | 0.685 |
| ENSP00000349893 | DLK2 | 6.06E-05 | <0.001 | 407 | 0.232 |
| ENSP00000356329 | RAET1G | 4.67E-05 | <0.001 | 401 | 0.275 |
| ENSP00000395544 | NFKBIL1 | 6.35E-05 | <0.001 | 283 | 0.414 |
| ENSP00000394486 | NFKBIL1 | 6.33E-05 | <0.001 | 283 | 0.414 |
| ENSP00000408146 | HLA-DPB1 | 6.10E-05 | <0.001 | 272 | 0.696 |
| ENSP00000365318 | NFKBIL1 | 6.18E-05 | <0.001 | 269 | 0.414 |
| ENSP00000411611 | NFKBIL1 | 6.14E-05 | <0.001 | 269 | 0.414 |
| ENSP00000336769 | KIR2DL1 | 1.42E-04 | 0.001 | 999 | 0.552 |
| ENSP00000379625 | MYD88 | 1.37E-04 | 0.001 | 999 | 0.880 |
| ENSP00000228534 | IL23A | 1.29E-04 | 0.001 | 988 | 0.796 |
| ENSP00000315997 | LILRB1 | 8.95E-05 | 0.001 | 953 | 0.473 |
| ENSP00000348986 | INS-IGF2 | 4.00E-04 | 0.001 | 948 | 0.768 |
| ENSP00000216117 | HMOX1 | 8.43E-05 | 0.001 | 940 | 0.697 |
| ENSP00000253513 | IDO1 | 1.16E-04 | 0.001 | 926 | 0.550 |
| ENSP00000349770 | IRF5 | 1.14E-04 | 0.001 | 925 | 0.777 |
| ENSP00000364114 | HLA-DRB5 | 1.77E-04 | 0.001 | 923 | 0.822 |
| ENSP00000347379 | OCLN | 8.68E-05 | 0.001 | 920 | 0.520 |
| ENSP00000287497 | ITGAM | 1.03E-04 | 0.001 | 917 | 0.748 |
| ENSP00000229281 | C12orf57 | 9.31E-05 | 0.001 | 917 | 0.189 |
| ENSP00000359663 | CD40LG | 1.02E-04 | 0.001 | 915 | 0.784 |
| ENSP00000282561 | GJA1 | 1.72E-04 | 0.001 | 905 | 0.639 |
| ENSP00000408534 | TNFAIP8 | 4.75E-05 | 0.001 | 904 | 0.154 |
| ENSP00000332766 | NPB | 3.75E-05 | 0.001 | 899 | 0.691 |
| ENSP00000225474 | CSF3 | 1.13E-04 | 0.001 | 885 | 0.753 |
| ENSP00000416022 | HLA-DRB3 | 1.26E-04 | 0.001 | 750 | 0.000 |
| ENSP00000410857 | HLA-DRB4 | 1.20E-04 | 0.001 | 673 | 0.768 |
| ENSP00000337144 | AQP12A | 7.95E-05 | 0.001 | 671 | 0.552 |
| ENSP00000416509 | LTA | 8.28E-05 | 0.001 | 646 | 0.674 |
| ENSP00000354612 | PTGS1 | 7.53E-05 | 0.001 | 615 | 0.633 |
| ENSP00000397587 | HLA-DPA1 | 5.93E-05 | 0.001 | 613 | 0.363 |
| ENSP00000358501 | CD58 | 1.00E-04 | 0.001 | 563 | 0.701 |
| ENSP00000417404 | HFE | 8.86E-05 | 0.001 | 563 | 0.360 |
| ENSP00000286380 | RAET1L | 4.28E-05 | 0.001 | 495 | 0.127 |
| ENSP00000355812 | FGFR1OP | 6.23E-05 | 0.001 | 461 | 0.108 |
| ENSP00000407133 | LTA | 6.10E-05 | 0.001 | 420 | 0.674 |
| ENSP00000416337 | LTA | 6.10E-05 | 0.001 | 420 | 0.674 |
| ENSP00000349709 | RAET1E | 4.40E-05 | 0.001 | 417 | 0.264 |
| ENSP00000288709 | MMEL1 | 4.62E-05 | 0.001 | 377 | 0.408 |
| ENSP00000366729 | TNFRSF9 | 5.75E-05 | 0.001 | 308 | 0.469 |
| ENSP00000280357 | IL18 | 1.21E-04 | 0.002 | 994 | 0.852 |
| ENSP00000305651 | CXCL10 | 1.05E-04 | 0.002 | 994 | 0.891 |
| ENSP00000351671 | CCL20 | 1.19E-04 | 0.002 | 970 | 0.785 |
| ENSP00000278385 | CD44 | 1.56E-04 | 0.002 | 932 | 0.769 |
| ENSP00000265421 | POLB | 4.85E-05 | 0.002 | 923 | 0.311 |
| ENSP00000361850 | PLAU | 1.29E-04 | 0.002 | 922 | 0.686 |
| ENSP00000392398 | GPX5 | 8.36E-05 | 0.002 | 919 | 0.890 |
| ENSP00000259607 | CCL21 | 7.02E-05 | 0.002 | 910 | 0.748 |
| ENSP00000396486 | HSPA1L | 1.13E-04 | 0.002 | 905 | 0.411 |
| ENSP00000333639 | IFNL2 | 7.54E-05 | 0.002 | 806 | 0.402 |
| ENSP00000229239 | GAPDH | 2.07E-04 | 0.002 | 752 | 0.824 |
| ENSP00000263321 | TYR | 1.84E-04 | 0.002 | 615 | 0.714 |
| ENSP00000243347 | TNFAIP6 | 5.54E-05 | 0.002 | 517 | 0.300 |
| ENSP00000383142 | NFKBIL1 | 6.15E-05 | 0.002 | 269 | 0.414 |
| ENSP00000393566 | HLA-DPA1 | 4.35E-05 | 0.002 | 228 | 0.363 |
| ENSP00000342215 | KIR2DL3 | 1.46E-04 | 0.003 | 999 | 0.526 |
| ENSP00000011653 | CD4 | 3.11E-04 | 0.003 | 998 | 0.870 |
| ENSP00000359998 | GSTA4 | 9.13E-05 | 0.003 | 983 | 0.679 |
| ENSP00000318472 | NCAM1 | 1.41E-04 | 0.003 | 958 | 0.653 |
| ENSP00000369293 | IL2RA | 1.37E-04 | 0.003 | 946 | 0.802 |
| ENSP00000264708 | POMC | 1.33E-04 | 0.003 | 939 | 0.666 |
| ENSP00000316328 | CIITA | 1.34E-04 | 0.003 | 933 | 0.638 |
| ENSP00000273550 | FTH1 | 1.31E-04 | 0.003 | 902 | 0.673 |
| ENSP00000356694 | FASLG | 1.13E-04 | 0.003 | 902 | 0.762 |
| ENSP00000376886 | NME1-NME2 | 7.37E-05 | 0.003 | 901 | 0.336 |
| ENSP00000360973 | AGTR2 | 5.94E-05 | 0.003 | 899 | 0.589 |
| ENSP00000228434 | CD69 | 9.55E-05 | 0.003 | 877 | 0.774 |
| ENSP00000313437 | MMP19 | 3.33E-05 | 0.003 | 859 | 0.067 |
| ENSP00000372607 | HLA-DRB3 | 1.29E-04 | 0.003 | 853 | 0.000 |
| ENSP00000349588 | ANK2 | 4.89E-05 | 0.003 | 769 | 0.655 |
| ENSP00000367851 | CYBB | 7.34E-05 | 0.003 | 662 | 0.524 |
| ENSP00000269280 | NLRP1 | 8.49E-05 | 0.003 | 427 | 0.481 |
| ENSP00000356832 | SGK1 | 7.42E-05 | 0.003 | 421 | 0.472 |
| ENSP00000239223 | DUSP1 | 5.58E-05 | 0.003 | 407 | 0.490 |
| ENSP00000382422 | HLA-DPB1 | 7.62E-05 | 0.003 | 334 | 0.696 |
| ENSP00000372582 | HLA-DPB1 | 7.78E-05 | 0.003 | 333 | 0.696 |
| ENSP00000372912 | LY6G6F | 1.68E-05 | 0.003 | 210 | 0.099 |
| ENSP00000258743 | IL6 | 3.62E-04 | 0.004 | 992 | 0.920 |
| ENSP00000296871 | CSF2 | 2.05E-04 | 0.004 | 977 | 0.825 |
| ENSP00000369647 | AVP | 1.39E-04 | 0.004 | 968 | 0.547 |
| ENSP00000252723 | EPO | 1.08E-04 | 0.004 | 934 | 0.712 |
| ENSP00000388001 | OAS1 | 1.05E-04 | 0.004 | 923 | 0.556 |
| ENSP00000366307 | THBD | 6.05E-05 | 0.004 | 922 | 0.574 |
| ENSP00000226279 | CD38 | 9.30E-05 | 0.004 | 917 | 0.686 |
| ENSP00000252486 | APOE | 1.25E-04 | 0.004 | 905 | 0.740 |
| ENSP00000322570 | POLE | 1.82E-04 | 0.004 | 903 | 0.719 |
| ENSP00000330138 | SSTR3 | 5.63E-05 | 0.004 | 899 | 0.591 |
| ENSP00000306697 | MRAP | 9.72E-05 | 0.004 | 849 | 0.403 |
| ENSP00000276927 | IFNA1 | 1.34E-04 | 0.004 | 808 | 0.734 |
| ENSP00000319788 | NQO1 | 5.86E-05 | 0.004 | 766 | 0.498 |
| ENSP00000315477 | CD209 | 6.48E-05 | 0.004 | 693 | 0.724 |
| ENSP00000226760 | WFS1 | 5.77E-05 | 0.004 | 657 | 0.208 |
| ENSP00000397420 | HLA-E | 7.13E-05 | 0.004 | 552 | 0.707 |
| ENSP00000278070 | PPRC1 | 4.54E-05 | 0.004 | 540 | 0.283 |
| ENSP00000219919 | AQP9 | 5.44E-05 | 0.004 | 435 | 0.448 |
| ENSP00000306523 | INSM2 | 3.91E-05 | 0.004 | 430 | 0.147 |
| ENSP00000297157 | RP9 | 5.17E-05 | 0.004 | 427 | 0.342 |
| ENSP00000188790 | FAP | 4.35E-05 | 0.004 | 423 | 0.078 |
| ENSP00000293218 | UNK | 2.75E-05 | 0.004 | 335 | 0.139 |
| ENSP00000407942 | C4B | 3.99E-05 | 0.004 | 327 | 0.217 |
| ENSP00000293276 | CCL15 | 4.54E-05 | 0.004 | 307 | 0.306 |
| ENSP00000302150 | PRL | 1.35E-04 | 0.005 | 995 | 0.717 |
| ENSP00000302961 | HSPA4 | 1.51E-04 | 0.005 | 985 | 0.638 |
| ENSP00000356087 | IKBKE | 8.12E-05 | 0.005 | 985 | 0.576 |
| ENSP00000189444 | NFKB2 | 9.63E-05 | 0.005 | 979 | 0.769 |
| ENSP00000306512 | IL8 | 1.82E-04 | 0.005 | 978 | 0.834 |
| ENSP00000369554 | IFNA2 | 9.36E-05 | 0.005 | 972 | 0.713 |
| ENSP00000263464 | BIRC3 | 8.28E-05 | 0.005 | 945 | 0.657 |
| ENSP00000363965 | ALPL | 2.12E-04 | 0.005 | 942 | 0.730 |
| ENSP00000296754 | ERAP1 | 7.24E-05 | 0.005 | 929 | 0.225 |
| ENSP00000328968 | SCN5A | 6.21E-05 | 0.005 | 861 | 0.842 |
| ENSP00000356213 | VIP | 9.01E-05 | 0.005 | 859 | 0.558 |
| ENSP00000303208 | PCSK9 | 8.27E-05 | 0.005 | 815 | 0.385 |
| ENSP00000394842 | GCA | 6.32E-05 | 0.005 | 794 | 0.150 |
| ENSP00000373091 | HLA-E | 7.54E-05 | 0.005 | 784 | 0.707 |
| ENSP00000263867 | CAPG | 4.33E-05 | 0.005 | 725 | 0.133 |
| ENSP00000389288 | HLA-DPB1 | 1.19E-04 | 0.005 | 669 | 0.696 |
| ENSP00000364784 | C6orf48 | 1.31E-05 | 0.005 | 553 | 0.145 |
| ENSP00000160262 | ICAM3 | 7.89E-05 | 0.005 | 544 | 0.663 |
| ENSP00000200652 | SLC22A4 | 7.70E-05 | 0.005 | 532 | 0.368 |
| ENSP00000390785 | MOG | 5.85E-05 | 0.005 | 480 | 0.615 |
| ENSP00000366095 | MOG | 5.85E-05 | 0.005 | 480 | 0.615 |
| ENSP00000343505 | TNFSF13 | 4.97E-05 | 0.005 | 401 | 0.597 |
| ENSP00000318355 | AQP10 | 5.54E-05 | 0.005 | 346 | 0.777 |
| ENSP00000257979 | MIP | 6.84E-05 | 0.005 | 335 | 0.606 |
| ENSP00000408697 | NUGGC | 1.25E-05 | 0.005 | 204 | 0.119 |
| ENSP00000248244 | TICAM1 | 4.45E-05 | 0.006 | 999 | 0.668 |
| ENSP00000311032 | CASP3 | 1.37E-04 | 0.006 | 997 | 0.802 |
| ENSP00000245414 | IRF1 | 1.06E-04 | 0.006 | 992 | 0.719 |
| ENSP00000382034 | HLA-DQB1 | 1.48E-04 | 0.006 | 985 | 0.770 |
| ENSP00000225831 | CCL2 | 1.46E-04 | 0.006 | 968 | 0.841 |
| ENSP00000291295 | CALM3 | 2.80E-04 | 0.006 | 967 | 0.719 |
| ENSP00000273430 | AGTR1 | 6.47E-05 | 0.006 | 960 | 0.648 |
| ENSP00000365651 | NPPB | 8.61E-05 | 0.006 | 959 | 0.406 |
| ENSP00000320084 | CD276 | 1.21E-04 | 0.006 | 955 | 0.823 |
| ENSP00000249075 | LIF | 1.14E-04 | 0.006 | 934 | 0.687 |
| ENSP00000306565 | ISG20 | 5.06E-05 | 0.006 | 914 | 0.492 |
| ENSP00000254322 | DNAJB1 | 6.66E-05 | 0.006 | 911 | 0.507 |
| ENSP00000255409 | CHI3L1 | 6.86E-05 | 0.006 | 893 | 0.383 |
| ENSP00000371327 | KLRC2 | 6.26E-05 | 0.006 | 851 | 0.349 |
| ENSP00000225275 | MPO | 1.03E-04 | 0.006 | 810 | 0.725 |
| ENSP00000263851 | IL7 | 9.14E-05 | 0.006 | 665 | 0.774 |
| ENSP00000316136 | KCNJ1 | 7.77E-05 | 0.006 | 659 | 0.693 |
| ENSP00000355330 | TGM2 | 7.65E-05 | 0.006 | 621 | 0.496 |
| ENSP00000396688 | C4A | 1.21E-04 | 0.006 | 609 | 0.420 |
| ENSP00000372815 | C4A | 1.20E-04 | 0.006 | 565 | 0.420 |
| ENSP00000390707 | HLA-E | 9.29E-05 | 0.006 | 556 | 0.707 |
| ENSP00000281834 | TNFSF4 | 4.07E-05 | 0.006 | 540 | 0.653 |
| ENSP00000364217 | AGER | 4.96E-05 | 0.006 | 534 | 0.620 |
| ENSP00000391898 | MOG | 5.85E-05 | 0.006 | 480 | 0.615 |
| ENSP00000220616 | TG | 6.00E-05 | 0.006 | 424 | 0.444 |
| ENSP00000280614 | CCRN4L | 3.56E-05 | 0.006 | 384 | 0.441 |
| ENSP00000274364 | IQGAP2 | 3.39E-05 | 0.006 | 375 | 0.255 |
| ENSP00000267079 | MAP3K12 | 3.09E-05 | 0.006 | 353 | 0.195 |
| ENSP00000284245 | C16orf74 | 1.31E-05 | 0.006 | 345 | 0.125 |
| ENSP00000275015 | NFKBIE | 5.26E-05 | 0.007 | 977 | 0.501 |
| ENSP00000356438 | PTGS2 | 1.94E-04 | 0.007 | 976 | 0.847 |
| ENSP00000373648 | KCNQ3 | 9.31E-05 | 0.007 | 962 | 0.773 |
| ENSP00000370381 | SLC12A1 | 9.24E-05 | 0.007 | 953 | 0.659 |
| ENSP00000217381 | SNTA1 | 5.33E-05 | 0.007 | 938 | 0.492 |
| ENSP00000295400 | TGFA | 6.37E-05 | 0.007 | 926 | 0.658 |
| ENSP00000352121 | PIK3CG | 9.93E-05 | 0.007 | 916 | 0.744 |
| ENSP00000370343 | IRF4 | 6.97E-05 | 0.007 | 905 | 0.681 |
| ENSP00000416599 | ZNRD1 | 7.26E-05 | 0.007 | 899 | 0.208 |
| ENSP00000354932 | TLR1 | 7.71E-05 | 0.007 | 804 | 0.695 |
| ENSP00000378723 | HLA-DMB | 4.85E-05 | 0.007 | 613 | 0.410 |
| ENSP00000398890 | HLA-DMB | 4.83E-05 | 0.007 | 613 | 0.410 |
| ENSP00000357624 | MARCKS | 5.26E-05 | 0.007 | 576 | 0.434 |
| ENSP00000356056 | DYNLT1 | 7.40E-05 | 0.007 | 565 | 0.145 |
| ENSP00000356320 | ULBP2 | 5.86E-05 | 0.007 | 468 | 0.524 |
| ENSP00000256649 | TRIM45 | 5.13E-05 | 0.007 | 461 | 0.264 |
| ENSP00000415941 | C4B | 4.74E-05 | 0.007 | 407 | 0.217 |
| ENSP00000409151 | HSPA1L | 8.73E-05 | 0.007 | 396 | 0.411 |
| ENSP00000374791 | IGKV6-21 | 2.72E-05 | 0.007 | 204 | 0.137 |
| ENSP00000155840 | KCNQ1 | 1.30E-04 | 0.008 | 999 | 0.825 |
| ENSP00000338018 | HIF1A | 1.01E-04 | 0.008 | 999 | 0.733 |
| ENSP00000376445 | TIRAP | 5.68E-05 | 0.008 | 999 | 0.761 |
| ENSP00000349467 | CALM1 | 3.28E-04 | 0.008 | 998 | 0.719 |
| ENSP00000365380 | FOXP3 | 1.71E-04 | 0.008 | 996 | 0.804 |
| ENSP00000329967 | TBK1 | 1.24E-04 | 0.008 | 994 | 0.644 |
| ENSP00000225245 | CCL3 | 5.30E-05 | 0.008 | 983 | 0.757 |
| ENSP00000361405 | MMP9 | 1.27E-04 | 0.008 | 958 | 0.801 |
| ENSP00000221515 | RETN | 5.25E-05 | 0.008 | 942 | 0.552 |
| ENSP00000300589 | NOD2 | 1.53E-04 | 0.008 | 929 | 0.773 |
| ENSP00000310036 | CD34 | 1.24E-04 | 0.008 | 924 | 0.728 |
| ENSP00000223190 | NRF1 | 6.41E-05 | 0.008 | 923 | 0.279 |
| ENSP00000353224 | TFRC | 1.14E-04 | 0.008 | 906 | 0.519 |
| ENSP00000013034 | NME1 | 7.58E-05 | 0.008 | 899 | 0.389 |
| ENSP00000402694 | HLA-E | 8.21E-05 | 0.008 | 896 | 0.707 |
| ENSP00000278379 | SLC1A2 | 5.41E-05 | 0.008 | 877 | 0.312 |
| ENSP00000252444 | LDLR | 1.14E-04 | 0.008 | 866 | 0.636 |
| ENSP00000291860 | KIR3DL3 | 4.63E-05 | 0.008 | 859 | 0.143 |
| ENSP00000216802 | PSME2 | 3.49E-05 | 0.008 | 829 | 0.174 |
| ENSP00000284240 | THY1 | 6.45E-05 | 0.008 | 820 | 0.479 |
| ENSP00000216254 | ACO2 | 6.19E-05 | 0.008 | 807 | 0.473 |
| ENSP00000290310 | KCNE2 | 5.70E-05 | 0.008 | 788 | 0.893 |
| ENSP00000364597 | PADI4 | 5.69E-05 | 0.008 | 752 | 0.387 |
| ENSP00000244043 | PTGIS | 3.85E-05 | 0.008 | 659 | 0.396 |
| ENSP00000265431 | CALB1 | 6.75E-05 | 0.008 | 657 | 0.344 |
| ENSP00000372793 | LTA | 8.33E-05 | 0.008 | 647 | 0.674 |
| ENSP00000395005 | MOG | 5.87E-05 | 0.008 | 480 | 0.615 |
| ENSP00000355136 | SORBS1 | 5.13E-05 | 0.008 | 424 | 0.211 |
| ENSP00000403495 | LTA | 6.13E-05 | 0.008 | 422 | 0.674 |
| ENSP00000296503 | HMGB2 | 5.15E-05 | 0.008 | 401 | 0.326 |
| ENSP00000407291 | RNF39 | 4.46E-05 | 0.008 | 401 | 0.107 |
| ENSP00000408539 | RNF39 | 4.46E-05 | 0.008 | 401 | 0.107 |
| ENSP00000244360 | RNF39 | 4.30E-05 | 0.008 | 385 | 0.107 |
| ENSP00000399298 | HLA-DPB1 | 6.19E-05 | 0.008 | 273 | 0.696 |
| ENSP00000387930 | RNF39 | 3.52E-05 | 0.008 | 273 | 0.107 |
| ENSP00000405838 | HLA-DPA1 | 1.88E-05 | 0.008 | 154 | 0.363 |
| ENSP00000386341 | TICAM2 | 3.85E-05 | 0.009 | 999 | 0.666 |
| ENSP00000241052 | CAT | 1.05E-04 | 0.009 | 992 | 0.753 |
| ENSP00000368349 | POLA1 | 1.84E-04 | 0.009 | 985 | 0.708 |
| ENSP00000221930 | TGFB1 | 1.88E-04 | 0.009 | 984 | 0.798 |
| ENSP00000231449 | IL4 | 1.12E-04 | 0.009 | 970 | 0.792 |
| ENSP00000272298 | CALM2 | 2.84E-04 | 0.009 | 967 | 0.716 |
| ENSP00000301408 | CGB5 | 3.18E-05 | 0.009 | 956 | 0.285 |
| ENSP00000358802 | KCNC4 | 6.37E-05 | 0.009 | 931 | 0.743 |
| ENSP00000320758 | NOS1 | 9.35E-05 | 0.009 | 906 | 0.672 |
| ENSP00000352035 | KCNQ2 | 8.17E-05 | 0.009 | 905 | 0.721 |
| ENSP00000357625 | BNIP3 | 5.80E-05 | 0.009 | 890 | 0.398 |
| ENSP00000313644 | MAP4K4 | 5.11E-05 | 0.009 | 884 | 0.316 |
| ENSP00000395701 | TAPBP | 7.75E-05 | 0.009 | 882 | 0.273 |
| ENSP00000259206 | IL1RN | 9.78E-05 | 0.009 | 808 | 0.817 |
| ENSP00000264867 | PPARGC1A | 7.17E-05 | 0.009 | 694 | 0.505 |
| ENSP00000407674 | HLA-DPB1 | 9.34E-05 | 0.009 | 629 | 0.696 |
| ENSP00000348918 | SAA1 | 4.76E-05 | 0.009 | 621 | 0.627 |
| ENSP00000262065 | MMD | 5.59E-05 | 0.009 | 460 | 0.329 |
| ENSP00000329869 | TPO | 4.86E-05 | 0.009 | 424 | 0.351 |
| ENSP00000308610 | GPD2 | 7.31E-05 | 0.009 | 404 | 0.329 |
| ENSP00000354581 | GPX6 | 3.72E-05 | 0.009 | 379 | 0.303 |
| ENSP00000218721 | MLNR | 4.25E-05 | 0.009 | 305 | 0.257 |
| ENSP00000339151 | IKBKB | 8.21E-05 | 0.01 | 997 | 0.716 |
| ENSP00000219070 | MMP2 | 8.55E-05 | 0.01 | 962 | 0.706 |
| ENSP00000265023 | KNG1 | 1.04E-04 | 0.01 | 954 | 0.684 |
| ENSP00000247461 | CANX | 1.34E-04 | 0.01 | 939 | 0.353 |
| ENSP00000386884 | CXCR4 | 1.47E-04 | 0.01 | 934 | 0.749 |
| ENSP00000365048 | TNFSF13B | 8.76E-05 | 0.01 | 931 | 0.724 |
| ENSP00000342952 | ADCY2 | 1.17E-04 | 0.01 | 925 | 0.610 |
| ENSP00000369581 | IFNB1 | 8.41E-05 | 0.01 | 901 | 0.702 |
| ENSP00000268638 | IRF8 | 6.28E-05 | 0.01 | 900 | 0.666 |
| ENSP00000003084 | CFTR | 1.80E-04 | 0.01 | 897 | 0.535 |
| ENSP00000378546 | TAPBP | 7.33E-05 | 0.01 | 886 | 0.273 |
| ENSP00000222823 | NOD1 | 7.14E-05 | 0.01 | 877 | 0.722 |
| ENSP00000364076 | HLA-DQA2 | 6.53E-05 | 0.01 | 659 | 0.726 |
| ENSP00000347427 | MINK1 | 5.51E-05 | 0.01 | 651 | 0.429 |
| ENSP00000349252 | ITGAL | 7.47E-05 | 0.01 | 633 | 0.709 |
| ENSP00000254325 | RFX1 | 6.79E-05 | 0.01 | 627 | 0.184 |
| ENSP00000361467 | DLG5 | 5.60E-05 | 0.01 | 578 | 0.322 |
| ENSP00000247829 | TSPAN8 | 5.68E-05 | 0.01 | 542 | 0.293 |
| ENSP00000261745 | NAA25 | 4.75E-05 | 0.01 | 373 | 0.191 |
| ENSP00000220166 | CTSH | 5.28E-05 | 0.01 | 266 | 0.296 |
| ENSP00000264246 | CD80 | 1.49E-04 | 0.011 | 999 | 0.813 |
| ENSP00000162749 | TNFRSF1A | 1.11E-04 | 0.011 | 999 | 0.825 |
| ENSP00000302665 | IGF1 | 1.22E-04 | 0.011 | 994 | 0.787 |
| ENSP00000362994 | TRAF1 | 7.70E-05 | 0.011 | 947 | 0.727 |
| ENSP00000236850 | APOA1 | 1.16E-04 | 0.011 | 942 | 0.562 |
| ENSP00000263923 | KDR | 7.30E-05 | 0.011 | 919 | 0.636 |
| ENSP00000335062 | PDCD1 | 5.63E-05 | 0.011 | 914 | 0.610 |
| ENSP00000363804 | KLF4 | 6.73E-05 | 0.011 | 902 | 0.628 |
| ENSP00000325663 | NFKBIZ | 4.62E-05 | 0.011 | 899 | 0.498 |
| ENSP00000366525 | FTL | 7.56E-05 | 0.011 | 859 | 0.615 |
| ENSP00000362057 | NOX1 | 6.36E-05 | 0.011 | 851 | 0.489 |
| ENSP00000372295 | GJB2 | 8.32E-05 | 0.011 | 804 | 0.506 |
| ENSP00000265800 | EPB49 | 5.87E-05 | 0.011 | 788 | 0.249 |
| ENSP00000233242 | APOB | 9.31E-05 | 0.011 | 770 | 0.545 |
| ENSP00000355627 | AGT | 1.48E-04 | 0.011 | 680 | 0.695 |
| ENSP00000266086 | SLC5A4 | 4.22E-05 | 0.011 | 645 | 0.463 |
| ENSP00000357651 | TUBE1 | 3.17E-05 | 0.011 | 569 | 0.257 |
| ENSP00000399309 | NEU1 | 9.59E-05 | 0.011 | 502 | 0.455 |
| ENSP00000364782 | NEU1 | 9.20E-05 | 0.011 | 500 | 0.455 |
| ENSP00000283977 | PGM3 | 9.13E-05 | 0.011 | 462 | 0.121 |
| ENSP00000360312 | BSND | 5.61E-05 | 0.011 | 451 | 0.521 |
| ENSP00000347314 | SPG20 | 4.83E-05 | 0.011 | 430 | 0.186 |
| ENSP00000363763 | EPHB2 | 6.63E-05 | 0.011 | 422 | 0.469 |
| ENSP00000348019 | SLC17A5 | 5.20E-05 | 0.011 | 343 | 0.391 |
| ENSP00000331827 | TNFAIP8L1 | 4.16E-05 | 0.011 | 336 | 0.101 |
| ENSP00000372718 | HLA-DMB | 1.99E-05 | 0.011 | 160 | 0.410 |
| ENSP00000413471 | HLA-DMB | 1.97E-05 | 0.011 | 160 | 0.410 |
| ENSP00000227758 | BIRC2 | 7.13E-05 | 0.012 | 993 | 0.598 |
| ENSP00000226730 | IL2 | 2.08E-04 | 0.012 | 989 | 0.809 |
| ENSP00000286758 | CXCL13 | 7.27E-05 | 0.012 | 956 | 0.752 |
| ENSP00000303231 | IL12A | 3.54E-05 | 0.012 | 950 | 0.727 |
| ENSP00000223095 | SERPINE1 | 8.49E-05 | 0.012 | 942 | 0.704 |
| ENSP00000377218 | IRF2 | 7.18E-05 | 0.012 | 916 | 0.588 |
| ENSP00000370473 | IGFBP3 | 6.68E-05 | 0.012 | 910 | 0.685 |
| ENSP00000358786 | KCNA10 | 5.14E-05 | 0.012 | 910 | 0.781 |
| ENSP00000262999 | UCP1 | 5.10E-05 | 0.012 | 894 | 0.471 |
| ENSP00000248071 | KLF2 | 5.69E-05 | 0.012 | 884 | 0.521 |
| ENSP00000382018 | HLA-DQB1 | 1.16E-04 | 0.012 | 658 | 0.770 |
| ENSP00000406872 | HLA-DQB2 | 4.37E-05 | 0.012 | 604 | 0.294 |
| ENSP00000353500 | CLEC4C | 2.84E-05 | 0.012 | 563 | 0.587 |
| ENSP00000412309 | SLC14A1 | 2.39E-05 | 0.012 | 551 | 0.420 |
| ENSP00000357152 | CD1C | 3.29E-05 | 0.012 | 468 | 0.637 |
| ENSP00000295463 | ALPI | 4.57E-05 | 0.012 | 434 | 0.204 |
| ENSP00000219660 | AQP8 | 5.05E-05 | 0.012 | 312 | 0.776 |
| ENSP00000397073 | TRIM10 | 3.07E-05 | 0.012 | 296 | 0.359 |
| ENSP00000362613 | CDX4 | 5.47E-05 | 0.012 | 268 | 0.374 |
| ENSP00000363941 | HLA-DPA1 | 1.92E-05 | 0.012 | 154 | 0.363 |
| ENSP00000283635 | CD8A | 1.06E-04 | 0.013 | 990 | 0.748 |
| ENSP00000347988 | NDUFA5 | 9.67E-05 | 0.013 | 968 | 0.448 |
| ENSP00000339933 | PKLR | 7.79E-05 | 0.013 | 909 | 0.433 |
| ENSP00000301974 | PTAFR | 6.20E-05 | 0.013 | 904 | 0.597 |
| ENSP00000006053 | CX3CL1 | 6.43E-05 | 0.013 | 903 | 0.494 |
| ENSP00000333656 | SIGIRR | 2.83E-05 | 0.013 | 709 | 0.726 |
| ENSP00000393646 | HLA-DMB | 5.93E-05 | 0.013 | 612 | 0.410 |
| ENSP00000170630 | IL4R | 4.69E-05 | 0.013 | 514 | 0.752 |
| ENSP00000226359 | AFP | 1.19E-04 | 0.013 | 493 | 0.393 |
| ENSP00000234590 | ENO1 | 7.91E-05 | 0.013 | 480 | 0.429 |
| ENSP00000401149 | TAP1 | 4.28E-05 | 0.013 | 283 | 0.371 |
| ENSP00000324827 | AKAP17A | 3.85E-05 | 0.013 | 249 | 0.318 |
| ENSP00000246081 | OTOR | 1.16E-05 | 0.013 | 227 | 0.343 |
| ENSP00000231228 | IL12B | 5.94E-05 | 0.014 | 981 | 0.763 |
| ENSP00000262186 | KCNH2 | 1.10E-04 | 0.014 | 959 | 0.781 |
| ENSP00000345809 | ZNF197 | 1.32E-05 | 0.014 | 927 | 0.131 |
| ENSP00000263125 | PRKCQ | 1.03E-04 | 0.014 | 919 | 0.416 |
| ENSP00000371729 | SACS | 5.14E-05 | 0.014 | 918 | 0.273 |
| ENSP00000402951 | HLA-DRA | 4.78E-05 | 0.014 | 917 | 0.420 |
| ENSP00000320709 | ADIPOQ | 4.39E-05 | 0.014 | 909 | 0.432 |
| ENSP00000414196 | HLA-DPB1 | 1.06E-04 | 0.014 | 843 | 0.696 |
| ENSP00000206544 | SLC22A17 | 2.96E-05 | 0.014 | 768 | 0.121 |
| ENSP00000307875 | B3GAT1 | 6.65E-05 | 0.014 | 565 | 0.489 |
| ENSP00000359799 | DNAJB4 | 3.93E-05 | 0.014 | 556 | 0.107 |
| ENSP00000229402 | KLRB1 | 6.27E-05 | 0.014 | 545 | 0.660 |
| ENSP00000307235 | EIF2AK3 | 6.14E-05 | 0.014 | 544 | 0.645 |
| ENSP00000371234 | CYS1 | 2.69E-05 | 0.014 | 435 | 0.309 |
| ENSP00000348831 | NUDT10 | 6.39E-05 | 0.014 | 429 | 0.069 |
| ENSP00000293826 | TNFSF12-TNFSF13 | 4.37E-05 | 0.014 | 427 | 0.503 |
| ENSP00000268296 | ITGAX | 7.01E-05 | 0.014 | 385 | 0.651 |
| ENSP00000261652 | TNFRSF13B | 4.62E-05 | 0.014 | 374 | 0.585 |
| ENSP00000362608 | PIM1 | 3.12E-05 | 0.014 | 340 | 0.156 |
| ENSP00000357013 | CD244 | 5.51E-05 | 0.014 | 281 | 0.424 |
| ENSP00000261514 | CLCN3 | 4.60E-05 | 0.014 | 270 | 0.433 |
| ENSP00000233946 | IL1R1 | 8.11E-05 | 0.015 | 999 | 0.858 |
| ENSP00000284818 | LY96 | 5.74E-05 | 0.015 | 999 | 0.754 |
| ENSP00000359424 | CHUK | 8.12E-05 | 0.015 | 992 | 0.498 |
| ENSP00000353940 | PMEL | 5.57E-05 | 0.015 | 953 | 0.381 |
| ENSP00000268182 | IQGAP1 | 5.19E-05 | 0.015 | 936 | 0.380 |
| ENSP00000215637 | MADCAM1 | 5.07E-05 | 0.015 | 909 | 0.636 |
| ENSP00000295598 | ATP1A1 | 5.75E-05 | 0.015 | 655 | 0.313 |
| ENSP00000285379 | CA2 | 9.84E-05 | 0.015 | 609 | 0.579 |
| ENSP00000266557 | CD27 | 7.29E-05 | 0.015 | 609 | 0.736 |
| ENSP00000409159 | HLA-DQB2 | 4.35E-05 | 0.015 | 604 | 0.294 |
| ENSP00000392762 | DCT | 5.78E-05 | 0.015 | 425 | 0.609 |
| ENSP00000417970 | FAM120B | 1.88E-05 | 0.015 | 340 | 0.375 |
| ENSP00000358815 | NEURL1B | 3.09E-05 | 0.015 | 291 | 0.297 |
| ENSP00000265026 | MAP3K13 | 2.63E-05 | 0.015 | 285 | 0.212 |
| ENSP00000349954 | CGB | 3.22E-05 | 0.016 | 972 | 0.315 |
| ENSP00000250151 | CCL4 | 6.49E-05 | 0.016 | 947 | 0.820 |
| ENSP00000334145 | F3 | 7.61E-05 | 0.016 | 930 | 0.660 |
| ENSP00000252321 | KCNA5 | 7.39E-05 | 0.016 | 927 | 0.831 |
| ENSP00000331602 | PRKCD | 9.11E-05 | 0.016 | 926 | 0.426 |
| ENSP00000271651 | CTSK | 7.92E-05 | 0.016 | 926 | 0.522 |
| ENSP00000363548 | CXCL12 | 1.02E-04 | 0.016 | 922 | 0.716 |
| ENSP00000384886 | IFI30 | 5.96E-05 | 0.016 | 922 | 0.456 |
| ENSP00000308165 | CD36 | 8.32E-05 | 0.016 | 914 | 0.672 |
| ENSP00000369889 | COL2A1 | 1.01E-04 | 0.016 | 845 | 0.468 |
| ENSP00000324648 | CYP2B6 | 9.00E-05 | 0.016 | 659 | 0.637 |
| ENSP00000327545 | NPTXR | 6.06E-05 | 0.016 | 615 | 0.449 |
| ENSP00000356489 | EPM2A | 3.81E-05 | 0.016 | 602 | 0.166 |
| ENSP00000345464 | NHLRC1 | 4.33E-05 | 0.016 | 595 | 0.128 |
| ENSP00000357025 | CD48 | 5.65E-05 | 0.016 | 461 | 0.619 |
| ENSP00000228916 | SCNN1A | 5.10E-05 | 0.016 | 461 | 0.512 |
| ENSP00000255427 | CHIT1 | 6.01E-05 | 0.016 | 432 | 0.253 |
| ENSP00000410815 | CFB | 2.28E-05 | 0.016 | 404 | 0.000 |
| ENSP00000292199 | NLRX1 | 6.09E-05 | 0.016 | 368 | 0.328 |
| ENSP00000364801 | HSPA1B | 5.68E-05 | 0.016 | 340 | 0.775 |
| ENSP00000375391 | HSPA1B | 5.67E-05 | 0.016 | 340 | 0.775 |
| ENSP00000375399 | HSPA1B | 5.67E-05 | 0.016 | 340 | 0.775 |
| ENSP00000403530 | HSPA1B | 5.67E-05 | 0.016 | 340 | 0.775 |
| ENSP00000372881 | HSPA1L | 5.22E-05 | 0.016 | 337 | 0.411 |
| ENSP00000408347 | HSPA1L | 5.22E-05 | 0.016 | 337 | 0.411 |
| ENSP00000297988 | AQP7 | 5.72E-05 | 0.016 | 332 | 0.676 |
| ENSP00000370003 | BNIP3L | 3.62E-05 | 0.016 | 313 | 0.236 |
| ENSP00000354632 | MT-ATP6 | 3.59E-05 | 0.016 | 305 | 0.299 |
| ENSP00000231656 | CDX1 | 5.28E-05 | 0.016 | 303 | 0.396 |
| ENSP00000418001 | GK5 | 4.55E-05 | 0.016 | 247 | 0.529 |
| ENSP00000216605 | MTHFD1 | 4.92E-05 | 0.017 | 981 | 0.442 |
| ENSP00000264657 | STAT3 | 1.26E-04 | 0.017 | 953 | 0.802 |
| ENSP00000276689 | NDUFB9 | 6.49E-05 | 0.017 | 937 | 0.514 |
| ENSP00000354782 | CD247 | 5.80E-05 | 0.017 | 932 | 0.566 |
| ENSP00000280193 | VEGFC | 5.64E-05 | 0.017 | 895 | 0.539 |
| ENSP00000263826 | AKT3 | 8.62E-05 | 0.017 | 874 | 0.406 |
| ENSP00000224237 | VIM | 1.18E-04 | 0.017 | 864 | 0.687 |
| ENSP00000235933 | CD160 | 3.97E-05 | 0.017 | 857 | 0.473 |
| ENSP00000374467 | ABCC8 | 5.76E-05 | 0.017 | 815 | 0.409 |
| ENSP00000348545 | CGB7 | 2.48E-05 | 0.017 | 752 | 0.298 |
| ENSP00000221972 | CD79A | 1.74E-04 | 0.017 | 750 | 0.733 |
| ENSP00000345492 | SH2B3 | 5.15E-05 | 0.017 | 627 | 0.288 |
| ENSP00000353679 | MME | 5.65E-05 | 0.017 | 563 | 0.522 |
| ENSP00000221466 | FCGRT | 6.59E-05 | 0.017 | 441 | 0.418 |
| ENSP00000261937 | FLT4 | 5.41E-05 | 0.017 | 433 | 0.549 |
| ENSP00000379500 | UEVLD | 4.54E-05 | 0.017 | 429 | 0.179 |
| ENSP00000383503 | ZNRD1 | 5.01E-05 | 0.017 | 388 | 0.208 |
| ENSP00000372726 | TAP2 | 3.99E-05 | 0.017 | 293 | 0.167 |
| ENSP00000259808 | RIPK1 | 6.16E-05 | 0.018 | 998 | 0.652 |
| ENSP00000360266 | JUN | 1.64E-04 | 0.018 | 994 | 0.787 |
| ENSP00000337825 | LCK | 1.28E-04 | 0.018 | 994 | 0.661 |
| ENSP00000211122 | GSTA3 | 6.52E-05 | 0.018 | 984 | 0.684 |
| ENSP00000362795 | CXCR3 | 6.24E-05 | 0.018 | 984 | 0.789 |
| ENSP00000379110 | CXCL1 | 7.15E-05 | 0.018 | 973 | 0.807 |
| ENSP00000358081 | BAG3 | 4.40E-05 | 0.018 | 968 | 0.261 |
| ENSP00000310127 | IRF3 | 7.93E-05 | 0.018 | 964 | 0.753 |
| ENSP00000319591 | KCND3 | 5.40E-05 | 0.018 | 915 | 0.715 |
| ENSP00000395337 | LDHA | 7.17E-05 | 0.018 | 884 | 0.428 |
| ENSP00000281537 | TJP1 | 9.75E-05 | 0.018 | 882 | 0.556 |
| ENSP00000370408 | CDX2 | 6.47E-05 | 0.018 | 858 | 0.570 |
| ENSP00000318340 | AC026703.1 | 4.68E-05 | 0.018 | 835 | 0.000 |
| ENSP00000331514 | ACTG1 | 1.23E-04 | 0.018 | 814 | 0.678 |
| ENSP00000357068 | KCNJ10 | 8.78E-05 | 0.018 | 792 | 0.583 |
| ENSP00000243457 | KCNJ2 | 5.17E-05 | 0.018 | 752 | 0.754 |
| ENSP00000266839 | MMAB | 4.37E-05 | 0.018 | 733 | 0.182 |
| ENSP00000342300 | NELFCD | 4.41E-05 | 0.018 | 675 | 0.681 |
| ENSP00000217407 | LBP | 3.88E-05 | 0.018 | 673 | 0.644 |
| ENSP00000386331 | MYO7A | 7.53E-05 | 0.018 | 542 | 0.339 |
| ENSP00000200676 | CETP | 3.58E-05 | 0.018 | 534 | 0.603 |
| ENSP00000365431 | TCF19 | 4.37E-05 | 0.018 | 523 | 0.257 |
| ENSP00000364028 | ECE1 | 2.14E-05 | 0.018 | 462 | 0.419 |
| ENSP00000356016 | CR1 | 5.69E-05 | 0.018 | 460 | 0.564 |
| ENSP00000229319 | LDHB | 5.92E-05 | 0.018 | 391 | 0.306 |
| ENSP00000387438 | TNXB | 4.02E-05 | 0.018 | 315 | 0.283 |
| ENSP00000389677 | TNXB | 4.02E-05 | 0.018 | 315 | 0.283 |
| ENSP00000359504 | GBP1 | 4.79E-05 | 0.019 | 914 | 0.523 |
| ENSP00000255631 | HSPBP1 | 4.39E-05 | 0.019 | 905 | 0.112 |
| ENSP00000354111 | DNAJC5 | 5.54E-05 | 0.019 | 864 | 0.167 |
| ENSP00000276570 | DNAJC5B | 4.40E-05 | 0.019 | 864 | 0.069 |
| ENSP00000332124 | MRC1L1 | 4.37E-05 | 0.019 | 859 | 0.000 |
| ENSP00000302812 | SP7 | 5.42E-05 | 0.019 | 803 | 0.640 |
| ENSP00000268704 | SPG7 | 5.69E-05 | 0.019 | 800 | 0.740 |
| ENSP00000313419 | CD19 | 1.19E-04 | 0.019 | 655 | 0.784 |
| ENSP00000341828 | CHIA | 4.07E-05 | 0.019 | 432 | 0.307 |
| ENSP00000379651 | LILRA6 | 4.32E-05 | 0.019 | 427 | 0.547 |
| ENSP00000229134 | IL26 | 4.13E-05 | 0.019 | 340 | 0.505 |
| ENSP00000406495 | TNXB | 4.03E-05 | 0.019 | 315 | 0.283 |
| ENSP00000354451 | IQGAP3 | 3.11E-05 | 0.019 | 270 | 0.234 |
| ENSP00000266003 | MLN | 4.07E-05 | 0.019 | 259 | 0.265 |
| ENSP00000351706 | GK2 | 4.55E-05 | 0.019 | 247 | 0.561 |
| ENSP00000299694 | BEAN1 | 2.26E-05 | 0.019 | 200 | 0.306 |
| ENSP00000312299 | FRG2C | 1.26E-05 | 0.019 | 196 | 0.325 |
| ENSP00000384730 | FRG2C | 1.26E-05 | 0.019 | 196 | 0.000 |
| ENSP00000347582 | CGB8 | 3.34E-05 | 0.02 | 999 | 0.294 |
| ENSP00000335620 | GSTA1 | 6.55E-05 | 0.02 | 984 | 0.670 |
| ENSP00000289081 | FANCC | 4.94E-05 | 0.02 | 941 | 0.424 |
| ENSP00000382025 | HLA-DQB1 | 1.22E-04 | 0.02 | 923 | 0.770 |
| ENSP00000356346 | PTPRC | 1.07E-04 | 0.02 | 913 | 0.761 |
| ENSP00000359497 | GBP2 | 4.53E-05 | 0.02 | 909 | 0.496 |
| ENSP00000355470 | CNST | 4.40E-05 | 0.02 | 909 | 0.360 |
| ENSP00000354652 | NPY1R | 4.42E-05 | 0.02 | 901 | 0.649 |
| ENSP00000239938 | EGR1 | 7.87E-05 | 0.02 | 899 | 0.695 |
| ENSP00000274353 | BHMT | 3.52E-05 | 0.02 | 835 | 0.484 |
| ENSP00000340089 | TLR5 | 5.29E-05 | 0.02 | 733 | 0.833 |
| ENSP00000250092 | CD68 | 8.94E-05 | 0.02 | 649 | 0.734 |
| ENSP00000381486 | NOTO | 3.18E-05 | 0.02 | 623 | 0.447 |
| ENSP00000371376 | TLR6 | 2.95E-05 | 0.02 | 516 | 0.828 |
| ENSP00000264037 | TECTA | 6.19E-05 | 0.02 | 466 | 0.352 |
| ENSP00000395497 | GTF2H4 | 3.73E-05 | 0.02 | 408 | 0.196 |
| ENSP00000333666 | ADI1 | 3.40E-05 | 0.02 | 386 | 0.278 |
| ENSP00000280200 | CD226 | 4.84E-05 | 0.02 | 374 | 0.482 |
| ENSP00000357892 | FAM24B | 3.46E-05 | 0.02 | 340 | 0.156 |
| ENSP00000295989 | CAND2 | 3.37E-05 | 0.02 | 319 | 0.443 |
| ENSP00000372599 | TAP2 | 3.90E-05 | 0.02 | 293 | 0.167 |
| ENSP00000357158 | FCRL1 | 3.84E-05 | 0.02 | 273 | 0.173 |
| ENSP00000397139 | HLA-DPA1 | 1.90E-05 | 0.02 | 154 | 0.363 |
| ENSP00000263734 | EPAS1 | 4.80E-05 | 0.021 | 999 | 0.508 |
| ENSP00000306884 | CXCL11 | 4.21E-05 | 0.021 | 982 | 0.782 |
| ENSP00000370880 | MLANA | 4.97E-05 | 0.021 | 954 | 0.377 |
| ENSP00000363976 | HLA-DMA | 9.74E-05 | 0.021 | 939 | 0.445 |
| ENSP00000253408 | GFAP | 9.28E-05 | 0.021 | 936 | 0.640 |
| ENSP00000344352 | ATF3 | 6.36E-05 | 0.021 | 913 | 0.565 |
| ENSP00000302707 | FPR1 | 5.99E-05 | 0.021 | 910 | 0.598 |
| ENSP00000356530 | RNASEL | 5.03E-05 | 0.021 | 906 | 0.464 |
| ENSP00000262375 | DNAJA3 | 5.24E-05 | 0.021 | 833 | 0.278 |
| ENSP00000001008 | FKBP4 | 4.15E-05 | 0.021 | 729 | 0.226 |
| ENSP00000263621 | ELANE | 7.63E-05 | 0.021 | 679 | 0.645 |
| ENSP00000396320 | SCN4A | 4.14E-05 | 0.021 | 601 | 0.710 |
| ENSP00000308895 | SLC19A1 | 3.32E-05 | 0.021 | 567 | 0.506 |
| ENSP00000348273 | MBP | 8.61E-05 | 0.021 | 543 | 0.609 |
| ENSP00000403721 | CYP21A2 | 4.10E-05 | 0.021 | 409 | 0.337 |
| ENSP00000040663 | MRI1 | 3.72E-05 | 0.021 | 392 | 0.273 |
| ENSP00000228438 | CLEC2B | 4.50E-05 | 0.021 | 372 | 0.573 |
| ENSP00000311857 | PTPN2 | 4.64E-05 | 0.021 | 238 | 0.420 |
| ENSP00000207870 | XYLB | 2.98E-05 | 0.021 | 223 | 0.175 |
| ENSP00000330959 | IL1R2 | 3.18E-05 | 0.022 | 999 | 0.637 |
| ENSP00000420168 | GSTA2 | 6.49E-05 | 0.022 | 984 | 0.680 |
| ENSP00000329380 | GP1BA | 4.95E-05 | 0.022 | 963 | 0.221 |
| ENSP00000321345 | IL23R | 7.64E-05 | 0.022 | 936 | 0.750 |
| ENSP00000380150 | CARD11 | 4.03E-05 | 0.022 | 914 | 0.526 |
| ENSP00000365572 | NME2 | 5.40E-05 | 0.022 | 888 | 0.318 |
| ENSP00000373477 | GPX3 | 5.20E-05 | 0.022 | 882 | 0.526 |
| ENSP00000382697 | ROCK1 | 5.24E-05 | 0.022 | 837 | 0.438 |
| ENSP00000216341 | GZMB | 8.17E-05 | 0.022 | 803 | 0.756 |
| ENSP00000301838 | FADD | 6.12E-05 | 0.022 | 715 | 0.642 |
| ENSP00000405295 | HLA-DRA | 4.90E-05 | 0.022 | 622 | 0.420 |
| ENSP00000221403 | DHDH | 4.42E-05 | 0.022 | 562 | 0.180 |
| ENSP00000253699 | ZFYVE20 | 3.82E-05 | 0.022 | 528 | 0.108 |
| ENSP00000363435 | ITPR3 | 7.01E-05 | 0.022 | 506 | 0.512 |
| ENSP00000415786 | SERPINE2 | 4.82E-05 | 0.022 | 437 | 0.279 |
| ENSP00000307218 | NAT1 | 4.95E-05 | 0.022 | 429 | 0.402 |
| ENSP00000272371 | OTOF | 4.65E-05 | 0.022 | 380 | 0.282 |
| ENSP00000408860 | CYP21A2 | 3.81E-05 | 0.022 | 340 | 0.337 |
| ENSP00000394942 | CYP21A2 | 3.80E-05 | 0.022 | 340 | 0.337 |
| ENSP00000333802 | ZNF599 | 1.11E-05 | 0.022 | 330 | 0.199 |
| ENSP00000216797 | NFKBIA | 9.20E-05 | 0.023 | 999 | 0.825 |
| ENSP00000270142 | SOD1 | 9.76E-05 | 0.023 | 998 | 0.668 |
| ENSP00000321203 | TNIP2 | 2.74E-05 | 0.023 | 992 | 0.495 |
| ENSP00000216274 | RIPK3 | 3.07E-05 | 0.023 | 954 | 0.492 |
| ENSP00000357981 | CTSS | 1.15E-04 | 0.023 | 931 | 0.720 |
| ENSP00000416330 | TGFBI | 1.14E-04 | 0.023 | 923 | 0.760 |
| ENSP00000358031 | C1orf138 | 2.02E-05 | 0.023 | 899 | 0.164 |
| ENSP00000353731 | DPP4 | 6.62E-05 | 0.023 | 896 | 0.367 |
| ENSP00000337088 | MEN1 | 5.32E-05 | 0.023 | 873 | 0.366 |
| ENSP00000356771 | F5 | 5.94E-05 | 0.023 | 867 | 0.539 |
| ENSP00000414817 | HLA-DMB | 4.11E-05 | 0.023 | 816 | 0.410 |
| ENSP00000364805 | HSPA1L | 6.56E-05 | 0.023 | 657 | 0.411 |
| ENSP00000257248 | GIF | 3.59E-05 | 0.023 | 467 | 0.454 |
| ENSP00000379566 | CCHCR1 | 4.39E-05 | 0.023 | 462 | 0.254 |
| ENSP00000393087 | HSPA1B | 7.87E-05 | 0.023 | 445 | 0.775 |
| ENSP00000222248 | SLC5A5 | 6.38E-05 | 0.023 | 429 | 0.468 |
| ENSP00000362463 | GLO1 | 6.63E-05 | 0.023 | 420 | 0.275 |
| ENSP00000320493 | TRIP10 | 4.53E-05 | 0.023 | 417 | 0.297 |
| ENSP00000345868 | GJB4 | 3.52E-05 | 0.023 | 393 | 0.682 |
| ENSP00000405636 | ZNRD1 | 4.74E-05 | 0.023 | 388 | 0.208 |
| ENSP00000240662 | KCNJ8 | 2.78E-05 | 0.023 | 374 | 0.735 |
| ENSP00000404619 | PRRC2A | 6.72E-05 | 0.023 | 362 | 0.125 |
| ENSP00000265598 | LAMP3 | 1.82E-05 | 0.023 | 319 | 0.606 |
| ENSP00000335392 | SPATA12 | 1.21E-05 | 0.023 | 319 | 0.109 |
| ENSP00000267436 | L2HGDH | 4.18E-05 | 0.023 | 223 | 0.449 |
| ENSP00000303532 | DEFB4A | 7.30E-05 | 0.024 | 973 | 0.556 |
| ENSP00000360247 | CYP2J2 | 6.70E-05 | 0.024 | 953 | 0.632 |
| ENSP00000009530 | CD74 | 1.29E-04 | 0.024 | 943 | 0.537 |
| ENSP00000264896 | SCARB2 | 7.76E-05 | 0.024 | 906 | 0.686 |
| ENSP00000261693 | SCARB1 | 7.68E-05 | 0.024 | 906 | 0.653 |
| ENSP00000409378 | CHRM4 | 5.03E-05 | 0.024 | 899 | 0.663 |
| ENSP00000397705 | HLA-F | 7.09E-05 | 0.024 | 845 | 0.389 |
| ENSP00000261187 | SLC16A7 | 3.58E-05 | 0.024 | 841 | 0.104 |
| ENSP00000310216 | KLRC4 | 3.05E-05 | 0.024 | 812 | 0.349 |
| ENSP00000263863 | GNLY | 6.08E-05 | 0.024 | 658 | 0.466 |
| ENSP00000404533 | HLA-DRA | 4.98E-05 | 0.024 | 619 | 0.420 |
| ENSP00000414905 | HLA-F | 6.51E-05 | 0.024 | 551 | 0.389 |
| ENSP00000391181 | CCHCR1 | 4.17E-05 | 0.024 | 462 | 0.254 |
| ENSP00000397598 | KY | 3.80E-05 | 0.024 | 423 | 0.342 |
| ENSP00000350005 | GIP | 3.84E-05 | 0.024 | 374 | 0.344 |
| ENSP00000304783 | SLC26A5 | 3.81E-05 | 0.024 | 364 | 0.261 |
| ENSP00000350415 | GJA9 | 4.18E-05 | 0.024 | 363 | 0.653 |
| ENSP00000264716 | FOSL2 | 6.05E-05 | 0.024 | 305 | 0.410 |
| ENSP00000353824 | PIM3 | 2.46E-05 | 0.024 | 287 | 0.073 |
| ENSP00000361592 | ERMAP | 2.20E-05 | 0.024 | 272 | 0.347 |
| ENSP00000372608 | HLA-DRA | 5.28E-05 | 0.025 | 982 | 0.420 |
| ENSP00000309757 | LPL | 9.39E-05 | 0.025 | 934 | 0.535 |
| ENSP00000347197 | C5AR1 | 4.68E-05 | 0.025 | 927 | 0.651 |
| ENSP00000264563 | IL11 | 3.21E-05 | 0.025 | 926 | 0.739 |
| ENSP00000353483 | MAPK8 | 1.14E-04 | 0.025 | 925 | 0.717 |
| ENSP00000229022 | VDR | 7.51E-05 | 0.025 | 920 | 0.697 |
| ENSP00000292144 | CD3G | 3.97E-05 | 0.025 | 916 | 0.379 |
| ENSP00000200307 | CCL7 | 2.94E-05 | 0.025 | 908 | 0.677 |
| ENSP00000355988 | IRF6 | 6.10E-05 | 0.025 | 904 | 0.516 |
| ENSP00000279441 | MMP10 | 4.32E-05 | 0.025 | 888 | 0.419 |
| ENSP00000239940 | PFN2 | 5.41E-05 | 0.025 | 859 | 0.378 |
| ENSP00000305958 | STIP1 | 4.49E-05 | 0.025 | 859 | 0.218 |
| ENSP00000316333 | CD55 | 5.07E-05 | 0.025 | 817 | 0.539 |
| ENSP00000416448 | HLA-DOA | 2.11E-05 | 0.025 | 800 | 0.237 |
| ENSP00000324302 | STXBP6 | 3.06E-05 | 0.025 | 790 | 0.126 |
| ENSP00000262932 | CNPY4 | 1.07E-05 | 0.025 | 782 | 0.279 |
| ENSP00000349493 | GUCA2A | 3.95E-05 | 0.025 | 752 | 0.365 |
| ENSP00000372746 | HLA-DRA | 6.62E-05 | 0.025 | 623 | 0.420 |
| ENSP00000176195 | SCT | 5.66E-05 | 0.025 | 621 | 0.388 |
| ENSP00000225844 | CCL13 | 4.07E-05 | 0.025 | 615 | 0.748 |
| ENSP00000262888 | KCNN4 | 3.85E-05 | 0.025 | 534 | 0.585 |
| ENSP00000412297 | PPP1R11 | 3.18E-05 | 0.025 | 430 | 0.100 |
| ENSP00000295718 | PTPRN | 6.80E-05 | 0.025 | 428 | 0.270 |
| ENSP00000274629 | KCNMB1 | 3.45E-05 | 0.025 | 423 | 0.354 |
| ENSP00000387477 | PRRC2A | 6.76E-05 | 0.025 | 362 | 0.125 |
| ENSP00000364396 | TNXB | 3.75E-05 | 0.025 | 315 | 0.283 |
| ENSP00000347032 | PIP4K2C | 4.17E-05 | 0.025 | 308 | 0.242 |
| ENSP00000415328 | TRIM26 | 3.06E-05 | 0.025 | 294 | 0.323 |
| ENSP00000338260 | APOL4 | 2.77E-05 | 0.025 | 263 | 0.259 |
| ENSP00000380727 | TMEM213 | 1.17E-05 | 0.025 | 243 | 0.240 |
| ENSP00000376410 | INF2 | 2.41E-05 | 0.025 | 216 | 0.378 |
| ENSP00000299166 | NDUFB8 | 5.32E-05 | 0.026 | 957 | 0.560 |
| ENSP00000368699 | ISG15 | 6.45E-05 | 0.026 | 925 | 0.583 |
| ENSP00000302234 | CCL11 | 3.48E-05 | 0.026 | 907 | 0.663 |
| ENSP00000359425 | KCNQ5 | 5.20E-05 | 0.026 | 904 | 0.761 |
| ENSP00000351908 | MAP3K5 | 6.40E-05 | 0.026 | 895 | 0.554 |
| ENSP00000324806 | GSK3B | 1.06E-04 | 0.026 | 888 | 0.636 |
| ENSP00000215631 | GADD45B | 3.73E-05 | 0.026 | 865 | 0.631 |
| ENSP00000307786 | CYCS | 1.10E-04 | 0.026 | 848 | 0.770 |
| ENSP00000291700 | S100B | 4.95E-05 | 0.026 | 844 | 0.535 |
| ENSP00000410443 | HLA-DRA | 4.98E-05 | 0.026 | 822 | 0.420 |
| ENSP00000262613 | SLC9A3R1 | 5.17E-05 | 0.026 | 734 | 0.507 |
| ENSP00000365016 | IRS2 | 6.11E-05 | 0.026 | 723 | 0.593 |
| ENSP00000217182 | EEF1A2 | 3.68E-05 | 0.026 | 655 | 0.457 |
| ENSP00000215838 | TCN2 | 3.70E-05 | 0.026 | 653 | 0.551 |
| ENSP00000350348 | GRM7 | 4.62E-05 | 0.026 | 636 | 0.237 |
| ENSP00000304822 | CSN3 | 4.74E-05 | 0.026 | 609 | 0.580 |
| ENSP00000028008 | RNASET2 | 6.37E-05 | 0.026 | 462 | 0.201 |
| ENSP00000389155 | ABHD16A | 3.65E-05 | 0.026 | 406 | 0.119 |
| ENSP00000363640 | PHF1 | 3.19E-05 | 0.026 | 373 | 0.112 |
| ENSP00000329507 | CD300C | 3.11E-05 | 0.026 | 308 | 0.174 |
| ENSP00000256119 | CA1 | 2.71E-05 | 0.026 | 291 | 0.356 |
| ENSP00000349960 | ACTB | 1.38E-04 | 0.027 | 919 | 0.677 |
| ENSP00000289153 | PIK3CB | 7.72E-05 | 0.027 | 916 | 0.737 |
| ENSP00000342070 | CTSB | 6.62E-05 | 0.027 | 908 | 0.640 |
| ENSP00000225655 | PFN1 | 6.09E-05 | 0.027 | 859 | 0.376 |
| ENSP00000370125 | HMGN1 | 3.03E-05 | 0.027 | 808 | 0.248 |
| ENSP00000410390 | HLA-DOB | 2.11E-05 | 0.027 | 800 | 0.307 |
| ENSP00000296435 | CAMP | 6.98E-05 | 0.027 | 772 | 0.580 |
| ENSP00000365465 | CDSN | 6.44E-05 | 0.027 | 554 | 0.383 |
| ENSP00000364831 | CLCNKB | 3.45E-05 | 0.027 | 469 | 0.545 |
| ENSP00000399259 | FCHSD1 | 4.35E-05 | 0.027 | 466 | 0.300 |
| ENSP00000354575 | LSR | 2.65E-05 | 0.027 | 433 | 0.499 |
| ENSP00000360217 | RHAG | 4.17E-05 | 0.027 | 412 | 0.232 |
| ENSP00000244061 | RNF114 | 3.51E-05 | 0.027 | 401 | 0.296 |
| ENSP00000340477 | TMEM179 | 1.81E-05 | 0.027 | 374 | 0.157 |
| ENSP00000222305 | USF2 | 2.84E-05 | 0.027 | 344 | 0.396 |
| ENSP00000350815 | NR3C2 | 4.53E-05 | 0.027 | 306 | 0.413 |
| ENSP00000290524 | RFX5 | 5.49E-05 | 0.027 | 305 | 0.222 |
| ENSP00000279544 | KLRF1 | 3.05E-05 | 0.027 | 262 | 0.509 |
| ENSP00000217426 | AHCY | 4.22E-05 | 0.028 | 964 | 0.394 |
| ENSP00000351255 | STAT4 | 5.99E-05 | 0.028 | 954 | 0.747 |
| ENSP00000315130 | CLU | 5.42E-05 | 0.028 | 924 | 0.582 |
| ENSP00000265969 | KCNC1 | 5.27E-05 | 0.028 | 913 | 0.763 |
| ENSP00000394624 | OPRM1 | 5.28E-05 | 0.028 | 908 | 0.493 |
| ENSP00000257981 | KCNH3 | 5.18E-05 | 0.028 | 906 | 0.569 |
| ENSP00000343325 | PKN1 | 6.70E-05 | 0.028 | 866 | 0.558 |
| ENSP00000328207 | TNFRSF18 | 2.60E-05 | 0.028 | 790 | 0.669 |
| ENSP00000301407 | CGB1 | 1.43E-05 | 0.028 | 735 | 0.089 |
| ENSP00000000412 | M6PR | 5.17E-05 | 0.028 | 670 | 0.332 |
| ENSP00000267169 | DIABLO | 3.08E-05 | 0.028 | 609 | 0.499 |
| ENSP00000387122 | CLEC16A | 5.48E-05 | 0.028 | 516 | 0.405 |
| ENSP00000238607 | PGF | 4.12E-05 | 0.028 | 508 | 0.610 |
| ENSP00000327890 | IL3RA | 2.65E-05 | 0.028 | 428 | 0.602 |
| ENSP00000339587 | DFNA5 | 4.65E-05 | 0.028 | 427 | 0.293 |
| ENSP00000008938 | PGLYRP1 | 5.53E-05 | 0.028 | 424 | 0.595 |
| ENSP00000358358 | GJA10 | 2.99E-05 | 0.028 | 396 | 0.615 |
| ENSP00000331172 | CD8B | 5.33E-05 | 0.028 | 318 | 0.474 |
| ENSP00000358966 | IMPG1 | 2.76E-05 | 0.028 | 259 | 0.141 |
| ENSP00000270202 | AKT1 | 2.43E-04 | 0.029 | 997 | 0.632 |
| ENSP00000354901 | CXCL9 | 6.37E-05 | 0.029 | 986 | 0.874 |
| ENSP00000375629 | LILRB2 | 6.76E-05 | 0.029 | 946 | 0.529 |
| ENSP00000361926 | CNPY3 | 2.61E-05 | 0.029 | 940 | 0.327 |
| ENSP00000343023 | SP100 | 4.75E-05 | 0.029 | 926 | 0.342 |
| ENSP00000320171 | PKM | 7.80E-05 | 0.029 | 908 | 0.511 |
| ENSP00000229812 | STK38 | 5.21E-05 | 0.029 | 900 | 0.449 |
| ENSP00000323568 | SLC2A2 | 4.86E-05 | 0.029 | 871 | 0.394 |
| ENSP00000220764 | DECR1 | 8.61E-05 | 0.029 | 839 | 0.619 |
| ENSP00000321334 | LPA | 4.54E-05 | 0.029 | 622 | 0.633 |
| ENSP00000264998 | TF | 1.15E-04 | 0.029 | 574 | 0.671 |
| ENSP00000262623 | ATP4A | 4.57E-05 | 0.029 | 514 | 0.437 |
| ENSP00000359540 | CREG1 | 4.31E-05 | 0.029 | 463 | 0.083 |
| ENSP00000344549 | CARD14 | 3.04E-05 | 0.029 | 364 | 0.269 |
| ENSP00000251377 | LILRA2 | 3.22E-05 | 0.029 | 318 | 0.551 |
| ENSP00000390497 | TRIM10 | 3.11E-05 | 0.029 | 296 | 0.359 |
| ENSP00000397351 | GRID2IP | 2.66E-05 | 0.029 | 273 | 0.386 |
| ENSP00000371067 | JAK2 | 9.74E-05 | 0.03 | 951 | 0.766 |
| ENSP00000376966 | KCNC2 | 4.50E-05 | 0.03 | 906 | 0.766 |
| ENSP00000407195 | TAPBP | 8.16E-05 | 0.03 | 896 | 0.273 |
| ENSP00000285735 | RHOC | 5.52E-05 | 0.03 | 889 | 0.253 |
| ENSP00000351497 | FCGR2B | 4.19E-05 | 0.03 | 843 | 0.656 |
| ENSP00000296585 | ITGA2 | 5.84E-05 | 0.03 | 825 | 0.516 |
| ENSP00000338019 | DNAJB2 | 3.02E-05 | 0.03 | 814 | 0.229 |
| ENSP00000409403 | LRTOMT | 2.08E-05 | 0.03 | 754 | 0.107 |
| ENSP00000357167 | FCRL3 | 3.75E-05 | 0.03 | 613 | 0.432 |
| ENSP00000379457 | FAF1 | 3.47E-05 | 0.03 | 420 | 0.150 |
| ENSP00000333193 | GJC1 | 4.53E-05 | 0.03 | 417 | 0.488 |
| ENSP00000311856 | SLC25A30 | 1.60E-05 | 0.03 | 411 | 0.672 |
| ENSP00000360262 | FGGY | 3.59E-05 | 0.03 | 408 | 0.175 |
| ENSP00000336832 | GJD3 | 5.11E-05 | 0.03 | 397 | 0.612 |
| ENSP00000296099 | UCN | 2.28E-05 | 0.03 | 360 | 0.414 |
| ENSP00000353259 | CD300A | 3.62E-05 | 0.03 | 326 | 0.312 |
| ENSP00000331111 | ZNRD1 | 3.99E-05 | 0.03 | 318 | 0.208 |
| ENSP00000381607 | GSTP1 | 5.61E-05 | 0.031 | 962 | 0.507 |
| ENSP00000305873 | CRTC2 | 2.96E-05 | 0.031 | 959 | 0.363 |
| ENSP00000326432 | CCR8 | 5.47E-05 | 0.031 | 951 | 0.612 |
| ENSP00000255030 | CRP | 1.21E-04 | 0.031 | 942 | 0.721 |
| ENSP00000414808 | PPP1R11 | 1.04E-05 | 0.031 | 899 | 0.100 |
| ENSP00000223366 | GCK | 4.52E-05 | 0.031 | 758 | 0.398 |
| ENSP00000333298 | LAMP1 | 8.00E-05 | 0.031 | 621 | 0.426 |
| ENSP00000395780 | HLA-DOB | 4.28E-05 | 0.031 | 587 | 0.307 |
| ENSP00000302517 | HLA-DRB3 | 2.81E-05 | 0.031 | 573 | 0.283 |
| ENSP00000388386 | CDSN | 6.59E-05 | 0.031 | 532 | 0.383 |
| ENSP00000262539 | PTPN3 | 5.57E-05 | 0.031 | 508 | 0.607 |
| ENSP00000361202 | IRS4 | 5.62E-05 | 0.031 | 490 | 0.282 |
| ENSP00000356399 | CFH | 5.28E-05 | 0.031 | 380 | 0.515 |
| ENSP00000292401 | AZGP1 | 4.76E-05 | 0.031 | 374 | 0.315 |
| ENSP00000358188 | NBPF15 | 1.16E-05 | 0.031 | 367 | 0.257 |
| ENSP00000337773 | NQO2 | 2.98E-05 | 0.031 | 303 | 0.161 |
| ENSP00000222286 | GAPDHS | 3.62E-05 | 0.031 | 295 | 0.281 |
| ENSP00000272065 | ACP1 | 3.97E-05 | 0.031 | 240 | 0.220 |
| ENSP00000250377 | KIAA0391 | 2.19E-05 | 0.031 | 195 | 0.242 |
| ENSP00000295897 | ALB | 4.28E-04 | 0.032 | 968 | 0.767 |
| ENSP00000319635 | CXCR2 | 5.64E-05 | 0.032 | 958 | 0.775 |
| ENSP00000262445 | MAP2K4 | 6.21E-05 | 0.032 | 952 | 0.527 |
| ENSP00000347847 | MKL1 | 2.21E-05 | 0.032 | 914 | 0.234 |
| ENSP00000375892 | AKT2 | 9.62E-05 | 0.032 | 906 | 0.534 |
| ENSP00000341170 | PTN | 5.62E-05 | 0.032 | 871 | 0.453 |
| ENSP00000310880 | MAL | 2.17E-05 | 0.032 | 848 | 0.189 |
| ENSP00000370109 | PSIP1 | 4.79E-05 | 0.032 | 811 | 0.249 |
| ENSP00000261448 | CASQ2 | 3.58E-05 | 0.032 | 609 | 0.679 |
| ENSP00000405108 | HLA-DOB | 4.30E-05 | 0.032 | 587 | 0.307 |
| ENSP00000317997 | DUOX1 | 1.41E-05 | 0.032 | 534 | 0.470 |
| ENSP00000358092 | PRDM1 | 4.25E-05 | 0.032 | 504 | 0.438 |
| ENSP00000402278 | C2 | 3.99E-05 | 0.032 | 438 | 0.511 |
| ENSP00000312673 | GH1 | 4.13E-05 | 0.032 | 424 | 0.482 |
| ENSP00000337854 | HEXDC | 2.05E-05 | 0.032 | 411 | 0.547 |
| ENSP00000370543 | SLC5A3 | 2.44E-05 | 0.032 | 374 | 0.235 |
| ENSP00000329212 | ATF7 | 3.26E-05 | 0.032 | 305 | 0.138 |
| ENSP00000395598 | TRIM10 | 3.09E-05 | 0.032 | 296 | 0.359 |
| ENSP00000391879 | TRIM26 | 3.06E-05 | 0.032 | 294 | 0.323 |
| ENSP00000411321 | HLA-DMB | 1.99E-05 | 0.032 | 160 | 0.410 |
| ENSP00000344460 | CBS | 6.05E-05 | 0.033 | 973 | 0.505 |
| ENSP00000312286 | PLA2G1B | 7.65E-05 | 0.033 | 916 | 0.679 |
| ENSP00000343318 | B3GALT5 | 4.37E-05 | 0.033 | 899 | 0.189 |
| ENSP00000314897 | ANGPT2 | 3.41E-05 | 0.033 | 867 | 0.650 |
| ENSP00000386161 | WWOX | 5.33E-05 | 0.033 | 864 | 0.389 |
| ENSP00000276914 | PLIN2 | 4.06E-05 | 0.033 | 861 | 0.305 |
| ENSP00000381237 | HM13 | 6.90E-05 | 0.033 | 822 | 0.022 |
| ENSP00000233057 | EIF2AK2 | 6.85E-05 | 0.033 | 631 | 0.475 |
| ENSP00000262177 | DNAJB6 | 3.00E-05 | 0.033 | 599 | 0.070 |
| ENSP00000278175 | ADM | 5.19E-05 | 0.033 | 505 | 0.622 |
| ENSP00000363079 | MBL2 | 5.72E-05 | 0.033 | 497 | 0.623 |
| ENSP00000225371 | EPX | 1.65E-05 | 0.033 | 412 | 0.437 |
| ENSP00000258317 | NPL | 5.35E-05 | 0.033 | 408 | 0.347 |
| ENSP00000290374 | GJD2 | 3.55E-05 | 0.033 | 406 | 0.396 |
| ENSP00000334122 | FGF3 | 4.27E-05 | 0.033 | 268 | 0.519 |
| ENSP00000348912 | ADAM33 | 3.64E-05 | 0.033 | 263 | 0.447 |
| ENSP00000360157 | FOXD3 | 4.03E-05 | 0.033 | 260 | 0.489 |
| ENSP00000373854 | ITGAD | 1.81E-05 | 0.033 | 260 | 0.154 |
| ENSP00000231509 | NR3C1 | 8.16E-05 | 0.034 | 994 | 0.794 |
| ENSP00000261267 | LYZ | 1.06E-04 | 0.034 | 993 | 0.644 |
| ENSP00000287600 | PDE6D | 2.33E-05 | 0.034 | 965 | 0.344 |
| ENSP00000314520 | KCNA2 | 5.33E-05 | 0.034 | 915 | 0.702 |
| ENSP00000298171 | TSHR | 5.85E-05 | 0.034 | 912 | 0.426 |
| ENSP00000282096 | PDE3B | 3.86E-05 | 0.034 | 910 | 0.332 |
| ENSP00000358784 | KCNA3 | 4.13E-05 | 0.034 | 904 | 0.658 |
| ENSP00000371328 | KLRC3 | 4.79E-05 | 0.034 | 829 | 0.228 |
| ENSP00000304915 | IL13 | 4.16E-05 | 0.034 | 647 | 0.724 |
| ENSP00000247153 | CFP | 3.57E-05 | 0.034 | 468 | 0.478 |
| ENSP00000246949 | DNASE1 | 3.15E-05 | 0.034 | 374 | 0.462 |
| ENSP00000299642 | CLEC3A | 3.32E-05 | 0.034 | 371 | 0.539 |
| ENSP00000360560 | TCTE1 | 2.66E-05 | 0.034 | 297 | 0.060 |
| ENSP00000261833 | CIT | 2.75E-05 | 0.034 | 247 | 0.239 |
| ENSP00000283243 | PLA2R1 | 3.29E-05 | 0.034 | 235 | 0.127 |
| ENSP00000312081 | SSH3 | 1.93E-05 | 0.034 | 228 | 0.234 |
| ENSP00000354394 | STAT1 | 1.15E-04 | 0.035 | 994 | 0.784 |
| ENSP00000304895 | IRS1 | 8.03E-05 | 0.035 | 994 | 0.708 |
| ENSP00000221452 | RELB | 4.56E-05 | 0.035 | 986 | 0.778 |
| ENSP00000368880 | FOXO1 | 5.23E-05 | 0.035 | 958 | 0.651 |
| ENSP00000328511 | KCNA4 | 5.44E-05 | 0.035 | 909 | 0.804 |
| ENSP00000223642 | C5 | 4.31E-05 | 0.035 | 905 | 0.603 |
| ENSP00000305941 | USH2A | 5.04E-05 | 0.035 | 898 | 0.165 |
| ENSP00000264664 | FGF10 | 6.24E-05 | 0.035 | 867 | 0.707 |
| ENSP00000229277 | ENO2 | 6.83E-05 | 0.035 | 833 | 0.519 |
| ENSP00000261170 | GUCY2C | 6.16E-05 | 0.035 | 833 | 0.930 |
| ENSP00000234347 | PRTN3 | 4.43E-05 | 0.035 | 804 | 0.608 |
| ENSP00000255226 | SLC14A2 | 4.65E-05 | 0.035 | 755 | 0.472 |
| ENSP00000348573 | AKAP9 | 5.30E-05 | 0.035 | 722 | 0.477 |
| ENSP00000274695 | CDKAL1 | 4.32E-05 | 0.035 | 663 | 0.243 |
| ENSP00000402316 | TAP1 | 5.20E-05 | 0.035 | 530 | 0.371 |
| ENSP00000237380 | MED28 | 2.75E-05 | 0.035 | 462 | 0.532 |
| ENSP00000299367 | C2 | 3.97E-05 | 0.035 | 433 | 0.511 |
| ENSP00000349959 | RICTOR | 5.72E-05 | 0.035 | 430 | 0.438 |
| ENSP00000271348 | GJA5 | 5.35E-05 | 0.035 | 412 | 0.503 |
| ENSP00000338812 | C1QTNF6 | 4.49E-05 | 0.035 | 319 | 0.341 |
| ENSP00000370088 | AMELX | 5.01E-05 | 0.035 | 305 | 0.203 |
| ENSP00000267890 | TTBK2 | 2.62E-05 | 0.035 | 273 | 0.064 |
| ENSP00000379908 | ICA1 | 4.76E-05 | 0.035 | 260 | 0.299 |
| ENSP00000351170 | GDA | 2.93E-05 | 0.035 | 257 | 0.191 |
| ENSP00000221459 | LIN7B | 2.15E-05 | 0.035 | 240 | 0.121 |
| ENSP00000296154 | CASR | 6.73E-05 | 0.036 | 978 | 0.526 |
| ENSP00000396308 | DHFR | 5.51E-05 | 0.036 | 950 | 0.433 |
| ENSP00000368683 | EDN1 | 7.49E-05 | 0.036 | 947 | 0.766 |
| ENSP00000354566 | CD3E | 5.81E-05 | 0.036 | 929 | 0.662 |
| ENSP00000382166 | CX3CR1 | 4.08E-05 | 0.036 | 916 | 0.673 |
| ENSP00000287295 | AIFM1 | 6.17E-05 | 0.036 | 910 | 0.486 |
| ENSP00000396538 | NFAT5 | 1.04E-04 | 0.036 | 890 | 0.606 |
| ENSP00000365991 | DNAJC3 | 3.72E-05 | 0.036 | 814 | 0.239 |
| ENSP00000319308 | RGS5 | 3.48E-05 | 0.036 | 800 | 0.257 |
| ENSP00000318480 | YME1L1 | 7.46E-05 | 0.036 | 659 | 0.518 |
| ENSP00000297784 | TMC1 | 5.63E-05 | 0.036 | 502 | 0.367 |
| ENSP00000273951 | GC | 3.07E-05 | 0.036 | 462 | 0.469 |
| ENSP00000355675 | GJC2 | 5.26E-05 | 0.036 | 394 | 0.423 |
| ENSP00000296882 | GJB7 | 2.59E-05 | 0.036 | 371 | 0.624 |
| ENSP00000216194 | ADSL | 5.99E-05 | 0.036 | 329 | 0.336 |
| ENSP00000317337 | CD300LB | 3.25E-05 | 0.036 | 328 | 0.066 |
| ENSP00000306190 | SLAMF1 | 3.09E-05 | 0.036 | 302 | 0.489 |
| ENSP00000251973 | CARD10 | 2.91E-05 | 0.036 | 247 | 0.269 |
| ENSP00000225519 | SHPK | 2.74E-05 | 0.036 | 223 | 0.180 |
| ENSP00000227507 | CCND1 | 1.42E-04 | 0.037 | 999 | 0.806 |
| ENSP00000360798 | EPS15 | 8.52E-05 | 0.037 | 980 | 0.268 |
| ENSP00000220751 | RIPK2 | 4.90E-05 | 0.037 | 929 | 0.662 |
| ENSP00000346103 | GPX4 | 4.93E-05 | 0.037 | 919 | 0.886 |
| ENSP00000317714 | STX4 | 5.56E-05 | 0.037 | 917 | 0.286 |
| ENSP00000303242 | ITGB2 | 8.10E-05 | 0.037 | 913 | 0.708 |
| ENSP00000368730 | PDE7A | 2.39E-05 | 0.037 | 912 | 0.422 |
| ENSP00000358430 | TECTB | 3.71E-05 | 0.037 | 778 | 0.350 |
| ENSP00000318687 | HSPH1 | 3.93E-05 | 0.037 | 619 | 0.159 |
| ENSP00000259089 | BLK | 5.36E-05 | 0.037 | 463 | 0.485 |
| ENSP00000357060 | ATP1A4 | 3.81E-05 | 0.037 | 462 | 0.291 |
| ENSP00000222330 | GSK3A | 5.03E-05 | 0.037 | 393 | 0.468 |
| ENSP00000261292 | LIPG | 4.90E-05 | 0.037 | 373 | 0.578 |
| ENSP00000231188 | GRM6 | 4.41E-05 | 0.037 | 352 | 0.133 |
| ENSP00000158762 | ACAP1 | 4.47E-05 | 0.037 | 340 | 0.182 |
| ENSP00000323280 | CD6 | 3.48E-05 | 0.037 | 306 | 0.499 |
| ENSP00000392726 | POM121L2 | 3.01E-05 | 0.037 | 228 | 0.093 |
| ENSP00000222307 | KXD1 | 4.86E-05 | 0.037 | 203 | 0.338 |
| ENSP00000358997 | IRAK1 | 7.49E-05 | 0.038 | 999 | 0.739 |
| ENSP00000341268 | TRADD | 4.62E-05 | 0.038 | 999 | 0.658 |
| ENSP00000329890 | DEFA5 | 5.31E-05 | 0.038 | 950 | 0.557 |
| ENSP00000296026 | CXCL3 | 3.61E-05 | 0.038 | 946 | 0.667 |
| ENSP00000327850 | NFATC1 | 3.87E-05 | 0.038 | 920 | 0.577 |
| ENSP00000256646 | NOTCH2 | 6.06E-05 | 0.038 | 905 | 0.869 |
| ENSP00000229264 | GNB3 | 5.20E-05 | 0.038 | 800 | 0.334 |
| ENSP00000357103 | DARC | 4.46E-05 | 0.038 | 735 | 0.483 |
| ENSP00000263946 | PKP1 | 3.26E-05 | 0.038 | 573 | 0.319 |
| ENSP00000340811 | GJB5 | 4.99E-05 | 0.038 | 461 | 0.588 |
| ENSP00000383746 | FCAMR | 3.69E-05 | 0.038 | 329 | 0.149 |
| ENSP00000002165 | FUCA2 | 1.55E-05 | 0.038 | 315 | 0.422 |
| ENSP00000317674 | APOL1 | 2.93E-05 | 0.038 | 260 | 0.356 |
| ENSP00000296027 | CXCL5 | 3.52E-05 | 0.039 | 958 | 0.746 |
| ENSP00000401548 | TCF19 | 5.43E-05 | 0.039 | 938 | 0.257 |
| ENSP00000318212 | KCNH6 | 4.24E-05 | 0.039 | 926 | 0.574 |
| ENSP00000329991 | IFNL1 | 3.64E-05 | 0.039 | 924 | 0.687 |
| ENSP00000366124 | CST3 | 4.09E-05 | 0.039 | 919 | 0.549 |
| ENSP00000366563 | PIK3CD | 7.39E-05 | 0.039 | 916 | 0.725 |
| ENSP00000333657 | MX2 | 3.91E-05 | 0.039 | 907 | 0.469 |
| ENSP00000315011 | EDNRA | 5.44E-05 | 0.039 | 906 | 0.590 |
| ENSP00000345708 | KCNJ11 | 5.22E-05 | 0.039 | 818 | 0.441 |
| ENSP00000409132 | HLA-G | 3.83E-05 | 0.039 | 805 | 0.647 |
| ENSP00000281317 | MMAA | 3.63E-05 | 0.039 | 675 | 0.240 |
| ENSP00000260630 | CYP1B1 | 6.80E-05 | 0.039 | 659 | 0.623 |
| ENSP00000354159 | NLRC4 | 3.43E-05 | 0.039 | 485 | 0.717 |
| ENSP00000402406 | PSMB8 | 4.22E-05 | 0.039 | 460 | 0.268 |
| ENSP00000379282 | ABHD16A | 3.69E-05 | 0.039 | 406 | 0.119 |
| ENSP00000216027 | HSCB | 2.83E-05 | 0.039 | 292 | 0.162 |
| ENSP00000304236 | CD14 | 5.92E-05 | 0.04 | 986 | 0.695 |
| ENSP00000229794 | MAPK14 | 1.17E-04 | 0.04 | 971 | 0.758 |
| ENSP00000320866 | CALR | 1.49E-04 | 0.04 | 956 | 0.496 |
| ENSP00000352721 | DNM2 | 6.55E-05 | 0.04 | 933 | 0.491 |
| ENSP00000261740 | TRPV4 | 4.70E-05 | 0.04 | 930 | 0.402 |
| ENSP00000381599 | MX1 | 5.24E-05 | 0.04 | 919 | 0.581 |
| ENSP00000261623 | CYBA | 3.15E-05 | 0.04 | 847 | 0.551 |
| ENSP00000409910 | HLA-E | 1.92E-05 | 0.04 | 800 | 0.707 |
| ENSP00000261405 | VWF | 8.31E-05 | 0.04 | 691 | 0.639 |
| ENSP00000338814 | BAG5 | 3.30E-05 | 0.04 | 476 | 0.195 |
| ENSP00000419945 | ERVW-1 | 6.69E-05 | 0.04 | 465 | 0.558 |
| ENSP00000274026 | CCNA2 | 6.10E-05 | 0.04 | 415 | 0.494 |
| ENSP00000368189 | ARID3C | 1.01E-05 | 0.04 | 336 | 0.077 |
| ENSP00000273183 | STAC | 3.45E-05 | 0.04 | 317 | 0.303 |
| ENSP00000260818 | DNAJC13 | 2.72E-05 | 0.04 | 315 | 0.079 |
| ENSP00000340292 | DLK1 | 4.87E-05 | 0.04 | 289 | 0.557 |
| ENSP00000410512 | HLA-DQB2 | 2.80E-05 | 0.04 | 223 | 0.294 |
| ENSP00000357470 | IL6R | 3.01E-05 | 0.041 | 925 | 0.706 |
| ENSP00000268097 | HEXA | 6.85E-05 | 0.041 | 923 | 0.397 |
| ENSP00000323479 | B3GALNT1 | 4.44E-05 | 0.041 | 909 | 0.320 |
| ENSP00000386439 | SYNC | 1.93E-05 | 0.041 | 891 | 0.291 |
| ENSP00000256999 | FOLH1 | 3.57E-05 | 0.041 | 823 | 0.343 |
| ENSP00000357283 | LMNA | 8.02E-05 | 0.041 | 723 | 0.544 |
| ENSP00000264954 | GRPEL1 | 4.38E-05 | 0.041 | 705 | 0.310 |
| ENSP00000306157 | IL7R | 3.53E-05 | 0.041 | 609 | 0.761 |
| ENSP00000264497 | IL21 | 1.85E-05 | 0.041 | 596 | 0.696 |
| ENSP00000261917 | HCN4 | 3.79E-05 | 0.041 | 505 | 0.619 |
| ENSP00000325775 | GJC3 | 3.77E-05 | 0.041 | 437 | 0.623 |
| ENSP00000348793 | AFF3 | 4.51E-05 | 0.041 | 379 | 0.435 |
| ENSP00000357150 | CD1B | 1.86E-05 | 0.041 | 307 | 0.448 |
| ENSP00000291906 | PKN3 | 2.26E-05 | 0.041 | 247 | 0.065 |
| ENSP00000391938 | HLA-DQB2 | 2.80E-05 | 0.041 | 223 | 0.294 |
| ENSP00000373023 | CDSN | 7.03E-05 | 0.042 | 928 | 0.383 |
| ENSP00000380073 | IRF9 | 5.50E-05 | 0.042 | 918 | 0.531 |
| ENSP00000378786 | HLA-DRA | 3.86E-05 | 0.042 | 664 | 0.420 |
| ENSP00000293330 | HCRT | 4.76E-05 | 0.042 | 621 | 0.443 |
| ENSP00000262570 | CHCHD3 | 4.93E-05 | 0.042 | 620 | 0.425 |
| ENSP00000342681 | CD5 | 7.08E-05 | 0.042 | 506 | 0.717 |
| ENSP00000356954 | CTGF | 5.02E-05 | 0.042 | 499 | 0.648 |
| ENSP00000224784 | ACTA2 | 6.40E-05 | 0.042 | 447 | 0.605 |
| ENSP00000388794 | CD177 | 2.25E-05 | 0.042 | 396 | 0.218 |
| ENSP00000268876 | UNC45B | 2.98E-05 | 0.042 | 364 | 0.279 |
| ENSP00000288071 | DDX19B | 3.76E-05 | 0.042 | 340 | 0.267 |
| ENSP00000355133 | NOS1AP | 2.86E-05 | 0.042 | 318 | 0.280 |
| ENSP00000343348 | VMAC | 1.72E-05 | 0.042 | 305 | 0.418 |
| ENSP00000327336 | BGN | 5.86E-05 | 0.043 | 958 | 0.419 |
| ENSP00000363431 | NPY4R | 3.46E-05 | 0.043 | 901 | 0.742 |
| ENSP00000407879 | MYH14 | 2.51E-05 | 0.043 | 893 | 0.246 |
| ENSP00000251241 | DHX40 | 3.99E-05 | 0.043 | 790 | 0.146 |
| ENSP00000283936 | KCNJ16 | 3.63E-05 | 0.043 | 790 | 0.638 |
| ENSP00000361173 | KCNE1L | 3.26E-05 | 0.043 | 752 | 0.766 |
| ENSP00000254868 | CLEC10A | 4.91E-05 | 0.043 | 676 | 0.561 |
| ENSP00000264381 | BCHE | 4.54E-05 | 0.043 | 540 | 0.456 |
| ENSP00000368174 | MCM8 | 7.93E-05 | 0.043 | 515 | 0.589 |
| ENSP00000401317 | TUBB | 4.78E-05 | 0.043 | 344 | 0.331 |
| ENSP00000386796 | SCN7A | 2.19E-05 | 0.043 | 203 | 0.596 |
| ENSP00000341940 | CAV3 | 5.75E-05 | 0.044 | 954 | 0.569 |
| ENSP00000346206 | TAP1 | 6.22E-05 | 0.044 | 937 | 0.371 |
| ENSP00000387699 | CREB1 | 1.11E-04 | 0.044 | 907 | 0.743 |
| ENSP00000330382 | PDGFB | 5.90E-05 | 0.044 | 893 | 0.688 |
| ENSP00000351314 | PSMB10 | 4.70E-05 | 0.044 | 519 | 0.173 |
| ENSP00000371372 | ATP12A | 4.70E-05 | 0.044 | 514 | 0.436 |
| ENSP00000413625 | FNBP1 | 3.76E-05 | 0.044 | 462 | 0.181 |
| ENSP00000356033 | TAGAP | 5.87E-05 | 0.044 | 443 | 0.427 |
| ENSP00000303276 | RNASE2 | 5.69E-05 | 0.044 | 429 | 0.548 |
| ENSP00000339867 | CLCN1 | 2.59E-05 | 0.044 | 363 | 0.288 |
| ENSP00000362296 | POU3F4 | 3.08E-05 | 0.044 | 334 | 0.457 |
| ENSP00000293195 | FDXR | 3.60E-05 | 0.044 | 265 | 0.386 |
| ENSP00000337632 | SARNP | 3.32E-05 | 0.044 | 241 | 0.170 |
| ENSP00000347198 | SRGAP1 | 2.29E-05 | 0.044 | 212 | 0.212 |
| ENSP00000216214 | FAM118A | 2.59E-05 | 0.044 | 203 | 0.129 |
| ENSP00000374902 | TRBV6-9 | 2.28E-05 | 0.044 | 161 | 0.122 |
| ENSP00000227471 | UNC93B1 | 3.80E-05 | 0.045 | 947 | 0.629 |
| ENSP00000315768 | STAT2 | 6.21E-05 | 0.045 | 914 | 0.647 |
| ENSP00000319248 | ZEB1 | 3.71E-05 | 0.045 | 908 | 0.579 |
| ENSP00000266395 | PDE6H | 2.28E-05 | 0.045 | 899 | 0.230 |
| ENSP00000356946 | FCGR3A | 6.31E-05 | 0.045 | 867 | 0.679 |
| ENSP00000244336 | CEACAM8 | 1.82E-05 | 0.045 | 854 | 0.473 |
| ENSP00000250018 | TPH1 | 4.28E-05 | 0.045 | 837 | 0.348 |
| ENSP00000289429 | CD1A | 4.55E-05 | 0.045 | 803 | 0.246 |
| ENSP00000221476 | CKM | 3.91E-05 | 0.045 | 800 | 0.277 |
| ENSP00000274520 | IL9 | 3.59E-05 | 0.045 | 569 | 0.753 |
| ENSP00000245903 | CD70 | 2.27E-05 | 0.045 | 542 | 0.662 |
| ENSP00000345789 | MUM1 | 1.44E-05 | 0.045 | 462 | 0.130 |
| ENSP00000252483 | PVRL2 | 4.20E-05 | 0.045 | 286 | 0.264 |
| ENSP00000308782 | GP6 | 3.70E-05 | 0.045 | 195 | 0.375 |
| ENSP00000386935 | UAP1L1 | 1.42E-05 | 0.045 | 159 | 0.175 |
| ENSP00000346839 | FN1 | 1.36E-04 | 0.046 | 998 | 0.733 |
| ENSP00000363822 | AR | 1.21E-04 | 0.046 | 959 | 0.838 |
| ENSP00000315931 | AHCYL2 | 2.61E-05 | 0.046 | 945 | 0.349 |
| ENSP00000360215 | ZBP1 | 2.65E-05 | 0.046 | 944 | 0.589 |
| ENSP00000255040 | APCS | 3.97E-05 | 0.046 | 909 | 0.572 |
| ENSP00000287641 | SST | 8.06E-05 | 0.046 | 900 | 0.640 |
| ENSP00000308716 | INHBC | 1.78E-05 | 0.046 | 899 | 0.301 |
| ENSP00000300134 | STAT6 | 5.57E-05 | 0.046 | 878 | 0.740 |
| ENSP00000248572 | GNGT1 | 4.48E-05 | 0.046 | 800 | 0.128 |
| ENSP00000294702 | GFI1 | 3.19E-05 | 0.046 | 778 | 0.357 |
| ENSP00000285930 | AKR1B1 | 5.71E-05 | 0.046 | 639 | 0.560 |
| ENSP00000367959 | HTR2A | 4.54E-05 | 0.046 | 625 | 0.317 |
| ENSP00000328150 | KCNJ12 | 6.05E-05 | 0.046 | 605 | 0.684 |
| ENSP00000394290 | GNL1 | 3.55E-05 | 0.046 | 523 | 0.326 |
| ENSP00000298596 | STOX1 | 3.39E-05 | 0.046 | 408 | 0.181 |
| ENSP00000302441 | NANP | 1.38E-05 | 0.046 | 317 | 0.248 |
| ENSP00000259895 | GTF2H4 | 3.07E-05 | 0.046 | 296 | 0.196 |
| ENSP00000396251 | GTF2H4 | 3.07E-05 | 0.046 | 296 | 0.196 |
| ENSP00000358795 | NEURL | 3.11E-05 | 0.046 | 291 | 0.329 |
| ENSP00000249066 | APOL2 | 2.74E-05 | 0.046 | 265 | 0.268 |
| ENSP00000374014 | TM6SF2 | 1.55E-05 | 0.046 | 188 | 0.076 |
| ENSP00000268035 | IGF1R | 6.33E-05 | 0.047 | 998 | 0.694 |
| ENSP00000304697 | UBB | 1.06E-04 | 0.047 | 932 | 0.377 |
| ENSP00000354251 | NCKAP1 | 3.45E-05 | 0.047 | 912 | 0.333 |
| ENSP00000360626 | KCNG1 | 4.22E-05 | 0.047 | 901 | 0.443 |
| ENSP00000310661 | PDE7B | 2.26E-05 | 0.047 | 901 | 0.191 |
| ENSP00000219596 | MEFV | 6.03E-05 | 0.047 | 900 | 0.639 |
| ENSP00000296930 | NPM1 | 7.11E-05 | 0.047 | 897 | 0.476 |
| ENSP00000368966 | TRPC3 | 4.98E-05 | 0.047 | 875 | 0.515 |
| ENSP00000327513 | CSF1 | 2.34E-05 | 0.047 | 769 | 0.615 |
| ENSP00000248935 | GSTT1 | 3.11E-05 | 0.047 | 565 | 0.421 |
| ENSP00000159060 | NOX3 | 1.61E-05 | 0.047 | 416 | 0.499 |
| ENSP00000349365 | IL27 | 1.68E-05 | 0.047 | 414 | 0.574 |
| ENSP00000324775 | TMIE | 2.11E-05 | 0.047 | 364 | 0.374 |
| ENSP00000236938 | FCRLA | 3.12E-05 | 0.047 | 336 | 0.104 |
| ENSP00000318902 | FOXP1 | 4.95E-05 | 0.047 | 326 | 0.396 |
| ENSP00000371798 | FSCN1 | 4.18E-05 | 0.047 | 312 | 0.494 |
| ENSP00000345751 | SCNN1B | 2.07E-05 | 0.047 | 283 | 0.401 |
| ENSP00000322486 | OTOS | 1.52E-05 | 0.047 | 254 | 0.523 |
| ENSP00000408453 | HLA-DMB | 3.76E-05 | 0.047 | 232 | 0.410 |
| ENSP00000306245 | FOS | 1.92E-04 | 0.048 | 991 | 0.804 |
| ENSP00000314425 | IL12RB1 | 3.06E-05 | 0.048 | 939 | 0.678 |
| ENSP00000216160 | TAB1 | 3.34E-05 | 0.048 | 933 | 0.588 |
| ENSP00000286063 | PDE11A | 2.30E-05 | 0.048 | 916 | 0.345 |
| ENSP00000336764 | OPRL1 | 3.28E-05 | 0.048 | 900 | 0.534 |
| ENSP00000400104 | RXRB | 2.73E-05 | 0.048 | 899 | 0.295 |
| ENSP00000355941 | TATDN3 | 2.46E-05 | 0.048 | 899 | 0.212 |
| ENSP00000356515 | UTRN | 3.40E-05 | 0.048 | 850 | 0.291 |
| ENSP00000350132 | PLN | 4.90E-05 | 0.048 | 823 | 0.635 |
| ENSP00000322191 | DEFB104B | 3.72E-05 | 0.048 | 752 | 0.758 |
| ENSP00000347948 | TNFRSF14 | 4.88E-05 | 0.048 | 621 | 0.377 |
| ENSP00000312326 | AOC3 | 3.06E-05 | 0.048 | 609 | 0.654 |
| ENSP00000236826 | MMP8 | 2.91E-05 | 0.048 | 549 | 0.548 |
| ENSP00000270538 | TIMM44 | 3.67E-05 | 0.048 | 511 | 0.326 |
| ENSP00000329384 | IL22 | 2.25E-05 | 0.048 | 429 | 0.637 |
| ENSP00000263119 | CABIN1 | 2.95E-05 | 0.048 | 369 | 0.402 |
| ENSP00000298690 | RNASE7 | 1.80E-05 | 0.048 | 340 | 0.556 |
| ENSP00000268605 | NOL3 | 3.88E-05 | 0.048 | 330 | 0.495 |
| ENSP00000248070 | EPS15L1 | 2.69E-05 | 0.048 | 318 | 0.250 |
| ENSP00000364016 | PSMB8 | 4.00E-05 | 0.048 | 307 | 0.268 |
| ENSP00000339001 | TUBB | 4.84E-05 | 0.048 | 305 | 0.331 |
| ENSP00000315602 | CHRNA3 | 3.90E-05 | 0.048 | 274 | 0.223 |
| ENSP00000384273 | RELA | 1.34E-04 | 0.049 | 999 | 0.807 |
| ENSP00000333685 | MAPK11 | 7.26E-05 | 0.049 | 959 | 0.513 |
| ENSP00000344479 | NR4A2 | 4.17E-05 | 0.049 | 903 | 0.352 |
| ENSP00000308549 | ADORA1 | 3.33E-05 | 0.049 | 899 | 0.589 |
| ENSP00000357130 | SPTA1 | 4.94E-05 | 0.049 | 851 | 0.000 |
| ENSP00000274306 | GZMA | 5.12E-05 | 0.049 | 771 | 0.678 |
| ENSP00000354900 | GJB1 | 4.63E-05 | 0.049 | 619 | 0.410 |
| ENSP00000342082 | SLPI | 3.43E-05 | 0.049 | 594 | 0.591 |
| ENSP00000248594 | PTPN12 | 5.51E-05 | 0.049 | 430 | 0.488 |
| ENSP00000410071 | TUBB | 4.99E-05 | 0.049 | 297 | 0.331 |
| ENSP00000255476 | RFXAP | 2.37E-05 | 0.049 | 286 | 0.229 |
| ENSP00000269576 | KRT10 | 2.41E-05 | 0.049 | 246 | 0.175 |
