# Supplementary material for: A network-based method using a random walk with restart algorithm and screening tests to identify novel genes associated with Menière's disease
Source: PLoS One. 2017 Aug 7;12(8):e0182592. doi: 10.1371/journal.pone.0182592 (PMC5546581; doi:10.1371/journal.pone.0182592)
Supplement: S4 Table — (DOCX) [file pone.0182592.s004.docx]

**S4 Table.** The 43 inferred genes with probabilities higher than 1E-05, permutation FDRs less than 0.05, *MIS*s greater than or equal to 900 and *MES*s larger than 0.8.

| **Ensembl ID** | **Gene symbol** | **Probability** | **P-value** | **MIS** | **MFS** |
| --- | --- | --- | --- | --- | --- |
| ENSP00000258743 | IL6 | 3.62E-04 | 0.004 | 992 | 0.920 |
| ENSP00000353874 | TLR9 | 8.65E-05 | <0.001 | 927 | 0.895 |
| ENSP00000305651 | CXCL10 | 1.05E-04 | 0.002 | 994 | 0.891 |
| ENSP00000392398 | GPX5 | 8.36E-05 | 0.002 | 919 | 0.890 |
| ENSP00000260010 | TLR2 | 1.80E-04 | <0.001 | 964 | 0.888 |
| ENSP00000346103 | GPX4 | 4.93E-05 | 0.037 | 919 | 0.886 |
| ENSP00000379625 | MYD88 | 1.37E-04 | 0.001 | 999 | 0.880 |
| ENSP00000354901 | CXCL9 | 6.37E-05 | 0.029 | 986 | 0.874 |
| ENSP00000011653 | CD4 | 3.11E-04 | 0.003 | 998 | 0.870 |
| ENSP00000256646 | NOTCH2 | 6.06E-05 | 0.038 | 905 | 0.869 |
| ENSP00000233946 | IL1R1 | 8.11E-05 | 0.015 | 999 | 0.858 |
| ENSP00000280357 | IL18 | 1.21E-04 | 0.002 | 994 | 0.852 |
| ENSP00000412237 | IL10 | 2.11E-04 | <0.001 | 976 | 0.851 |
| ENSP00000356438 | PTGS2 | 1.94E-04 | 0.007 | 976 | 0.847 |
| ENSP00000225831 | CCL2 | 1.46E-04 | 0.006 | 968 | 0.841 |
| ENSP00000292303 | CCR5 | 2.01E-04 | <0.001 | 999 | 0.841 |
| ENSP00000361359 | CD40 | 2.35E-04 | <0.001 | 969 | 0.839 |
| ENSP00000363822 | AR | 1.21E-04 | 0.046 | 959 | 0.838 |
| ENSP00000264832 | ICAM1 | 2.49E-04 | <0.001 | 977 | 0.835 |
| ENSP00000306512 | IL8 | 1.82E-04 | 0.005 | 978 | 0.834 |
| ENSP00000252321 | KCNA5 | 7.39E-05 | 0.016 | 927 | 0.831 |
| ENSP00000296871 | CSF2 | 2.05E-04 | 0.004 | 977 | 0.825 |
| ENSP00000216797 | NFKBIA | 9.20E-05 | 0.023 | 999 | 0.825 |
| ENSP00000155840 | KCNQ1 | 1.30E-04 | 0.008 | 999 | 0.825 |
| ENSP00000162749 | TNFRSF1A | 1.11E-04 | 0.011 | 999 | 0.825 |
| ENSP00000320084 | CD276 | 1.21E-04 | 0.006 | 955 | 0.823 |
| ENSP00000364114 | HLA-DRB5 | 1.77E-04 | 0.001 | 923 | 0.822 |
| ENSP00000250151 | CCL4 | 6.49E-05 | 0.016 | 947 | 0.820 |
| ENSP00000294728 | VCAM1 | 1.75E-04 | <0.001 | 968 | 0.819 |
| ENSP00000264246 | CD80 | 1.49E-04 | 0.011 | 999 | 0.813 |
| ENSP00000332049 | CD86 | 2.00E-04 | <0.001 | 999 | 0.812 |
| ENSP00000226730 | IL2 | 2.08E-04 | 0.012 | 989 | 0.809 |
| ENSP00000384273 | RELA | 1.34E-04 | 0.049 | 999 | 0.807 |
| ENSP00000379110 | CXCL1 | 7.15E-05 | 0.018 | 973 | 0.807 |
| ENSP00000329411 | IRF7 | 1.32E-04 | <0.001 | 917 | 0.806 |
| ENSP00000227507 | CCND1 | 1.42E-04 | 0.037 | 999 | 0.806 |
| ENSP00000306245 | FOS | 1.92E-04 | 0.048 | 991 | 0.804 |
| ENSP00000328511 | KCNA4 | 5.44E-05 | 0.035 | 909 | 0.804 |
| ENSP00000365380 | FOXP3 | 1.71E-04 | 0.008 | 996 | 0.804 |
| ENSP00000311032 | CASP3 | 1.37E-04 | 0.006 | 997 | 0.802 |
| ENSP00000369293 | IL2RA | 1.37E-04 | 0.003 | 946 | 0.802 |
| ENSP00000264657 | STAT3 | 1.26E-04 | 0.017 | 953 | 0.802 |
| ENSP00000361405 | MMP9 | 1.27E-04 | 0.008 | 958 | 0.801 |
