# Supplementary material for: A network-based method using a random walk with restart algorithm and screening tests to identify novel genes associated with Menière's disease
Source: PLoS One. 2017 Aug 7;12(8):e0182592. doi: 10.1371/journal.pone.0182592 (PMC5546581; doi:10.1371/journal.pone.0182592)
Supplement: S5 Table — (DOCX) [file pone.0182592.s005.docx]

**S5 Table.** The interactions used for drawing Fig 2

| **Node1** | **Node2** | **Weight** |
| --- | --- | --- |
| CD4 | ESR1(ENSP00000206249) | 378 |
| CD4 | MIF(ENSP00000215754) | 420 |
| CD4 | CHGA(ENSP00000216492) | 168 |
| CD4 | TLR8(ENSP00000218032) | 506 |
| CD4 | PON1(ENSP00000222381) | 260 |
| CD4 | NFKB1(ENSP00000226574) | 290 |
| CD4 | IFNG(ENSP00000229135) | 985 |
| CD4 | LTF(ENSP00000231751) | 430 |
| CD4 | TNFAIP3(ENSP00000237289) | 190 |
| CD4 | GJB6(ENSP00000241124) | 340 |
| CD4 | IL1A(ENSP00000263339) | 542 |
| CD4 | IL1B(ENSP00000263341) | 665 |
| CD4 | NOTCH3(ENSP00000263388) | 319 |
| CD4 | CCL5(ENSP00000293272) | 659 |
| CD4 | REL(ENSP00000295025) | 540 |
| CD4 | TLR3(ENSP00000296795) | 613 |
| CD4 | DEFB1(ENSP00000297439) | 196 |
| CD4 | NOS3(ENSP00000297494) | 330 |
| CD4 | AQP3(ENSP00000297991) | 229 |
| CD4 | CTLA4(ENSP00000303939) | 853 |
| CD4 | TLR10(ENSP00000308925) | 305 |
| CD4 | AQP1(ENSP00000311165) | 186 |
| CD4 | TNIP1(ENSP00000317891) | 199 |
| CD4 | NOS2(ENSP00000327251) | 540 |
| CD4 | SOD2(ENSP00000337127) | 429 |
| CD4 | CAV1(ENSP00000339191) | 235 |
| CD4 | HLA-DQA1(ENSP00000339398) | 864 |
| CD4 | ESR2(ENSP00000343925) | 180 |
| CD4 | PTPN22(ENSP00000352833) | 319 |
| CD4 | HLA-DRB1(ENSP00000353099) | 998 |
| CD4 | PARP1(ENSP00000355759) | 424 |
| CD4 | NPR1(ENSP00000357669) | 195 |
| CD4 | FLNA(ENSP00000358866) | 374 |
| CD4 | TLR4(ENSP00000363089) | 752 |
| CD4 | HLA-C(ENSP00000365402) | 767 |
| CD4 | HLA-A(ENSP00000366005) | 690 |
| CD4 | TLR7(ENSP00000370034) | 655 |
| CD4 | HLA-DQA1(ENSP00000372738) | 347 |
| CD4 | HLA-C(ENSP00000372975) | 518 |
| CD4 | HLA-A(ENSP00000373114) | 637 |
| CD4 | HLA-C(ENSP00000383245) | 514 |
| CD4 | HLA-DQA1(ENSP00000387892) | 241 |
| CD4 | HLA-A(ENSP00000388526) | 801 |
| CD4 | HLA-A(ENSP00000388724) | 637 |
| CD4 | TNF(ENSP00000389265) | 839 |
| CD4 | HLA-C(ENSP00000390282) | 663 |
| CD4 | TNF(ENSP00000392858) | 839 |
| CD4 | HLA-C(ENSP00000397867) | 464 |
| CD4 | HLA-A(ENSP00000398188) | 661 |
| CD4 | TNF(ENSP00000398698) | 863 |
| CD4 | HLA-B(ENSP00000399168) | 950 |
| CD4 | HLA-B(ENSP00000400842) | 680 |
| CD4 | HLA-DQA1(ENSP00000401760) | 238 |
| CD4 | MICA(ENSP00000402134) | 299 |
| CD4 | HSPA1A(ENSP00000404524) | 241 |
| CD4 | HSPA1A(ENSP00000406359) | 246 |
| CD4 | GPX1(ENSP00000407375) | 271 |
| CD4 | HLA-C(ENSP00000407431) | 562 |
| CD4 | HLA-DQA1(ENSP00000409127) | 249 |
| CD4 | HLA-A(ENSP00000410645) | 691 |
| CD4 | HLA-C(ENSP00000413992) | 767 |
| CD4 | HLA-DQA1(ENSP00000414360) | 238 |
| CD4 | HLA-A(ENSP00000416233) | 635 |
| KCNQ1 | AQP2(ENSP00000199280) | 182 |
| KCNQ1 | GJB6(ENSP00000241124) | 196 |
| KCNQ1 | MTNR1B(ENSP00000257068) | 510 |
| KCNQ1 | KCNQ4(ENSP00000262916) | 908 |
| KCNQ1 | REL(ENSP00000295025) | 201 |
| KCNQ1 | AQP3(ENSP00000297991) | 180 |
| KCNQ1 | KCNE3(ENSP00000310557) | 954 |
| KCNQ1 | SLC8A1(ENSP00000332931) | 306 |
| KCNQ1 | KCNE1(ENSP00000337255) | 999 |
| KCNQ1 | CACNA1A(ENSP00000353362) | 202 |
| KCNQ1 | MTR(ENSP00000355536) | 201 |
| KCNQ1 | GJB3(ENSP00000362460) | 193 |
| KCNQ1 | HLA-A(ENSP00000366005) | 195 |
| KCNQ1 | AQP4(ENSP00000372654) | 201 |
| KCNQ1 | HLA-A(ENSP00000373114) | 199 |
| KCNQ1 | HLA-A(ENSP00000388526) | 187 |
| KCNQ1 | HLA-A(ENSP00000388724) | 195 |
| KCNQ1 | HLA-A(ENSP00000398188) | 195 |
| KCNQ1 | HLA-B(ENSP00000399168) | 193 |
| KCNQ1 | HLA-B(ENSP00000400842) | 193 |
| KCNQ1 | HLA-A(ENSP00000410645) | 193 |
| KCNQ1 | HLA-A(ENSP00000416233) | 199 |
| TNFRSF1A | ESR1(ENSP00000206249) | 240 |
| TNFRSF1A | MIF(ENSP00000215754) | 274 |
| TNFRSF1A | TLR8(ENSP00000218032) | 260 |
| TNFRSF1A | NFKB1(ENSP00000226574) | 932 |
| TNFRSF1A | IFNG(ENSP00000229135) | 505 |
| TNFRSF1A | TNFAIP3(ENSP00000237289) | 971 |
| TNFRSF1A | IL1A(ENSP00000263339) | 424 |
| TNFRSF1A | IL1B(ENSP00000263341) | 540 |
| TNFRSF1A | CCL5(ENSP00000293272) | 347 |
| TNFRSF1A | REL(ENSP00000295025) | 518 |
| TNFRSF1A | TLR3(ENSP00000296795) | 488 |
| TNFRSF1A | NOS3(ENSP00000297494) | 252 |
| TNFRSF1A | CTLA4(ENSP00000303939) | 540 |
| TNFRSF1A | NOS2(ENSP00000327251) | 859 |
| TNFRSF1A | SOD2(ENSP00000337127) | 378 |
| TNFRSF1A | CAV1(ENSP00000339191) | 964 |
| TNFRSF1A | ESR2(ENSP00000343925) | 154 |
| TNFRSF1A | HLA-DRB1(ENSP00000353099) | 229 |
| TNFRSF1A | PARP1(ENSP00000355759) | 497 |
| TNFRSF1A | TLR4(ENSP00000363089) | 611 |
| TNFRSF1A | HSPA1A(ENSP00000364802) | 202 |
| TNFRSF1A | HLA-C(ENSP00000365402) | 249 |
| TNFRSF1A | HLA-A(ENSP00000366005) | 170 |
| TNFRSF1A | TLR7(ENSP00000370034) | 374 |
| TNFRSF1A | HLA-DQA1(ENSP00000372738) | 177 |
| TNFRSF1A | HLA-A(ENSP00000388526) | 227 |
| TNFRSF1A | TNF(ENSP00000389265) | 810 |
| TNFRSF1A | TNF(ENSP00000392858) | 999 |
| TNFRSF1A | TNF(ENSP00000398698) | 999 |
| TNFRSF1A | HLA-B(ENSP00000399168) | 307 |
| TNFRSF1A | HSPA1A(ENSP00000404524) | 167 |
| TNFRSF1A | HSPA1A(ENSP00000406359) | 204 |
| TNFRSF1A | GPX1(ENSP00000407375) | 180 |
| TNFRSF1A | HLA-A(ENSP00000410645) | 201 |
| TNFRSF1A | TNF(ENSP00000410668) | 800 |
| TNFRSF1A | HLA-C(ENSP00000413992) | 176 |
| NFKBIA | ESR1(ENSP00000206249) | 343 |
| NFKBIA | MIF(ENSP00000215754) | 219 |
| NFKBIA | TLR8(ENSP00000218032) | 243 |
| NFKBIA | NFKB1(ENSP00000226574) | 999 |
| NFKBIA | IFNG(ENSP00000229135) | 505 |
| NFKBIA | TNFAIP3(ENSP00000237289) | 877 |
| NFKBIA | VHL(ENSP00000256474) | 177 |
| NFKBIA | IL1A(ENSP00000263339) | 438 |
| NFKBIA | IL1B(ENSP00000263341) | 955 |
| NFKBIA | SIK1(ENSP00000270162) | 150 |
| NFKBIA | CCL5(ENSP00000293272) | 315 |
| NFKBIA | REL(ENSP00000295025) | 995 |
| NFKBIA | TLR3(ENSP00000296795) | 933 |
| NFKBIA | NOS3(ENSP00000297494) | 319 |
| NFKBIA | TLR10(ENSP00000308925) | 177 |
| NFKBIA | TNIP1(ENSP00000317891) | 434 |
| NFKBIA | NOS2(ENSP00000327251) | 412 |
| NFKBIA | SOD2(ENSP00000337127) | 603 |
| NFKBIA | PARP1(ENSP00000355759) | 379 |
| NFKBIA | TLR4(ENSP00000363089) | 543 |
| NFKBIA | HSPA1A(ENSP00000364802) | 470 |
| NFKBIA | HLA-C(ENSP00000365402) | 169 |
| NFKBIA | TLR7(ENSP00000370034) | 306 |
| NFKBIA | TNF(ENSP00000389265) | 609 |
| NFKBIA | TNF(ENSP00000392858) | 611 |
| NFKBIA | TNF(ENSP00000398698) | 752 |
| NFKBIA | HLA-B(ENSP00000399168) | 184 |
| NFKBIA | HSPA1A(ENSP00000404524) | 273 |
| NFKBIA | HSPA1A(ENSP00000406359) | 317 |
| NFKBIA | GPX1(ENSP00000407375) | 274 |
| CCL2 | ESR1(ENSP00000206249) | 306 |
| CCL2 | MIF(ENSP00000215754) | 501 |
| CCL2 | TLR8(ENSP00000218032) | 330 |
| CCL2 | PON1(ENSP00000222381) | 266 |
| CCL2 | PON2(ENSP00000222572) | 191 |
| CCL2 | NFKB1(ENSP00000226574) | 296 |
| CCL2 | IFNG(ENSP00000229135) | 659 |
| CCL2 | LTF(ENSP00000231751) | 266 |
| CCL2 | TNFAIP3(ENSP00000237289) | 341 |
| CCL2 | IL1A(ENSP00000263339) | 698 |
| CCL2 | IL1B(ENSP00000263341) | 962 |
| CCL2 | CCL5(ENSP00000293272) | 964 |
| CCL2 | REL(ENSP00000295025) | 427 |
| CCL2 | TLR3(ENSP00000296795) | 480 |
| CCL2 | NOS3(ENSP00000297494) | 540 |
| CCL2 | CTLA4(ENSP00000303939) | 273 |
| CCL2 | TLR10(ENSP00000308925) | 157 |
| CCL2 | NOS2(ENSP00000327251) | 424 |
| CCL2 | SOD2(ENSP00000337127) | 560 |
| CCL2 | CAV1(ENSP00000339191) | 241 |
| CCL2 | HLA-DRB1(ENSP00000353099) | 195 |
| CCL2 | PARP1(ENSP00000355759) | 844 |
| CCL2 | NPR1(ENSP00000357669) | 190 |
| CCL2 | FLNA(ENSP00000358866) | 210 |
| CCL2 | TLR4(ENSP00000363089) | 655 |
| CCL2 | HSPA1A(ENSP00000364802) | 185 |
| CCL2 | TNF(ENSP00000365290) | 339 |
| CCL2 | HLA-C(ENSP00000365402) | 307 |
| CCL2 | MTHFR(ENSP00000365775) | 160 |
| CCL2 | HLA-A(ENSP00000366005) | 176 |
| CCL2 | TLR7(ENSP00000370034) | 425 |
| CCL2 | TNF(ENSP00000372988) | 339 |
| CCL2 | HLA-A(ENSP00000388526) | 219 |
| CCL2 | TNF(ENSP00000389265) | 962 |
| CCL2 | TNF(ENSP00000389490) | 339 |
| CCL2 | TNF(ENSP00000389492) | 339 |
| CCL2 | TNF(ENSP00000392858) | 962 |
| CCL2 | TNF(ENSP00000398698) | 968 |
| CCL2 | HLA-B(ENSP00000399168) | 270 |
| CCL2 | HLA-B(ENSP00000400842) | 185 |
| CCL2 | HSPA1A(ENSP00000404524) | 196 |
| CCL2 | HSPA1A(ENSP00000406359) | 219 |
| CCL2 | GPX1(ENSP00000407375) | 317 |
| CCL2 | HLA-A(ENSP00000410645) | 200 |
| CCL2 | TNF(ENSP00000410668) | 339 |
| IL2 | ESR1(ENSP00000206249) | 371 |
| IL2 | MIF(ENSP00000215754) | 330 |
| IL2 | TLR8(ENSP00000218032) | 200 |
| IL2 | NFKB1(ENSP00000226574) | 290 |
| IL2 | IFNG(ENSP00000229135) | 989 |
| IL2 | POU4F3(ENSP00000230732) | 297 |
| IL2 | LTF(ENSP00000231751) | 335 |
| IL2 | TNFAIP3(ENSP00000237289) | 196 |
| IL2 | VHL(ENSP00000256474) | 270 |
| IL2 | IL1A(ENSP00000263339) | 647 |
| IL2 | IL1B(ENSP00000263341) | 929 |
| IL2 | SIK1(ENSP00000270162) | 187 |
| IL2 | CCL5(ENSP00000293272) | 350 |
| IL2 | REL(ENSP00000295025) | 540 |
| IL2 | TLR3(ENSP00000296795) | 272 |
| IL2 | NOS3(ENSP00000297494) | 260 |
| IL2 | AQP3(ENSP00000297991) | 200 |
| IL2 | CTLA4(ENSP00000303939) | 752 |
| IL2 | NOS2(ENSP00000327251) | 308 |
| IL2 | SOD2(ENSP00000337127) | 379 |
| IL2 | CAV1(ENSP00000339191) | 185 |
| IL2 | PTPN22(ENSP00000352833) | 157 |
| IL2 | HLA-DRB1(ENSP00000353099) | 374 |
| IL2 | PARP1(ENSP00000355759) | 859 |
| IL2 | NPR1(ENSP00000357669) | 241 |
| IL2 | FLNA(ENSP00000358866) | 200 |
| IL2 | TLR4(ENSP00000363089) | 412 |
| IL2 | TNF(ENSP00000365290) | 258 |
| IL2 | HLA-C(ENSP00000365402) | 611 |
| IL2 | HLA-A(ENSP00000366005) | 543 |
| IL2 | TLR7(ENSP00000370034) | 286 |
| IL2 | HLA-C(ENSP00000372975) | 371 |
| IL2 | TNF(ENSP00000372988) | 258 |
| IL2 | HLA-A(ENSP00000373114) | 515 |
| IL2 | HLA-C(ENSP00000383245) | 371 |
| IL2 | HLA-A(ENSP00000388526) | 750 |
| IL2 | HLA-A(ENSP00000388724) | 515 |
| IL2 | TNF(ENSP00000389265) | 957 |
| IL2 | TNF(ENSP00000389490) | 258 |
| IL2 | TNF(ENSP00000389492) | 258 |
| IL2 | HLA-C(ENSP00000390282) | 500 |
| IL2 | TNF(ENSP00000392858) | 958 |
| IL2 | HLA-C(ENSP00000397867) | 319 |
| IL2 | HLA-A(ENSP00000398188) | 514 |
| IL2 | TNF(ENSP00000398698) | 972 |
| IL2 | HLA-B(ENSP00000399168) | 926 |
| IL2 | HLA-B(ENSP00000400842) | 540 |
| IL2 | MICA(ENSP00000402134) | 191 |
| IL2 | HSPA1A(ENSP00000406359) | 189 |
| IL2 | GPX1(ENSP00000407375) | 243 |
| IL2 | HLA-C(ENSP00000407431) | 427 |
| IL2 | HLA-A(ENSP00000410645) | 540 |
| IL2 | TNF(ENSP00000410668) | 258 |
| IL2 | HLA-C(ENSP00000413992) | 540 |
| IL2 | HLA-A(ENSP00000416233) | 514 |
| CCND1 | ESR1(ENSP00000206249) | 999 |
| CCND1 | MIF(ENSP00000215754) | 238 |
| CCND1 | CHGA(ENSP00000216492) | 302 |
| CCND1 | NFKB1(ENSP00000226574) | 502 |
| CCND1 | IFNG(ENSP00000229135) | 366 |
| CCND1 | LTF(ENSP00000231751) | 202 |
| CCND1 | VHL(ENSP00000256474) | 874 |
| CCND1 | IL1A(ENSP00000263339) | 201 |
| CCND1 | IL1B(ENSP00000263341) | 329 |
| CCND1 | NOTCH3(ENSP00000263388) | 340 |
| CCND1 | REL(ENSP00000295025) | 649 |
| CCND1 | NOS3(ENSP00000297494) | 346 |
| CCND1 | NOS2(ENSP00000327251) | 165 |
| CCND1 | SOD2(ENSP00000337127) | 379 |
| CCND1 | CAV1(ENSP00000339191) | 866 |
| CCND1 | ESR2(ENSP00000343925) | 433 |
| CCND1 | MTR(ENSP00000355536) | 159 |
| CCND1 | PARP1(ENSP00000355759) | 902 |
| CCND1 | NPR1(ENSP00000357669) | 182 |
| CCND1 | TLR4(ENSP00000363089) | 235 |
| CCND1 | HSPA1A(ENSP00000364802) | 406 |
| CCND1 | TNF(ENSP00000365290) | 259 |
| CCND1 | MTHFR(ENSP00000365775) | 241 |
| CCND1 | AQP4(ENSP00000372654) | 196 |
| CCND1 | TNF(ENSP00000372988) | 259 |
| CCND1 | DIAPH1(ENSP00000381565) | 201 |
| CCND1 | HSPA1A(ENSP00000382915) | 350 |
| CCND1 | HLA-A(ENSP00000388526) | 160 |
| CCND1 | TNF(ENSP00000389265) | 909 |
| CCND1 | TNF(ENSP00000389490) | 259 |
| CCND1 | TNF(ENSP00000389492) | 259 |
| CCND1 | TNF(ENSP00000392858) | 910 |
| CCND1 | TNF(ENSP00000398698) | 922 |
| CCND1 | HLA-B(ENSP00000399168) | 201 |
| CCND1 | HSPA1A(ENSP00000404524) | 409 |
| CCND1 | HSPA1A(ENSP00000406359) | 420 |
| CCND1 | GPX1(ENSP00000407375) | 262 |
| CCND1 | HSPA1A(ENSP00000408907) | 350 |
| CCND1 | TNF(ENSP00000410668) | 259 |
| IL1R1 | ESR1(ENSP00000206249) | 180 |
| IL1R1 | MIF(ENSP00000215754) | 243 |
| IL1R1 | TLR8(ENSP00000218032) | 529 |
| IL1R1 | NFKB1(ENSP00000226574) | 920 |
| IL1R1 | IFNG(ENSP00000229135) | 425 |
| IL1R1 | LTF(ENSP00000231751) | 167 |
| IL1R1 | TNFAIP3(ENSP00000237289) | 252 |
| IL1R1 | IL1A(ENSP00000263339) | 996 |
| IL1R1 | IL1B(ENSP00000263341) | 999 |
| IL1R1 | CCL5(ENSP00000293272) | 374 |
| IL1R1 | REL(ENSP00000295025) | 429 |
| IL1R1 | TLR3(ENSP00000296795) | 752 |
| IL1R1 | CTLA4(ENSP00000303939) | 276 |
| IL1R1 | TLR10(ENSP00000308925) | 607 |
| IL1R1 | NOS2(ENSP00000327251) | 424 |
| IL1R1 | SOD2(ENSP00000337127) | 271 |
| IL1R1 | TLR4(ENSP00000363089) | 788 |
| IL1R1 | HLA-C(ENSP00000365402) | 180 |
| IL1R1 | TLR7(ENSP00000370034) | 613 |
| IL1R1 | TNF(ENSP00000389265) | 659 |
| IL1R1 | TNF(ENSP00000392858) | 659 |
| IL1R1 | TNF(ENSP00000398698) | 669 |
| IL1R1 | HLA-B(ENSP00000399168) | 160 |
| CCL4 | ESR1(ENSP00000206249) | 154 |
| CCL4 | MIF(ENSP00000215754) | 201 |
| CCL4 | TLR8(ENSP00000218032) | 317 |
| CCL4 | NFKB1(ENSP00000226574) | 846 |
| CCL4 | IFNG(ENSP00000229135) | 546 |
| CCL4 | TNFAIP3(ENSP00000237289) | 427 |
| CCL4 | IL1A(ENSP00000263339) | 468 |
| CCL4 | IL1B(ENSP00000263341) | 947 |
| CCL4 | CCL5(ENSP00000293272) | 550 |
| CCL4 | REL(ENSP00000295025) | 196 |
| CCL4 | TLR3(ENSP00000296795) | 372 |
| CCL4 | CTLA4(ENSP00000303939) | 266 |
| CCL4 | TLR10(ENSP00000308925) | 193 |
| CCL4 | NOS2(ENSP00000327251) | 305 |
| CCL4 | SOD2(ENSP00000337127) | 262 |
| CCL4 | FLNA(ENSP00000358866) | 227 |
| CCL4 | TLR4(ENSP00000363089) | 507 |
| CCL4 | HSPA1A(ENSP00000364802) | 195 |
| CCL4 | HLA-C(ENSP00000365402) | 213 |
| CCL4 | TLR7(ENSP00000370034) | 344 |
| CCL4 | HLA-A(ENSP00000388526) | 221 |
| CCL4 | TNF(ENSP00000389265) | 559 |
| CCL4 | TNF(ENSP00000392858) | 601 |
| CCL4 | TNF(ENSP00000398698) | 631 |
| CCL4 | HLA-B(ENSP00000399168) | 180 |
| CCL4 | HSPA1A(ENSP00000404524) | 193 |
| CCL4 | HSPA1A(ENSP00000406359) | 193 |
| CCL4 | HLA-A(ENSP00000410645) | 187 |
| KCNA5 | KCNQ4(ENSP00000262916) | 913 |
| KCNA5 | NOS3(ENSP00000297494) | 308 |
| KCNA5 | KCNE3(ENSP00000310557) | 412 |
| KCNA5 | SLC8A1(ENSP00000332931) | 288 |
| KCNA5 | SOD2(ENSP00000337127) | 196 |
| KCNA5 | KCNE1(ENSP00000337255) | 927 |
| KCNA5 | CAV1(ENSP00000339191) | 446 |
| KCNA5 | CACNA1A(ENSP00000353362) | 238 |
| KCNA5 | NPR1(ENSP00000357669) | 260 |
| KCNA5 | FLNA(ENSP00000358866) | 159 |
| NOTCH2 | ESR1(ENSP00000206249) | 240 |
| NOTCH2 | TNFAIP3(ENSP00000237289) | 160 |
| NOTCH2 | MTNR1B(ENSP00000257068) | 430 |
| NOTCH2 | NOTCH3(ENSP00000263388) | 905 |
| NOTCH2 | REL(ENSP00000295025) | 185 |
| NOTCH2 | TNF(ENSP00000365290) | 284 |
| NOTCH2 | TNF(ENSP00000372988) | 284 |
| NOTCH2 | TNF(ENSP00000389265) | 349 |
| NOTCH2 | TNF(ENSP00000389490) | 284 |
| NOTCH2 | TNF(ENSP00000389492) | 284 |
| NOTCH2 | TNF(ENSP00000392858) | 355 |
| NOTCH2 | TNF(ENSP00000398698) | 363 |
| NOTCH2 | MICA(ENSP00000402134) | 199 |
| NOTCH2 | TNF(ENSP00000410668) | 284 |
| IL6 | ESR1(ENSP00000206249) | 941 |
| IL6 | MIF(ENSP00000215754) | 611 |
| IL6 | CHGA(ENSP00000216492) | 273 |
| IL6 | TLR8(ENSP00000218032) | 517 |
| IL6 | PON1(ENSP00000222381) | 340 |
| IL6 | PON2(ENSP00000222572) | 159 |
| IL6 | NFKB1(ENSP00000226574) | 958 |
| IL6 | IFNG(ENSP00000229135) | 968 |
| IL6 | POU4F3(ENSP00000230732) | 219 |
| IL6 | LTF(ENSP00000231751) | 901 |
| IL6 | TNFAIP3(ENSP00000237289) | 530 |
| IL6 | GJB6(ENSP00000241124) | 235 |
| IL6 | VHL(ENSP00000256474) | 229 |
| IL6 | IL1A(ENSP00000263339) | 976 |
| IL6 | IL1B(ENSP00000263341) | 992 |
| IL6 | NOTCH3(ENSP00000263388) | 185 |
| IL6 | SIK1(ENSP00000270162) | 180 |
| IL6 | CCL5(ENSP00000293272) | 927 |
| IL6 | REL(ENSP00000295025) | 753 |
| IL6 | TLR3(ENSP00000296795) | 929 |
| IL6 | DEFB1(ENSP00000297439) | 162 |
| IL6 | NOS3(ENSP00000297494) | 917 |
| IL6 | AQP3(ENSP00000297991) | 241 |
| IL6 | CTLA4(ENSP00000303939) | 562 |
| IL6 | TLR10(ENSP00000308925) | 350 |
| IL6 | AQP1(ENSP00000311165) | 176 |
| IL6 | NOS2(ENSP00000327251) | 754 |
| IL6 | SOD2(ENSP00000337127) | 943 |
| IL6 | CAV1(ENSP00000339191) | 267 |
| IL6 | ESR2(ENSP00000343925) | 374 |
| IL6 | PTPN22(ENSP00000352833) | 204 |
| IL6 | HLA-DRB1(ENSP00000353099) | 341 |
| IL6 | PARP1(ENSP00000355759) | 467 |
| IL6 | NPR1(ENSP00000357669) | 347 |
| IL6 | CGA(ENSP00000358595) | 229 |
| IL6 | FLNA(ENSP00000358866) | 251 |
| IL6 | TLR4(ENSP00000363089) | 839 |
| IL6 | HSPA1A(ENSP00000364802) | 534 |
| IL6 | TNF(ENSP00000365290) | 340 |
| IL6 | HLA-C(ENSP00000365402) | 516 |
| IL6 | MTHFR(ENSP00000365775) | 374 |
| IL6 | HLA-A(ENSP00000366005) | 396 |
| IL6 | TLR7(ENSP00000370034) | 926 |
| IL6 | AQP4(ENSP00000372654) | 837 |
| IL6 | HLA-DQA1(ENSP00000372738) | 176 |
| IL6 | HLA-C(ENSP00000372975) | 235 |
| IL6 | TNF(ENSP00000372988) | 340 |
| IL6 | HLA-A(ENSP00000373114) | 307 |
| IL6 | HSPA1A(ENSP00000382915) | 337 |
| IL6 | HLA-C(ENSP00000383245) | 241 |
| IL6 | HLA-DQA1(ENSP00000387892) | 185 |
| IL6 | HLA-A(ENSP00000388526) | 460 |
| IL6 | HLA-A(ENSP00000388724) | 318 |
| IL6 | TNF(ENSP00000389265) | 980 |
| IL6 | TNF(ENSP00000389490) | 340 |
| IL6 | TNF(ENSP00000389492) | 340 |
| IL6 | HLA-C(ENSP00000390282) | 318 |
| IL6 | TNF(ENSP00000392858) | 980 |
| IL6 | HLA-C(ENSP00000397867) | 219 |
| IL6 | HLA-A(ENSP00000398188) | 319 |
| IL6 | TNF(ENSP00000398698) | 987 |
| IL6 | HLA-B(ENSP00000399168) | 515 |
| IL6 | HLA-B(ENSP00000400842) | 348 |
| IL6 | HLA-DQA1(ENSP00000401760) | 185 |
| IL6 | HSPA1A(ENSP00000404524) | 911 |
| IL6 | HSPA1A(ENSP00000406359) | 918 |
| IL6 | GPX1(ENSP00000407375) | 925 |
| IL6 | HLA-C(ENSP00000407431) | 241 |
| IL6 | HSPA1A(ENSP00000408907) | 337 |
| IL6 | HLA-DQA1(ENSP00000409127) | 180 |
| IL6 | HLA-A(ENSP00000410645) | 427 |
| IL6 | TNF(ENSP00000410668) | 340 |
| IL6 | HLA-C(ENSP00000413992) | 340 |
| IL6 | HLA-DQA1(ENSP00000414360) | 185 |
| IL6 | HLA-A(ENSP00000416233) | 306 |
| TLR2 | MIF(ENSP00000215754) | 317 |
| TLR2 | TLR8(ENSP00000218032) | 513 |
| TLR2 | NFKB1(ENSP00000226574) | 863 |
| TLR2 | IFNG(ENSP00000229135) | 563 |
| TLR2 | LTF(ENSP00000231751) | 369 |
| TLR2 | TNFAIP3(ENSP00000237289) | 332 |
| TLR2 | IL1A(ENSP00000263339) | 424 |
| TLR2 | IL1B(ENSP00000263341) | 754 |
| TLR2 | CCL5(ENSP00000293272) | 595 |
| TLR2 | REL(ENSP00000295025) | 431 |
| TLR2 | TLR3(ENSP00000296795) | 321 |
| TLR2 | DEFB1(ENSP00000297439) | 274 |
| TLR2 | NOS3(ENSP00000297494) | 200 |
| TLR2 | CTLA4(ENSP00000303939) | 340 |
| TLR2 | TLR10(ENSP00000308925) | 668 |
| TLR2 | TNIP1(ENSP00000317891) | 193 |
| TLR2 | NOS2(ENSP00000327251) | 893 |
| TLR2 | SOD2(ENSP00000337127) | 445 |
| TLR2 | HLA-DRB1(ENSP00000353099) | 200 |
| TLR2 | PARP1(ENSP00000355759) | 219 |
| TLR2 | NPR1(ENSP00000357669) | 193 |
| TLR2 | TLR4(ENSP00000363089) | 491 |
| TLR2 | HSPA1A(ENSP00000364802) | 219 |
| TLR2 | TNF(ENSP00000365290) | 258 |
| TLR2 | HLA-C(ENSP00000365402) | 272 |
| TLR2 | HLA-A(ENSP00000366005) | 227 |
| TLR2 | TLR7(ENSP00000370034) | 303 |
| TLR2 | TNF(ENSP00000372988) | 258 |
| TLR2 | HLA-A(ENSP00000373114) | 157 |
| TLR2 | HLA-A(ENSP00000388526) | 276 |
| TLR2 | HLA-A(ENSP00000388724) | 174 |
| TLR2 | TNF(ENSP00000389265) | 958 |
| TLR2 | TNF(ENSP00000389490) | 258 |
| TLR2 | TNF(ENSP00000389492) | 258 |
| TLR2 | TNF(ENSP00000392858) | 958 |
| TLR2 | HLA-A(ENSP00000398188) | 168 |
| TLR2 | TNF(ENSP00000398698) | 964 |
| TLR2 | HLA-B(ENSP00000399168) | 273 |
| TLR2 | HLA-B(ENSP00000400842) | 168 |
| TLR2 | HSPA1A(ENSP00000404524) | 318 |
| TLR2 | HSPA1A(ENSP00000406359) | 341 |
| TLR2 | HSPA1A(ENSP00000408907) | 800 |
| TLR2 | HLA-A(ENSP00000410645) | 241 |
| TLR2 | TNF(ENSP00000410668) | 258 |
| TLR2 | HLA-A(ENSP00000416233) | 163 |
| CD80 | ESR1(ENSP00000206249) | 191 |
| CD80 | TLR8(ENSP00000218032) | 317 |
| CD80 | NFKB1(ENSP00000226574) | 165 |
| CD80 | IFNG(ENSP00000229135) | 958 |
| CD80 | IL1A(ENSP00000263339) | 285 |
| CD80 | IL1B(ENSP00000263341) | 430 |
| CD80 | CCL5(ENSP00000293272) | 274 |
| CD80 | REL(ENSP00000295025) | 236 |
| CD80 | TLR3(ENSP00000296795) | 374 |
| CD80 | CTLA4(ENSP00000303939) | 999 |
| CD80 | NOS2(ENSP00000327251) | 207 |
| CD80 | HLA-DQA1(ENSP00000339398) | 229 |
| CD80 | ESR2(ENSP00000343925) | 212 |
| CD80 | PTPN22(ENSP00000352833) | 227 |
| CD80 | HLA-DRB1(ENSP00000353099) | 930 |
| CD80 | NPR1(ENSP00000357669) | 195 |
| CD80 | TLR4(ENSP00000363089) | 514 |
| CD80 | HLA-C(ENSP00000365402) | 609 |
| CD80 | HLA-A(ENSP00000366005) | 659 |
| CD80 | TLR7(ENSP00000370034) | 428 |
| CD80 | HLA-DQA1(ENSP00000372738) | 266 |
| CD80 | HLA-C(ENSP00000372975) | 427 |
| CD80 | HLA-A(ENSP00000373114) | 565 |
| CD80 | HLA-C(ENSP00000383245) | 409 |
| CD80 | HLA-A(ENSP00000388526) | 786 |
| CD80 | HLA-A(ENSP00000388724) | 565 |
| CD80 | TNF(ENSP00000389265) | 516 |
| CD80 | HLA-C(ENSP00000390282) | 468 |
| CD80 | TNF(ENSP00000392858) | 515 |
| CD80 | HLA-C(ENSP00000397867) | 425 |
| CD80 | HLA-A(ENSP00000398188) | 609 |
| CD80 | TNF(ENSP00000398698) | 609 |
| CD80 | HLA-B(ENSP00000399168) | 669 |
| CD80 | HLA-B(ENSP00000400842) | 613 |
| CD80 | MICA(ENSP00000402134) | 195 |
| CD80 | HLA-C(ENSP00000407431) | 516 |
| CD80 | HLA-A(ENSP00000410645) | 673 |
| CD80 | HLA-C(ENSP00000413992) | 504 |
| CD80 | HLA-A(ENSP00000416233) | 542 |
| STAT3 | ESR1(ENSP00000206249) | 814 |
| STAT3 | MIF(ENSP00000215754) | 241 |
| STAT3 | CHGA(ENSP00000216492) | 177 |
| STAT3 | TLR8(ENSP00000218032) | 201 |
| STAT3 | NFKB1(ENSP00000226574) | 733 |
| STAT3 | IFNG(ENSP00000229135) | 953 |
| STAT3 | LTF(ENSP00000231751) | 201 |
| STAT3 | TNFAIP3(ENSP00000237289) | 215 |
| STAT3 | VHL(ENSP00000256474) | 236 |
| STAT3 | IL1A(ENSP00000263339) | 340 |
| STAT3 | IL1B(ENSP00000263341) | 516 |
| STAT3 | NOTCH3(ENSP00000263388) | 382 |
| STAT3 | CCL5(ENSP00000293272) | 366 |
| STAT3 | REL(ENSP00000295025) | 462 |
| STAT3 | TLR3(ENSP00000296795) | 379 |
| STAT3 | NOS3(ENSP00000297494) | 425 |
| STAT3 | CTLA4(ENSP00000303939) | 332 |
| STAT3 | NOS2(ENSP00000327251) | 900 |
| STAT3 | SOD2(ENSP00000337127) | 884 |
| STAT3 | CAV1(ENSP00000339191) | 286 |
| STAT3 | ESR2(ENSP00000343925) | 229 |
| STAT3 | HLA-DRB1(ENSP00000353099) | 174 |
| STAT3 | PARP1(ENSP00000355759) | 430 |
| STAT3 | NPR1(ENSP00000357669) | 174 |
| STAT3 | TLR4(ENSP00000363089) | 902 |
| STAT3 | HSPA1A(ENSP00000364802) | 159 |
| STAT3 | HLA-C(ENSP00000365402) | 238 |
| STAT3 | HLA-A(ENSP00000366005) | 275 |
| STAT3 | TLR7(ENSP00000370034) | 852 |
| STAT3 | HLA-DQA1(ENSP00000372738) | 165 |
| STAT3 | HLA-A(ENSP00000388526) | 185 |
| STAT3 | TNF(ENSP00000389265) | 645 |
| STAT3 | TNF(ENSP00000392858) | 647 |
| STAT3 | TNF(ENSP00000398698) | 669 |
| STAT3 | HLA-B(ENSP00000399168) | 241 |
| STAT3 | MICA(ENSP00000402134) | 800 |
| STAT3 | HSPA1A(ENSP00000404524) | 229 |
| STAT3 | HSPA1A(ENSP00000406359) | 243 |
| STAT3 | GPX1(ENSP00000407375) | 229 |
| STAT3 | HLA-A(ENSP00000410645) | 162 |
| STAT3 | HLA-C(ENSP00000413992) | 188 |
| ICAM1 | ESR1(ENSP00000206249) | 319 |
| ICAM1 | MIF(ENSP00000215754) | 429 |
| ICAM1 | TLR8(ENSP00000218032) | 219 |
| ICAM1 | PON1(ENSP00000222381) | 201 |
| ICAM1 | NFKB1(ENSP00000226574) | 943 |
| ICAM1 | IFNG(ENSP00000229135) | 947 |
| ICAM1 | LTF(ENSP00000231751) | 340 |
| ICAM1 | TNFAIP3(ENSP00000237289) | 597 |
| ICAM1 | IL1A(ENSP00000263339) | 929 |
| ICAM1 | IL1B(ENSP00000263341) | 963 |
| ICAM1 | MTRR(ENSP00000264668) | 251 |
| ICAM1 | CCL5(ENSP00000293272) | 534 |
| ICAM1 | AQP5(ENSP00000293599) | 160 |
| ICAM1 | REL(ENSP00000295025) | 540 |
| ICAM1 | TLR3(ENSP00000296795) | 422 |
| ICAM1 | NOS3(ENSP00000297494) | 940 |
| ICAM1 | CTLA4(ENSP00000303939) | 563 |
| ICAM1 | TNIP1(ENSP00000317891) | 213 |
| ICAM1 | NOS2(ENSP00000327251) | 649 |
| ICAM1 | SOD2(ENSP00000337127) | 720 |
| ICAM1 | CAV1(ENSP00000339191) | 846 |
| ICAM1 | HLA-DQA1(ENSP00000339398) | 195 |
| ICAM1 | ESR2(ENSP00000343925) | 227 |
| ICAM1 | HLA-DRB1(ENSP00000353099) | 922 |
| ICAM1 | PARP1(ENSP00000355759) | 878 |
| ICAM1 | NPR1(ENSP00000357669) | 319 |
| ICAM1 | FLNA(ENSP00000358866) | 382 |
| ICAM1 | TLR4(ENSP00000363089) | 917 |
| ICAM1 | HSPA1A(ENSP00000364802) | 240 |
| ICAM1 | TNF(ENSP00000365290) | 338 |
| ICAM1 | HLA-C(ENSP00000365402) | 536 |
| ICAM1 | MTHFR(ENSP00000365775) | 249 |
| ICAM1 | HLA-A(ENSP00000366005) | 540 |
| ICAM1 | TLR7(ENSP00000370034) | 340 |
| ICAM1 | HLA-DQA1(ENSP00000372738) | 228 |
| ICAM1 | HLA-C(ENSP00000372975) | 922 |
| ICAM1 | TNF(ENSP00000372988) | 338 |
| ICAM1 | HLA-A(ENSP00000373114) | 420 |
| ICAM1 | HLA-C(ENSP00000383245) | 273 |
| ICAM1 | HLA-A(ENSP00000388526) | 951 |
| ICAM1 | HLA-A(ENSP00000388724) | 412 |
| ICAM1 | TNF(ENSP00000389265) | 967 |
| ICAM1 | TNF(ENSP00000389490) | 338 |
| ICAM1 | TNF(ENSP00000389492) | 338 |
| ICAM1 | HLA-C(ENSP00000390282) | 378 |
| ICAM1 | TNF(ENSP00000392858) | 969 |
| ICAM1 | HLA-C(ENSP00000397867) | 914 |
| ICAM1 | HLA-A(ENSP00000398188) | 412 |
| ICAM1 | TNF(ENSP00000398698) | 977 |
| ICAM1 | HLA-B(ENSP00000399168) | 515 |
| ICAM1 | HLA-B(ENSP00000400842) | 939 |
| ICAM1 | HSPA1A(ENSP00000404524) | 243 |
| ICAM1 | HSPA1A(ENSP00000406359) | 318 |
| ICAM1 | GPX1(ENSP00000407375) | 369 |
| ICAM1 | HLA-C(ENSP00000407431) | 317 |
| ICAM1 | HLA-A(ENSP00000410645) | 429 |
| ICAM1 | TNF(ENSP00000410668) | 338 |
| ICAM1 | HLA-C(ENSP00000413992) | 412 |
| ICAM1 | HLA-A(ENSP00000416233) | 419 |
| IL18 | ESR1(ENSP00000206249) | 201 |
| IL18 | MIF(ENSP00000215754) | 508 |
| IL18 | TLR8(ENSP00000218032) | 429 |
| IL18 | NFKB1(ENSP00000226574) | 918 |
| IL18 | IFNG(ENSP00000229135) | 994 |
| IL18 | LTF(ENSP00000231751) | 306 |
| IL18 | TNFAIP3(ENSP00000237289) | 195 |
| IL18 | IL1A(ENSP00000263339) | 619 |
| IL18 | IL1B(ENSP00000263341) | 994 |
| IL18 | CCL5(ENSP00000293272) | 532 |
| IL18 | REL(ENSP00000295025) | 340 |
| IL18 | TLR3(ENSP00000296795) | 639 |
| IL18 | NOS3(ENSP00000297494) | 340 |
| IL18 | AQP3(ENSP00000297991) | 180 |
| IL18 | CTLA4(ENSP00000303939) | 430 |
| IL18 | TLR10(ENSP00000308925) | 273 |
| IL18 | TNIP1(ENSP00000317891) | 168 |
| IL18 | NOS2(ENSP00000327251) | 434 |
| IL18 | SOD2(ENSP00000337127) | 340 |
| IL18 | PTPN22(ENSP00000352833) | 193 |
| IL18 | HLA-DRB1(ENSP00000353099) | 238 |
| IL18 | PARP1(ENSP00000355759) | 241 |
| IL18 | TLR4(ENSP00000363089) | 752 |
| IL18 | HSPA1A(ENSP00000364802) | 171 |
| IL18 | HLA-C(ENSP00000365402) | 374 |
| IL18 | HLA-A(ENSP00000366005) | 202 |
| IL18 | TLR7(ENSP00000370034) | 543 |
| IL18 | HLA-C(ENSP00000372975) | 204 |
| IL18 | HLA-C(ENSP00000383245) | 219 |
| IL18 | HLA-A(ENSP00000388526) | 307 |
| IL18 | HLA-A(ENSP00000388724) | 180 |
| IL18 | TNF(ENSP00000389265) | 752 |
| IL18 | HLA-C(ENSP00000390282) | 281 |
| IL18 | TNF(ENSP00000392858) | 752 |
| IL18 | HLA-C(ENSP00000397867) | 213 |
| IL18 | HLA-A(ENSP00000398188) | 180 |
| IL18 | TNF(ENSP00000398698) | 788 |
| IL18 | HLA-B(ENSP00000399168) | 306 |
| IL18 | HLA-B(ENSP00000400842) | 260 |
| IL18 | HSPA1A(ENSP00000406359) | 195 |
| IL18 | GPX1(ENSP00000407375) | 208 |
| IL18 | HLA-C(ENSP00000407431) | 218 |
| IL18 | HLA-A(ENSP00000410645) | 251 |
| IL18 | HLA-C(ENSP00000413992) | 246 |
| CCR5 | ESR1(ENSP00000206249) | 201 |
| CCR5 | MIF(ENSP00000215754) | 340 |
| CCR5 | TLR8(ENSP00000218032) | 377 |
| CCR5 | PON1(ENSP00000222381) | 213 |
| CCR5 | PON2(ENSP00000222572) | 266 |
| CCR5 | IFNG(ENSP00000229135) | 913 |
| CCR5 | LTF(ENSP00000231751) | 249 |
| CCR5 | VHL(ENSP00000256474) | 195 |
| CCR5 | MTNR1B(ENSP00000257068) | 899 |
| CCR5 | IL1A(ENSP00000263339) | 319 |
| CCR5 | IL1B(ENSP00000263341) | 572 |
| CCR5 | CCL5(ENSP00000293272) | 999 |
| CCR5 | REL(ENSP00000295025) | 285 |
| CCR5 | TLR3(ENSP00000296795) | 425 |
| CCR5 | NOS3(ENSP00000297494) | 243 |
| CCR5 | CTLA4(ENSP00000303939) | 505 |
| CCR5 | KCNE3(ENSP00000310557) | 245 |
| CCR5 | NOS2(ENSP00000327251) | 305 |
| CCR5 | SOD2(ENSP00000337127) | 219 |
| CCR5 | CAV1(ENSP00000339191) | 185 |
| CCR5 | HLA-DQA1(ENSP00000339398) | 193 |
| CCR5 | PTPN22(ENSP00000352833) | 199 |
| CCR5 | HLA-DRB1(ENSP00000353099) | 200 |
| CCR5 | PARP1(ENSP00000355759) | 156 |
| CCR5 | FLNA(ENSP00000358866) | 293 |
| CCR5 | TLR4(ENSP00000363089) | 907 |
| CCR5 | HSPA1A(ENSP00000364802) | 341 |
| CCR5 | HLA-C(ENSP00000365402) | 500 |
| CCR5 | HLA-A(ENSP00000366005) | 379 |
| CCR5 | TLR7(ENSP00000370034) | 472 |
| CCR5 | HLA-DQA1(ENSP00000372738) | 196 |
| CCR5 | HLA-C(ENSP00000372975) | 273 |
| CCR5 | HLA-A(ENSP00000373114) | 274 |
| CCR5 | HSPA1A(ENSP00000382915) | 335 |
| CCR5 | HLA-C(ENSP00000383245) | 279 |
| CCR5 | HLA-DQA1(ENSP00000387892) | 207 |
| CCR5 | HLA-A(ENSP00000388526) | 430 |
| CCR5 | HLA-A(ENSP00000388724) | 340 |
| CCR5 | TNF(ENSP00000389265) | 609 |
| CCR5 | HLA-C(ENSP00000390282) | 330 |
| CCR5 | TNF(ENSP00000392858) | 609 |
| CCR5 | HLA-C(ENSP00000397867) | 246 |
| CCR5 | HLA-A(ENSP00000398188) | 339 |
| CCR5 | TNF(ENSP00000398698) | 659 |
| CCR5 | HLA-B(ENSP00000399168) | 429 |
| CCR5 | HLA-B(ENSP00000400842) | 379 |
| CCR5 | HLA-DQA1(ENSP00000401760) | 207 |
| CCR5 | HSPA1A(ENSP00000404524) | 365 |
| CCR5 | HSPA1A(ENSP00000406359) | 374 |
| CCR5 | HLA-C(ENSP00000407431) | 286 |
| CCR5 | HSPA1A(ENSP00000408907) | 335 |
| CCR5 | HLA-DQA1(ENSP00000409127) | 199 |
| CCR5 | HLA-A(ENSP00000410645) | 379 |
| CCR5 | HLA-C(ENSP00000413992) | 249 |
| CCR5 | HLA-DQA1(ENSP00000414360) | 207 |
| CCR5 | HLA-A(ENSP00000416233) | 283 |
| VCAM1 | ESR1(ENSP00000206249) | 368 |
| VCAM1 | MIF(ENSP00000215754) | 379 |
| VCAM1 | PON1(ENSP00000222381) | 246 |
| VCAM1 | NFKB1(ENSP00000226574) | 311 |
| VCAM1 | IFNG(ENSP00000229135) | 916 |
| VCAM1 | LTF(ENSP00000231751) | 227 |
| VCAM1 | TNFAIP3(ENSP00000237289) | 219 |
| VCAM1 | IL1A(ENSP00000263339) | 901 |
| VCAM1 | IL1B(ENSP00000263341) | 927 |
| VCAM1 | NOTCH3(ENSP00000263388) | 154 |
| VCAM1 | CCL5(ENSP00000293272) | 482 |
| VCAM1 | REL(ENSP00000295025) | 508 |
| VCAM1 | TLR3(ENSP00000296795) | 317 |
| VCAM1 | NOS3(ENSP00000297494) | 669 |
| VCAM1 | CTLA4(ENSP00000303939) | 860 |
| VCAM1 | NOS2(ENSP00000327251) | 430 |
| VCAM1 | SOD2(ENSP00000337127) | 540 |
| VCAM1 | CAV1(ENSP00000339191) | 274 |
| VCAM1 | ESR2(ENSP00000343925) | 162 |
| VCAM1 | HLA-DRB1(ENSP00000353099) | 908 |
| VCAM1 | PARP1(ENSP00000355759) | 319 |
| VCAM1 | NPR1(ENSP00000357669) | 340 |
| VCAM1 | TLR4(ENSP00000363089) | 506 |
| VCAM1 | TNF(ENSP00000365290) | 335 |
| VCAM1 | HLA-C(ENSP00000365402) | 340 |
| VCAM1 | MTHFR(ENSP00000365775) | 235 |
| VCAM1 | HLA-A(ENSP00000366005) | 369 |
| VCAM1 | TLR7(ENSP00000370034) | 200 |
| VCAM1 | HLA-C(ENSP00000372975) | 910 |
| VCAM1 | TNF(ENSP00000372988) | 335 |
| VCAM1 | HLA-A(ENSP00000373114) | 235 |
| VCAM1 | HLA-C(ENSP00000383245) | 162 |
| VCAM1 | HLA-A(ENSP00000388526) | 926 |
| VCAM1 | HLA-A(ENSP00000388724) | 229 |
| VCAM1 | TNF(ENSP00000389265) | 962 |
| VCAM1 | TNF(ENSP00000389490) | 335 |
| VCAM1 | TNF(ENSP00000389492) | 335 |
| VCAM1 | HLA-C(ENSP00000390282) | 210 |
| VCAM1 | TNF(ENSP00000392858) | 962 |
| VCAM1 | HLA-C(ENSP00000397867) | 908 |
| VCAM1 | HLA-A(ENSP00000398188) | 229 |
| VCAM1 | TNF(ENSP00000398698) | 968 |
| VCAM1 | HLA-B(ENSP00000399168) | 379 |
| VCAM1 | HLA-B(ENSP00000400842) | 922 |
| VCAM1 | HSPA1A(ENSP00000406359) | 180 |
| VCAM1 | GPX1(ENSP00000407375) | 366 |
| VCAM1 | HLA-C(ENSP00000407431) | 165 |
| VCAM1 | HLA-A(ENSP00000410645) | 299 |
| VCAM1 | TNF(ENSP00000410668) | 335 |
| VCAM1 | HLA-C(ENSP00000413992) | 243 |
| VCAM1 | HLA-A(ENSP00000416233) | 236 |
| CSF2 | ESR1(ENSP00000206249) | 340 |
| CSF2 | MIF(ENSP00000215754) | 366 |
| CSF2 | TLR8(ENSP00000218032) | 306 |
| CSF2 | NFKB1(ENSP00000226574) | 919 |
| CSF2 | IFNG(ENSP00000229135) | 788 |
| CSF2 | LTF(ENSP00000231751) | 465 |
| CSF2 | TNFAIP3(ENSP00000237289) | 173 |
| CSF2 | GJB6(ENSP00000241124) | 202 |
| CSF2 | IL1A(ENSP00000263339) | 952 |
| CSF2 | IL1B(ENSP00000263341) | 954 |
| CSF2 | MTRR(ENSP00000264668) | 256 |
| CSF2 | CCL5(ENSP00000293272) | 536 |
| CSF2 | REL(ENSP00000295025) | 514 |
| CSF2 | TLR3(ENSP00000296795) | 462 |
| CSF2 | NOS3(ENSP00000297494) | 449 |
| CSF2 | AQP3(ENSP00000297991) | 180 |
| CSF2 | CTLA4(ENSP00000303939) | 563 |
| CSF2 | TLR10(ENSP00000308925) | 243 |
| CSF2 | NOS2(ENSP00000327251) | 542 |
| CSF2 | SOD2(ENSP00000337127) | 430 |
| CSF2 | HLA-DRB1(ENSP00000353099) | 270 |
| CSF2 | PARP1(ENSP00000355759) | 272 |
| CSF2 | NPR1(ENSP00000357669) | 201 |
| CSF2 | FLNA(ENSP00000358866) | 162 |
| CSF2 | TLR4(ENSP00000363089) | 609 |
| CSF2 | TNF(ENSP00000365290) | 335 |
| CSF2 | HLA-C(ENSP00000365402) | 901 |
| CSF2 | HLA-A(ENSP00000366005) | 468 |
| CSF2 | TLR7(ENSP00000370034) | 430 |
| CSF2 | HLA-C(ENSP00000372975) | 201 |
| CSF2 | TNF(ENSP00000372988) | 335 |
| CSF2 | HLA-A(ENSP00000373114) | 374 |
| CSF2 | DTNA(ENSP00000382064) | 344 |
| CSF2 | HLA-C(ENSP00000383245) | 196 |
| CSF2 | HLA-A(ENSP00000388526) | 649 |
| CSF2 | HLA-A(ENSP00000388724) | 369 |
| CSF2 | TNF(ENSP00000389265) | 962 |
| CSF2 | TNF(ENSP00000389490) | 335 |
| CSF2 | TNF(ENSP00000389492) | 335 |
| CSF2 | HLA-C(ENSP00000390282) | 317 |
| CSF2 | TNF(ENSP00000392858) | 962 |
| CSF2 | HLA-A(ENSP00000398188) | 374 |
| CSF2 | TNF(ENSP00000398698) | 977 |
| CSF2 | HLA-B(ENSP00000399168) | 517 |
| CSF2 | HLA-B(ENSP00000400842) | 378 |
| CSF2 | MICA(ENSP00000402134) | 307 |
| CSF2 | HSPA1A(ENSP00000404524) | 162 |
| CSF2 | HSPA1A(ENSP00000406359) | 160 |
| CSF2 | GPX1(ENSP00000407375) | 184 |
| CSF2 | HLA-C(ENSP00000407431) | 207 |
| CSF2 | HLA-A(ENSP00000410645) | 378 |
| CSF2 | TNF(ENSP00000410668) | 335 |
| CSF2 | HLA-C(ENSP00000413992) | 346 |
| CSF2 | HLA-A(ENSP00000416233) | 373 |
| CXCL10 | MIF(ENSP00000215754) | 317 |
| CXCL10 | TLR8(ENSP00000218032) | 429 |
| CXCL10 | NFKB1(ENSP00000226574) | 300 |
| CXCL10 | IFNG(ENSP00000229135) | 950 |
| CXCL10 | LTF(ENSP00000231751) | 180 |
| CXCL10 | TNFAIP3(ENSP00000237289) | 386 |
| CXCL10 | MTNR1B(ENSP00000257068) | 899 |
| CXCL10 | IL1A(ENSP00000263339) | 600 |
| CXCL10 | IL1B(ENSP00000263341) | 591 |
| CXCL10 | CCL5(ENSP00000293272) | 994 |
| CXCL10 | REL(ENSP00000295025) | 241 |
| CXCL10 | TLR3(ENSP00000296795) | 618 |
| CXCL10 | NOS3(ENSP00000297494) | 177 |
| CXCL10 | CTLA4(ENSP00000303939) | 318 |
| CXCL10 | TLR10(ENSP00000308925) | 291 |
| CXCL10 | NOS2(ENSP00000327251) | 335 |
| CXCL10 | SOD2(ENSP00000337127) | 273 |
| CXCL10 | TLR4(ENSP00000363089) | 613 |
| CXCL10 | HSPA1A(ENSP00000364802) | 193 |
| CXCL10 | HLA-C(ENSP00000365402) | 272 |
| CXCL10 | HLA-A(ENSP00000366005) | 201 |
| CXCL10 | TLR7(ENSP00000370034) | 564 |
| CXCL10 | HLA-A(ENSP00000373114) | 174 |
| CXCL10 | HLA-A(ENSP00000388526) | 371 |
| CXCL10 | HLA-A(ENSP00000388724) | 177 |
| CXCL10 | TNF(ENSP00000389265) | 649 |
| CXCL10 | HLA-C(ENSP00000390282) | 157 |
| CXCL10 | TNF(ENSP00000392858) | 650 |
| CXCL10 | HLA-A(ENSP00000398188) | 179 |
| CXCL10 | TNF(ENSP00000398698) | 674 |
| CXCL10 | HLA-B(ENSP00000399168) | 241 |
| CXCL10 | HLA-B(ENSP00000400842) | 180 |
| CXCL10 | HSPA1A(ENSP00000404524) | 193 |
| CXCL10 | HSPA1A(ENSP00000406359) | 193 |
| CXCL10 | HLA-A(ENSP00000410645) | 187 |
| CXCL10 | HLA-C(ENSP00000413992) | 195 |
| CXCL10 | HLA-A(ENSP00000416233) | 173 |
| FOS | AQP2(ENSP00000199280) | 297 |
| FOS | ESR1(ENSP00000206249) | 991 |
| FOS | MIF(ENSP00000215754) | 259 |
| FOS | CHGA(ENSP00000216492) | 272 |
| FOS | NFKB1(ENSP00000226574) | 744 |
| FOS | IFNG(ENSP00000229135) | 943 |
| FOS | LTF(ENSP00000231751) | 330 |
| FOS | TNFAIP3(ENSP00000237289) | 201 |
| FOS | GJB6(ENSP00000241124) | 201 |
| FOS | VHL(ENSP00000256474) | 213 |
| FOS | IL1A(ENSP00000263339) | 429 |
| FOS | IL1B(ENSP00000263341) | 982 |
| FOS | SIK1(ENSP00000270162) | 555 |
| FOS | CCL5(ENSP00000293272) | 838 |
| FOS | REL(ENSP00000295025) | 659 |
| FOS | TLR3(ENSP00000296795) | 260 |
| FOS | NOS3(ENSP00000297494) | 510 |
| FOS | AQP3(ENSP00000297991) | 180 |
| FOS | CTLA4(ENSP00000303939) | 219 |
| FOS | HCFC1(ENSP00000309555) | 180 |
| FOS | AQP1(ENSP00000311165) | 180 |
| FOS | NOS2(ENSP00000327251) | 347 |
| FOS | SOD2(ENSP00000337127) | 899 |
| FOS | CAV1(ENSP00000339191) | 288 |
| FOS | ESR2(ENSP00000343925) | 936 |
| FOS | HLA-DRB1(ENSP00000353099) | 905 |
| FOS | MTR(ENSP00000355536) | 265 |
| FOS | PARP1(ENSP00000355759) | 429 |
| FOS | NPR1(ENSP00000357669) | 413 |
| FOS | CGA(ENSP00000358595) | 337 |
| FOS | TLR4(ENSP00000363089) | 429 |
| FOS | HSPA1A(ENSP00000364802) | 305 |
| FOS | TNF(ENSP00000365290) | 258 |
| FOS | HLA-C(ENSP00000365402) | 170 |
| FOS | HLA-A(ENSP00000366005) | 156 |
| FOS | TLR7(ENSP00000370034) | 162 |
| FOS | AQP4(ENSP00000372654) | 288 |
| FOS | TNF(ENSP00000372988) | 258 |
| FOS | HLA-A(ENSP00000388526) | 204 |
| FOS | TNF(ENSP00000389265) | 695 |
| FOS | TNF(ENSP00000389490) | 258 |
| FOS | TNF(ENSP00000389492) | 258 |
| FOS | TNF(ENSP00000392858) | 722 |
| FOS | TNF(ENSP00000398698) | 957 |
| FOS | HLA-B(ENSP00000399168) | 272 |
| FOS | HSPA1A(ENSP00000404524) | 380 |
| FOS | HSPA1A(ENSP00000406359) | 446 |
| FOS | GPX1(ENSP00000407375) | 411 |
| FOS | HLA-A(ENSP00000410645) | 182 |
| FOS | TNF(ENSP00000410668) | 841 |
| FOS | HLA-C(ENSP00000413992) | 193 |
| IL8 | ESR1(ENSP00000206249) | 412 |
| IL8 | MIF(ENSP00000215754) | 524 |
| IL8 | CHGA(ENSP00000216492) | 157 |
| IL8 | TLR8(ENSP00000218032) | 318 |
| IL8 | NFKB1(ENSP00000226574) | 933 |
| IL8 | IFNG(ENSP00000229135) | 929 |
| IL8 | LTF(ENSP00000231751) | 536 |
| IL8 | TNFAIP3(ENSP00000237289) | 540 |
| IL8 | GJB6(ENSP00000241124) | 174 |
| IL8 | VHL(ENSP00000256474) | 240 |
| IL8 | IL1A(ENSP00000263339) | 946 |
| IL8 | IL1B(ENSP00000263341) | 978 |
| IL8 | CCL5(ENSP00000293272) | 679 |
| IL8 | REL(ENSP00000295025) | 543 |
| IL8 | TLR3(ENSP00000296795) | 894 |
| IL8 | DEFB1(ENSP00000297439) | 271 |
| IL8 | NOS3(ENSP00000297494) | 466 |
| IL8 | AQP3(ENSP00000297991) | 177 |
| IL8 | CTLA4(ENSP00000303939) | 200 |
| IL8 | TLR10(ENSP00000308925) | 263 |
| IL8 | NOS2(ENSP00000327251) | 340 |
| IL8 | SOD2(ENSP00000337127) | 722 |
| IL8 | CAV1(ENSP00000339191) | 201 |
| IL8 | HLA-DRB1(ENSP00000353099) | 243 |
| IL8 | PARP1(ENSP00000355759) | 340 |
| IL8 | NPR1(ENSP00000357669) | 268 |
| IL8 | FLNA(ENSP00000358866) | 201 |
| IL8 | TLR4(ENSP00000363089) | 941 |
| IL8 | HSPA1A(ENSP00000364802) | 219 |
| IL8 | TNF(ENSP00000365290) | 258 |
| IL8 | HLA-C(ENSP00000365402) | 340 |
| IL8 | MTHFR(ENSP00000365775) | 201 |
| IL8 | HLA-A(ENSP00000366005) | 212 |
| IL8 | TLR7(ENSP00000370034) | 369 |
| IL8 | AQP4(ENSP00000372654) | 241 |
| IL8 | TNF(ENSP00000372988) | 258 |
| IL8 | HLA-A(ENSP00000388526) | 238 |
| IL8 | HLA-A(ENSP00000388724) | 165 |
| IL8 | TNF(ENSP00000389265) | 958 |
| IL8 | TNF(ENSP00000389490) | 258 |
| IL8 | TNF(ENSP00000389492) | 258 |
| IL8 | HLA-C(ENSP00000390282) | 165 |
| IL8 | TNF(ENSP00000392858) | 958 |
| IL8 | HLA-C(ENSP00000397867) | 195 |
| IL8 | HLA-A(ENSP00000398188) | 163 |
| IL8 | TNF(ENSP00000398698) | 975 |
| IL8 | HLA-B(ENSP00000399168) | 330 |
| IL8 | HLA-B(ENSP00000400842) | 219 |
| IL8 | HSPA1A(ENSP00000404524) | 833 |
| IL8 | HSPA1A(ENSP00000406359) | 845 |
| IL8 | GPX1(ENSP00000407375) | 340 |
| IL8 | HLA-A(ENSP00000410645) | 262 |
| IL8 | TNF(ENSP00000410668) | 258 |
| CASP3 | ESR1(ENSP00000206249) | 540 |
| CASP3 | MIF(ENSP00000215754) | 243 |
| CASP3 | CHGA(ENSP00000216492) | 227 |
| CASP3 | NFKB1(ENSP00000226574) | 269 |
| CASP3 | IFNG(ENSP00000229135) | 515 |
| CASP3 | LTF(ENSP00000231751) | 219 |
| CASP3 | TNFAIP3(ENSP00000237289) | 179 |
| CASP3 | VHL(ENSP00000256474) | 201 |
| CASP3 | IL1A(ENSP00000263339) | 317 |
| CASP3 | IL1B(ENSP00000263341) | 906 |
| CASP3 | NOTCH3(ENSP00000263388) | 219 |
| CASP3 | ADD1(ENSP00000264758) | 960 |
| CASP3 | CCL5(ENSP00000293272) | 260 |
| CASP3 | AQP5(ENSP00000293599) | 172 |
| CASP3 | REL(ENSP00000295025) | 462 |
| CASP3 | TLR3(ENSP00000296795) | 317 |
| CASP3 | NOS3(ENSP00000297494) | 562 |
| CASP3 | CTLA4(ENSP00000303939) | 196 |
| CASP3 | NOS2(ENSP00000327251) | 427 |
| CASP3 | SLC8A1(ENSP00000332931) | 243 |
| CASP3 | SOD2(ENSP00000337127) | 750 |
| CASP3 | CAV1(ENSP00000339191) | 290 |
| CASP3 | ESR2(ENSP00000343925) | 308 |
| CASP3 | HLA-DRB1(ENSP00000353099) | 176 |
| CASP3 | CACNA1A(ENSP00000353362) | 229 |
| CASP3 | MTR(ENSP00000355536) | 157 |
| CASP3 | PARP1(ENSP00000355759) | 997 |
| CASP3 | NPR1(ENSP00000357669) | 375 |
| CASP3 | TLR4(ENSP00000363089) | 469 |
| CASP3 | HSPA1A(ENSP00000364802) | 385 |
| CASP3 | HLA-C(ENSP00000365402) | 183 |
| CASP3 | HLA-A(ENSP00000366005) | 177 |
| CASP3 | TLR7(ENSP00000370034) | 201 |
| CASP3 | AQP4(ENSP00000372654) | 229 |
| CASP3 | DIAPH1(ENSP00000381565) | 266 |
| CASP3 | HLA-A(ENSP00000388526) | 217 |
| CASP3 | TNF(ENSP00000389265) | 771 |
| CASP3 | TNF(ENSP00000392858) | 771 |
| CASP3 | TNF(ENSP00000398698) | 803 |
| CASP3 | HLA-B(ENSP00000399168) | 307 |
| CASP3 | HSPA1A(ENSP00000404524) | 430 |
| CASP3 | HSPA1A(ENSP00000406359) | 497 |
| CASP3 | GPX1(ENSP00000407375) | 562 |
| CD276 | MIF(ENSP00000215754) | 163 |
| CD276 | TLR8(ENSP00000218032) | 348 |
| CD276 | IFNG(ENSP00000229135) | 565 |
| CD276 | IL1A(ENSP00000263339) | 243 |
| CD276 | IL1B(ENSP00000263341) | 329 |
| CD276 | CCL5(ENSP00000293272) | 348 |
| CD276 | REL(ENSP00000295025) | 305 |
| CD276 | TLR3(ENSP00000296795) | 504 |
| CD276 | CTLA4(ENSP00000303939) | 955 |
| CD276 | TLR10(ENSP00000308925) | 196 |
| CD276 | NOS2(ENSP00000327251) | 249 |
| CD276 | HLA-DQA1(ENSP00000339398) | 243 |
| CD276 | PTPN22(ENSP00000352833) | 227 |
| CD276 | HLA-DRB1(ENSP00000353099) | 180 |
| CD276 | TLR4(ENSP00000363089) | 609 |
| CD276 | HLA-C(ENSP00000365402) | 232 |
| CD276 | HLA-A(ENSP00000366005) | 373 |
| CD276 | TLR7(ENSP00000370034) | 480 |
| CD276 | HLA-DQA1(ENSP00000372738) | 310 |
| CD276 | HLA-C(ENSP00000372975) | 219 |
| CD276 | HLA-A(ENSP00000373114) | 274 |
| CD276 | HLA-C(ENSP00000383245) | 219 |
| CD276 | HLA-DQA1(ENSP00000387892) | 177 |
| CD276 | HLA-A(ENSP00000388526) | 483 |
| CD276 | HLA-A(ENSP00000388724) | 267 |
| CD276 | TNF(ENSP00000389265) | 609 |
| CD276 | HLA-C(ENSP00000390282) | 290 |
| CD276 | TNF(ENSP00000392858) | 609 |
| CD276 | HLA-C(ENSP00000397867) | 200 |
| CD276 | HLA-A(ENSP00000398188) | 271 |
| CD276 | TNF(ENSP00000398698) | 649 |
| CD276 | HLA-B(ENSP00000399168) | 379 |
| CD276 | HLA-B(ENSP00000400842) | 265 |
| CD276 | HLA-DQA1(ENSP00000401760) | 177 |
| CD276 | HLA-C(ENSP00000407431) | 210 |
| CD276 | HLA-DQA1(ENSP00000409127) | 251 |
| CD276 | HLA-A(ENSP00000410645) | 305 |
| CD276 | HLA-C(ENSP00000413992) | 236 |
| CD276 | HLA-DQA1(ENSP00000414360) | 177 |
| CD276 | HLA-A(ENSP00000416233) | 274 |
| KCNA4 | KCNQ4(ENSP00000262916) | 909 |
| KCNA4 | KCNE3(ENSP00000310557) | 340 |
| KCNA4 | SLC8A1(ENSP00000332931) | 201 |
| KCNA4 | KCNE1(ENSP00000337255) | 527 |
| KCNA4 | CAV1(ENSP00000339191) | 336 |
| IRF7 | ESR1(ENSP00000206249) | 159 |
| IRF7 | TLR8(ENSP00000218032) | 609 |
| IRF7 | NFKB1(ENSP00000226574) | 273 |
| IRF7 | IFNG(ENSP00000229135) | 379 |
| IRF7 | TNFAIP3(ENSP00000237289) | 690 |
| IRF7 | IL1A(ENSP00000263339) | 165 |
| IRF7 | IL1B(ENSP00000263341) | 242 |
| IRF7 | CCL5(ENSP00000293272) | 596 |
| IRF7 | REL(ENSP00000295025) | 319 |
| IRF7 | TLR3(ENSP00000296795) | 755 |
| IRF7 | TLR10(ENSP00000308925) | 260 |
| IRF7 | NOS2(ENSP00000327251) | 200 |
| IRF7 | HLA-DRB1(ENSP00000353099) | 903 |
| IRF7 | PARP1(ENSP00000355759) | 195 |
| IRF7 | FLNA(ENSP00000358866) | 228 |
| IRF7 | TLR4(ENSP00000363089) | 613 |
| IRF7 | TNF(ENSP00000365290) | 258 |
| IRF7 | TLR7(ENSP00000370034) | 752 |
| IRF7 | HLA-C(ENSP00000372975) | 908 |
| IRF7 | TNF(ENSP00000372988) | 258 |
| IRF7 | HLA-A(ENSP00000388526) | 917 |
| IRF7 | TNF(ENSP00000389265) | 903 |
| IRF7 | TNF(ENSP00000389490) | 258 |
| IRF7 | TNF(ENSP00000389492) | 258 |
| IRF7 | HLA-C(ENSP00000390282) | 171 |
| IRF7 | TNF(ENSP00000392858) | 903 |
| IRF7 | HLA-C(ENSP00000397867) | 906 |
| IRF7 | TNF(ENSP00000398698) | 912 |
| IRF7 | HLA-B(ENSP00000399168) | 185 |
| IRF7 | HLA-B(ENSP00000400842) | 908 |
| IRF7 | HSPA1A(ENSP00000406359) | 804 |
| IRF7 | HLA-A(ENSP00000410645) | 188 |
| IRF7 | TNF(ENSP00000410668) | 258 |
| CD86 | ESR1(ENSP00000206249) | 168 |
| CD86 | MIF(ENSP00000215754) | 235 |
| CD86 | TLR8(ENSP00000218032) | 736 |
| CD86 | NFKB1(ENSP00000226574) | 176 |
| CD86 | IFNG(ENSP00000229135) | 992 |
| CD86 | LTF(ENSP00000231751) | 219 |
| CD86 | IL1A(ENSP00000263339) | 333 |
| CD86 | IL1B(ENSP00000263341) | 526 |
| CD86 | CCL5(ENSP00000293272) | 581 |
| CD86 | REL(ENSP00000295025) | 345 |
| CD86 | TLR3(ENSP00000296795) | 651 |
| CD86 | CTLA4(ENSP00000303939) | 999 |
| CD86 | TLR10(ENSP00000308925) | 318 |
| CD86 | NOS2(ENSP00000327251) | 339 |
| CD86 | SOD2(ENSP00000337127) | 179 |
| CD86 | CAV1(ENSP00000339191) | 842 |
| CD86 | HLA-DQA1(ENSP00000339398) | 228 |
| CD86 | ESR2(ENSP00000343925) | 193 |
| CD86 | PTPN22(ENSP00000352833) | 240 |
| CD86 | HLA-DRB1(ENSP00000353099) | 922 |
| CD86 | PARP1(ENSP00000355759) | 851 |
| CD86 | TLR4(ENSP00000363089) | 951 |
| CD86 | TNF(ENSP00000365290) | 258 |
| CD86 | HLA-C(ENSP00000365402) | 561 |
| CD86 | HLA-A(ENSP00000366005) | 653 |
| CD86 | TLR7(ENSP00000370034) | 803 |
| CD86 | HLA-DQA1(ENSP00000372738) | 369 |
| CD86 | HLA-C(ENSP00000372975) | 204 |
| CD86 | TNF(ENSP00000372988) | 258 |
| CD86 | HLA-A(ENSP00000373114) | 469 |
| CD86 | HLA-C(ENSP00000383245) | 204 |
| CD86 | HLA-A(ENSP00000388526) | 613 |
| CD86 | HLA-A(ENSP00000388724) | 463 |
| CD86 | TNF(ENSP00000389265) | 802 |
| CD86 | TNF(ENSP00000389490) | 258 |
| CD86 | TNF(ENSP00000389492) | 258 |
| CD86 | HLA-C(ENSP00000390282) | 344 |
| CD86 | TNF(ENSP00000392858) | 802 |
| CD86 | HLA-C(ENSP00000397867) | 174 |
| CD86 | HLA-A(ENSP00000398188) | 466 |
| CD86 | TNF(ENSP00000398698) | 958 |
| CD86 | HLA-B(ENSP00000399168) | 518 |
| CD86 | HLA-B(ENSP00000400842) | 463 |
| CD86 | HSPA1A(ENSP00000406359) | 162 |
| CD86 | HLA-C(ENSP00000407431) | 238 |
| CD86 | HLA-A(ENSP00000410645) | 465 |
| CD86 | TNF(ENSP00000410668) | 258 |
| CD86 | HLA-C(ENSP00000413992) | 462 |
| CD86 | HLA-A(ENSP00000416233) | 465 |
| GPX4 | ESR1(ENSP00000206249) | 299 |
| GPX4 | MIF(ENSP00000215754) | 349 |
| GPX4 | PON1(ENSP00000222381) | 319 |
| GPX4 | IFNG(ENSP00000229135) | 268 |
| GPX4 | LTF(ENSP00000231751) | 369 |
| GPX4 | IL1A(ENSP00000263339) | 185 |
| GPX4 | IL1B(ENSP00000263341) | 379 |
| GPX4 | REL(ENSP00000295025) | 219 |
| GPX4 | NOS3(ENSP00000297494) | 540 |
| GPX4 | NOS2(ENSP00000327251) | 369 |
| GPX4 | SOD2(ENSP00000337127) | 919 |
| GPX4 | MTR(ENSP00000355536) | 200 |
| GPX4 | PARP1(ENSP00000355759) | 378 |
| GPX4 | NPR1(ENSP00000357669) | 379 |
| GPX4 | TLR4(ENSP00000363089) | 196 |
| GPX4 | HSPA1A(ENSP00000364802) | 201 |
| GPX4 | MTHFR(ENSP00000365775) | 270 |
| GPX4 | AQP4(ENSP00000372654) | 196 |
| GPX4 | TNF(ENSP00000389265) | 433 |
| GPX4 | TNF(ENSP00000392858) | 461 |
| GPX4 | TNF(ENSP00000398698) | 540 |
| GPX4 | HSPA1A(ENSP00000404524) | 307 |
| GPX4 | HSPA1A(ENSP00000406359) | 341 |
| GPX4 | GPX1(ENSP00000407375) | 443 |
| TLR9 | MIF(ENSP00000215754) | 180 |
| TLR9 | TLR8(ENSP00000218032) | 910 |
| TLR9 | NFKB1(ENSP00000226574) | 219 |
| TLR9 | IFNG(ENSP00000229135) | 518 |
| TLR9 | LTF(ENSP00000231751) | 182 |
| TLR9 | TNFAIP3(ENSP00000237289) | 199 |
| TLR9 | IL1A(ENSP00000263339) | 369 |
| TLR9 | IL1B(ENSP00000263341) | 462 |
| TLR9 | CCL5(ENSP00000293272) | 462 |
| TLR9 | REL(ENSP00000295025) | 285 |
| TLR9 | TLR3(ENSP00000296795) | 927 |
| TLR9 | CTLA4(ENSP00000303939) | 424 |
| TLR9 | TLR10(ENSP00000308925) | 332 |
| TLR9 | NOS2(ENSP00000327251) | 340 |
| TLR9 | PTPN22(ENSP00000352833) | 191 |
| TLR9 | FLNA(ENSP00000358866) | 195 |
| TLR9 | TLR4(ENSP00000363089) | 325 |
| TLR9 | HLA-C(ENSP00000365402) | 260 |
| TLR9 | HLA-A(ENSP00000366005) | 170 |
| TLR9 | TLR7(ENSP00000370034) | 911 |
| TLR9 | HLA-A(ENSP00000388526) | 260 |
| TLR9 | TNF(ENSP00000389265) | 659 |
| TLR9 | TNF(ENSP00000392858) | 659 |
| TLR9 | TNF(ENSP00000398698) | 750 |
| TLR9 | HLA-B(ENSP00000399168) | 219 |
| TLR9 | HSPA1A(ENSP00000406359) | 162 |
| TLR9 | HLA-A(ENSP00000410645) | 185 |
| TLR9 | HLA-C(ENSP00000413992) | 180 |
| CXCL9 | ESR1(ENSP00000206249) | 160 |
| CXCL9 | MIF(ENSP00000215754) | 274 |
| CXCL9 | TLR8(ENSP00000218032) | 271 |
| CXCL9 | IFNG(ENSP00000229135) | 944 |
| CXCL9 | LTF(ENSP00000231751) | 160 |
| CXCL9 | MTNR1B(ENSP00000257068) | 899 |
| CXCL9 | IL1A(ENSP00000263339) | 305 |
| CXCL9 | IL1B(ENSP00000263341) | 440 |
| CXCL9 | CCL5(ENSP00000293272) | 986 |
| CXCL9 | REL(ENSP00000295025) | 301 |
| CXCL9 | TLR3(ENSP00000296795) | 435 |
| CXCL9 | NOS3(ENSP00000297494) | 180 |
| CXCL9 | CTLA4(ENSP00000303939) | 317 |
| CXCL9 | NOS2(ENSP00000327251) | 299 |
| CXCL9 | SOD2(ENSP00000337127) | 187 |
| CXCL9 | HLA-DRB1(ENSP00000353099) | 167 |
| CXCL9 | TLR4(ENSP00000363089) | 457 |
| CXCL9 | HLA-C(ENSP00000365402) | 243 |
| CXCL9 | HLA-A(ENSP00000366005) | 160 |
| CXCL9 | TLR7(ENSP00000370034) | 374 |
| CXCL9 | HLA-A(ENSP00000388526) | 280 |
| CXCL9 | TNF(ENSP00000389265) | 540 |
| CXCL9 | TNF(ENSP00000392858) | 542 |
| CXCL9 | TNF(ENSP00000398698) | 611 |
| CXCL9 | HLA-B(ENSP00000399168) | 229 |
| CXCL9 | HLA-B(ENSP00000400842) | 160 |
| CXCL9 | HLA-A(ENSP00000410645) | 160 |
| PTGS2 | AQP2(ENSP00000199280) | 319 |
| PTGS2 | ESR1(ENSP00000206249) | 917 |
| PTGS2 | MIF(ENSP00000215754) | 425 |
| PTGS2 | CHGA(ENSP00000216492) | 219 |
| PTGS2 | TLR8(ENSP00000218032) | 218 |
| PTGS2 | PON1(ENSP00000222381) | 190 |
| PTGS2 | NFKB1(ENSP00000226574) | 351 |
| PTGS2 | IFNG(ENSP00000229135) | 563 |
| PTGS2 | LTF(ENSP00000231751) | 238 |
| PTGS2 | TNFAIP3(ENSP00000237289) | 438 |
| PTGS2 | VHL(ENSP00000256474) | 235 |
| PTGS2 | IL1A(ENSP00000263339) | 918 |
| PTGS2 | IL1B(ENSP00000263341) | 976 |
| PTGS2 | NOTCH3(ENSP00000263388) | 202 |
| PTGS2 | SIK1(ENSP00000270162) | 215 |
| PTGS2 | CCL5(ENSP00000293272) | 373 |
| PTGS2 | REL(ENSP00000295025) | 540 |
| PTGS2 | TLR3(ENSP00000296795) | 866 |
| PTGS2 | NOS3(ENSP00000297494) | 937 |
| PTGS2 | AQP3(ENSP00000297991) | 201 |
| PTGS2 | CTLA4(ENSP00000303939) | 241 |
| PTGS2 | TLR10(ENSP00000308925) | 188 |
| PTGS2 | AQP1(ENSP00000311165) | 219 |
| PTGS2 | TNIP1(ENSP00000317891) | 168 |
| PTGS2 | NOS2(ENSP00000327251) | 794 |
| PTGS2 | SOD2(ENSP00000337127) | 712 |
| PTGS2 | CAV1(ENSP00000339191) | 975 |
| PTGS2 | ESR2(ENSP00000343925) | 379 |
| PTGS2 | PARP1(ENSP00000355759) | 514 |
| PTGS2 | NPR1(ENSP00000357669) | 429 |
| PTGS2 | TLR4(ENSP00000363089) | 946 |
| PTGS2 | HSPA1A(ENSP00000364802) | 285 |
| PTGS2 | TNF(ENSP00000365290) | 337 |
| PTGS2 | HLA-C(ENSP00000365402) | 236 |
| PTGS2 | MTHFR(ENSP00000365775) | 243 |
| PTGS2 | HLA-A(ENSP00000366005) | 180 |
| PTGS2 | TLR7(ENSP00000370034) | 263 |
| PTGS2 | AQP4(ENSP00000372654) | 168 |
| PTGS2 | HLA-C(ENSP00000372975) | 193 |
| PTGS2 | TNF(ENSP00000372988) | 337 |
| PTGS2 | HLA-C(ENSP00000383245) | 193 |
| PTGS2 | HLA-A(ENSP00000388526) | 177 |
| PTGS2 | HLA-A(ENSP00000388724) | 183 |
| PTGS2 | TNF(ENSP00000389265) | 962 |
| PTGS2 | TNF(ENSP00000389490) | 337 |
| PTGS2 | TNF(ENSP00000389492) | 337 |
| PTGS2 | HLA-C(ENSP00000390282) | 193 |
| PTGS2 | TNF(ENSP00000392858) | 962 |
| PTGS2 | HLA-C(ENSP00000397867) | 193 |
| PTGS2 | HLA-A(ENSP00000398188) | 183 |
| PTGS2 | TNF(ENSP00000398698) | 968 |
| PTGS2 | HLA-B(ENSP00000399168) | 241 |
| PTGS2 | HLA-B(ENSP00000400842) | 177 |
| PTGS2 | HSPA1A(ENSP00000404524) | 281 |
| PTGS2 | HSPA1A(ENSP00000406359) | 340 |
| PTGS2 | GPX1(ENSP00000407375) | 466 |
| PTGS2 | HLA-C(ENSP00000407431) | 193 |
| PTGS2 | HLA-A(ENSP00000410645) | 200 |
| PTGS2 | TNF(ENSP00000410668) | 337 |
| CD40 | ESR1(ENSP00000206249) | 249 |
| CD40 | MIF(ENSP00000215754) | 274 |
| CD40 | TLR8(ENSP00000218032) | 877 |
| CD40 | NFKB1(ENSP00000226574) | 930 |
| CD40 | IFNG(ENSP00000229135) | 768 |
| CD40 | LTF(ENSP00000231751) | 818 |
| CD40 | TNFAIP3(ENSP00000237289) | 440 |
| CD40 | IL1A(ENSP00000263339) | 643 |
| CD40 | IL1B(ENSP00000263341) | 654 |
| CD40 | CCL5(ENSP00000293272) | 533 |
| CD40 | REL(ENSP00000295025) | 907 |
| CD40 | TLR3(ENSP00000296795) | 611 |
| CD40 | NOS3(ENSP00000297494) | 307 |
| CD40 | CTLA4(ENSP00000303939) | 784 |
| CD40 | TLR10(ENSP00000308925) | 241 |
| CD40 | NOS2(ENSP00000327251) | 388 |
| CD40 | SOD2(ENSP00000337127) | 281 |
| CD40 | CAV1(ENSP00000339191) | 922 |
| CD40 | ESR2(ENSP00000343925) | 185 |
| CD40 | PTPN22(ENSP00000352833) | 378 |
| CD40 | HLA-DRB1(ENSP00000353099) | 271 |
| CD40 | PARP1(ENSP00000355759) | 319 |
| CD40 | TLR4(ENSP00000363089) | 947 |
| CD40 | HSPA1A(ENSP00000364802) | 419 |
| CD40 | TNF(ENSP00000365290) | 362 |
| CD40 | HLA-C(ENSP00000365402) | 516 |
| CD40 | HLA-A(ENSP00000366005) | 540 |
| CD40 | TLR7(ENSP00000370034) | 611 |
| CD40 | HLA-DQA1(ENSP00000372738) | 193 |
| CD40 | HLA-C(ENSP00000372975) | 165 |
| CD40 | TNF(ENSP00000372988) | 362 |
| CD40 | HLA-A(ENSP00000373114) | 374 |
| CD40 | HSPA1A(ENSP00000382915) | 339 |
| CD40 | HLA-C(ENSP00000383245) | 162 |
| CD40 | HLA-A(ENSP00000388526) | 543 |
| CD40 | HLA-A(ENSP00000388724) | 374 |
| CD40 | TNF(ENSP00000389265) | 963 |
| CD40 | TNF(ENSP00000389490) | 362 |
| CD40 | TNF(ENSP00000389492) | 362 |
| CD40 | HLA-C(ENSP00000390282) | 317 |
| CD40 | TNF(ENSP00000392858) | 963 |
| CD40 | HLA-A(ENSP00000398188) | 377 |
| CD40 | TNF(ENSP00000398698) | 969 |
| CD40 | HLA-B(ENSP00000399168) | 510 |
| CD40 | HLA-B(ENSP00000400842) | 374 |
| CD40 | HSPA1A(ENSP00000404524) | 438 |
| CD40 | HSPA1A(ENSP00000406359) | 460 |
| CD40 | HLA-C(ENSP00000407431) | 176 |
| CD40 | HSPA1A(ENSP00000408907) | 339 |
| CD40 | HLA-A(ENSP00000410645) | 374 |
| CD40 | TNF(ENSP00000410668) | 362 |
| CD40 | HLA-C(ENSP00000413992) | 428 |
| CD40 | HLA-A(ENSP00000416233) | 374 |
| MMP9 | ESR1(ENSP00000206249) | 515 |
| MMP9 | MIF(ENSP00000215754) | 878 |
| MMP9 | CHGA(ENSP00000216492) | 162 |
| MMP9 | TLR8(ENSP00000218032) | 183 |
| MMP9 | NFKB1(ENSP00000226574) | 915 |
| MMP9 | IFNG(ENSP00000229135) | 894 |
| MMP9 | LTF(ENSP00000231751) | 418 |
| MMP9 | TNFAIP3(ENSP00000237289) | 157 |
| MMP9 | VHL(ENSP00000256474) | 249 |
| MMP9 | IL1A(ENSP00000263339) | 896 |
| MMP9 | IL1B(ENSP00000263341) | 932 |
| MMP9 | NOTCH3(ENSP00000263388) | 176 |
| MMP9 | CCL5(ENSP00000293272) | 878 |
| MMP9 | REL(ENSP00000295025) | 394 |
| MMP9 | TLR3(ENSP00000296795) | 272 |
| MMP9 | NOS3(ENSP00000297494) | 906 |
| MMP9 | NOS2(ENSP00000327251) | 379 |
| MMP9 | SOD2(ENSP00000337127) | 496 |
| MMP9 | CAV1(ENSP00000339191) | 302 |
| MMP9 | ESR2(ENSP00000343925) | 274 |
| MMP9 | PARP1(ENSP00000355759) | 379 |
| MMP9 | NPR1(ENSP00000357669) | 229 |
| MMP9 | FLNA(ENSP00000358866) | 200 |
| MMP9 | TLR4(ENSP00000363089) | 524 |
| MMP9 | HSPA1A(ENSP00000364802) | 187 |
| MMP9 | TNF(ENSP00000365290) | 264 |
| MMP9 | HLA-C(ENSP00000365402) | 262 |
| MMP9 | MTHFR(ENSP00000365775) | 260 |
| MMP9 | TLR7(ENSP00000370034) | 165 |
| MMP9 | AQP4(ENSP00000372654) | 241 |
| MMP9 | TNF(ENSP00000372988) | 264 |
| MMP9 | TNF(ENSP00000389265) | 943 |
| MMP9 | TNF(ENSP00000389490) | 264 |
| MMP9 | TNF(ENSP00000389492) | 264 |
| MMP9 | TNF(ENSP00000392858) | 943 |
| MMP9 | TNF(ENSP00000398698) | 958 |
| MMP9 | HLA-B(ENSP00000399168) | 196 |
| MMP9 | HSPA1A(ENSP00000404524) | 185 |
| MMP9 | HSPA1A(ENSP00000406359) | 185 |
| MMP9 | GPX1(ENSP00000407375) | 334 |
| MMP9 | TNF(ENSP00000410668) | 264 |
| AR | AQP2(ENSP00000199280) | 201 |
| AR | ESR1(ENSP00000206249) | 701 |
| AR | MIF(ENSP00000215754) | 196 |
| AR | CHGA(ENSP00000216492) | 427 |
| AR | NFKB1(ENSP00000226574) | 663 |
| AR | IFNG(ENSP00000229135) | 201 |
| AR | LTF(ENSP00000231751) | 236 |
| AR | VHL(ENSP00000256474) | 162 |
| AR | IL1A(ENSP00000263339) | 170 |
| AR | IL1B(ENSP00000263341) | 241 |
| AR | NOTCH3(ENSP00000263388) | 193 |
| AR | REL(ENSP00000295025) | 318 |
| AR | NOS3(ENSP00000297494) | 377 |
| AR | HCFC1(ENSP00000309555) | 202 |
| AR | AQP1(ENSP00000311165) | 162 |
| AR | NOS2(ENSP00000327251) | 155 |
| AR | SOD2(ENSP00000337127) | 340 |
| AR | CAV1(ENSP00000339191) | 959 |
| AR | ESR2(ENSP00000343925) | 228 |
| AR | CACNA1A(ENSP00000353362) | 319 |
| AR | PARP1(ENSP00000355759) | 378 |
| AR | NPR1(ENSP00000357669) | 159 |
| AR | CGA(ENSP00000358595) | 167 |
| AR | FLNA(ENSP00000358866) | 870 |
| AR | HSPA1A(ENSP00000364802) | 667 |
| AR | TNF(ENSP00000365290) | 335 |
| AR | MTHFR(ENSP00000365775) | 183 |
| AR | TNF(ENSP00000372988) | 335 |
| AR | TNF(ENSP00000389265) | 904 |
| AR | TNF(ENSP00000389490) | 335 |
| AR | TNF(ENSP00000389492) | 335 |
| AR | TNF(ENSP00000392858) | 904 |
| AR | TNF(ENSP00000398698) | 913 |
| AR | HSPA1A(ENSP00000404524) | 260 |
| AR | HSPA1A(ENSP00000406359) | 260 |
| AR | GPX1(ENSP00000407375) | 219 |
| AR | TNF(ENSP00000410668) | 335 |
| HLA-DRB5 | IFNG(ENSP00000229135) | 301 |
| HLA-DRB5 | TNFAIP3(ENSP00000237289) | 319 |
| HLA-DRB5 | IL1A(ENSP00000263339) | 168 |
| HLA-DRB5 | IL1B(ENSP00000263341) | 245 |
| HLA-DRB5 | CCL5(ENSP00000293272) | 235 |
| HLA-DRB5 | AQP5(ENSP00000293599) | 187 |
| HLA-DRB5 | CTLA4(ENSP00000303939) | 499 |
| HLA-DRB5 | NOS2(ENSP00000327251) | 193 |
| HLA-DRB5 | SOD2(ENSP00000337127) | 245 |
| HLA-DRB5 | HLA-DQA1(ENSP00000339398) | 852 |
| HLA-DRB5 | ESR2(ENSP00000343925) | 193 |
| HLA-DRB5 | PTPN22(ENSP00000352833) | 669 |
| HLA-DRB5 | HLA-DRB1(ENSP00000353099) | 902 |
| HLA-DRB5 | TLR4(ENSP00000363089) | 180 |
| HLA-DRB5 | HLA-C(ENSP00000365402) | 287 |
| HLA-DRB5 | MTHFR(ENSP00000365775) | 180 |
| HLA-DRB5 | HLA-A(ENSP00000366005) | 265 |
| HLA-DRB5 | HLA-DQA1(ENSP00000372738) | 297 |
| HLA-DRB5 | HLA-C(ENSP00000372975) | 915 |
| HLA-DRB5 | HLA-A(ENSP00000373114) | 270 |
| HLA-DRB5 | HLA-C(ENSP00000383245) | 206 |
| HLA-DRB5 | HLA-DQA1(ENSP00000387892) | 266 |
| HLA-DRB5 | HLA-A(ENSP00000388526) | 923 |
| HLA-DRB5 | HLA-A(ENSP00000388724) | 272 |
| HLA-DRB5 | TNF(ENSP00000389265) | 374 |
| HLA-DRB5 | HLA-C(ENSP00000390282) | 214 |
| HLA-DRB5 | TNF(ENSP00000392858) | 377 |
| HLA-DRB5 | HLA-C(ENSP00000397867) | 916 |
| HLA-DRB5 | HLA-A(ENSP00000398188) | 272 |
| HLA-DRB5 | TNF(ENSP00000398698) | 461 |
| HLA-DRB5 | HLA-B(ENSP00000399168) | 264 |
| HLA-DRB5 | HLA-B(ENSP00000400842) | 922 |
| HLA-DRB5 | HLA-DQA1(ENSP00000401760) | 266 |
| HLA-DRB5 | MICA(ENSP00000402134) | 422 |
| HLA-DRB5 | GPX1(ENSP00000407375) | 200 |
| HLA-DRB5 | HLA-C(ENSP00000407431) | 210 |
| HLA-DRB5 | HLA-DQA1(ENSP00000409127) | 640 |
| HLA-DRB5 | HLA-A(ENSP00000410645) | 277 |
| HLA-DRB5 | HLA-C(ENSP00000413992) | 184 |
| HLA-DRB5 | HLA-DQA1(ENSP00000414360) | 267 |
| HLA-DRB5 | HLA-A(ENSP00000416233) | 273 |
| FOXP3 | ESR1(ENSP00000206249) | 218 |
| FOXP3 | MIF(ENSP00000215754) | 241 |
| FOXP3 | TLR8(ENSP00000218032) | 413 |
| FOXP3 | IFNG(ENSP00000229135) | 659 |
| FOXP3 | POU4F3(ENSP00000230732) | 283 |
| FOXP3 | IL1A(ENSP00000263339) | 160 |
| FOXP3 | IL1B(ENSP00000263341) | 368 |
| FOXP3 | NOTCH3(ENSP00000263388) | 168 |
| FOXP3 | CCL5(ENSP00000293272) | 430 |
| FOXP3 | REL(ENSP00000295025) | 936 |
| FOXP3 | TLR3(ENSP00000296795) | 422 |
| FOXP3 | CTLA4(ENSP00000303939) | 996 |
| FOXP3 | TLR10(ENSP00000308925) | 828 |
| FOXP3 | NOS2(ENSP00000327251) | 274 |
| FOXP3 | SOD2(ENSP00000337127) | 165 |
| FOXP3 | ESR2(ENSP00000343925) | 179 |
| FOXP3 | PTPN22(ENSP00000352833) | 319 |
| FOXP3 | HLA-DRB1(ENSP00000353099) | 201 |
| FOXP3 | PARP1(ENSP00000355759) | 188 |
| FOXP3 | NPR1(ENSP00000357669) | 195 |
| FOXP3 | TLR4(ENSP00000363089) | 563 |
| FOXP3 | TNF(ENSP00000365290) | 258 |
| FOXP3 | HLA-C(ENSP00000365402) | 461 |
| FOXP3 | HLA-A(ENSP00000366005) | 317 |
| FOXP3 | TLR7(ENSP00000370034) | 900 |
| FOXP3 | HLA-C(ENSP00000372975) | 180 |
| FOXP3 | TNF(ENSP00000372988) | 258 |
| FOXP3 | HLA-A(ENSP00000373114) | 274 |
| FOXP3 | HLA-C(ENSP00000383245) | 183 |
| FOXP3 | HLA-A(ENSP00000388526) | 465 |
| FOXP3 | HLA-A(ENSP00000388724) | 273 |
| FOXP3 | TNF(ENSP00000389265) | 941 |
| FOXP3 | TNF(ENSP00000389490) | 258 |
| FOXP3 | TNF(ENSP00000389492) | 258 |
| FOXP3 | HLA-C(ENSP00000390282) | 259 |
| FOXP3 | TNF(ENSP00000392858) | 940 |
| FOXP3 | HLA-A(ENSP00000398188) | 274 |
| FOXP3 | TNF(ENSP00000398698) | 942 |
| FOXP3 | HLA-B(ENSP00000399168) | 319 |
| FOXP3 | HLA-B(ENSP00000400842) | 305 |
| FOXP3 | MICA(ENSP00000402134) | 185 |
| FOXP3 | HLA-C(ENSP00000407431) | 180 |
| FOXP3 | HLA-A(ENSP00000410645) | 317 |
| FOXP3 | TNF(ENSP00000410668) | 258 |
| FOXP3 | HLA-C(ENSP00000413992) | 308 |
| FOXP3 | HLA-A(ENSP00000416233) | 267 |
| IL2RA | ESR1(ENSP00000206249) | 219 |
| IL2RA | MIF(ENSP00000215754) | 228 |
| IL2RA | TLR8(ENSP00000218032) | 180 |
| IL2RA | NFKB1(ENSP00000226574) | 706 |
| IL2RA | IFNG(ENSP00000229135) | 663 |
| IL2RA | LTF(ENSP00000231751) | 200 |
| IL2RA | TNFAIP3(ENSP00000237289) | 217 |
| IL2RA | IL1A(ENSP00000263339) | 500 |
| IL2RA | IL1B(ENSP00000263341) | 543 |
| IL2RA | CCL5(ENSP00000293272) | 340 |
| IL2RA | REL(ENSP00000295025) | 501 |
| IL2RA | TLR3(ENSP00000296795) | 201 |
| IL2RA | CTLA4(ENSP00000303939) | 763 |
| IL2RA | NOS2(ENSP00000327251) | 243 |
| IL2RA | SOD2(ENSP00000337127) | 243 |
| IL2RA | CAV1(ENSP00000339191) | 179 |
| IL2RA | HLA-DQA1(ENSP00000339398) | 156 |
| IL2RA | PTPN22(ENSP00000352833) | 467 |
| IL2RA | HLA-DRB1(ENSP00000353099) | 318 |
| IL2RA | PARP1(ENSP00000355759) | 229 |
| IL2RA | TLR4(ENSP00000363089) | 340 |
| IL2RA | HLA-C(ENSP00000365402) | 469 |
| IL2RA | HLA-A(ENSP00000366005) | 379 |
| IL2RA | TLR7(ENSP00000370034) | 229 |
| IL2RA | HLA-C(ENSP00000372975) | 165 |
| IL2RA | HLA-A(ENSP00000373114) | 364 |
| IL2RA | NAGA(ENSP00000379680) | 394 |
| IL2RA | HLA-C(ENSP00000383245) | 168 |
| IL2RA | HLA-A(ENSP00000388526) | 462 |
| IL2RA | HLA-A(ENSP00000388724) | 343 |
| IL2RA | TNF(ENSP00000389265) | 916 |
| IL2RA | HLA-C(ENSP00000390282) | 274 |
| IL2RA | TNF(ENSP00000392858) | 916 |
| IL2RA | HLA-C(ENSP00000397867) | 157 |
| IL2RA | HLA-A(ENSP00000398188) | 345 |
| IL2RA | TNF(ENSP00000398698) | 946 |
| IL2RA | HLA-B(ENSP00000399168) | 513 |
| IL2RA | HLA-B(ENSP00000400842) | 369 |
| IL2RA | MICA(ENSP00000402134) | 226 |
| IL2RA | GPX1(ENSP00000407375) | 193 |
| IL2RA | HLA-C(ENSP00000407431) | 190 |
| IL2RA | HLA-A(ENSP00000410645) | 380 |
| IL2RA | HLA-C(ENSP00000413992) | 340 |
| IL2RA | HLA-A(ENSP00000416233) | 348 |
| CXCL1 | ESR1(ENSP00000206249) | 200 |
| CXCL1 | MIF(ENSP00000215754) | 290 |
| CXCL1 | TLR8(ENSP00000218032) | 180 |
| CXCL1 | NFKB1(ENSP00000226574) | 274 |
| CXCL1 | IFNG(ENSP00000229135) | 427 |
| CXCL1 | LTF(ENSP00000231751) | 160 |
| CXCL1 | TNFAIP3(ENSP00000237289) | 610 |
| CXCL1 | MTNR1B(ENSP00000257068) | 899 |
| CXCL1 | IL1A(ENSP00000263339) | 904 |
| CXCL1 | IL1B(ENSP00000263341) | 793 |
| CXCL1 | CCL5(ENSP00000293272) | 973 |
| CXCL1 | REL(ENSP00000295025) | 306 |
| CXCL1 | TLR3(ENSP00000296795) | 375 |
| CXCL1 | NOS3(ENSP00000297494) | 170 |
| CXCL1 | CTLA4(ENSP00000303939) | 183 |
| CXCL1 | TLR10(ENSP00000308925) | 193 |
| CXCL1 | TNIP1(ENSP00000317891) | 178 |
| CXCL1 | NOS2(ENSP00000327251) | 243 |
| CXCL1 | SOD2(ENSP00000337127) | 492 |
| CXCL1 | PARP1(ENSP00000355759) | 195 |
| CXCL1 | TLR4(ENSP00000363089) | 521 |
| CXCL1 | HSPA1A(ENSP00000364802) | 162 |
| CXCL1 | TLR7(ENSP00000370034) | 305 |
| CXCL1 | TNF(ENSP00000389265) | 566 |
| CXCL1 | TNF(ENSP00000392858) | 566 |
| CXCL1 | TNF(ENSP00000398698) | 616 |
| CXCL1 | GPX1(ENSP00000407375) | 196 |
| MYD88 | ESR1(ENSP00000206249) | 154 |
| MYD88 | MIF(ENSP00000215754) | 204 |
| MYD88 | TLR8(ENSP00000218032) | 978 |
| MYD88 | NFKB1(ENSP00000226574) | 319 |
| MYD88 | IFNG(ENSP00000229135) | 554 |
| MYD88 | LTF(ENSP00000231751) | 219 |
| MYD88 | TNFAIP3(ENSP00000237289) | 305 |
| MYD88 | IL1A(ENSP00000263339) | 937 |
| MYD88 | IL1B(ENSP00000263341) | 990 |
| MYD88 | CCL5(ENSP00000293272) | 505 |
| MYD88 | REL(ENSP00000295025) | 465 |
| MYD88 | TLR3(ENSP00000296795) | 942 |
| MYD88 | NOS3(ENSP00000297494) | 170 |
| MYD88 | CTLA4(ENSP00000303939) | 317 |
| MYD88 | TLR10(ENSP00000308925) | 842 |
| MYD88 | TNIP1(ENSP00000317891) | 184 |
| MYD88 | NOS2(ENSP00000327251) | 983 |
| MYD88 | SOD2(ENSP00000337127) | 249 |
| MYD88 | PTPN22(ENSP00000352833) | 180 |
| MYD88 | PARP1(ENSP00000355759) | 180 |
| MYD88 | TLR4(ENSP00000363089) | 999 |
| MYD88 | HLA-C(ENSP00000365402) | 196 |
| MYD88 | TLR7(ENSP00000370034) | 954 |
| MYD88 | HLA-DQA1(ENSP00000387892) | 177 |
| MYD88 | HLA-A(ENSP00000388526) | 162 |
| MYD88 | TNF(ENSP00000389265) | 750 |
| MYD88 | TNF(ENSP00000392858) | 750 |
| MYD88 | TNF(ENSP00000398698) | 752 |
| MYD88 | HLA-B(ENSP00000399168) | 196 |
| MYD88 | HLA-DQA1(ENSP00000401760) | 177 |
| MYD88 | HSPA1A(ENSP00000404524) | 165 |
| MYD88 | HSPA1A(ENSP00000406359) | 229 |
| MYD88 | HLA-DQA1(ENSP00000409127) | 176 |
| MYD88 | HLA-A(ENSP00000410645) | 157 |
| MYD88 | HLA-DQA1(ENSP00000414360) | 177 |
| RELA | ESR1(ENSP00000206249) | 985 |
| RELA | MIF(ENSP00000215754) | 227 |
| RELA | TLR8(ENSP00000218032) | 180 |
| RELA | PON1(ENSP00000222381) | 170 |
| RELA | NFKB1(ENSP00000226574) | 999 |
| RELA | IFNG(ENSP00000229135) | 412 |
| RELA | TNFAIP3(ENSP00000237289) | 932 |
| RELA | IL1A(ENSP00000263339) | 318 |
| RELA | IL1B(ENSP00000263341) | 944 |
| RELA | CCL5(ENSP00000293272) | 867 |
| RELA | REL(ENSP00000295025) | 984 |
| RELA | TLR3(ENSP00000296795) | 932 |
| RELA | NOS3(ENSP00000297494) | 597 |
| RELA | AQP3(ENSP00000297991) | 305 |
| RELA | CTLA4(ENSP00000303939) | 174 |
| RELA | TNIP1(ENSP00000317891) | 217 |
| RELA | NOS2(ENSP00000327251) | 619 |
| RELA | SOD2(ENSP00000337127) | 412 |
| RELA | CAV1(ENSP00000339191) | 195 |
| RELA | HLA-DQA1(ENSP00000339398) | 185 |
| RELA | ESR2(ENSP00000343925) | 162 |
| RELA | PARP1(ENSP00000355759) | 991 |
| RELA | TLR4(ENSP00000363089) | 901 |
| RELA | HSPA1A(ENSP00000364802) | 157 |
| RELA | TLR7(ENSP00000370034) | 235 |
| RELA | HLA-DQA1(ENSP00000372738) | 184 |
| RELA | HLA-DQA1(ENSP00000387892) | 195 |
| RELA | TNF(ENSP00000389265) | 935 |
| RELA | TNF(ENSP00000392858) | 936 |
| RELA | TNF(ENSP00000398698) | 951 |
| RELA | HLA-B(ENSP00000399168) | 182 |
| RELA | HLA-DQA1(ENSP00000401760) | 195 |
| RELA | HSPA1A(ENSP00000406359) | 180 |
| RELA | GPX1(ENSP00000407375) | 202 |
| RELA | HLA-DQA1(ENSP00000409127) | 195 |
| RELA | TNF(ENSP00000410668) | 816 |
| RELA | HLA-DQA1(ENSP00000414360) | 195 |
| GPX5 | ESR1(ENSP00000206249) | 288 |
| GPX5 | MIF(ENSP00000215754) | 204 |
| GPX5 | PON1(ENSP00000222381) | 330 |
| GPX5 | IFNG(ENSP00000229135) | 266 |
| GPX5 | LTF(ENSP00000231751) | 340 |
| GPX5 | IL1A(ENSP00000263339) | 180 |
| GPX5 | IL1B(ENSP00000263341) | 379 |
| GPX5 | REL(ENSP00000295025) | 205 |
| GPX5 | NOS3(ENSP00000297494) | 540 |
| GPX5 | NOS2(ENSP00000327251) | 369 |
| GPX5 | SOD2(ENSP00000337127) | 919 |
| GPX5 | MTR(ENSP00000355536) | 201 |
| GPX5 | PARP1(ENSP00000355759) | 379 |
| GPX5 | NPR1(ENSP00000357669) | 379 |
| GPX5 | TLR4(ENSP00000363089) | 188 |
| GPX5 | HSPA1A(ENSP00000364802) | 183 |
| GPX5 | TNF(ENSP00000365290) | 336 |
| GPX5 | MTHFR(ENSP00000365775) | 260 |
| GPX5 | AQP4(ENSP00000372654) | 198 |
| GPX5 | TNF(ENSP00000372988) | 336 |
| GPX5 | TNF(ENSP00000389265) | 597 |
| GPX5 | TNF(ENSP00000389490) | 336 |
| GPX5 | TNF(ENSP00000389492) | 336 |
| GPX5 | TNF(ENSP00000392858) | 617 |
| GPX5 | TNF(ENSP00000398698) | 675 |
| GPX5 | HSPA1A(ENSP00000404524) | 302 |
| GPX5 | HSPA1A(ENSP00000406359) | 340 |
| GPX5 | TNF(ENSP00000410668) | 336 |
| IL10 | ESR1(ENSP00000206249) | 263 |
| IL10 | MIF(ENSP00000215754) | 468 |
| IL10 | TLR8(ENSP00000218032) | 306 |
| IL10 | NFKB1(ENSP00000226574) | 283 |
| IL10 | IFNG(ENSP00000229135) | 841 |
| IL10 | POU4F3(ENSP00000230732) | 308 |
| IL10 | LTF(ENSP00000231751) | 374 |
| IL10 | TNFAIP3(ENSP00000237289) | 207 |
| IL10 | IL1A(ENSP00000263339) | 620 |
| IL10 | IL1B(ENSP00000263341) | 782 |
| IL10 | CCL5(ENSP00000293272) | 463 |
| IL10 | REL(ENSP00000295025) | 393 |
| IL10 | TLR3(ENSP00000296795) | 557 |
| IL10 | NOS3(ENSP00000297494) | 874 |
| IL10 | CTLA4(ENSP00000303939) | 927 |
| IL10 | TLR10(ENSP00000308925) | 200 |
| IL10 | NOS2(ENSP00000327251) | 973 |
| IL10 | SOD2(ENSP00000337127) | 430 |
| IL10 | PTPN22(ENSP00000352833) | 200 |
| IL10 | HLA-DRB1(ENSP00000353099) | 340 |
| IL10 | PARP1(ENSP00000355759) | 290 |
| IL10 | NPR1(ENSP00000357669) | 213 |
| IL10 | FLNA(ENSP00000358866) | 201 |
| IL10 | TLR4(ENSP00000363089) | 951 |
| IL10 | HSPA1A(ENSP00000364802) | 201 |
| IL10 | TNF(ENSP00000365290) | 258 |
| IL10 | HLA-C(ENSP00000365402) | 906 |
| IL10 | MTHFR(ENSP00000365775) | 219 |
| IL10 | HLA-A(ENSP00000366005) | 379 |
| IL10 | TLR7(ENSP00000370034) | 428 |
| IL10 | HLA-DQA1(ENSP00000372738) | 179 |
| IL10 | HLA-C(ENSP00000372975) | 201 |
| IL10 | TNF(ENSP00000372988) | 258 |
| IL10 | HLA-A(ENSP00000373114) | 317 |
| IL10 | HLA-C(ENSP00000383245) | 204 |
| IL10 | HLA-A(ENSP00000388526) | 462 |
| IL10 | HLA-A(ENSP00000388724) | 319 |
| IL10 | TNF(ENSP00000389265) | 958 |
| IL10 | TNF(ENSP00000389490) | 258 |
| IL10 | TNF(ENSP00000389492) | 258 |
| IL10 | HLA-C(ENSP00000390282) | 340 |
| IL10 | TNF(ENSP00000392858) | 958 |
| IL10 | HLA-C(ENSP00000397867) | 195 |
| IL10 | HLA-A(ENSP00000398188) | 319 |
| IL10 | TNF(ENSP00000398698) | 976 |
| IL10 | HLA-B(ENSP00000399168) | 502 |
| IL10 | HLA-B(ENSP00000400842) | 340 |
| IL10 | MICA(ENSP00000402134) | 821 |
| IL10 | HSPA1A(ENSP00000404524) | 185 |
| IL10 | HSPA1A(ENSP00000406359) | 272 |
| IL10 | GPX1(ENSP00000407375) | 272 |
| IL10 | HLA-C(ENSP00000407431) | 229 |
| IL10 | HLA-A(ENSP00000410645) | 461 |
| IL10 | TNF(ENSP00000410668) | 258 |
| IL10 | HLA-C(ENSP00000413992) | 340 |
| IL10 | HLA-A(ENSP00000416233) | 308 |
